# Mouse–human co-clinical trials demonstrate superior anti-tumour effects of buparlisib (BKM120) and cetuximab combination in squamous cell carcinoma of head and neck

**DOI:** 10.1038/s41416-020-01074-2

**Published:** 2020-09-23

**Authors:** Hye Ryun Kim, Han Na Kang, Mi Ran Yun, Kwon Young Ju, Jae Woo Choi, Dong Min Jung, Kyoung Ho Pyo, Min Hee Hong, Myoung-Ju Ahn, Jong-Mu Sun, Han Sang Kim, Jinna Kim, Jinseon Yoo, Kyu Ryung Kim, Yoon Woo Koh, Se Heon Kim, Eun Chang Choi, Sun Ock Yoon, Hyo Sup Shim, Soonmyung Paik, Tae-Min Kim, Byoung Chul Cho

**Affiliations:** 1 Division of Medical Oncology, Yonsei Cancer Center, Yonsei University College of Medicine, Seoul, Korea; 2 JE-UK Institute for Cancer Research, JEUK Co., Ltd, Gumi-City, Kyungbuk Korea; 3 Severance Biomedical Science Institute, Yonsei University of College of Medicine, Yonsei Cancer Research Institute, Seoul, Korea; 4 Division of Hematology and Oncology, Samsung Medical Center, Sungkyunkwan University School of Medicine, Seoul, Korea; 5 Department of Radiology, Severance Hospital, Yonsei University College of Medicine, Seoul, Korea; 6 Department of Medical Informatics, College of Medicine, The Catholic University of Korea, Seoul, Korea; 7 Department of Otorhinolaryngology, Yonsei University College of Medicine, Seoul, Republic of Korea; 8 Department of Pathology, Yonsei University College of Medicine, Seoul, Republic of Korea; 9 Division of Pathology, NSABP, Pittsburgh, PA USA

**Keywords:** Head and neck cancer, Head and neck cancer

## Abstract

**Background:**

Recurrent and/or metastatic squamous cell carcinoma of head and neck (R/M SCCHN) is a common cancer with high recurrence and mortality. Current treatments have low response rates (RRs).

**Methods:**

Fifty-three patients with R/M SCCHN received continuous oral buparlisib. In parallel, patient-derived xenografts (PDXs) were established in mice to evaluate resistance mechanisms and efficacy of buparlisib/cetuximab combination. Baseline and on-treatment tumour genomes and transcriptomes were sequenced. Based on the integrated clinical and PDX data, 11 patients with progression under buparlisib monotherapy were treated with a combination of buparlisib and cetuximab.

**Results:**

For buparlisib monotherapy, disease control rate (DCR) was 49%, RR was 3% and median progression-free survival (PFS) and overall survival (OS) were 63 and 143 days, respectively. For combination therapy, DCR was 91%, RR was 18% and median PFS and OS were 111 and 206 days, respectively. Four PDX models were originated from patients enrolled in the current clinical trial. While buparlisib alone did not inhibit tumour growth, combination therapy achieved tumour inhibition in three of seven PDXs. Genes associated with apoptosis and cell-cycle arrest were expressed at higher levels with combination treatment than with buparlisib or cetuximab alone.

**Conclusions:**

The buparlisib/cetuximab combination has significant promise as a treatment strategy for R/M SCCHN.

**Clinical Trial Registration:**

NCT01527877.

## Background

Squamous cell carcinoma of head and neck (SCCHN) is the sixth most frequent cancer with a dismal prognosis and high mortality.^1^ Low survival, in combination with the significant toxicity of current treatment strategies, emphasises the necessity for novel therapies.^2^ In recurrent/metastatic SCCHN (R/M SCCHN), the only approved targeted therapy is cetuximab, a monoclonal antibody against the epidermal growth factor receptor (EGFR), with a response rate (RR) of 10–15%.^3^ Anti-programmed death 1 (PD-1) inhibitors, including pembrolizumab and nivolumab, were recently approved for SCCHN, which is refractory to platinum-based therapy. Although anti-PD-1 therapy showed improved outcomes over previous standard chemotherapies (taxane, methotrexate or cetuximab), objective responses have been reported in only 15–20% of patients.^4–6^

Recent genomic studies have suggested potential therapeutic opportunities. Genetic alterations of the phosphatidylinositol 3-kinase (PI3K)–mTOR cell signalling pathway are common, particularly gain-of-function mutations of PI3K catalytic subunit α and loss-of-function mutations of *PTEN*.^7,8^ Buparlisib (BKM120) is a novel, oral pan-PI3K inhibitor. Recently, buparlisib combined with paclitaxel showed improved efficacy in the treatment of R/M SCCHN patients over paclitaxel alone, suggesting the importance of PI3K inhibition.^9^ We therefore conducted a Phase 2 study of buparlisib in R/M SCCHN.

To overcome the limited predictive value of conventional preclinical models, patient-derived tumour xenograft (PDX) models are a promising advance in oncology.^10–12^ These models, created by direct implantation of the patient’s tumour into immunodeficient mice, preserve histologic and genetic characteristics of the original patient tumours. They have been shown to be predictive of clinical outcomes and are being used for translational research, preclinical drug screening and biomarker identification/validation.^13–15^ The mouse–human co-clinical trial is a new concept in which the treatment of interest is simultaneously tested in patients and PDX models derived from tumours of the patients enrolled in the clinical trial.^15^ This provides a platform to develop new combination treatments, identify predictive biomarkers and provide insights into resistance mechanisms.

We conducted a co-clinical trial mirroring an ongoing clinical study to identify predictive markers and optimal combinational strategies. We established PDX models that faithfully replicated the histologic, genomic and drug responses observed in the corresponding patients. We tested the efficacy of a combination of buparlisib and cetuximab on the PDX models. Based on the results, we revised the clinical trial protocol to treat patients with a combination of buparlisib and cetuximab, with promising results.

## Methods

### Study design

This was a multicentre, Phase 2 study of buparlisib (100 mg/day) monotherapy in patients with R/M SCCHN who had progressed on platinum-based chemotherapy. The primary endpoint was disease control rate (DCR). Secondary endpoints included RR, progression-free survival rate (PFS), overall survival (OS) and safety.

Patients with histologically confirmed R/M SCCHN were enrolled. Patients were at least 18 years old and had an Eastern Cooperative Oncology Group performance status (ECOG-PS) of 0–2, at least one measurable disease and documented progressive disease after platinum-based chemotherapy for R/M SCCHN. Patients received continuous oral buparlisib (100 mg/day) until disease progression, death or unacceptable adverse events (AEs). Treatment cycles were 28 days long. Drug doses were withheld and/or reduced for intolerable grade 2 or grade 3/4 toxic effects. A maximum of two dose-level reductions was permitted (80 mg, then 60 mg).

Response evaluations were defined according to RECIST 1.1 guidelines.^16^ Radiographic imaging was conducted at week 4, then at every 8 weeks thereafter until disease progression or when clinically indicated. Safety assessments included physical examinations, documentation of AEs and laboratory measurements on day 1 of each cycle. AEs were graded according to the Common Terminology Criteria for Adverse Events version 4.0. This study was conducted under approval by the institutional review boards of Severance Hospital and all patients provided informed consent.

We revised the treatment protocol to the combination for patients that progressed under buparlisib monotherapy. This revised protocol was approved by the institutional review boards of Severance Hospital. After protocol amendment, 11 patients were treated with buparlisib/cetuximab.

### PDX models

A total of seven tumour samples were obtained from four patients (YHIM-01, -02, -06 and 07) with R/M SCCHN treated with buparlisib in the Phase 2 trial and from three patients (YHIM-03, -04 and 05) with SCCHN who had undergone surgery. Tumours and paired peripheral blood samples were collected prior to initial buparlisib treatment.

Six- to 8-week-old female severe combined immunodeficient (NOG) and nude (nu/nu) mice (OrientBio, Seoul, Korea) were used as recipients. After completion of experiments, we sacrificed mice by inhalation of anaesthetics with CO_2_. All mice models were maintained in the specific pathogen-free facility of the Avison BioMedical Research Center (ABMRC) Animal Research Center at Yonsei University College of Medicine. All methods were performed in accordance with the guidelines of the Animal Research Committee of Yonsei University College of Medicine and were approved by the Association of Assessment and Accreditation of Laboratory Animal Care. Patient tumour samples were cut into ~3 mm cubes and implanted subcutaneously into six or seven mice for each patient. When tumours grew to 1.5 cm in diameter, they were excised, dissected into ~3-mm cubes and implanted into another set of mice by the same procedure. The passage harbouring the patient-derived material was termed F0, with subsequent generations numbered consecutively (F1, F2, etc.). The rest of the carcinoma was cryopreserved and processed for biological studies. Tumour cells from the third passage (F3) were expanded for the in vivo drug efficacy test.

### PDX-derived cell models

F3 tumours were excised and chopped and/or sliced. Spill-out cells were also collected. Samples were incubated with collagenase/dispase II for 1 h at 37 °C in phosphate-buffered saline (PBS) and pelleted at 1500 r.p.m. for 10 min. The cell pellet was gently resuspended in RPMI-1640 culture medium and cells were plated onto collagen-coated culture surfaces. Cultures were kept in a humidified 5% CO_2_ atmosphere at 37 °C. On the following day, cultures were washed twice with PBS (37 °C) to remove cell debris and nonadherent blood cells. Mouse Cell Depletion (MACS, 130-104-694) and Cancer Isolation (MACS, 130-108-339) Kits were used to enrich for human tumour cells. The EpCAM-positive cell subpopulation in primary cultures was analysed using flow cytometry. Successfully established models were free of stromal cells and maintained a >95% EpCAM-positive cell subpopulation. The medium was replenished with fresh medium every 3 days.

### In vivo drug tests

When tumour volumes reached 200–250 mm^3^, mice were segregated into treatment groups based on tumour volume, growth rate and mouse weight. Vehicle (5 mM citrate buffer) was administered to the control group. Ten mice per group were randomised and treated with vehicle or buparlisib (35 mg/kg, QD, oral) alone or in combination with cetuximab (10 mg/kg, Q3D, intraperitoneally).

Tumour dimensions were measured twice a week with a digital calliper and tumour volume was calculated by the following formula: tumour volume = [length × width^2^]/2. Percentage tumour growth inhibition [%TGI = 1 − (change of tumour volume in treatment group/change of tumour volume in control group) × 100] was used for the evaluation of anti-tumour efficacy.^15^

### Cell viability assays

Cells were seeded at 3000/well in 96-well clear-bottom microplates, incubated overnight and subsequently treated with drugs for 3 days. Cell viability was analysed using CellTiter-Glo according to the manufacturer’s protocol. IC_50_ values were calculated using v5.0 GraphPad Prism (GraphPad Software, Inc., La Jolla, CA, USA). Drugs used were cetuximab (Selleckchem A2000) and BKM120 (Selleckchem S2247). Combination indices (CIs) were calculated using the CacluSyn method.^17^ For colony formation assays, single cells were seeded onto 6-well plates at a density of 3000/well. After overnight attachment, cells were treated for 14 days. Medium including drugs was replaced every 3 days. After treatment, cells were washed with PBS, fixed in 4% paraformaldehyde in PBS for 10 min and stained with 0.5% crystal violet in 20% methanol for 20 min. To evaluate clonogenicity, images were captured using a flatbed scanner and cells were dissolved with 20% acetic acid in 20% methanol. The optical density of each well was read at 570 nm using a SpectraMax 250 microplate reader (Molecular Devices).^18^

### Immunoblot analysis

Cell lysates containing equal amounts of protein were separated by sodium dodecyl sulfate-polyacrylamide gel electrophoresis and transferred to a membrane, and then probed with primary and secondary antibodies. Signals were detected with SuperSignal™ West Pico Chemiluminescent Substrate (Thermo Fisher Scientific, MA, USA). Primary antibodies were against cyclin B (Cell Signaling Technology, cat. no. 122231), cyclin D (Cell Signaling Technology, cat. no. 8396), cyclin E (Santa Cruz Biotechnology, cat. no. 481), cleaved poly (ADP-ribose) polymerase (PARP) (Cell Signaling Technology, cat. no. 5625), cleaved caspase-3 (Cell Signaling Technology, cat. no. 9664), cleaved caspase-7 (Cell Signaling Technology, cat. no. 8438), BCL-2 (Cell Signaling Technology, cat. no. 2872), cleaved caspase-9 (Cell Signaling Technology, cat. no. 7237) and Myc (Cell Signaling Technology, cat. no. 9402). After washing, blots were probed with horseradish peroxidase-conjugated rabbit secondary antibodies (Cell Signaling Technology, Boston, MA, USA, cat. no. 7074). The membrane was stripped and re-probed with an anti-β-actin (Sigma-Aldrich, cat. no. A3854) antibody as an internal control.

### Histology

Tissues from all PDX models were harvested and fixed in 10% buffered formalin within 30 min of resection, and then processed by routine procedures after 24 h fixation. Sections were stained with haematoxylin and eosin (H&E) and reviewed by a pathologist to confirm SCCHN.

### Whole-exome sequencing (WES) and somatic variants

WES was used to identify somatic single-nucleotide variations or substitutions (SNVs) and short insertions/deletions (indels) by comparing the tumour genomes with matched normal genomes. To minimise false positives from xenocontamination, sequencing reads from host (mouse) genomes were filtered out from those from tumour (human) genomes using the Xenome software.^19^ Matched normal genomes were prepared from patients’ peripheral blood. Exome-captured DNA by Agilent SureSelect Human All Exome 50 Mb Kit (Agilent, USA) was used to prepare libraries and 100-bp paired-end sequencing was performed using an Illumina HiSeq2000 platform (Illumina, USA). Alignment of sequencing reads onto the human reference genome (hg19) was performed using BWA-MEM (Burrows–Wheeler aligner-mem).^20^ Local realignment and score recalibration of sequencing reads were performed using Genome Analysis ToolKit (GATK).^21^ SNVs and indels (somatic mutations, hereafter) were identified by MuTect and Indelocator,^22^ respectively, and collectively analysed as somatic mutations of PDTX tumours. Somatic copy number alterations (SCNAs) were identified by comparing the sequencing depth of tumour and matched normal genomes using Excavator.^23^

### Targeted deep-sequencing and germline variants

Genomic DNA from formalin-fixed paraffin-embedded (FFPE) or fresh tissue, prepared using the QIAamp DNA FFPE Tissue Kit (Qiagen) or DNeasy Blood and Tissue Kit (Qiagen), was screened for 244 cancer-related genes using a customised SureSelect Kit (Agilent Technologies, Santa Clara, CA, USA) (Supplementary Table S1). Targeted sequencing was performed using an Illumina HiSeq2000 with an average coverage depth of 500–1000×. The BWA^20^ with a hybrid human (hg19) and mouse (mm10) genome was used to separate 100-bp paired-end sequence reads at PDX-F2 into human and mouse reads. Each read was aligned to both genomes and the best-matched read of the human genome was established.^24^ After pre-processing, the GATK^21^ was used for mark duplication, local realignment and base quality-score recalibration of the reads. The GATK Haplotype caller was run to identify germline mutations. Poor-quality variants were filtered out using GATK VariantFiltration with the following criteria: read depth < 20, quality by depth < 2.0, Fisher strand > 60.0, root-mean-square mapping quality (MQ) < 20.0, MQRankSum < −12.5, ReadPosRankSum < −8.0 and mutant allele frequency (MAF) < 0.2. ANNOVAR^25^ was used for variant annotation, and the CIVIC^26^ and DoCM^27^ databases were used to annotate mutations that could be used for targeted therapy.

### RNA-sequencing-based gene expression profiling

To identify changes in gene expression, we used RNA-sequencing. A cDNA library was generated and 100 bp paired-end sequencing was performed using Illumina HiSeq2000 (Illumina, USA). Splice-aware alignment was conducted using the TopHat aligner.^28^ Gene-level summaries of expression levels into fragments per kilobase million were performed using CuffLinks software.^28^

### Visualisation

Oncoprint heatmaps were drawn for the patterns of overall mutations using the ComplexHeatmap.^29^ Lollipop plots were created for frequently mutated genes using MAFtools,^30^ which offers a multitude of analysis and visualisation modules that are commonly used in cancer genomic studies, to identify the recurrence of genomic loci with variants.

### Statistical analyses

A Simon’s two-stage design was used to test the null hypothesis (P0) with a 10% significance level that the objective response rate (ORR) is ≤30% vs. the alternative hypothesis (P1) that the ORR is ≥45%. The expected sample size is 48 patients to provide 80% power to reject P0 when the true ORR is 45%. Twenty-three will be accrued during stage I, and if six or fewer responses are observed in the first stage, the trial is stopped early. Allowing for a follow-up loss rate of 10%, the total sample size is 53 patients. Based on the preclinical data, protocol amendment was in progress and at that time 42 patients were enrolled. After protocol amendment, additional 11 eligible patients were treated with buparlisib + cetuximab after failure to buparlisib.

Results are presented as the mean ± standard deviation of at least three experiments for each group. Statistical differences were determined using analysis of variance and the Wilcoxon’s rank-sum test for independent samples. Kaplan–Meier method was used to depict survival distribution, and the log-rank test was used for comparison. DCR was defined as the percentage of patients showing complete response (CR), partial response (PR) and stable disease (SD) to treatment based on RECIST 1.1. RR was defined as the percentage of patients who had achieved CR and PR based on RECIST 1.1. PFS was defined as the time from the initiation of therapy until evidence of disease progression or death. OS was defined as the time from the initiation of therapy until death from any cause. A *P* value (*P*) < 0.05 was considered statistically significant.

## Results

### Clinical outcomes

A total of 53 patients were enrolled, of which 7 were excluded due to rapid progression or withdrawal. Patient characteristics included median age 55 years (range, 31–82); male (85%); ECOG performance status 0/1/2 (11%/76%/13%); locoregional/metastatic/both (30%/32%/38%); oral cavity/oropharynx/larynx primary (36%/30%/13%); prior chemotherapy regimens 1/≥2 (38%/62%; Table 1).

**Table 1:** Baseline patient characteristics.

Characteristic	No. of patients	%
Sex		
Male	45	85
Female	8	15
Age (years)		
Median (range)	55	(31–82)
Performance status		
0	7	11
1	39	76
2	7	13
Smoking history		
Never smoker	16	37
Smoker	37	63
Time from initial diagnosis to study entry (month)		
Median (range)	39	(5–159)
Primary site		
Oral cavity	19	36
Oropharynx	16	30
Hypopharynx	5	9
Larynx	7	13
Sinus (nasal, maxillary and ethmoid)	6	11
Disease status at study entry		
Locoregional	16	30
Distant	17	32
Both	20	38
Number of prior chemotherapy regimens		
1	20	38
≥2	33	62

**Fig. 1:**
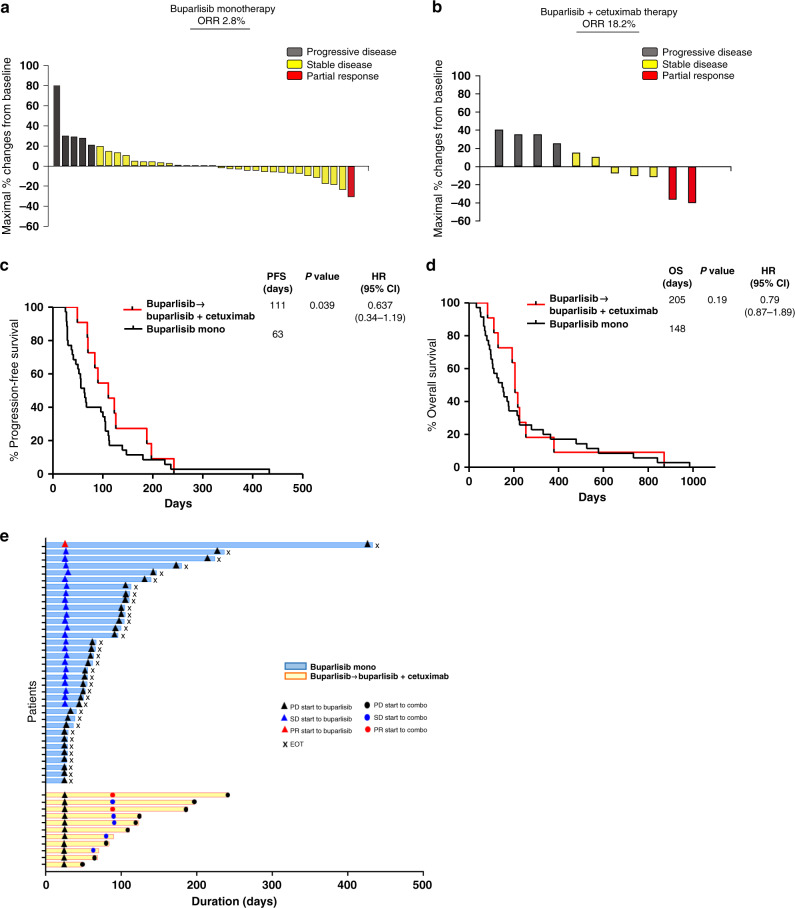
Clinical response the patients enrolled in clinical trial. **a**, **b** Best tumour volume change from baseline in patients with at least one post-baseline measurement **a** for buparlisib monotherapy, and **b** for the buparlisib/cetuximab combination phase. **c** Kaplan–Meier curves for progression-free survival. (**d**) Kaplan–Meier curve for overall survival. (**e**) Duration of response.

In the buparlisib monotherapy phase, 35 patients were evaluated. DCR was 49% and RR was 3% (Fig. 1a). After protocol revision, 11 patients who showed progression under buparlisib monotherapy were treated with the combination of buparlisib and cetuximab. In the combination phase, DCR was 91% and RR was 18% (Fig. 1b). Median PFS for buparlisib, followed by combination therapy was significantly longer than that for buparlisib monotherapy (63 and 111 days, *P* = 0.039; Fig. 1c). Median OS for buparlisib followed by combination therapy tended to be prolonged compared to that for buparlisib monotherapy (148 and 205 days, *P* = 0.19; Fig. 1d).

Treatment-related AEs are summarised in Supplementary Table S2. Grade 3–4 AEs were reported in 18 of 35 patients in the buparlisib monotherapy group and 4 of 11 patients in the combination phase. The most common grade 3–4 AEs were hyperglycaemia (10 in the buparlisib monotherapy vs. 3 in combination phase). Treatment discontinuation for AEs occurred in 19 patients in buparlisib and 3 in combination phase. Treatment-related toxicities were not significantly increased by the combination.

### Genomic fidelity of PDX tumours in terms of somatic mutations

We performed targeted deep sequencing to detect somatic mutations in seven original tumour (F0) and PDX-F2 tumour pairs to determine whether F0 and F2 samples would exhibit identical mutations. The coincidence of most germline mutations indicated that F0 and F2 samples were nearly identical, with Jaccard similarity index scores of >80% for most.^30^ Of the F0 germline mutations identified in the F2 specimens, YHIM-02 and YHIM-01 received the highest scores of 94% and 92%, respectively (Supplementary Fig. S1). The MAF values for F0-common and F2-common mutations exhibited overall concordance (*R* = 0.89 and 0.95 for YHIM-01 and YHIM-02, respectively), suggesting that most mutations in the F2 samples corresponded with those in the F0 samples (Supplementary Fig. S1).

### Faithful replication of clinical responses to buparlisib and cetuximab in PDX models

We established YHIM-01, -02, -06 and -07 directly from advanced R/M SCCHN patients who were treated in this Phase 2 trial. In addition, YHIM-03, -04 and -05 were established from surgically resected or biopsied SCCHN patients (Supplementary Table S3).

Tissue sections from these models were characterised using H&E staining and p63 immunohistochemistry (IHC; Supplementary Fig. S2). The histology of PDX tumours (F2) matched well with that of primary tumours (F0) by both methods. Xenografts expressed squamoid features, including keratinisation and intercellular bridges, found in the original tumours. In seven established F3 generation PDXs, we tested buparlisib or cetuximab alone, or in combination.

**Fig. 2:**
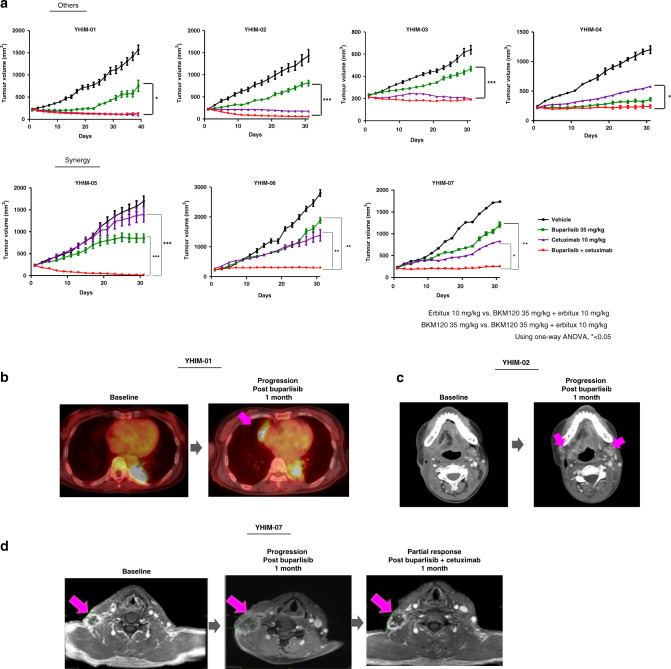
Drug responses of PDX models and corresponding patients. **a** Buparlisib, cetuximab and buparlisib/cetuximab tested in PDX models. **b** Correlation between patient YHIM-01 and PDX treated with buparlisib. **c** Rapid progression of patient YHIM-02 and PDX under buparlisib monotherapy. **d** Response of patient YHIM-07 to buparlisib/cetuximab therapy after progression under buparlisib monotherapy. After one cycle of buparlisib, the neck node progressed; however, it improved after the addition of cetuximab. Pink arrowhead indicates the measurable target tumour lesions.

To evaluate whether preclinical responses in PDX models mimicked the clinical response in patients, we treated YHIM-01, -02, -06 and -07 with buparlisib, in parallel with the corresponding R/M SCCHN patients from whom the PDX tumours were derived, in a prospective trial. We observed that preclinical responses to buparlisib in YHIM-01, -02, -06 and -07 precisely replicated the clinical responses of their corresponding patients (Fig. 2a–c). All seven established PDX models had a strong resistance to buparlisib monotherapy, displaying rapid tumour progression within a period of 30 days. Furthermore, marked tumour regression was observed in buparlisib/cetuximab combination therapy compared with buparlisib monotherapy in all PDX models. Notably, YHIM-05, -06 and -07 demonstrated strong resistance to each monotherapy, but treatment with buparlisib/cetuximab showed more prominently and synergistically inhibited the tumour growth than each buparlisib or cetuximab monotherapy [the average TGI (%) in YHIM-05, 114.92% of buparlisib/cetuximab vs. 58.59% of buparlisib (*P* < 0.0001) and vs. 20.65% of cetuximab (*P* < 0.0001); in YHIM-06, 98.01% of buparlisib/cetuximab vs. 42.37% of buparlisib (*P* < 0.001) and vs. 78.63% of cetuximab (*P* < 0.001); in YHIM-07, 102.09% of buparlisib/cetuximab vs. 52.24% of buparlisib (*P* < 0.001) and vs. 76.72% of cetuximab (*P* < 0.05)] (Fig. 2a and Supplementary Table S4). Data from the PDX and clinical trials were comprehensively integrated to identify predictive biomarkers of buparlisib.

Based on the strong synergy observed in the PDX models, we revised the treatment protocol to the combination for cases that progressed under buparlisib monotherapy. After protocol revision, 11 patients were treated with buparlisib/cetuximab. In the combination phase, PR was observed in 18% and SD was 46% in patients who had failed to respond to buparlisib (Fig. 1b).

Interestingly, the patient corresponding to YHIM-07 was enrolled and treated with buparlisib/cetuximab after progression under buparlisib monotherapy based on the revised protocol. Indeed, the response of this patient was similar to that of YHIM-07 (Fig. 2d). After one cycle of buparlisib, the neck node showed progression; however, after adding cetuximab, the neck node improved, showing PR (Fig. 2d). Thus, this patient showed strong synergistic inhibition with the combination, recapitulating the responses observed in the corresponding PDX model. Taken together, faithful replication of the clinical efficacy of buparlisib/cetuximab in PDX highlights the potential of co-clinical trials to inform and predict clinical outcomes.

### Analysis of somatic mutation profiles in PDX tumours

**Fig. 3:**
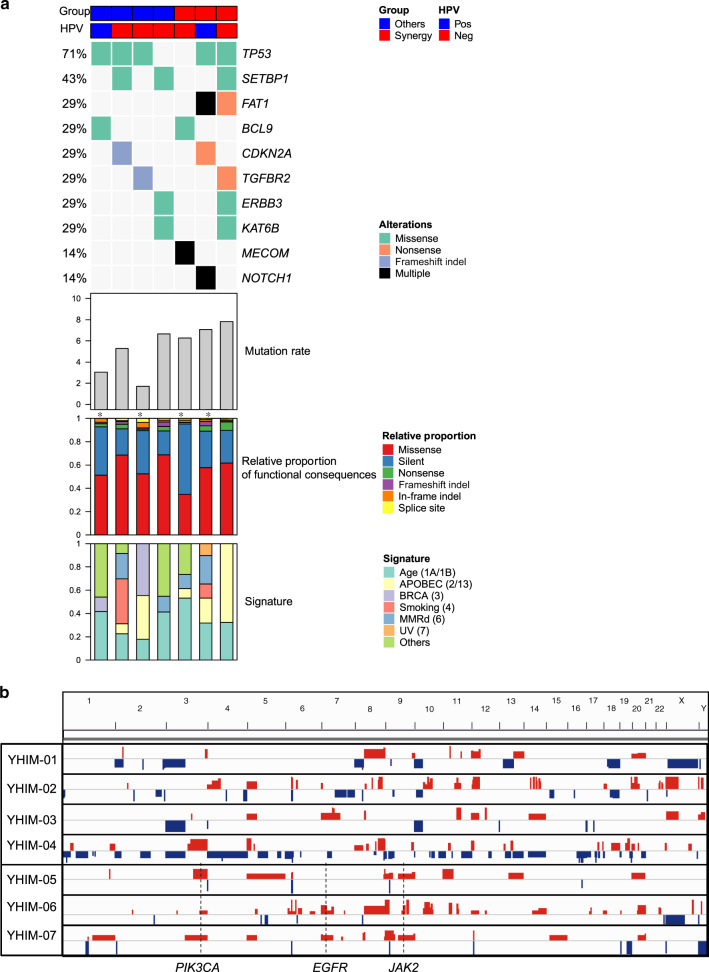
Genetic alteration profile in patient-derived xenograft models. **a** Somatic mutations in known cancer-related genes based on the Cancer Gene Census for all seven PDX models (first panel); mutation rates for all seven PDX models (second panel); relative proportions of functional consequences (third panel); and signatures (fourth panel). **b** Genome-wide somatic copy number alteration profiles of seven PDX models using whole-exome sequencing data. Asterisks indicate the cases with NS/S (nonsynonymous/synonymous) ratios less than 2.0.

Given the similarity of drug sensitivities between xenografts and patients, we set out to identify predictive biomarkers of buparlisib and cetuximab using the genetic and transcriptomic profiles of PDX tumours. Somatic mutations in known cancer-related genes based on the Cancer Gene Census^29^ are shown in Fig. 3a and Supplementary Table S5. Recurrent non-silent mutations (≥2) in 10 genes (*TP53*, *SETBP1*, *FAT1*, *BCL9*, *CDKN2A*, *MECOM*, *TGFBR2*, *ERBB2*, *KAT6B* and *NOTCH1*) are shown with mutation types (e.g. missense and nonsense). The annotated amino-acid residue changes of these mutations along with those of singletons are listed in Supplementary Table S5. The most common mutations were *TP53* mutations (YHIM-01, -02, -03, -06 and -07). Mutations in *FAT1*, *CDKN2A*, *NOTCH1* and *TGFBR2* have been reported to be frequent in SCCHN; all mutations of these loci in our cohort were found to be truncating (either as nonsense or frameshift) and often appeared as a double hit, suggestive of inactivation of both alleles (i.e. YHIM-06 and -07 showed nonsense and frameshift mutations for *FAT1* and *NOTCH1*, respectively). However, no *PIK3CA* mutations or recently reported novel SCCHN-related mutations, such as *AJUBA* and *NSD1*, were found.

We also examined singleton mutations in cancer-related genes. Of note, YHIM-05 harboured mutations in *HRAS* and *CASP8*, but retained wild-type *TP53*, which is expected to have favourable clinical outcomes, and a deficit of SCNAs^7^ was found as a synergy group. For the recurrent cancer-related genes shown in Supplementary Table S5, no mutations showed significant enrichment toward cases with synergy; this was also true for the remaining non-silent mutations in all the mutated genes. The somatic mutations identified in seven cases are presented in Supplementary Table 6.

The mutation rates (i.e. the number of exonic mutations per Mb) for all seven PDX models are shown in Fig. 3a. Cases corresponding to YHIM-05, -06 and -07 showed relatively elevated mutation burdens (6.3–7.8 mutations/Mb) compared with the others (YHIM-01, -02, -03 and -04) (1.7–6.7 mutations/Mb) (*P* = 0.075). The relative proportions of missense mutations as well as mutation signatures are illustrated in Fig. 3a. The nonsynonymous/synonymous ratio was relatively low (<2.0) for four cases (YHIM-01, -03, -05 and -06), suggesting that xenocontamination was still substantial and may have produced elevated mutation rates for those cases (asterisks in Fig. 3a). Mutation signature analysis revealed age-related signatures (signatures 1A and 1B; annotated as previously proposed)^31^ that were universally observed across the genomes examined (17–46%). Previous signature analyses on TCGA head and neck cancer genomes revealed that APOBEC, smoking and ultraviolet radiation-related signatures (signature 2/13, 4 and 7, respectively) comprise the major mutation signatures of SCCHN.^7^ In this study, these signatures were observed in 4, 2 and 1 case(s), respectively.

We also investigated somatic mutations in 31 patients from whom tumour tissue was available. Significant genetic alterations related to drug response were not observed (Supplementary Fig. S3). Because there were no matched normal samples for these 31 patients, we investigated all the mutations of these samples using HaplotypeCaller. Thereafter, to assess the cancer-specific mutations, we employed CIVic^26^ and DoCM,^27^ which are specialised databases for interpretation of cancer mutations. Consequently, there were no targetable mutations, such as *PIK3CA* E542K, E545K and H1047R, which are known as actionable mutations. Supplementary Figure S3A was drawn to confirm the pattern of the cancer-specific mutations. *TP53* (1 for L72Q, 1 for Y166C, 1 for Y181C, 1 for R209W, 2 for R234H), *MLH1* (3 for V384D), *CDKN2A* (1 for R80X, 1 for W110X), *STK11* (1 for P281L) and *MAPK1* (1 for E332K) mutations were included in the CIVic and DoCM databases. Supplementary Figure S3B is a needle plot showing the hotspot mutations of *TP53*.

### Copy number alteration profiles in PDX tumours

SCNAs were evaluated using WES data. Genome-wide SCNA profiles of seven cases examined are illustrated in Fig. 3b. Recurrent losses were observed in 3p and 10p, along with recurrent gains in 3q, 5p, 8q, 9p/q, 12q and 20p/q, consistent with the previously reported frequencies of SCNA in SCCHN genomes.^7^ We also examined focal, gene-level amplifications or deletions of genes previously reported to be related to SCCHN,^7^ including the locus chr9:1–17 Mb (Fig. 3b). Of note, the amplification of *PIK3CA* and *JAK2* was relatively frequent for YHIM-05, -06 and -07, and *EGFR* amplification was observed in YHIM-06 and -07 (Fig. 3b).

### Combination treatment upregulated genes associated with apoptotic and cell-cycle arrest

**Fig. 4:**
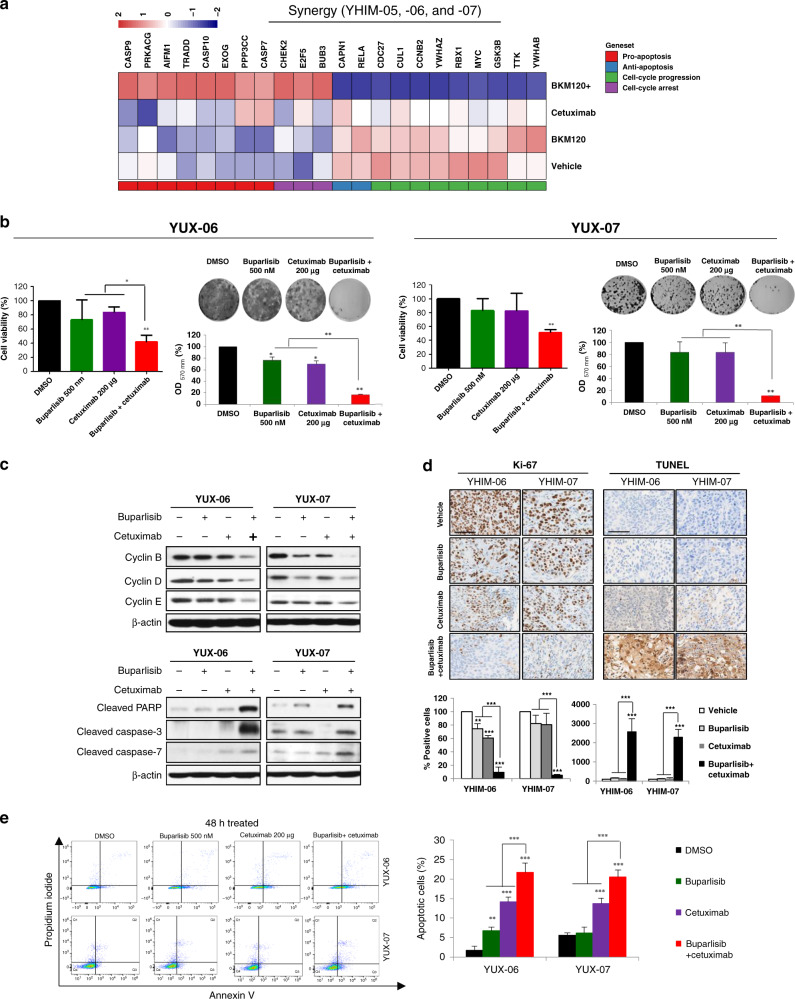
In vitro experiments for cell proliferation in the PDX models showing synergy of buparlisib/cetuximab. **a** Comparison of gene expression profiles between buparlisib, cetuximab, buparlisib/cetuximab and control in PDX models YHIM-05, -06 and -07, showing that genes associated with apoptosis and cell-cycle arrest were significantly upregulated by combination therapy. **b** Cell viability as a function of drug treatment of cell lines from PDX models YUX-06 and -07). **c** Expression of genes related to cell-cycle progression (cyclins B, D and E) and apoptosis (cleaved PARP, cleaved caspase-3 and cleaved-7) in YUX-06 and -07. **d** IHC showing proliferative (Ki67) and apoptotic (TUNEL) markers in YHIM-06 and -07. **e** Flow cytometry showing apoptosis in YUX-06 and -07.

To elucidate the mechanism underlying the synergistic effect of buparlisib and cetuximab, we performed a comparative analysis of gene expression in YHIM-05, -06 and -07 at baseline, after buparlisib or cetuximab alone and after combination therapy. Genes associated with apoptotic and cell-cycle arrest were significantly upregulated with combination therapy compared with that after each buparlisib or cetuximab monotherapy. In contrast, the expression of genes related to anti-apoptotic and cell-cycle progression was downregulated (Fig. 4a).

To explore the mechanism of combination therapy, we performed cell viability assays and immunoblots using PDX-derived tumour cell lines from YUX-06 and -07 (YHIM-06 and -07). The combination of buparlisib with cetuximab induced a synergistic anti-tumour effect, as evidenced by CI < 1 (CI value, 0.628 (YUX-06); 0.756 (YUX-07)) and induced apoptosis (Fig. 4b). Dual inhibition of the EGFR and PI3K pathways induced the downregulation of molecules related to cell-cycle progression (cyclin B, cyclin D and cyclin E) and upregulation of molecules related to apoptosis (cleaved PARP, cleaved caspase-3 and caspase-7) to greater extents than single inhibition (Fig. 4c). The combination treatment significantly increased apoptosis and decreased proliferation in YHIM-06 and -07 based on IHC (*P* < 0.001; Fig. 4d). Moreover, apoptosis was dramatically increased in YUX-06 and -07 by flow cytometry (*P* < 0.001; Fig. 4e). In YUX-01 and -02, the levels of Bcl-2, cleaved caspase-9 and Myc were not changed by buparlisib monotherapy; however, increased cleaved caspase-9 and decreased Bcl-2 and Myc were observed after cetuximab as well as after buparlisib/cetuximab combination therapy (Supplementary Figure S4).

## Discussion

This study showed that the pan-PI3K inhibitor buparlisib was insufficient for the treatment of patients with R/M SCCHN, even though PI3K pathway alterations are frequently caused by mutations associated with SCCHN.^7,8^ We conducted a mouse–human co-clinical trial, with PDX models derived from biopsied tumour samples of the patients enrolled in this Phase 2 clinical trial. This co-clinical trial indicated that all PDX models were resistant to buparlisib monotherapy, while some PDX models showed remarkable sensitivity to the combination of buparlisib/cetuximab. Based on these results, we revised the clinical trial protocol and switched patients who failed to respond to buparlisib monotherapy to the combination of buparlisib/cetuximab. The combination improved treatment outcomes without any significant increase in treatment-related toxicities. Transcriptomic analyses indicated that genes related to apoptosis and cell-cycle arrest were significantly upregulated upon combination treatment compared with treatment with buparlisib or cetuximab alone.

We successfully established PDX models from R/M SCCHN patients and conducted a co-clinical trial in parallel with a Phase 2 clinical trial. We demonstrated that histologic and genetic characteristics were highly preserved between patient tumours and corresponding PDX tumours. Although the PI3K–AKT–mTOR pathway is frequently activated in SCCHN,^7,8^ the efficacy data from the clinical trial as well as from PDX models showed the tumour to be highly resistant to buparlisib monotherapy. Our findings indicated that the combination treatment induced a synergistic anti-tumour effect as evidenced by CI < 1, and induced apoptosis and cell-cycle arrest. To exclude exactly that combination regimen is not the effect of cetuximab monotherapy, the clinical study design comparing combination (buparlisib/cetuximab), buparlisib monotherapy and cetuximab monotherapy is necessary. There were lots of clinical trials with cetuximab monotherapy in head and neck cancer patients. In general, the RR of cetuximab monotherapy has been known as 10–20%,^32^ even though the RR was different depending on the trials.

To identify predictive genomic alterations in response to combination therapy, we conducted comprehensive genomic and transcriptomic analyses of the baseline and on-treatment tumour samples. Unexpectedly, predictive genomic alterations upon combination therapy were not observed in either PDX or patient samples, which would be limited by a small sample size. Expression of genes associated with apoptosis and cell-cycle arrest was significantly upregulated by combination treatment. These preclinical data strongly suggest that combination therapies with buparlisib and cetuximab exert their effects by facilitating apoptosis and cell-cycle arrest. Thus, the therapeutic strategy of PI3K inhibitor plus EGFR monoclonal antibody could improve treatment outcomes in R/M SCCHN patients.

Treatment-related toxicities must be considered when developing combination therapies for cancer patients. Known AEs associated with buparlisib, including hyperglycaemia and gastrointestinal AEs (e.g. stomatitis, diarrhoea, nausea and vomiting), could be managed with the established strategies of dose reduction and treatment of symptoms with appropriate concomitant medication. Moreover, the occurrence of AEs related to cetuximab, including skin rash, mucositis or diarrhoea,^3^ was not increased in patients by the combination therapy. However, in the combination phase of this study, patients are congruent with the AE of these combined agents and high discontinuation rate. This study has a limitation that only 11 patients were treated with buparlisib/cetuximab after revising the protocol, which is a small number. Thus, cautious interpretation and additional clinical trials are needed to confirm our results.

To our knowledge, this is the first study demonstrating the additive or synergistic effects of buparlisib and cetuximab in a clinical trial based on preclinical PDX data, which can significantly contribute to the clinical development of PI3K inhibitors in R/M SCCHN patients. A combination of buparlisib/cetuximab may overcome resistance to buparlisib and represent a more effective option for treating patients with R/M SCCHN.

## Supplementary information

File_including Table_Fig_supplementary

## Data Availability

All data presented within the article and its Supplementary information files are available upon request from the corresponding author.
