# Supplementary material for: Mouse–human co-clinical trials demonstrate superior anti-tumour effects of buparlisib (BKM120) and cetuximab combination in squamous cell carcinoma of head and neck
Source: Br J Cancer. 2020 Sep 23;123(12):1720–9. doi: 10.1038/s41416-020-01074-2 (PMC7722843; doi:10.1038/s41416-020-01074-2)
Supplement: Supplementary file 1 — File_including Table_Fig_supplementary [file 41416_2020_1074_MOESM1_ESM.pdf]

Figure S1

YHIM-02

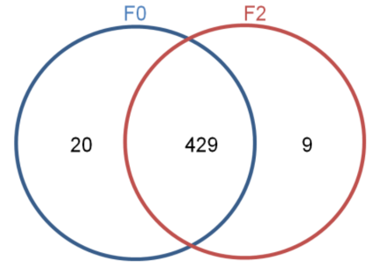

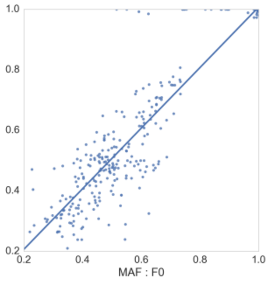

Jaccard similarity: 0.937

YHIM-01

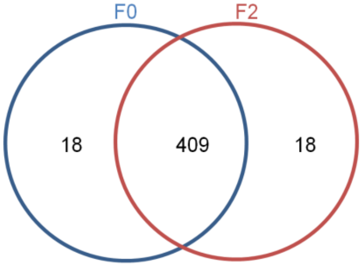

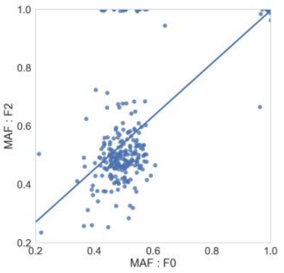

Jaccard similarity: 0.919

Sample ID	F0 specific	F0/F2 common	F2 specific	Jaccard similarity
YHIM-02	20	429	9	0.937
YHIM-07	14	459	26	0.920
YHIM-01	18	409	18	0.919
YHIM-04	55	371	21	0.830
YHIM-06	73	376	7	0.825

Figure S2

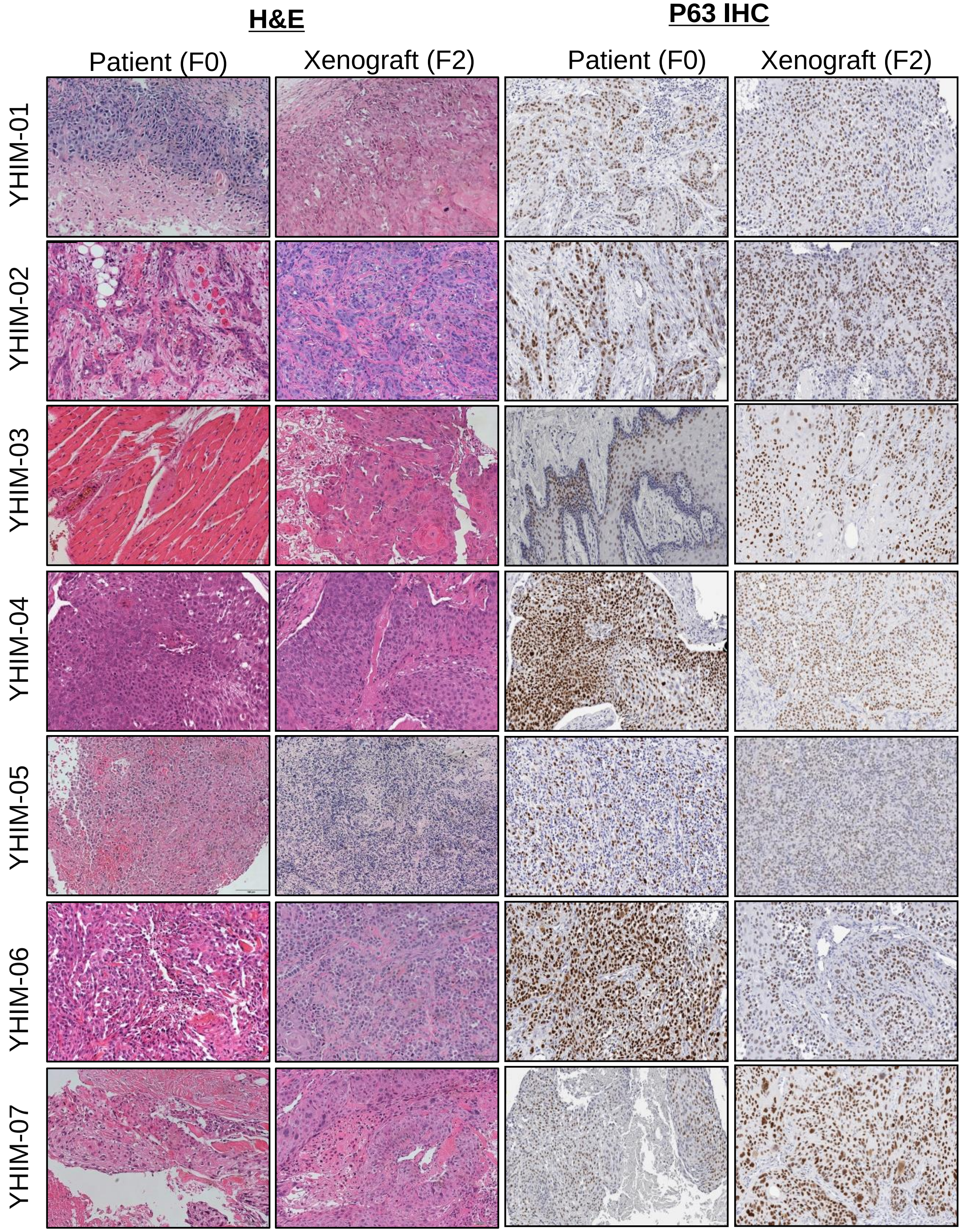

H&E
P63 IHC
Patient (F0)
Xenograft (F2)
Patient (F0)
Xenograft (F2)
YHIM-01
YHIM-02
YHIM-03
YHIM-04
YHIM-05
YHIM-06
YHIM-07

Figure S3

A

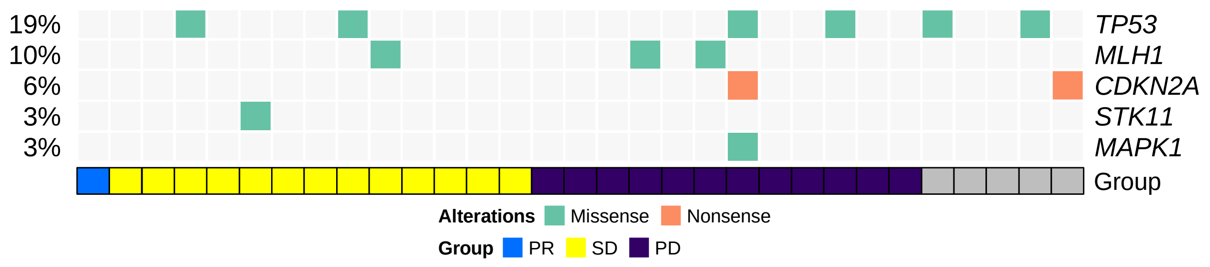

19%
10%
6%
3%
3%
TP53
MLH1
CDKN2A
STK11
MAPK1
Group
Alterations Missense Nonsense
Group PR SD PD

B

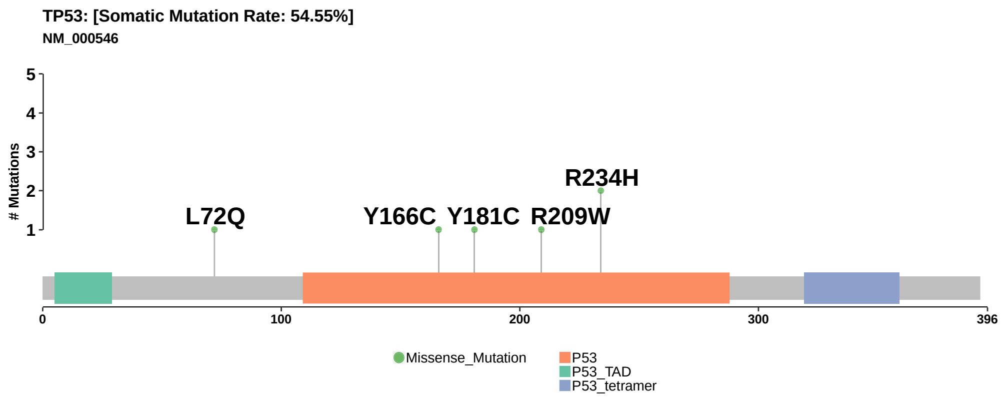

TP53: [Somatic Mutation Rate: 54.55%]
NM_000546
Mutations
5
4
3
2
1
R234H
L72Q
Y166C
Y181C
R209W
0
100
200
300
396
Missense_Mutation
P53
P53_TAD
P53_tetramer

Figure S4

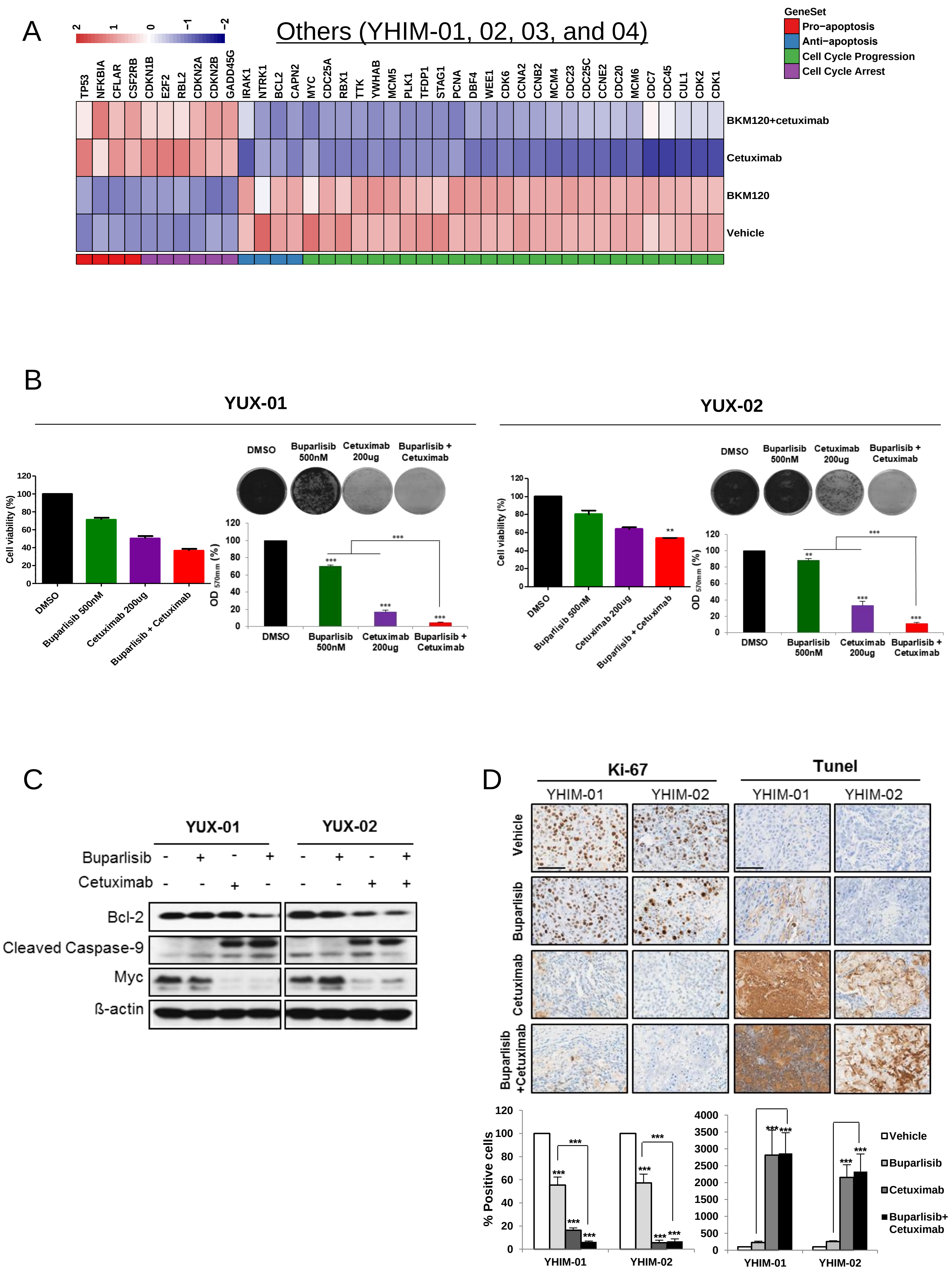
A
2
1
0
−1
−2
Others (YHIM-01, 02, 03, and 04)
GeneSet
Pro−apoptosis
Anti−apoptosis
Cell Cycle Progression
Cell Cycle Arrest
TP53
NFKBIA
CFLAR
CSF2RB
CDKN1B
E2F2
RBL2
CDKN2A
CDKN2B
GADD45G
IRAK1
NTRK1
BCL2
CAPN2
MYC
CDC25A
RBX1
TTK
YWHAB
MCM5
PLK1
TFDP1
STAG1
PCNA
DBF4
WEE1
CDK6
CCNA2
CCNB2
MCM4
CDC23
CDC25C
CCNE2
CDC20
MCM6
CDC7
CDC45
CUL1
CDK2
CDK1
BKM120+cetuximab
Cetuximab
BKM120
Vehicle
B
YUX-01
YUX-02
Cell viability (%)
DMSO
Buparlisib 500nM
Cetuximab 200ug
Buparlisib + Cetuximab
Buparlisib 500nM
Cetuximab 200ug
Buparlisib + Cetuximab
OD 570mm (%)
C
YUX-01
YUX-02
Buparlisib
Cetuximab
Bcl-2
Cleaved Caspase-9
Myc
ß-actin
D
Ki-67
Tunel
YHIM-01
YHIM-02
Vehicle
Buparlisib
Cetuximab
Buparlisib +Cetuximab
% Positive cells
Vehicle
Buparlisib
Cetuximab
Buparlisib+ Cetuximab

Table S1. 244 cancer-related genes list using a customized SureSelect kit for targeted capture sequencing

Gene	ID
ABL1	25
ABL2	27
AKT1	207
AKT2	208
AKT3	10000
APC	324
AR	367
ARAF	369
ARID1A	8289
ARID2	196528
ASH1L	55870
ASXL1	171023
ASXL3	80816
ATM	472
ATR	545
ATRX	546
AURKA	6790
AURKB	9212
AXL	558
BAP1	8314
BARD1	580
BIRC3	330
BIRC6	57448
BLM	641
BRAF	673
BRCA1	672
BRCA2	675
BRD3	8019
BRD4	23476
BRD7	29117
BUB1	699
CASP8	841
CBL	867
CCND1	595
CCND2	894
CCND3	896
CCNE1	898
CDH1	999
CDH10	1008
CDH9	1007
CDK12	51755
CDK4	1019
CDK6	1021
CDK8	1024
CDKN1B	1027
CDKN2A	1029
CDKN2B	1030
CDKN2C	1031
CEBPA	1050
CEP350	9857
CFTR	1080
CHD2	1106
CHD4	1108
CHD5	26038
CHD7	55636
CHEK1	1111
CHEK2	11200
CREBBP	1387
CRKL	1399
CTNNA1	1495
CTNNA2	1496
CTNNB1	1499

CTNND2	1501
CTSK	1513
CTTN	2017
CUL3	8452
DACH1	1602
DAXX	1616
DDR2	4921
DNMT1	1786
DNMT3A	1788
DNMT3B	1789
DOT1L	84444
DUSP27	92235
EGFR	1956
EP300	2033
EPCAM	4072
EPHA1	2041
EPHA2	1969
EPHA3	2042
EPHA4	2043
EPHA5	2044
EPHA7	2045
EPHB1	2047
EPHB4	2050
ERBB	1956
ERBB2	2064
ERBB3	2065
ERBB4	2066
ERG	2078
EZH2	2146
FAM123B(AMER1)	1392285
FAT1	2195
FAT2	2196
FAT3	120114
FAT4	79633
FBN2	2201
FBXW7	55294
FES	2242
FGFR1	2260
FGFR2	2263
FGFR3	2261
FGFR4	2264
FOXP1	27086
GATA1	14460
GATA2	2624
GATA3	2625
GNA11	2767
GNA13	10672
GNAQ	2776
GNAS	2778
GPR98	84059
HDAC1	3065
HDAC2	3066
HDAC3	8841
HDAC5	10014
HDAC6	10013
HDAC7A	51564
HDAC9	9734
HRAS	3265
IDH1	3417
IDH2	3418
IGF1R	3480
IKBKE	9641
IL7R	3575
ITGB1	3688

ITGB3	3690
JAK1	3716
JAK2	3717
JAK3	3718
KDM5A	5927
KDM5C	8242
KDM6A	7403
KDR	3791
KEAP1	9817
KIT	3815
KLHL1	57626
KLHL6	89857
KMT2D	8085
KRAS	3845
LAMA2	3908
LAMA3	3909
LAMB3	3914
LAMC2	3918
MAP2K1	5604
MAP2K2	5605
MAP2K4	6416
MAP3K1	4214
MAP3K8	1326
MAP3K9	4293
MAPK1	5594
MAPK9	5601
MBD1	4152
MBD2	8932
MDM4	4194
MET	4233
MLH1	4292
MLL1(KMT2A)	4297
MLL2(MLL4, KMT2D)	8085
MLL3(KMT2C)	58508
MTOR	2475
MYC	4609
MYCL1(MYCL)	4610
MYCN	4613
NF1	4763
NF2	4771
NFE2L2	4780
NFKBIA	4792
NKX2-1	7080
NOTCH1	4851
NOTCH2	4853
NPM1	4869
NRAS	4893
NSD1	64324
PAK3	5063
PAK5(PAK7)	57144
PBRM1	55193
PDGFRA	5156
PDGFRB	5159
PDK1	5163
PIK3C2A	5286
PIK3C2B	5287
PIK3C2G	5288
PIK3C3	5289
PIK3CA	5290
PIK3CB	5291
PIK3CD	5293
PIK3CG	5294
PIK3R1	5295
PIK3R2	5296

PIK3R3	8503
PIK3R6	146850
PPFIA2	8499
PPP2R1A	5518
PRB1	5542
PRB2	653247
PRKCI	5584
PTCH1	5727
PTEN	5728
PTK2B	2185
PTPN11	5781
PTPRD	5789
RAC1	5879
RAD50	10111
RAD51	5888
RAF1	5894
RASSF1A	11186
RB1	5925
RELN	5649
RHOA	387
RICTOR	253260
RIT1	6016
RPS6KA1	6195
RPTOR	57521
SETD2	29072
SIRT1	23411
SIRT2	22933
SMAD2	4087
SMAD3	4088
SMAD4	4089
SMARCA2	6595
SMARCA4(BRG1)	6597
SMARCB1	6598
SMO	6608
SOCS1	8651
SOX10	6663
SOX2	6657
SRC	6714
STAT3	6774
STAT4	6775
STK11	6794
STYK1	55359
TERT	7015
TET2	54790
TGFBR2	7048
TOP1	7150
TP53	7157
TP63	8626
TRIO	7204
TSC1	7248
TSC2	7249
VHL	7428
WHSC1L1	54904
XPO1	7514

Table S2. Treatment related toxicity profile in clinical trial

	Buparlisib mono (N = 35)		Buparlisib → buparlisib + cetuximab (N = 11)	
**Toxicity**	All grades n (%)	Grade ≥ 3 n (%)	All grades n (%)	Grade ≥ 3 n (%)
**Hematological**				
Anemia	3 (8.6%)	3 (8.6%)	1 (9.1%)	1 (9.1%)
Neutropenia	2 (5.8%)	0 (0%)	0 (0%)	0 (0%)
Thrombocytopenia	2 (5.8%)	0 (0%)	0 (0%)	0 (0%)
**Non-hematological**				
Pruritus	6 (17.2%)	0 (0%)	0 (0%)	0 (0%)
Skin rash	3 (8.6%)	0 (0%)	5 (54.6%)	0 (0%)
Mucositis	1 (2.9%)	0 (0%)	7 (63.7%)	0 (0%)
General weakness	5 (14.3%)	1 (2.9%)	4 (36.4%)	0 (0%)
Fatigue	11 (31.4%)	3 (8.6%)	7 (63.7%)	1 (9.1%)
Nausea	7 (20.0%)	1 (2.9%)	2 (18.2%)	1 (9.1%)
Constipation	3 (8.6%)	0 (0%)	2 (18.2%)	0 (0%)
Diarrhea	3 (8.6%)	2 (5.8%)	1 (9.1%)	0 (0%)
Anorexia	14 (40.0%)	5 (14.3%)	1 (9.1%)	1 (9.1%)
Dyspepsia	4 (11.5%)	0 (0%)	0 (0%)	0 (0%)
Weight loss	15 (42.9%)	0 (0%)	4 (36.4%)	0 (0%)
Dyspnea	4 (11.5%)	1 (2.9%)	4 (36.4%)	0 (0%)
Hyper-glycemia	10 (28.6%)	10 (28.6%)	1 (9.1%)	3 (27.3%)
Increased ALT/ALT	6 (17.2%)	1 (2.9%)	0 (0%)	0 (0%)
Increased Creatinine	6 (17.2%)	0 (0%)	0 (0%)	0 (0%)
Hypertension	2 (5.8%)	2 (5.8%)	1 (9.1%)	0 (0%)
Fever	5 (14.3%)	0 (0%)	3 (27.3%)	0 (0%)
Pneumonia	3 (8.6%)	3 (8.6%)	1 (9.1%)	0 (0%)
**Psychological**				
Insomnia	5 (14.3%)	0 (0%)	1 (9.1%)	0 (0%)
Anxiety	5 (14.3%)	0 (0%)	1 (9.1%)	0 (0%)
Delirium	1 (2.9%)	1 (2.9%)	3 (27.3%)	0 (0%)
Depression	9 (25.7%)	1 (2.9%)	3 (27.3%)	0 (0%)
Confusion	1 (2.9%)	0 (0%)	1 (9.1%)	0 (0%)
Cognitive disorder	1 (2.9%)	1 (2.9%)	1 (9.1%)	0 (0%)

Table S3. Detailed information of patient derived xenograft model in 'co-clinical trial'

PDX ID		Cancer type	HPV (p16)	Stage at tumor acquisition	Tumor acquisition method	Tumor acquisition site	Prior therapy
YHIM-01	Co-clinical	Tongue	Positive	Stage IV	Biopsy	Paraspinal muscle	Surgery, RT, 5FU/DDP
YHIM-02	Co-clinical	Tongue	Negative	Stage III	Surgery	Tongue	Surgery
YHIM-03		Tongue	Negative	Stage III	Surgery	Tongue	Surgery,
YHIM-04		Oropharynx	Positive	Stage IVA	Biopsy	Skin nodule	Surgery, CCRT, 5FU/DDP
YHIM-05		Tongue	Negative	Stage IVA	Surgery	Tongue	Surgery
YHIM-06	Co-clinical	Hypopharynx	Negative	Stage IVB	Biopsy	Tongue	Induction Docetaxel/DDP, Surgery, CCRT
YHIM-07	Co-clinical	Ethmoid sinus	Negative	Stage IVB	Biopsy	Neck node	CCRT, 5FU/DDP

Table S4. Tumor growth inhibition by each dug or combination therapy

Model ID	% TGI by bupalrisib	% TGI by cetuximab	% TGI by buparlisib+cetuximab	P-value (buplarisib vs. combo)	P-value (cetuximab vs. combo)
YHIM-01	58.83	110.04	106.95	<0.05	NS
YHIM-02	50.77	103.42	114.32	<0.0001	NS
YHIM-03	43.86	104.31	105.76	<0.0001	NS
YHIM-04	84.78	62.18	98.46	<0.05	NS
YHIM-05	58.59	20.65	114.92	<0.0001	<0.0001
YHIM-06	42.37	78.63	98.01	<0.001	<0.001
YHIM-07	54.24	76.72	102.09	<0.001	<0.05

Table S5. Mutations in cancer-related genes

Gene	YHIM-01	YHIM-02	YHIM-03	YHIM-04	YHIM-05	YHIM-06	YHIM-07
TP53	p.A6V, p.I63T	p.R116W	p.V25F			p.R141H	p.Y73C
SETBP1		p.P506T		p.E1591D			p.K939N
FAT1						FS, NS	NS
BCL9	p.Q52P				p.Q52P		
CDKN2A		FS				NS	
MECOM					p.I417L, p.F382L		
TGFBR2			FS				NS
ERBB3				p.R1173W			p.S1285F
KAT6B				p.L697P			p.E822K
NOTCH1						FS, FS	
Singletons		ATR (NS), CCND1 (p.L99P), GNAS (p.A229T), GPHN (p.I71K)	KMT2C (p.I745T), MYH11 (NS)	ATP2B3 (p.K335N), BAP1 (p.I47M), CARD11 (p.E282A), ERC1 (p.F435C), GPC3 (p.C368S), IKBKB (p.R29G), MED12 (p.E509Q), MLLT4 (p.R1211T), NCOR2 (p.S1209L), PER1 (p.R669S), PPFIBP1 (p.E558Q), PTCH1 (NS), TSC1 (p.G38C)	ARHGAP26 (p.S674L), CASP8 (NS), EXT1 (p.L26V), HRAS (p.G12D), TET1 (p.R1780Q)	CTNNB1 (p.Q143E), ESR1 (p.R217S), FAT4 (NS), KDM6A (p.S204I), KMT2D (p.R1601H), MUC1 (p.P134A), NCOR1 (p.E133K), NRG1 (p.S26P), PDE4DIP (p.E1121D), PPARG (p.V248F), TBX3 (p.S663L), TRRAP (p.R661T)	ARID2 (NS), CHN1 (p.T83S), NAB2 (p.K169R), NF2 (NS), RNF213 (p.R4659K), RSPO3 (p.G40C), TRIM33 (p.P629H), VHL (p.H125Q)

Table S6. List of somatic mutations identified in established seven PDX models

Case	Synergism	Gene	Chr	Start	End	Ref	Alt	Function	Exonic.Function	mRNA	exon	CDS.mutation	AA.mutation	CCG	MAF	REF_count	ALT_count	Mutation
YHIM-01	Non-synergy	ABCA4	chr1	94495020	94495020	C	T	exonic	nonsynonymous SNV	NM_000350	30	c.G4520A	p.G1507E		0.35714286	36	20	SNV
YHIM-01	Non-synergy	ABI2	chr2	204291916	204291916	C	T	exonic	synonymous SNV	NM_001282927	9	c.C1047T	p.D349D		0.04545455	147	7	SNV
YHIM-01	Non-synergy	ARC	chr8	143694555	143694555	C	T	exonic	nonsynonymous SNV	NM_015193	1	c.G1078A	p.E360K		0.2259887	137	40	SNV
YHIM-01	Non-synergy	ARHGAP44	chr17	12823072	12823072	G	T	exonic	nonsynonymous SNV	NM_014859	6	c.G388T	p.V130L		0.2	132	33	SNV
YHIM-01	Non-synergy	ASH1L	chr1	155311845	155311845	C	T	exonic	nonsynonymous SNV	NM_018489	25	c.G8342A	p.R2781Q		0.45806452	84	71	SNV
YHIM-01	Non-synergy	ATRN	chr20	3528046	3528046	C	T	exonic	nonsynonymous SNV	NM_001207047	5	c.C505T	p.P169S		0.40091116	263	176	SNV
YHIM-01	Non-synergy	BCL9	chr1	147084783	147084783	A	C	exonic	nonsynonymous SNV	NM_004326	5	c.A155C	p.Q52P	CCG	0.05555556	85	5	SNV
YHIM-01	Non-synergy	BOD1L2	chr18	54814770	54814770	C	T	exonic	nonsynonymous SNV	NM_001257964	1	c.C227T	p.A76V		0.99264706	1	135	SNV
YHIM-01	Non-synergy	C2orf81	chr2	74642029	74642029	G	C	exonic	synonymous SNV	NM_001316766	2	c.C990G	p.P330P		0.42957747	81	61	SNV
YHIM-01	Non-synergy	CCDC85C	chr14	100070090	100070090	G	A	exonic	synonymous SNV	NM_001144995	1	c.C207T	p.G69G		0.08045977	160	14	SNV
YHIM-01	Non-synergy	CDH8	chr16	61687851	61687851	A	G	exonic	synonymous SNV	NM_001796	12	c.T2061C	p.N687N		0.04659498	266	13	SNV
YHIM-01	Non-synergy	CDK11A	chr1	1636355	1636355	A	G	exonic	synonymous SNV	NM_001313896	13	c.T1455C	p.I485I		0.04032258	119	5	SNV
YHIM-01	Non-synergy	CDKN2A	chr9	21974727	21974744	CCTC	-	exonic	nonframeshift deletion	NM_000077	1	c.83_100del	p.28_34del	CCG				Indel
YHIM-01	Non-synergy	CELF4	chr18	34839207	34839207	A	G	exonic	nonsynonymous SNV	NM_001025087	11	c.T1267C	p.F423L		0.12765957	41	6	SNV
YHIM-01	Non-synergy	CFAP221	chr2	120317283	120317283	A	T	exonic	nonsynonymous SNV	NM_001271049	4	c.A259T	p.N87Y		0.23880597	255	80	SNV
YHIM-01	Non-synergy	COL23A1	chr5	177669120	177669120	C	T	exonic	nonsynonymous SNV	NM_173465	27	c.G1504A	p.G502R		0.57575758	42	57	SNV
YHIM-01	Non-synergy	CSNK1E,LO(	chr22	38694911	38694911	G	A	exonic	synonymous SNV	NM_001894	7	c.C765T	p.C255C		0.52444444	107	118	SNV
YHIM-01	Non-synergy	DENND1A	chr9	126554884	126554884	C	T	exonic	synonymous SNV	NM_020946	3	c.G114A	p.P38P		0.04347826	154	7	SNV
YHIM-01	Non-synergy	DGKZ	chr11	46396520	46396520	G	A	exonic	synonymous SNV	NM_001199268	18	c.G1566A	p.G522G		0.30967742	107	48	SNV
YHIM-01	Non-synergy	DST	chr6	56394512	56394512	G	A	exonic	stopgain	NM_015548	47	c.C9460T	p.Q3154X		0.3037037	94	41	SNV
YHIM-01	Non-synergy	EDC3	chr15	74948121	74948121	T	C	exonic	nonsynonymous SNV	NM_025083	4	c.A773G	p.Y258C		0.50909091	27	28	SNV
YHIM-01	Non-synergy	EEF1B2	chr2	207024766	207024766	C	T	exonic	synonymous SNV	NM_001959	1	c.C63T	p.D21D		0.42352941	49	36	SNV
YHIM-01	Non-synergy	EFCAB7	chr1	63997564	63997564	G	A	exonic	synonymous SNV	NM_032437	3	c.G261A	p.L87L		0.06363636	103	7	SNV
YHIM-01	Non-synergy	FAM120A	chr9	96294642	96294642	T	C	exonic	nonsynonymous SNV	NM_001286723	10	c.T1940C	p.F647S		0.05102041	93	5	SNV
YHIM-01	Non-synergy	FAM13B	chr5	137289149	137289149	C	T	exonic	nonsynonymous SNV	NM_001101800	15	c.G1658A	p.R553Q		0.47244095	67	60	SNV
YHIM-01	Non-synergy	FAM98A	chr2	33813408	33813408	T	C	exonic	synonymous SNV	NM_015475	4	c.A516G	p.E172E		0.16363636	46	9	SNV
YHIM-01	Non-synergy	FAR1	chr11	13736138	13736138	T	C	exonic	synonymous SNV	NM_032228	9	c.T1038C	p.H346H		0.3	35	15	SNV
YHIM-01	Non-synergy	FBRS	chr16	30675684	30675684	T	C	exonic	synonymous SNV	NM_001105079	7	c.T1204C	p.L402L		0.07142857	39	3	SNV
YHIM-01	Non-synergy	FLG	chr1	152281403	152281403	G	A	exonic	nonsynonymous SNV	NM_002016	3	c.C5959T	p.R1987C		0.32941177	114	56	SNV
YHIM-01	Non-synergy	FOSL2	chr2	28616674	28616674	C	T	exonic	synonymous SNV	NM_005253	1	c.C87T	p.G29G		0.11428571	31	4	SNV
YHIM-01	Non-synergy	GMDS	chr6	1742713	1742713	T	C	exonic	synonymous SNV	NM_001253846	8	c.A789G	p.G263G		0.32642487	130	63	SNV
YHIM-01	Non-synergy	GOLGA8H	chr15	30900671	30900671	T	C	exonic	nonsynonymous SNV	NM_001282490	7	c.T398C	p.V133A		0.75	2	6	SNV
YHIM-01	Non-synergy	GPLD1	chr6	24489653	24489653	G	A	exonic	synonymous SNV	NM_001503	1	c.C87T	p.H29H		0.125	63	9	SNV
YHIM-01	Non-synergy	HAT1	chr2	172822963	172822963	C	T	exonic	synonymous SNV	NM_003642	7	c.C645T	p.L215L		0.27586207	273	104	SNV
YHIM-01	Non-synergy	HCLS1	chr3	121351332	121351332	-	A	exonic	stopgain	NM_001292041	11	c.975_976insT	p.E326_P327delinsX					Indel
YHIM-01	Non-synergy	HCN3	chr1	155257605	155257605	C	T	exonic	nonsynonymous SNV	NM_020897	8	c.C1676T	p.S559F		0.54477612	61	73	SNV
YHIM-01	Non-synergy	HIST1H1A	chr6	26017608	26017608	T	C	exonic	nonsynonymous SNV	NM_005325	1	c.A353G	p.E118G		0.69318182	54	122	SNV
YHIM-01	Non-synergy	HNRNPH1	chr5	179044095	179044095	T	C	exonic	synonymous SNV	NM_001257293	10	c.A1074G	p.E358E		0.0472973	141	7	SNV
YHIM-01	Non-synergy	HSPA12A	chr10	118458162	118458162	T	A	exonic	nonsynonymous SNV	NM_025015	5	c.A530T	p.K177M		0.25	42	14	SNV
YHIM-01	Non-synergy	HSPD1	chr2	198363501	198363501	C	T	exonic	synonymous SNV	NM_002156	2	c.G72A	p.R24R		0.05128205	74	4	SNV
YHIM-01	Non-synergy	HTR5A	chr7	154863006	154863006	A	G	exonic	nonsynonymous SNV	NM_024012	1	c.A397G	p.T133A		0.05813954	243	15	SNV
YHIM-01	Non-synergy	HYDIN	chr16	70852324	70852324	T	C	exonic	nonsynonymous SNV	NM_001270974	84	c.A14579G	p.Y4860C		0.46218487	64	55	SNV
YHIM-01	Non-synergy	ILF2	chr1	153634976	153634976	C	T	exonic	nonsynonymous SNV	NM_001267809	14	c.G955A	p.A319T		0.48	130	120	SNV
YHIM-01	Non-synergy	INO80D	chr2	206927659	206927659	T	G	exonic	synonymous SNV	NM_017759	3	c.A82C	p.R28R		0.05769231	147	9	SNV
YHIM-01	Non-synergy	IRX3	chr16	54318974	54318974	C	T	exonic	synonymous SNV	NM_024336	2	c.G819A	p.A273A		0.471875	169	151	SNV
YHIM-01	Non-synergy	KAZN	chr1	14925619	14925619	-	GGA	exonic	nonframeshift insertion	NM_015209	1	c.126_127insGG	p.G42delinsGG					Indel
YHIM-01	Non-synergy	KCNJ5	chr11	128781201	128781201	G	A	exonic	synonymous SNV	NM_000890	2	c.G33A	p.Q11Q	CCG	0.48803828	214	204	SNV
YHIM-01	Non-synergy	KIAA1715	chr2	176794780	176794780	G	A	exonic	nonsynonymous SNV	NM_001305011	11	c.C833T	p.S278L		0.47876448	135	124	SNV
YHIM-01	Non-synergy	KRTAP4-5	chr17	39305775	39305775	-	GGCA(	exonic	nonframeshift insertion	NM_033188	1	c.244_245insGC	p.Q82delinsRPSCCQ					Indel
YHIM-01	Non-synergy	KRTAP9-8	chr17	39394344	39394344	G	T	exonic	nonsynonymous SNV	NM_031963	1	c.G41T	p.C14F		0.5	7	7	SNV
YHIM-01	Non-synergy	LARS	chr5	145510607	145510607	A	C	exonic	nonsynonymous SNV	NM_020117	24	c.T2462G	p.L821W		0.36206897	37	21	SNV
YHIM-01	Non-synergy	LMTK3	chr19	49002212	49002212	T	C	exonic	nonsynonymous SNV	NM_001080434	12	c.A2201G	p.E734G		0.90909091	1	10	SNV
YHIM-01	Non-synergy	LONRF2	chr2	100910764	100910764	G	A	exonic	nonsynonymous SNV	NM_198461	9	c.C1684T	p.R562W		0.52439024	39	43	SNV
YHIM-01	Non-synergy	LRP3	chr19	33696238	33696238	G	A	exonic	nonsynonymous SNV	NM_002333	5	c.G562A	p.V188M		0.31730769	142	66	SNV
YHIM-01	Non-synergy	LRRC63	chr13	46824581	46824581	G	C	exonic	nonsynonymous SNV	NM_001282460	7	c.G1181C	p.R394T		0.51351351	18	19	SNV
YHIM-01	Non-synergy	MAML2	chr11	95825372	95825374	TGT	-	exonic	nonframeshift deletion	NM_032427	2	c.1821_1823del	p.607_608del	CCG				Indel
YHIM-01	Non-synergy	MAML2	chr11	95825407	95825407	C	T	exonic	synonymous SNV	NM_032427	2	c.G1788A	p.Q596Q	CCG	0.1	54	6	SNV
YHIM-01	Non-synergy	11-Mar	chr5	16067708	16067708	G	A	exonic	stopgain	NM_001102562	4	c.C1081T	p.Q361X		0.4125	94	66	SNV
YHIM-01	Non-synergy	MATR3	chr5	138643914	138643914	A	G	exonic	synonymous SNV	NM_001194955	2	c.A810G	p.R270R		0.45059289	139	114	SNV

YHIM-01	Non-synergy	MATR3	chr5	138643173	138643173	G	A	exonic	synonymous SNV	NM_001194955	2	c.G69A	p.A23A		0.17105263	189	39	SNV
YHIM-01	Non-synergy	MTMR3	chr22	30418060	30418060	T	G	exonic	nonsynonymous SNV	NM_021090	18	c.T3268G	p.L1090V		0.06060606	186	12	SNV
YHIM-01	Non-synergy	MUC4	chr3	195515290	195515290	C	G	exonic	nonsynonymous SNV	NM_018406	2	c.G3161C	p.S1054T		0.12765957	41	6	SNV
YHIM-01	Non-synergy	MUC4	chr3	195512531	195512531	C	T	exonic	nonsynonymous SNV	NM_018406	2	c.G5920A	p.A1974T		0.05194805	73	4	SNV
YHIM-01	Non-synergy	MUC4	chr3	195511911	195511911	G	A	exonic	synonymous SNV	NM_018406	2	c.C6540T	p.T2180T		0.07692308	72	6	SNV
YHIM-01	Non-synergy	MYCBP2	chr13	77752044	77752044	G	C	exonic	nonsynonymous SNV	NM_015057	35	c.C5179G	p.R1727G		0.25274725	204	69	SNV
YHIM-01	Non-synergy	MYH14	chr19	50755898	50755898	C	T	exonic	synonymous SNV	NM_024729	15	c.C1809T	p.V603V		0.33333333	140	70	SNV
YHIM-01	Non-synergy	MYH2	chr17	10442826	10442826	C	A	exonic	nonsynonymous SNV	NM_001100112	13	c.G1201T	p.A401S		0.53977273	81	95	SNV
YHIM-01	Non-synergy	MYH7	chr14	23886492	23886492	C	A	exonic	synonymous SNV	NM_000257	32	c.G4389T	p.S1463S		0.19512195	66	16	SNV
YHIM-01	Non-synergy	NASP	chr1	46073323	46073323	G	A	exonic	nonsynonymous SNV	NM_001195193	4	c.G548A	p.G183E		0.0625	45	3	SNV
YHIM-01	Non-synergy	NELL2	chr12	45169812	45169812	G	A	exonic	nonsynonymous SNV	NM_001145108	8	c.C884T	p.T295I		0.26923077	57	21	SNV
YHIM-01	Non-synergy	NFIB	chr9	14113010	14113010	T	C	exonic	synonymous SNV	NM_001282787	9	c.A699G	p.L233L	CCG	0.07291667	89	7	SNV
YHIM-01	Non-synergy	NIPBL	chr5	36984991	36984991	T	C	exonic	nonsynonymous SNV	NM_015384	10	c.T1709C	p.I570T		0.06930693	94	7	SNV
YHIM-01	Non-synergy	NKTR	chr3	42684016	42684016	G	A	exonic	nonsynonymous SNV	NM_005385	14	c.G4070A	p.R1357Q		1	0	40	SNV
YHIM-01	Non-synergy	NKX2-1	chr14	36988572	36988572	G	T	exonic	synonymous SNV	NM_001079668	2	c.C81A	p.R27R	CCG	0.06349206	59	4	SNV
YHIM-01	Non-synergy	NKX2-1	chr14	36988266	36988266	C	A	exonic	synonymous SNV	NM_003317	1	c.G297T	p.P99P	CCG	0.05434783	87	5	SNV
YHIM-01	Non-synergy	NPTN	chr15	73889631	73889631	G	A	exonic	synonymous SNV	NM_001161363	2	c.C171T	p.S57S		0.0770751	467	39	SNV
YHIM-01	Non-synergy	NR1H4	chr12	100934501	100934501	C	T	exonic	nonsynonymous SNV	NM_001206992	7	c.C1001T	p.A334V		0.49122807	174	168	SNV
YHIM-01	Non-synergy	NR2E1	chr6	108501544	108501544	T	C	exonic	synonymous SNV	NM_001286102	6	c.T771C	p.D257D		0.12195122	72	10	SNV
YHIM-01	Non-synergy	NR2E1	chr6	108501547	108501547	T	G	exonic	synonymous SNV	NM_001286102	6	c.T774G	p.A258A		0.13793103	75	12	SNV
YHIM-01	Non-synergy	NR2F1	chr5	92920960	92920960	G	A	exonic	synonymous SNV	NM_005654	1	c.G231A	p.S77S		0.07027027	172	13	SNV
YHIM-01	Non-synergy	NR2F2	chr15	96875715	96875715	T	C	exonic	synonymous SNV	NM_021005	1	c.T381C	p.H127H		0.03525641	301	11	SNV
YHIM-01	Non-synergy	NR4A3	chr9	102626128	102626128	C	T	exonic	synonymous SNV	NM_006981	8	c.C1860T	p.F620F	CCG	0.26984127	46	17	SNV
YHIM-01	Non-synergy	NUP210L	chr1	153973368	153973368	C	G	exonic	nonsynonymous SNV	NM_207308	37	c.G5350C	p.V1784L		0.24096386	63	20	SNV
YHIM-01	Non-synergy	OSBPL10	chr3	31725448	31725448	C	T	exonic	synonymous SNV	NM_001174060	7	c.G1212A	p.E404E		0.51724138	42	45	SNV
YHIM-01	Non-synergy	OTOGL	chr12	80749655	80749655	A	T	exonic	synonymous SNV	NM_173591	46	c.A5706T	p.P1902P		0.21940928	185	52	SNV
YHIM-01	Non-synergy	PABPC1	chr8	101721385	101721385	T	C	exonic	nonsynonymous SNV	NM_002568	9	c.A1312G	p.T438A		0.16666667	55	11	SNV
YHIM-01	Non-synergy	PABPC1	chr8	101733644	101733644	A	G	exonic	synonymous SNV	NM_002568	1	c.T168C	p.Y56Y		0.0516129	147	8	SNV
YHIM-01	Non-synergy	PAPPA2	chr1	176664948	176664948	G	A	exonic	nonsynonymous SNV	NM_020318	7	c.G2699A	p.R900Q		0.54022989	40	47	SNV
YHIM-01	Non-synergy	PAX6	chr11	31822288	31822288	G	T	exonic	synonymous SNV	NM_001310160	4	c.C66A	p.T22T		0.12173913	101	14	SNV
YHIM-01	Non-synergy	PBX1	chr1	164815841	164815841	T	C	exonic	synonymous SNV	NM_002585	9	c.T1221C	p.D407D	CCG	0.05185185	128	7	SNV
YHIM-01	Non-synergy	PBX1	chr1	164815853	164815853	T	C	exonic	synonymous SNV	NM_002585	9	c.T1233C	p.P411P	CCG	0.06557377	114	8	SNV
YHIM-01	Non-synergy	PCLO	chr7	82579011	82579011	T	C	exonic	synonymous SNV	NM_014510	6	c.A10893G	p.S3631S		0.44748858	121	98	SNV
YHIM-01	Non-synergy	PDE10A	chr6	165809847	165809847	C	A	exonic	nonsynonymous SNV	NM_001130690	14	c.G1380T	p.M460I		0.32820513	131	64	SNV
YHIM-01	Non-synergy	PDE1A	chr2	183033008	183033008	G	A	exonic	nonsynonymous SNV	NM_001258314	13	c.C1472T	p.T491I		0.55714286	31	39	SNV
YHIM-01	Non-synergy	PIAS3	chr1	145585484	145585484	C	T	exonic	synonymous SNV	NM_006099	14	c.C1749T	p.S583S		0.2815534	74	29	SNV
YHIM-01	Non-synergy	PKD1	chr16	2152406	2152406	-	G	exonic	frameshift insertion	NM_000296	25	c.9176dupC	p.P3059fs					Indel
YHIM-01	Non-synergy	PLCG1	chr20	39798141	39798141	C	G	exonic	synonymous SNV	NM_002660	23	c.C2625G	p.V875V	CCG	0.2260274	113	33	SNV
YHIM-01	Non-synergy	PLCH1	chr3	155200090	155200090	G	T	exonic	nonsynonymous SNV	NM_014996	22	c.C3635A	p.S1212Y		0.17207792	255	53	SNV
YHIM-01	Non-synergy	POU6F2	chr7	39125590	39125590	T	C	exonic	nonsynonymous SNV	NM_001166018	3	c.T149C	p.V50A		0.2195122	128	36	SNV
YHIM-01	Non-synergy	POU6F2	chr7	39125588	39125588	T	C	exonic	synonymous SNV	NM_001166018	3	c.T147C	p.P49P		0.21637427	134	37	SNV
YHIM-01	Non-synergy	PRB2	chr12	11546036	11546036	G	A	exonic	stopgain	NM_006248	3	c.C976T	p.Q326X		0.6119403	26	41	SNV
YHIM-01	Non-synergy	PRR25	chr16	863442	863442	C	T	exonic	nonsynonymous SNV	NM_001013638	3	c.C790T	p.R264W		0.44270833	107	85	SNV
YHIM-01	Non-synergy	PRRX1	chr1	170633575	170633575	C	T	exonic	synonymous SNV	NM_006902	1	c.C216T	p.S72S	CCG	0.50847458	29	30	SNV
YHIM-01	Non-synergy	PTDSS2	chr11	473923	473923	T	A	exonic	nonsynonymous SNV	NM_030783	3	c.T313A	p.L105I		0.33195021	161	80	SNV
YHIM-01	Non-synergy	RBMX	chrX	135961560	135961560	C	G	exonic	nonsynonymous SNV	NM_001164803	2	c.G27C	p.K9N		0.11111111	32	4	SNV
YHIM-01	Non-synergy	RBMXL3	chrX	114426551	114426551	C	T	exonic	synonymous SNV	NM_001145346	1	c.C2547T	p.Y849Y		1	0	194	SNV
YHIM-01	Non-synergy	REG3G	chr2	79254226	79254226	G	A	exonic	nonsynonymous SNV	NM_001008387	4	c.G262A	p.V88M		0.47169811	168	150	SNV
YHIM-01	Non-synergy	RELN	chr7	103137054	103137054	G	C	exonic	nonsynonymous SNV	NM_005045	56	c.C9112G	p.R3038G		0.47478992	125	113	SNV
YHIM-01	Non-synergy	RIN3	chr14	93118070	93118070	T	C	exonic	nonsynonymous SNV	NM_024832	6	c.T676C	p.S226P		0.20754717	168	44	SNV
YHIM-01	Non-synergy	RPS6KB2	chr11	67197061	67197061	A	T	exonic	nonsynonymous SNV	NM_003952	4	c.A304T	p.R102W		0.23364486	82	25	SNV
YHIM-01	Non-synergy	RRP7A	chr22	42910199	42910199	G	A	exonic	nonsynonymous SNV	NM_015703	6	c.C670T	p.R224W		0.06666667	56	4	SNV
YHIM-01	Non-synergy	SAMD4A	chr14	55203869	55203869	C	T	exonic	synonymous SNV	NM_015589	3	c.C843T	p.P281P		0.2161017	185	51	SNV
YHIM-01	Non-synergy	SEC61A2	chr10	12191911	12191911	G	A	exonic	nonsynonymous SNV	NM_001142628	5	c.G347A	p.G116E		0.52238806	64	70	SNV
YHIM-01	Non-synergy	SENP5	chr3	196612862	196612862	A	C	exonic	nonsynonymous SNV	NM_001308045	2	c.A810C	p.K270N		0.27719298	206	79	SNV
YHIM-01	Non-synergy	SIX4	chr14	61190147	61190147	G	A	exonic	synonymous SNV	NM_017420	1	c.C646T	p.L216L		0.05696203	298	18	SNV
YHIM-01	Non-synergy	SLA2	chr20	35242302	35242302	C	G	exonic	synonymous SNV	NM_032214	8	c.G753C	p.L251L		0.42857143	120	90	SNV
YHIM-01	Non-synergy	SLC14A2	chr18	43262454	43262454	A	G	exonic	nonsynonymous SNV	NM_007163	20	c.A2733G	p.I911M		0.63888889	13	23	SNV
YHIM-01	Non-synergy	SLC16A7	chr12	60098608	60098608	C	G	exonic	nonsynonymous SNV	NM_004731	2	c.C26G	p.P9R		0.45652174	25	21	SNV
YHIM-01	Non-synergy	SLC25A25	chr9	130830684	130830684	C	T	exonic	nonsynonymous SNV	NM_001006641	1	c.C86T	p.P29L		0.1980198	81	20	SNV
YHIM-01	Non-synergy	SLC6A17	chr1	110740892	110740892	C	T	exonic	synonymous SNV	NM_001010898	12	c.C2010T	p.N670N		0.05923345	270	17	SNV
YHIM-01	Non-synergy	SLF2	chr10	102672968	102672968	A	C	exonic	nonsynonymous SNV	NM_001136123	1	c.A101C	p.H34P		0.04861111	137	7	SNV

YHIM-01	Non-synergy	SLTM	chr15	59224565	59224565	A	G	exonic	synonymous SNV	NM_001013843	2	c.T240C	p.T80T		0.06849315	68	5	SNV
YHIM-01	Non-synergy	SNAI1	chr20	48599670	48599670	C	G	exonic	nonsynonymous SNV	NM_005985	1	c.C74G	p.S25C		0.19863014	234	58	SNV
YHIM-01	Non-synergy	SRRD	chr22	26879947	26879967	GAGC	-	exonic	nonframeshift deletion	NM_001013694	1	c.91_111del	p.31_37del					Indel
YHIM-01	Non-synergy	SRRM2	chr16	2806561	2806561	G	A	exonic	nonsynonymous SNV	NM_016333	2	c.G196A	p.V66I		0.04861111	137	7	SNV
YHIM-01	Non-synergy	STRBP	chr9	125921478	125921478	T	C	exonic	synonymous SNV	NM_001171137	9	c.A690G	p.E230E		0.14285714	138	23	SNV
YHIM-01	Non-synergy	TACR3	chr4	104510988	104510988	C	T	exonic	nonsynonymous SNV	NM_001059	5	c.G1249A	p.D417N		0.50462963	107	109	SNV
YHIM-01	Non-synergy	TENM1	chrX	123518367	123518367	G	A	exonic	synonymous SNV	NM_014253	29	c.C6393T	p.G2131G		0.43478261	91	70	SNV
YHIM-01	Non-synergy	TMEM120B	chr12	122190111	122190111	G	A	exonic	nonsynonymous SNV	NM_001080825	5	c.G443A	p.R148Q		0.5188172	179	193	SNV
YHIM-01	Non-synergy	TP53	chr17	7578517	7578517	G	A	exonic	nonsynonymous SNV	NM_001126115	1	c.C17T	p.A6V	CCG	0.5	49	49	SNV
YHIM-01	Non-synergy	TP53	chr17	7578265	7578265	A	G	exonic	nonsynonymous SNV	NM_001126115	2	c.T188C	p.I63T	CCG	0.49726776	92	91	SNV
YHIM-01	Non-synergy	TRIM48	chr11	55032500	55032500	C	T	exonic	nonsynonymous SNV	NM_024114	2	c.C169T	p.L57F		0.23076923	10	3	SNV
YHIM-01	Non-synergy	TRPS1	chr8	116426279	116426279	A	G	exonic	nonsynonymous SNV	NM_001282902	6	c.T3830C	p.V1277A		0.03363914	316	11	SNV
YHIM-01	Non-synergy	TRPS1	chr8	116426278	116426278	C	T	exonic	synonymous SNV	NM_001282902	6	c.G3831A	p.V1277V		0.0345912	307	11	SNV
YHIM-01	Non-synergy	USP34	chr2	61507446	61507446	C	A	exonic	nonsynonymous SNV	NM_014709	39	c.G5189T	p.G1730V		0.22222222	21	6	SNV
YHIM-01	Non-synergy	USP34	chr2	61441453	61441453	G	C	exonic	synonymous SNV	NM_014709	68	c.C8424G	p.V2808V		0.03333333	435	15	SNV
YHIM-01	Non-synergy	UTF1	chr10	135044495	135044495	G	A	exonic	nonsynonymous SNV	NM_003577	2	c.G575A	p.R192H		0.5	7	7	SNV
YHIM-01	Non-synergy	VWF	chr12	6153597	6153597	G	C	exonic	nonsynonymous SNV	NM_000552	18	c.C2302G	p.R768G		0.2810219	197	77	SNV
YHIM-01	Non-synergy	WHAMM	chr15	83501995	83501995	G	T	exonic	nonsynonymous SNV	NM_001080435	10	c.G2137T	p.V713L		0.4375	54	42	SNV
YHIM-01	Non-synergy	WNK1	chr12	977564	977564	G	T	exonic	nonsynonymous SNV	NM_001184985	9	c.G2672T	p.G891V		0.15	85	15	SNV
YHIM-01	Non-synergy	YWHAZ	chr8	101932970	101932970	G	C	exonic	nonsynonymous SNV	NM_001135699	6	c.C689G	p.S230W		0.10638298	42	5	SNV
YHIM-01	Non-synergy	ZFYVE9	chr1	52732439	52732439	T	C	exonic	synonymous SNV	NM_004799	6	c.T2391C	p.A797A		0.04575163	146	7	SNV
YHIM-01	Non-synergy	ZIC3	chrX	136652205	136652205	T	C	exonic	synonymous SNV	NM_003413	3	c.T1380C	p.P460P		0.08433735	76	7	SNV
YHIM-01	Non-synergy	ZNF131	chr5	43161698	43161698	C	T	exonic	nonsynonymous SNV	NM_001297548	5	c.C719T	p.T240M		0.06451613	406	28	SNV
YHIM-01	Non-synergy	ZNF131	chr5	43161813	43161813	G	A	exonic	synonymous SNV	NM_001297548	5	c.G834A	p.E278E		0.22519084	203	59	SNV
YHIM-01	Non-synergy	ZNF207	chr17	30693698	30693698	C	A	exonic	synonymous SNV	NM_003457	8	c.C795A	p.V265V		0.06375839	279	19	SNV
YHIM-01	Non-synergy	ZNF316	chr7	6693683	6693684	CG	-	exonic	frameshift deletion	NM_001278559	9	c.2456_2457del	p.T819fs					Indel
YHIM-01	Non-synergy	ZNF326	chr1	90472996	90472996	G	C	exonic	nonsynonymous SNV	NM_182976	5	c.G302C	p.S101T		0.05660377	150	9	SNV
YHIM-01	Non-synergy	ZNF419	chr19	58004744	58004744	C	T	exonic	synonymous SNV	NM_001098495	3	c.C684T	p.H228H		0.58947368	39	56	SNV
YHIM-01	Non-synergy	ZNF479	chr7	57188013	57188013	C	T	exonic	nonsynonymous SNV	NM_033273	5	c.G1109A	p.R370K		0.26086957	68	24	SNV
YHIM-01	Non-synergy	ZNF679	chr7	63726907	63726907	A	T	exonic	nonsynonymous SNV	NM_153363	5	c.A896T	p.E299V		0.52272727	21	23	SNV
YHIM-01	Non-synergy	ZNF729	chr19	22497579	22497579	C	A	exonic	nonsynonymous SNV	NM_001242680	4	c.C1360A	p.H454N		0.53488372	20	23	SNV
YHIM-02	Non-synergy	ABCA5	chr17	67255877	67255877	C	A	exonic	nonsynonymous SNV	NM_018672	27	c.G3701T	p.G1234V		0.20408163	39	10	SNV
YHIM-02	Non-synergy	ABCC10	chr6	43400075	43400075	G	T	exonic	synonymous SNV	NM_033450	1	c.G228T	p.L76L		0.7456446	73	214	SNV
YHIM-02	Non-synergy	ABRA	chr8	107781884	107781884	G	C	exonic	nonsynonymous SNV	NM_139166	1	c.C535G	p.Q179E		0.22758621	224	66	SNV
YHIM-02	Non-synergy	ACAN	chr15	89400137	89400137	A	G	exonic	nonsynonymous SNV	NM_001135	12	c.A4321G	p.T1441A		0.33333333	8	4	SNV
YHIM-02	Non-synergy	ACAN	chr15	89400157	89400157	G	C	exonic	nonsynonymous SNV	NM_001135	12	c.G4341C	p.E1447D		0.41176471	10	7	SNV
YHIM-02	Non-synergy	ACTN1	chr14	69343941	69343941	G	A	exonic	nonsynonymous SNV	NM_001102	20	c.C2378T	p.A793V		0.1980198	243	60	SNV
YHIM-02	Non-synergy	ACTN1	chr14	69343940	69343940	G	A	exonic	synonymous SNV	NM_001102	20	c.C2379T	p.A793A		0.197411	248	61	SNV
YHIM-02	Non-synergy	ACTRT2	chr1	2939096	2939096	C	A	exonic	nonsynonymous SNV	NM_080431	1	c.C846A	p.S282R		0.37278107	106	63	SNV
YHIM-02	Non-synergy	ADAM18	chr8	39466580	39466580	T	A	exonic	nonsynonymous SNV	NM_001190956	4	c.T208A	p.F70I		0.24590164	46	15	SNV
YHIM-02	Non-synergy	ADAMTS14	chr10	72503896	72503896	C	A	exonic	synonymous SNV	NM_080722	14	c.C2133A	p.S711S		0.20231214	276	70	SNV
YHIM-02	Non-synergy	AIM2	chr1	159043057	159043057	G	T	exonic	nonsynonymous SNV	NM_004833	2	c.C233A	p.A78E		0.25	249	83	SNV
YHIM-02	Non-synergy	AKAP4	chrX	49957914	49957914	A	T	exonic	nonsynonymous SNV	NM_003886	5	c.T1450A	p.S484T		0.07924107	825	71	SNV
YHIM-02	Non-synergy	AKAP4	chrX	49957913	49957913	G	T	exonic	stopgain	NM_003886	5	c.C1451A	p.S484X		0.08044693	823	72	SNV
YHIM-02	Non-synergy	AMN1	chr12	31854817	31854817	C	T	exonic	nonsynonymous SNV	NM_001113402	3	c.G296A	p.R99Q		0.27692308	47	18	SNV
YHIM-02	Non-synergy	AMOTL1	chr11	94603931	94603931	G	A	exonic	nonsynonymous SNV	NM_001301007	12	c.G2692A	p.D898N		0.39669422	73	48	SNV
YHIM-02	Non-synergy	ANK3	chr10	61835960	61835960	G	C	exonic	stopgain	NM_020987	37	c.C4679G	p.S1560X		0.06846473	898	66	SNV
YHIM-02	Non-synergy	ANKRD6	chr6	90312802	90312802	G	A	exonic	nonsynonymous SNV	NM_001242814	3	c.G274A	p.A92T		0.51724138	140	150	SNV
YHIM-02	Non-synergy	ARAP1	chr11	72437838	72437838	G	A	exonic	synonymous SNV	NM_001040118	3	c.C336T	p.P112P		0.19512195	33	8	SNV
YHIM-02	Non-synergy	ARHGAP25	chr2	69049610	69049610	C	T	exonic	nonsynonymous SNV	NM_001166276	9	c.C1318T	p.R440W		0.22648084	222	65	SNV
YHIM-02	Non-synergy	ARHGAP44	chr17	12888175	12888175	C	T	exonic	nonsynonymous SNV	NM_014859	20	c.C2267T	p.T756M		0.25142857	131	44	SNV
YHIM-02	Non-synergy	ARHGEF11	chr1	156917676	156917676	G	T	exonic	synonymous SNV	NM_014784	24	c.C2106A	p.G702G		0.25225225	83	28	SNV
YHIM-02	Non-synergy	ARMCX4	chrX	100747620	100747620	C	A	exonic	synonymous SNV	NM_001256155	2	c.C4044A	p.S1348S		0.09027778	131	13	SNV
YHIM-02	Non-synergy	ATP6V1H	chr8	54730070	54730070	G	T	exonic	nonsynonymous SNV	NM_015941	5	c.C327A	p.S109R		0.18320611	214	48	SNV
YHIM-02	Non-synergy	ATR	chr3	142279184	142279184	C	A	exonic	stopgain	NM_001184	6	c.G1462T	p.E488X	CCG	0.31055901	111	50	SNV
YHIM-02	Non-synergy	BCKDHB	chr6	80982908	80982908	C	A	exonic	synonymous SNV	NM_000056	9	c.C1008A	p.G336G		0.27536232	150	57	SNV
YHIM-02	Non-synergy	BIVM,BIVM-E	chr13	103459845	103459845	C	G	exonic	synonymous SNV	NM_001204425	1	c.C228G	p.L76L		0.4549763	115	96	SNV
YHIM-02	Non-synergy	BYSL	chr6	41889319	41889319	G	T	exonic	nonsynonymous SNV	NM_004053	1	c.G19T	p.A7S		0.2631579	98	35	SNV
YHIM-02	Non-synergy	C1orf167	chr1	11844564	11844564	C	T	exonic	synonymous SNV	NM_001010881	15	c.C3339T	p.C1113C		0.21212121	52	14	SNV
YHIM-02	Non-synergy	C2CD2L	chr11	118983574	118983574	C	T	exonic	synonymous SNV	NM_001290474	10	c.C1377T	p.D459D		0.28787879	47	19	SNV
YHIM-02	Non-synergy	C6orf222	chr6	36298377	36298377	A	G	exonic	nonsynonymous SNV	NM_001010903	2	c.T91C	p.S31P		0.18	164	36	SNV
YHIM-02	Non-synergy	C9orf114	chr9	131592038	131592038	G	A	exonic	nonsynonymous SNV	NM_016390	1	c.C22T	p.R8W		0.42021277	109	79	SNV

YHIM-02	Non-synergy	CACNA1A	chr19	13323204	13323204	G	T	exonic	nonsynonymous SNV	NM_001127221	42	c.C6186A	p.N2062K		0.232	96	29	SNV
YHIM-02	Non-synergy	CACNA1C	chr12	2602536	2602536	C	T	exonic	nonsynonymous SNV	NM_000719	7	c.C1097T	p.T366M		0.26027397	162	57	SNV
YHIM-02	Non-synergy	CADPS2	chr7	122377021	122377021	C	A	exonic	synonymous SNV	NM_001009571	2	c.G441T	p.R147R		0.29651163	121	51	SNV
YHIM-02	Non-synergy	CBX4	chr17	77808441	77808441	C	T	exonic	nonsynonymous SNV	NM_003655	5	c.G1000A	p.G334R		0.24137931	88	28	SNV
YHIM-02	Non-synergy	CCDC129	chr7	31681850	31681850	C	T	exonic	nonsynonymous SNV	NM_194300	9	c.C1289T	p.P430L		0.22222222	42	12	SNV
YHIM-02	Non-synergy	CCDC129	chr7	31682367	31682367	C	T	exonic	synonymous SNV	NM_194300	10	c.C1383T	p.S461S		0.54634146	93	112	SNV
YHIM-02	Non-synergy	CCND1	chr11	69457896	69457896	T	C	exonic	nonsynonymous SNV	NM_053056	2	c.T296C	p.L99P	CCG	0.09868421	411	45	SNV
YHIM-02	Non-synergy	CCND1	chr11	69457897	69457897	G	A	exonic	synonymous SNV	NM_053056	2	c.G297A	p.L99L	CCG	0.09868421	411	45	SNV
YHIM-02	Non-synergy	CCT6B	chr17	33269517	33269517	C	T	exonic	nonsynonymous SNV	NM_001193529	6	c.G760A	p.V254I		0.15789474	32	6	SNV
YHIM-02	Non-synergy	CCT8L2	chr22	17072784	17072784	G	T	exonic	synonymous SNV	NM_014406	1	c.C657A	p.L219L		0.35443038	102	56	SNV
YHIM-02	Non-synergy	CD22	chr19	35831923	35831923	G	A	exonic	synonymous SNV	NM_001185101	5	c.G858A	p.E286E		0.2428115	237	76	SNV
YHIM-02	Non-synergy	CDC42BPA	chr1	227259989	227259989	T	A	exonic	nonsynonymous SNV	NM_014826	19	c.A2504T	p.D835V		0.24444444	102	33	SNV
YHIM-02	Non-synergy	CDKN2A	chr9	21974723	21974738	CCCG	-	exonic	frameshift deletion	NM_000077	1	c.89_104del	p.A30fs	CCG				Indel
YHIM-02	Non-synergy	CDR1	chrX	139865777	139865777	G	T	exonic	nonsynonymous SNV	NM_004065	1	c.C755A	p.S252Y		0.164	209	41	SNV
YHIM-02	Non-synergy	CDR1	chrX	139865778	139865778	A	T	exonic	nonsynonymous SNV	NM_004065	1	c.T754A	p.S252T		0.16015625	215	41	SNV
YHIM-02	Non-synergy	CECR6	chr22	17601051	17601051	T	C	exonic	nonsynonymous SNV	NM_031890	1	c.A967G	p.I323V		0.34065934	120	62	SNV
YHIM-02	Non-synergy	CELF4	chr18	34846524	34846524	G	T	exonic	synonymous SNV	NM_001025087	9	c.C1134A	p.A378A		0.34285714	69	36	SNV
YHIM-02	Non-synergy	CHRM2	chr7	136700059	136700059	C	A	exonic	synonymous SNV	NM_001006629	2	c.C447A	p.V149V		0.47115385	55	49	SNV
YHIM-02	Non-synergy	CHRNA10	chr11	3688868	3688868	G	A	exonic	synonymous SNV	NM_020402	4	c.C489T	p.F163F		0.59574468	76	112	SNV
YHIM-02	Non-synergy	CLCA4	chr1	87040412	87040412	T	A	exonic	nonsynonymous SNV	NM_012128	10	c.T1657A	p.Y553N		0.09655172	131	14	SNV
YHIM-02	Non-synergy	CLPSL1	chr6	35755677	35755677	C	A	exonic	nonsynonymous SNV	NM_001010886	3	c.C256A	p.L86M		0.15469613	153	28	SNV
YHIM-02	Non-synergy	CLPSL1	chr6	35755676	35755676	C	A	exonic	stopgain	NM_001010886	3	c.C255A	p.C85X		0.15469613	153	28	SNV
YHIM-02	Non-synergy	COL3A1	chr2	189864109	189864109	G	A	exonic	synonymous SNV	NM_000090	30	c.G2121A	p.K707K		0.17213115	101	21	SNV
YHIM-02	Non-synergy	COL6A3	chr2	238296513	238296513	C	T	exonic	nonsynonymous SNV	NM_057165	3	c.G406A	p.V136M		0.46089386	193	165	SNV
YHIM-02	Non-synergy	CR1	chr1	207793286	207793286	A	T	exonic	nonsynonymous SNV	NM_000573	35	c.A5778T	p.K1926N		0.25510204	73	25	SNV
YHIM-02	Non-synergy	CRYBB1	chr22	27012261	27012261	G	A	exonic	nonsynonymous SNV	NM_001887	2	c.C23T	p.S8L		0.35051546	63	34	SNV
YHIM-02	Non-synergy	CSNK2A1	chr20	469398	469398	C	T	exonic	nonsynonymous SNV	NM_177560	9	c.G340A	p.G114R		0.12440191	183	26	SNV
YHIM-02	Non-synergy	CYLC2	chr9	105767087	105767087	G	T	exonic	nonsynonymous SNV	NM_001340	4	c.G291T	p.Q97H		0.21774194	97	27	SNV
YHIM-02	Non-synergy	DARS	chr2	136718971	136718971	A	T	exonic	synonymous SNV	NM_001293312	3	c.T15A	p.A5A		0.04347826	242	11	SNV
YHIM-02	Non-synergy	DCAF5	chr14	69520962	69520962	C	T	exonic	nonsynonymous SNV	NM_001284206	9	c.G2438A	p.R813K		0.29299363	111	46	SNV
YHIM-02	Non-synergy	DCC	chr18	50985665	50985665	T	A	exonic	nonsynonymous SNV	NM_005215	24	c.T3456A	p.D1152E		0.24324324	28	9	SNV
YHIM-02	Non-synergy	DCLK1	chr13	36348751	36348751	G	T	exonic	nonsynonymous SNV	NM_001195416	13	c.C1223A	p.P408H		0.06451613	232	16	SNV
YHIM-02	Non-synergy	DHTKD1	chr10	12162226	12162226	C	T	exonic	nonsynonymous SNV	NM_018706	16	c.C2618T	p.S873L		0.53153153	52	59	SNV
YHIM-02	Non-synergy	DHX40	chr17	57663558	57663558	C	G	exonic	nonsynonymous SNV	NM_001166301	10	c.C1157G	p.A386G		0.43697479	67	52	SNV
YHIM-02	Non-synergy	DHX57	chr2	39089411	39089411	G	T	exonic	nonsynonymous SNV	NM_198963	4	c.C448A	p.P150T		0.23469388	75	23	SNV
YHIM-02	Non-synergy	DIAPH2	chrX	96854299	96854299	G	A	exonic	nonsynonymous SNV	NM_006729	27	c.G3289A	p.A1097T		0.21590909	138	38	SNV
YHIM-02	Non-synergy	DNAH17	chr17	76456023	76456023	C	T	exonic	nonsynonymous SNV	NM_173628	60	c.G9496A	p.A3166T		0.66292135	30	59	SNV
YHIM-02	Non-synergy	DPP10	chr2	116497394	116497394	T	A	exonic	synonymous SNV	NM_001004360	9	c.T756A	p.S252S		0.30603448	161	71	SNV
YHIM-02	Non-synergy	DPP4	chr2	162875296	162875296	G	A	exonic	stopgain	NM_001935	16	c.C1363T	p.Q455X		0.26808511	172	63	SNV
YHIM-02	Non-synergy	DYNC1H1	chr14	102452896	102452896	C	A	exonic	synonymous SNV	NM_001376	8	c.C2334A	p.A778A		0.16666667	210	42	SNV
YHIM-02	Non-synergy	EIF4A1	chr17	7480003	7480003	A	T	exonic	nonsynonymous SNV	NM_001204510	5	c.A507T	p.R169S		0.25321888	174	59	SNV
YHIM-02	Non-synergy	EMILIN1	chr2	27308777	27308777	C	T	exonic	nonsynonymous SNV	NM_007046	8	c.C2945T	p.T982M		0.51515152	32	34	SNV
YHIM-02	Non-synergy	ENTHD1	chr22	40257925	40257925	C	A	exonic	nonsynonymous SNV	NM_152512	3	c.G437T	p.R146L		0.27884615	75	29	SNV
YHIM-02	Non-synergy	EPB41L5	chr2	120861812	120861812	T	A	exonic	nonsynonymous SNV	NM_001184938	17	c.T1514A	p.I505K		0.3	98	42	SNV
YHIM-02	Non-synergy	ETFB	chr19	51856535	51856535	G	A	exonic	nonsynonymous SNV	NM_001014763	2	c.C499T	p.R167C		0.3919598	121	78	SNV
YHIM-02	Non-synergy	EVI2B	chr17	29632292	29632292	T	C	exonic	nonsynonymous SNV	NM_006495	2	c.A336G	p.I112M		0.25815217	273	95	SNV
YHIM-02	Non-synergy	EXOSC10	chr1	11128119	11128119	A	C	exonic	nonsynonymous SNV	NM_002685	23	c.T2498G	p.I833S		0.30167598	125	54	SNV
YHIM-02	Non-synergy	FAM120C	chrX	54209435	54209435	G	A	exonic	nonsynonymous SNV	NM_001300788	1	c.C197T	p.P66L		0.76760563	66	218	SNV
YHIM-02	Non-synergy	FAM160B2	chr8	21960404	21960404	C	T	exonic	nonsynonymous SNV	NM_022749	17	c.C2194T	p.R732C		0.3033419	271	118	SNV
YHIM-02	Non-synergy	FAM65B	chr6	24873918	24873918	G	A	exonic	nonsynonymous SNV	NM_001286445	3	c.C298T	p.P100S		0.31845238	229	107	SNV
YHIM-02	Non-synergy	FAM71D	chr14	67671688	67671688	A	G	exonic	nonsynonymous SNV	NM_173526	5	c.A794G	p.E265G		0.19282511	180	43	SNV
YHIM-02	Non-synergy	FARSA	chr19	13035972	13035972	C	T	exonic	synonymous SNV	NM_004461	8	c.G858A	p.L286L		0.33333333	14	7	SNV
YHIM-02	Non-synergy	FHOD1	chr16	67265699	67265699	C	T	exonic	synonymous SNV	NM_013241	15	c.G2226A	p.T742T		0.30769231	90	40	SNV
YHIM-02	Non-synergy	FOXO1	chr13	41239879	41239879	G	T	exonic	synonymous SNV	NM_002015	1	c.C471A	p.R157R	CCG	0.46031746	102	87	SNV
YHIM-02	Non-synergy	FRMPD2	chr10	49392856	49392856	G	T	exonic	nonsynonymous SNV	NM_001018071	19	c.C2428A	p.P810T		0.20930233	136	36	SNV
YHIM-02	Non-synergy	FRS2	chr12	69968441	69968441	G	T	exonic	nonsynonymous SNV	NM_001278357	7	c.G1233T	p.R411S		0.34841629	144	77	SNV
YHIM-02	Non-synergy	GABRP	chr5	170232844	170232844	G	A	exonic	synonymous SNV	NM_001291985	7	c.G666A	p.S222S		0.73780488	43	121	SNV
YHIM-02	Non-synergy	GALNS	chr16	88901685	88901685	C	T	exonic	synonymous SNV	NM_000512	8	c.G834A	p.A278A		0.32967033	122	60	SNV
YHIM-02	Non-synergy	GANAB	chr11	62406552	62406552	G	A	exonic	stopgain	NM_198334	4	c.C283T	p.Q95X		0.18285714	143	32	SNV
YHIM-02	Non-synergy	GDF11	chr12	56143355	56143355	G	A	exonic	nonsynonymous SNV	NM_005811	3	c.G913A	p.D305N		0.25882353	189	66	SNV
YHIM-02	Non-synergy	GJA10	chr6	90605632	90605632	G	A	exonic	nonsynonymous SNV	NM_032602	1	c.G1445A	p.R482K		0.23039216	157	47	SNV
YHIM-02	Non-synergy	GJC2	chr1	228345916	228345916	G	A	exonic	nonsynonymous SNV	NM_020435	2	c.G457A	p.E153K		0.17647059	14	3	SNV

YHIM-02	Non-synergy	GLTSCR1	chr19	48184336	48184336	C	T	exonic	synonymous SNV	NM_015711	6	c.C1909T	p.L637L		0.53846154	6	7	SNV
YHIM-02	Non-synergy	GNAS	chr20	57415846	57415846	G	A	exonic	nonsynonymous SNV	NM_016592	1	c.G685A	p.A229T	CCG	0.2145749	194	53	SNV
YHIM-02	Non-synergy	GPATCH8	chr17	42477061	42477061	C	A	exonic	nonsynonymous SNV	NM_001304943	5	c.G2150T	p.C717F		0.25165563	226	76	SNV
YHIM-02	Non-synergy	GPHN	chr14	67291202	67291202	T	A	exonic	nonsynonymous SNV	NM_001024218	4	c.T212A	p.I71K	CCG	0.40525328	317	216	SNV
YHIM-02	Non-synergy	GPR158	chr10	25701248	25701248	C	A	exonic	nonsynonymous SNV	NM_020752	4	c.C1181A	p.P394H		0.45081967	67	55	SNV
YHIM-02	Non-synergy	GPR158	chr10	25701249	25701249	T	C	exonic	synonymous SNV	NM_020752	4	c.T1182C	p.P394P		0.45528455	67	56	SNV
YHIM-02	Non-synergy	GREB1	chr2	11725980	11725980	G	A	exonic	nonsynonymous SNV	NM_014668	9	c.G1108A	p.V370I		0.22330097	80	23	SNV
YHIM-02	Non-synergy	GRIN3B	chr19	1009376	1009376	G	A	exonic	synonymous SNV	NM_138690	9	c.G2907A	p.P969P		0.27272727	8	3	SNV
YHIM-02	Non-synergy	HCK	chr20	30689215	30689215	C	T	exonic	nonsynonymous SNV	NM_001172129	13	c.C1411T	p.R471C		0.15969582	221	42	SNV
YHIM-02	Non-synergy	HCN3	chr1	155257687	155257687	G	T	exonic	synonymous SNV	NM_020897	8	c.G1758T	p.R586R		0.17834395	258	56	SNV
YHIM-02	Non-synergy	HELZ2	chr20	62196465	62196465	A	G	exonic	nonsynonymous SNV	NM_033405	3	c.T2003C	p.V668A		0.2971246	220	93	SNV
YHIM-02	Non-synergy	HERC6	chr4	89300135	89300135	C	A	exonic	nonsynonymous SNV	NM_001165136	1	c.C62A	p.P21H		0.44761905	58	47	SNV
YHIM-02	Non-synergy	HOXC10	chr12	54379481	54379481	C	T	exonic	synonymous SNV	NM_017409	1	c.C438T	p.P146P		0.20264317	181	46	SNV
YHIM-02	Non-synergy	HTR1F	chr3	88040464	88040464	G	C	exonic	nonsynonymous SNV	NM_000866	2	c.G565C	p.A189P		0.32692308	210	102	SNV
YHIM-02	Non-synergy	HUNK	chr21	33355902	33355902	G	T	exonic	nonsynonymous SNV	NM_014586	8	c.G1237T	p.A413S		0.32142857	19	9	SNV
YHIM-02	Non-synergy	ICK	chr6	52897392	52897392	C	T	exonic	nonsynonymous SNV	NM_014920	4	c.G217A	p.D73N		0.13953488	74	12	SNV
YHIM-02	Non-synergy	IDO1	chr8	39775472	39775472	C	G	exonic	nonsynonymous SNV	NM_002164	2	c.C166G	p.R56G		0.64285714	40	72	SNV
YHIM-02	Non-synergy	IFIT3	chr10	91099579	91099579	C	A	exonic	nonsynonymous SNV	NM_001031683	2	c.C1167A	p.S389R		0.26573427	210	76	SNV
YHIM-02	Non-synergy	IL18	chr11	112019378	112019378	T	G	exonic	nonsynonymous SNV	NM_001243211	4	c.A296C	p.K99T		0.27272727	24	9	SNV
YHIM-02	Non-synergy	INHBC	chr12	57843372	57843372	C	T	exonic	nonsynonymous SNV	NM_005538	2	c.C626T	p.S209L		0.23809524	240	75	SNV
YHIM-02	Non-synergy	IPO13	chr1	44424418	44424418	C	G	exonic	nonsynonymous SNV	NM_014652	11	c.C1885G	p.P629A		0.32298137	109	52	SNV
YHIM-02	Non-synergy	JMJD1C	chr10	64974452	64974452	T	C	exonic	nonsynonymous SNV	NM_001282948	7	c.A929G	p.E310G		0.07002188	425	32	SNV
YHIM-02	Non-synergy	KALRN	chr3	124352709	124352709	G	T	exonic	nonsynonymous SNV	NM_007064	3	c.G383T	p.G128V		0.22026432	177	50	SNV
YHIM-02	Non-synergy	KCNB1	chr20	48098796	48098796	A	T	exonic	nonsynonymous SNV	NM_004975	1	c.T222A	p.D74E		0.27810651	122	47	SNV
YHIM-02	Non-synergy	KCNC1	chr11	17757696	17757696	C	A	exonic	nonsynonymous SNV	NM_001112741	1	c.C147A	p.D49E		0.26285714	129	46	SNV
YHIM-02	Non-synergy	KCNG1	chr20	49620701	49620701	C	A	exonic	stopgain	NM_002237	3	c.G1417T	p.E473X		0.75070822	88	265	SNV
YHIM-02	Non-synergy	KIF5C	chr2	149806447	149806447	C	A	exonic	nonsynonymous SNV	NM_004522	9	c.C809A	p.A270E		0.61870504	53	86	SNV
YHIM-02	Non-synergy	KLHL13	chrX	117053621	117053621	G	T	exonic	nonsynonymous SNV	NM_001168300	4	c.C415A	p.L139I		0.07111111	209	16	SNV
YHIM-02	Non-synergy	KLHL13	chrX	117053622	117053622	A	T	exonic	synonymous SNV	NM_001168300	4	c.T414A	p.G138G		0.07174888	207	16	SNV
YHIM-02	Non-synergy	KRT14	chr17	39742807	39742807	C	T	exonic	nonsynonymous SNV	NM_000526	1	c.G280A	p.A94T		0.13043478	40	6	SNV
YHIM-02	Non-synergy	LACE1	chr6	108843594	108843594	A	T	exonic	nonsynonymous SNV	NM_145315	13	c.A1412T	p.Q471L		0.11976048	294	40	SNV
YHIM-02	Non-synergy	LCT	chr2	136566261	136566261	G	A	exonic	nonsynonymous SNV	NM_002299	8	c.C3656T	p.P1219L		0.30501931	180	79	SNV
YHIM-02	Non-synergy	LDB3	chr10	88476354	88476354	C	T	exonic	nonsynonymous SNV	NM_001080114	9	c.C1172T	p.A391V		0.24852071	127	42	SNV
YHIM-02	Non-synergy	LMBRD2	chr5	36137536	36137536	A	C	exonic	nonsynonymous SNV	NM_001007527	5	c.T376G	p.L126V		0.11764706	75	10	SNV
YHIM-02	Non-synergy	LMNB2	chr19	2435150	2435150	C	T	exonic	nonsynonymous SNV	NM_032737	5	c.G704A	p.R235Q		0.41176471	20	14	SNV
YHIM-02	Non-synergy	LTK	chr15	41804034	41804042	CCCC	-	exonic	nonframeshift deletion	NM_001135685	5	c.630_638del	p.210_213del					Indel
YHIM-02	Non-synergy	MAGEB4	chrX	30260436	30260436	C	A	exonic	nonsynonymous SNV	NM_002367	1	c.C184A	p.P62T		0.11563518	543	71	SNV
YHIM-02	Non-synergy	MAGED2	chrX	54838081	54838081	G	T	exonic	stopgain	NM_014599	6	c.G985T	p.E329X		0.1328125	222	34	SNV
YHIM-02	Non-synergy	MAML3	chr4	140811064	140811075	TGCT	-	exonic	frameshift deletion	NM_018717	3	c.1513_1514del	p.Q505fs					Indel
YHIM-02	Non-synergy	MAML3	chr4	140811084	140811084	C	T	exonic	synonymous SNV	NM_018717	2	c.G1506A	p.Q502Q		0.40350877	68	46	SNV
YHIM-02	Non-synergy	MAP2K6	chr17	67515460	67515460	C	A	exonic	synonymous SNV	NM_002758	5	c.C253A	p.R85R		0.20588235	189	49	SNV
YHIM-02	Non-synergy	MAST4	chr5	66255106	66255106	C	A	exonic	nonsynonymous SNV	NM_001290226	1	c.C31A	p.P11T		0.25	60	20	SNV
YHIM-02	Non-synergy	MATR3	chr5	138643914	138643914	A	G	exonic	synonymous SNV	NM_001194955	2	c.A810G	p.R270R		0.17021277	117	24	SNV
YHIM-02	Non-synergy	MED1	chr17	37566896	37566896	G	A	exonic	synonymous SNV	NM_004774	17	c.C1578T	p.S526S		0.03243243	179	6	SNV
YHIM-02	Non-synergy	MED8	chr1	43853219	43853219	G	A	exonic	synonymous SNV	NM_052877	3	c.C225T	p.V75V		0.28571429	125	50	SNV
YHIM-02	Non-synergy	MEN1	chr11	64577267	64577267	G	T	exonic	synonymous SNV	NM_000244	2	c.C315A	p.L105L	CCG	0.19952494	337	84	SNV
YHIM-02	Non-synergy	MLH3	chr14	75516048	75516048	G	C	exonic	nonsynonymous SNV	NM_001040108	2	c.C311G	p.A104G		0.10185185	485	55	SNV
YHIM-02	Non-synergy	MMRN1	chr4	90856688	90856688	A	C	exonic	nonsynonymous SNV	NM_007351	6	c.A1857C	p.Q619H		0.32065217	125	59	SNV
YHIM-02	Non-synergy	MRGPRF	chr11	68773203	68773203	G	A	exonic	nonsynonymous SNV	NM_001098515	3	c.C575T	p.A192V		0.32608696	93	45	SNV
YHIM-02	Non-synergy	MTMR9	chr8	11163742	11163742	G	A	exonic	nonsynonymous SNV	NM_015458	5	c.G635A	p.R212K		0.75	11	33	SNV
YHIM-02	Non-synergy	MUC12	chr7	100643351	100643351	C	A	exonic	synonymous SNV	NM_001164462	2	c.C9507A	p.G3169G		0.140625	55	9	SNV
YHIM-02	Non-synergy	MYO15A	chr17	18023073	18023073	C	T	exonic	nonsynonymous SNV	NM_016239	2	c.C959T	p.S320L		0.27112232	578	215	SNV
YHIM-02	Non-synergy	MYOM1	chr18	3134739	3134739	C	A	exonic	nonsynonymous SNV	NM_003803	16	c.G2293T	p.A765S		0.18951613	201	47	SNV
YHIM-02	Non-synergy	MYRIP	chr3	40085692	40085692	G	A	exonic	nonsynonymous SNV	NM_001284423	3	c.G262A	p.V88I		0.26890756	174	64	SNV
YHIM-02	Non-synergy	NFATC4	chr14	24839454	24839454	C	G	exonic	nonsynonymous SNV	NM_001198965	2	c.C850G	p.L284V		0.31958763	66	31	SNV
YHIM-02	Non-synergy	NFIA	chr1	61553863	61553863	C	T	exonic	stopgain	NM_001134673	2	c.C70T	p.R24X		0.32026144	104	49	SNV
YHIM-02	Non-synergy	NLRP8	chr19	56465878	56465878	C	T	exonic	nonsynonymous SNV	NM_001317000	3	c.C454T	p.R152W		0.24444444	238	77	SNV
YHIM-02	Non-synergy	NPFFR2	chr4	72897639	72897639	C	A	exonic	synonymous SNV	NM_004885	1	c.C21A	p.T7T		0.37566138	118	71	SNV
YHIM-02	Non-synergy	NRBF2	chr10	64905983	64905983	C	G	exonic	nonsynonymous SNV	NM_030759	2	c.C45G	p.S15R		0.06410256	73	5	SNV
YHIM-02	Non-synergy	NRXN1	chr2	51255107	51255107	G	A	exonic	nonsynonymous SNV	NM_001135659	2	c.C305T	p.T102M		0.99683544	1	315	SNV
YHIM-02	Non-synergy	NTN1	chr17	9083235	9083235	G	T	exonic	nonsynonymous SNV	NM_004822	4	c.G1319T	p.G440V		0.06102362	477	31	SNV
YHIM-02	Non-synergy	NUP155	chr5	37303396	37303396	C	A	exonic	nonsynonymous SNV	NM_001278312	27	c.G3091T	p.A1031S		0.026534	587	16	SNV

YHIM-02	Non-synergy	NWD2	chr4	37246828	37246828	G	A	exonic	nonsynonymous SNV	NM_001144990	1	c.G139A	p.A47T		0.63258786	115	198	SNV
YHIM-02	Non-synergy	OR10G4	chr11	123886341	123886341	G	C	exonic	synonymous SNV	NM_001004462	1	c.G60C	p.G20G		0.32	187	88	SNV
YHIM-02	Non-synergy	OR1C1	chr1	247920899	247920899	G	A	exonic	synonymous SNV	NM_012353	1	c.C810T	p.S270S		0.18076923	213	47	SNV
YHIM-02	Non-synergy	OR2C1	chr16	3406152	3406152	T	A	exonic	nonsynonymous SNV	NM_012368	1	c.T212A	p.L71H		0.28832117	195	79	SNV
YHIM-02	Non-synergy	OR4C46	chr11	51516040	51516040	A	G	exonic	nonsynonymous SNV	NM_001004703	1	c.A759G	p.I253M		0.15841584	85	16	SNV
YHIM-02	Non-synergy	OR56A5	chr11	5989532	5989532	G	T	exonic	nonsynonymous SNV	NM_001146033	1	c.C193A	p.L65M		0.36507937	40	23	SNV
YHIM-02	Non-synergy	OR56A5	chr11	5989531	5989531	A	G	exonic	nonsynonymous SNV	NM_001146033	1	c.T194C	p.L65P		0.36507937	40	23	SNV
YHIM-02	Non-synergy	OR9Q1	chr11	57947761	57947761	C	T	exonic	nonsynonymous SNV	NM_001005212	3	c.C845T	p.P282L		0.37878788	82	50	SNV
YHIM-02	Non-synergy	PBX1	chr1	164768983	164768983	A	G	exonic	synonymous SNV	NM_001204961	4	c.A558G	p.Q186Q	CCG	0.20503597	221	57	SNV
YHIM-02	Non-synergy	PCDH12	chr5	141336390	141336390	G	A	exonic	synonymous SNV	NM_016580	1	c.C1027T	p.L343L		0.31756757	101	47	SNV
YHIM-02	Non-synergy	PCDH9	chr13	67800780	67800780	T	A	exonic	nonsynonymous SNV	NM_020403	2	c.A1793T	p.D598V		0.26582279	174	63	SNV
YHIM-02	Non-synergy	PCDHA1	chr5	140165948	140165948	G	T	exonic	stopgain	NM_018900	1	c.G73T	p.E25X		0.33333333	26	13	SNV
YHIM-02	Non-synergy	PCDHA4	chr5	140186809	140186809	C	T	exonic	nonsynonymous SNV	NM_018907	1	c.C37T	p.R13C		0.20408163	78	20	SNV
YHIM-02	Non-synergy	PCDHAC1	chr5	140308465	140308465	T	C	exonic	nonsynonymous SNV	NM_018898	1	c.T1988C	p.L663S		0.20408163	78	20	SNV
YHIM-02	Non-synergy	PCLO	chr7	82544295	82544295	C	A	exonic	nonsynonymous SNV	NM_014510	7	c.G13007T	p.R4336I		0.52564103	74	82	SNV
YHIM-02	Non-synergy	PEG3	chr19	57327140	57327140	G	T	exonic	nonsynonymous SNV	NM_001146186	7	c.C2670A	p.N890K		0.05776173	261	16	SNV
YHIM-02	Non-synergy	PEG3	chr19	57327139	57327139	A	T	exonic	nonsynonymous SNV	NM_001146186	7	c.T2671A	p.Y891N		0.0569395	265	16	SNV
YHIM-02	Non-synergy	PFKL	chr21	45725269	45725269	G	A	exonic	nonsynonymous SNV	NM_001002021	3	c.G68A	p.S23N		0.41463415	72	51	SNV
YHIM-02	Non-synergy	PLA1A	chr3	119334945	119334945	G	A	exonic	nonsynonymous SNV	NM_001206961	5	c.G232A	p.A78T		0.2	72	18	SNV
YHIM-02	Non-synergy	POGZ	chr1	151377600	151377600	C	T	exonic	stopgain	NM_145796	17	c.G3626A	p.W1209X		0.26048951	423	149	SNV
YHIM-02	Non-synergy	POU4F2	chr4	147561082	147561082	G	C	exonic	nonsynonymous SNV	NM_004575	2	c.G352C	p.D118H		0.36842105	96	56	SNV
YHIM-02	Non-synergy	PRB3	chr12	11420460	11420460	-	G	exonic	frameshift insertion	NM_006249	3	c.722dupC	p.P241fs					Indel
YHIM-02	Non-synergy	PRKACG	chr9	71628947	71628947	G	T	exonic	nonsynonymous SNV	NM_002732	1	c.C62A	p.A21D		0.29207921	143	59	SNV
YHIM-02	Non-synergy	PRMT8	chr12	3692305	3692305	G	A	exonic	nonsynonymous SNV	NM_001256536	8	c.G883A	p.V295I		0.44642857	62	50	SNV
YHIM-02	Non-synergy	PTPRR	chr12	71286531	71286531	A	T	exonic	nonsynonymous SNV	NM_002849	2	c.T285A	p.N95K		0.33828996	178	91	SNV
YHIM-02	Non-synergy	PVALB	chr22	37209787	37209787	T	A	exonic	nonsynonymous SNV	NM_001315532	3	c.A207T	p.K69N		0.07317073	76	6	SNV
YHIM-02	Non-synergy	PZP	chr12	9318729	9318729	T	G	exonic	nonsynonymous SNV	NM_002864	18	c.A2177C	p.N726T		0.14503817	336	57	SNV
YHIM-02	Non-synergy	RABGEF1	chr7	66270184	66270184	A	C	exonic	nonsynonymous SNV	NM_001287061	8	c.A920C	p.K307T		0.1010101	89	10	SNV
YHIM-02	Non-synergy	RAD51AP2	chr2	17698314	17698314	C	T	exonic	nonsynonymous SNV	NM_001099218	1	c.G1369A	p.E457K		0.27067669	97	36	SNV
YHIM-02	Non-synergy	RASIP1	chr19	49224143	49224143	C	T	exonic	nonsynonymous SNV	NM_017805	12	c.G2804A	p.R935H		0.60897436	61	95	SNV
YHIM-02	Non-synergy	RASSF9	chr12	86198932	86198932	C	T	exonic	nonsynonymous SNV	NM_005447	2	c.G856A	p.E286K		0.35555556	203	112	SNV
YHIM-02	Non-synergy	RC3H1	chr1	173961908	173961908	C	G	exonic	nonsynonymous SNV	NM_001300850	1	c.G216C	p.Q72H		0.21818182	86	24	SNV
YHIM-02	Non-synergy	RNF128	chrX	106016238	106016238	G	A	exonic	nonsynonymous SNV	NM_024539	2	c.G502A	p.E168K		0.20979021	339	90	SNV
YHIM-02	Non-synergy	RNF166	chr16	88765464	88765464	C	A	exonic	nonsynonymous SNV	NM_001171815	3	c.G212T	p.C71F		0.3129771	90	41	SNV
YHIM-02	Non-synergy	RNMT	chr18	13731704	13731704	A	G	exonic	nonsynonymous SNV	NM_001308263	3	c.A188G	p.E63G		0.19533528	276	67	SNV
YHIM-02	Non-synergy	ROR2	chr9	94486393	94486393	G	A	exonic	nonsynonymous SNV	NM_004560	9	c.C2383T	p.P795S		0.29323308	94	39	SNV
YHIM-02	Non-synergy	RP1L1	chr8	10468217	10468217	C	T	exonic	nonsynonymous SNV	NM_178857	4	c.G3391A	p.D1131N		0.08646617	243	23	SNV
YHIM-02	Non-synergy	RSF1	chr11	77378255	77378255	T	A	exonic	nonsynonymous SNV	NM_016578	16	c.A4033T	p.S1345C		0.14418605	368	62	SNV
YHIM-02	Non-synergy	RTF1	chr15	41769455	41769455	C	T	exonic	synonymous SNV	NM_015138	13	c.C1653T	p.R551R		0.23312883	125	38	SNV
YHIM-02	Non-synergy	SAMD4A	chr14	55203869	55203869	C	T	exonic	synonymous SNV	NM_015589	3	c.C843T	p.P281P		0.07326007	253	20	SNV
YHIM-02	Non-synergy	SCMH1	chr1	41579089	41579089	-	T	exonic	frameshift insertion	NM_001172220	7	c.397_398insA	p.G133fs					Indel
YHIM-02	Non-synergy	SCRT2	chr20	644878	644878	G	A	exonic	nonsynonymous SNV	NM_033129	2	c.C361T	p.R121W		0.37837838	23	14	SNV
YHIM-02	Non-synergy	SETBP1	chr18	42530821	42530821	C	A	exonic	nonsynonymous SNV	NM_015559	4	c.C1516A	p.P506T	CCG	0.50980392	25	26	SNV
YHIM-02	Non-synergy	SETX	chr9	135211875	135211875	T	C	exonic	nonsynonymous SNV	NM_015046	6	c.A526G	p.R176G		0.21794872	122	34	SNV
YHIM-02	Non-synergy	SEZ6L	chr22	26693009	26693009	G	A	exonic	synonymous SNV	NM_001184773	4	c.G1125A	p.Q375Q		0.28225807	89	35	SNV
YHIM-02	Non-synergy	SFTPA1	chr10	81372071	81372071	C	T	exonic	nonsynonymous SNV	NM_001164647	3	c.C176T	p.P59L		0.11515152	292	38	SNV
YHIM-02	Non-synergy	SGSH	chr17	78184498	78184498	G	A	exonic	nonsynonymous SNV	NM_000199	8	c.C1262T	p.T421M		0.25462963	161	55	SNV
YHIM-02	Non-synergy	SH2D1A	chrX	123480600	123480600	C	T	exonic	synonymous SNV	NM_001114937	1	c.C108T	p.S36S		0.48648649	114	108	SNV
YHIM-02	Non-synergy	SH3RF2	chr5	145393421	145393421	C	T	exonic	nonsynonymous SNV	NM_152550	5	c.C856T	p.R286C		0.38604651	132	83	SNV
YHIM-02	Non-synergy	SH3TC2	chr5	148389940	148389940	C	A	exonic	nonsynonymous SNV	NM_024577	14	c.G3220T	p.A1074S		0.28888889	32	13	SNV
YHIM-02	Non-synergy	SLC5A7	chr2	108626706	108626706	G	C	exonic	nonsynonymous SNV	NM_001305006	8	c.G817C	p.V273L		0.20809249	137	36	SNV
YHIM-02	Non-synergy	SLC6A16	chr19	49814551	49814551	A	T	exonic	synonymous SNV	NM_014037	2	c.T54A	p.T18T		0.4321608	113	86	SNV
YHIM-02	Non-synergy	SLC6A18	chr5	1246174	1246174	C	T	exonic	nonsynonymous SNV	NM_182632	12	c.C1868T	p.P623L		0.0794702	139	12	SNV
YHIM-02	Non-synergy	SLC6A8	chrX	152959034	152959034	A	G	exonic	synonymous SNV	NM_001142805	7	c.A1104G	p.A368A		0.41176471	20	14	SNV
YHIM-02	Non-synergy	SLC9A4	chr2	103148959	103148959	T	A	exonic	nonsynonymous SNV	NM_001011552	12	c.T2209A	p.L737M		0.18974359	158	37	SNV
YHIM-02	Non-synergy	SLITRK4	chrX	142717933	142717933	G	T	exonic	nonsynonymous SNV	NM_001184749	2	c.C992A	p.S331Y		0.20833333	247	65	SNV
YHIM-02	Non-synergy	SMAD1	chr4	146461055	146461055	C	A	exonic	nonsynonymous SNV	NM_001003688	3	c.C500A	p.P167Q		0.3497537	132	71	SNV
YHIM-02	Non-synergy	SMAD7	chr18	46476198	46476198	G	A	exonic	synonymous SNV	NM_001190821	1	c.C597T	p.S199S		0.03367003	287	10	SNV
YHIM-02	Non-synergy	SMS	chrX	21958978	21958978	G	C	exonic	nonsynonymous SNV	NM_001258423	1	c.G36C	p.M12I		0.14814815	23	4	SNV
YHIM-02	Non-synergy	SRRD	chr22	26879947	26879967	GAGC	-	exonic	nonframeshift deletion	NM_001013694	1	c.91_111del	p.31_37del					Indel
YHIM-02	Non-synergy	SRRM4	chr12	119583454	119583454	A	G	exonic	nonsynonymous SNV	NM_194286	9	c.A1040G	p.E347G		0.13138686	119	18	SNV
YHIM-02	Non-synergy	STPG1	chr1	24687383	24687383	G	A	exonic	nonsynonymous SNV	NM_178122	7	c.C745T	p.R249W		0.35036496	89	48	SNV

YHIM-02	Non-synergy	STRBP	chr9	125921478	125921478	T	C	exonic	synonymous SNV	NM_001171137	9	c.A690G	p.E230E		0.04	120	5	SNV
YHIM-02	Non-synergy	SVIL	chr10	29752506	29752506	G	A	exonic	synonymous SNV	NM_003174	33	c.C4914T	p.I1638I		0.46875	68	60	SNV
YHIM-02	Non-synergy	SYAP1	chrX	16775308	16775308	C	G	exonic	nonsynonymous SNV	NM_032796	8	c.C807G	p.S269R		0.03177258	579	19	SNV
YHIM-02	Non-synergy	SYNE3	chr14	95899693	95899693	A	G	exonic	synonymous SNV	NM_152592	15	c.T2592C	p.R864R		0.24603175	285	93	SNV
YHIM-02	Non-synergy	TAS2R9	chr12	10962602	10962602	C	T	exonic	nonsynonymous SNV	NM_023917	1	c.G73A	p.G25R		0.13246269	465	71	SNV
YHIM-02	Non-synergy	TCF7L1	chr2	85510645	85510645	G	A	exonic	nonsynonymous SNV	NM_031283	4	c.G469A	p.D157N		0.21232877	115	31	SNV
YHIM-02	Non-synergy	TDG	chr12	104376608	104376608	G	A	exonic	synonymous SNV	NM_003211	5	c.G510A	p.E170E		0.08108108	34	3	SNV
YHIM-02	Non-synergy	TENM1	chrX	123514393	123514393	C	T	exonic	nonsynonymous SNV	NM_014253	31	c.G8171A	p.R2724K		0.27240143	203	76	SNV
YHIM-02	Non-synergy	THOC5	chr22	29908039	29908040	TC	-	exonic	frameshift deletion	NM_001002879	18	c.1767_1768del	p.E589fs					Indel
YHIM-02	Non-synergy	TKTL2	chr4	164394507	164394507	C	A	exonic	nonsynonymous SNV	NM_032136	1	c.G380T	p.G127V		0.30215827	97	42	SNV
YHIM-02	Non-synergy	TMCO1	chr1	165712414	165712414	G	A	exonic	nonsynonymous SNV	NM_001256164	6	c.C509T	p.S170L		0.24832215	112	37	SNV
YHIM-02	Non-synergy	TMEM151A	chr11	66062435	66062435	G	A	exonic	nonsynonymous SNV	NM_153266	2	c.G718A	p.A240T		0.09883721	620	68	SNV
YHIM-02	Non-synergy	TMEM238	chr19	55895330	55895330	C	T	exonic	nonsynonymous SNV	NM_001190764	1	c.G298A	p.E100K		0.08602151	170	16	SNV
YHIM-02	Non-synergy	TNIK	chr3	170858204	170858204	C	T	exonic	nonsynonymous SNV	NM_001161560	13	c.G1316A	p.R439H		0.27472528	66	25	SNV
YHIM-02	Non-synergy	TNK2	chr3	195609116	195609116	G	A	exonic	synonymous SNV	NM_001010938	6	c.C882T	p.R294R		0.24172185	229	73	SNV
YHIM-02	Non-synergy	TP53	chr17	7577539	7577539	G	A	exonic	nonsynonymous SNV	NM_001126115	3	c.C346T	p.R116W	CCG	1	0	132	SNV
YHIM-02	Non-synergy	TRDN	chr6	123869757	123869757	G	C	exonic	nonsynonymous SNV	NM_001251987	3	c.C233G	p.A78G		0.23913044	70	22	SNV
YHIM-02	Non-synergy	TRIP10	chr19	6749963	6749963	G	A	exonic	nonsynonymous SNV	NM_001288963	11	c.G1113A	p.M371I		0.40983607	36	25	SNV
YHIM-02	Non-synergy	TRO	chrX	54949215	54949215	C	A	exonic	nonsynonymous SNV	NM_001039705	3	c.C250A	p.P84T		0.0513834	240	13	SNV
YHIM-02	Non-synergy	TRO	chrX	54949217	54949217	T	C	exonic	synonymous SNV	NM_001039705	3	c.T252C	p.P84P		0.0515873	239	13	SNV
YHIM-02	Non-synergy	TRPM3	chr9	73233843	73233843	C	G	exonic	nonsynonymous SNV	NM_206944	15	c.G1773C	p.Q591H		0.07211539	193	15	SNV
YHIM-02	Non-synergy	TSNARE1	chr8	143413192	143413192	G	C	exonic	nonsynonymous SNV	NM_001291931	3	c.C89G	p.A30G		0.03883495	198	8	SNV
YHIM-02	Non-synergy	TUBGCP5	chr15	22845842	22845842	A	T	exonic	nonsynonymous SNV	NM_001102610	7	c.A703T	p.S235C		0.43617021	53	41	SNV
YHIM-02	Non-synergy	UBE3A	chr15	25653792	25653792	C	T	exonic	nonsynonymous SNV	NM_000462	4	c.G4A	p.E2K		0.5	50	50	SNV
YHIM-02	Non-synergy	UBQLN2	chrX	56591472	56591472	C	T	exonic	nonsynonymous SNV	NM_013444	1	c.C1166T	p.A389V		0.10299626	479	55	SNV
YHIM-02	Non-synergy	UNC5D	chr8	35542211	35542211	G	A	exonic	nonsynonymous SNV	NM_080872	6	c.G863A	p.G288E		0.55882353	30	38	SNV
YHIM-02	Non-synergy	UTRN	chr6	144747562	144747562	G	C	exonic	nonsynonymous SNV	NM_007124	6	c.G545C	p.G182A		0.32258065	63	30	SNV
YHIM-02	Non-synergy	VAV1	chr19	6853002	6853003	CT	-	exonic	frameshift deletion	NM_001258206	24	c.2178_2179del	p.N726fs					Indel
YHIM-02	Non-synergy	VRK1	chr14	97342371	97342371	G	A	exonic	nonsynonymous SNV	NM_003384	12	c.G1073A	p.R358Q		0.7195122	23	59	SNV
YHIM-02	Non-synergy	VWA9	chr15	65897469	65897469	G	T	exonic	nonsynonymous SNV	NM_001207059	2	c.C157A	p.Q53K		0.3206278	303	143	SNV
YHIM-02	Non-synergy	WDR27	chr6	170033111	170033111	C	T	exonic	nonsynonymous SNV	NM_001202550	18	c.G1774A	p.A592T		0.21052632	75	20	SNV
YHIM-02	Non-synergy	WTIP	chr19	34973447	34973447	G	A	exonic	nonsynonymous SNV	NM_001080436	1	c.G568A	p.G190S		0.35294118	11	6	SNV
YHIM-02	Non-synergy	XPO6	chr16	28137089	28137089	C	A	exonic	nonsynonymous SNV	NM_015171	13	c.G1687T	p.A563S		0.2392638	248	78	SNV
YHIM-02	Non-synergy	YPEL2	chr17	57474541	57474541	G	C	exonic	nonsynonymous SNV	NM_001005404	5	c.G350C	p.G117A		0.31636364	188	87	SNV
YHIM-02	Non-synergy	ZC2HC1A	chr8	79610745	79610745	C	T	exonic	nonsynonymous SNV	NM_016010	7	c.C701T	p.A234V		0.05737705	115	7	SNV
YHIM-02	Non-synergy	ZCCHC18	chrX	103359375	103359375	G	T	exonic	nonsynonymous SNV	NM_001143978	3	c.G573T	p.R191S		0.05434783	174	10	SNV
YHIM-02	Non-synergy	ZFHX4	chr8	77776199	77776199	G	C	exonic	nonsynonymous SNV	NM_024721	11	c.G10249C	p.A3417P		0.19111111	182	43	SNV
YHIM-02	Non-synergy	ZFP91	chr11	58384822	58384822	C	T	exonic	synonymous SNV	NM_001197051	11	c.C1353T	p.S451S		0.20634921	150	39	SNV
YHIM-02	Non-synergy	ZGRF1	chr4	113510976	113510976	A	T	exonic	nonsynonymous SNV	NM_018392	11	c.T3031A	p.L1011M		0.35046729	139	75	SNV
YHIM-02	Non-synergy	ZNF107	chr7	64166973	64166973	A	T	exonic	nonsynonymous SNV	NM_001282360	3	c.A402T	p.R134S		0.1300813	107	16	SNV
YHIM-02	Non-synergy	ZNF672	chr1	249141738	249141738	T	G	exonic	nonsynonymous SNV	NM_024836	4	c.T265G	p.L89V		0.06748466	304	22	SNV
YHIM-02	Non-synergy	ZNF750	chr17	80789076	80789076	C	T	exonic	nonsynonymous SNV	NM_024702	2	c.G1255A	p.D419N		0.26578073	221	80	SNV
YHIM-02	Non-synergy	AUP1	chr2	74755374	74755374	C	A	splicing	splicing	NM_181575	6	c.671+1G>T			0.22093023	67	19	SNV
YHIM-02	Non-synergy	GLCCI1	chr7	8099879	8099879	G	T	splicing	splicing	NM_138426	5	c.966+1G>T			0.05970149	126	8	SNV
YHIM-02	Non-synergy	KIAA0922	chr4	154517409	154517409	G	T	splicing	splicing	NM_015196	20	c.1993-1G>T			0.27225131	139	52	SNV
YHIM-02	Non-synergy	RASGRF1	chr15	79382564	79382564	C	T	splicing	splicing	NM_001145648	1	c.276+1G>A			0.15151515	28	5	SNV
YHIM-02	Non-synergy	TENM4	chr11	78399273	78399273	T	C	splicing	splicing	NM_001098816	29	c.5088-2A>G			0.17261905	139	29	SNV
YHIM-03	Non-synergy	ADCY5	chr3	123047587	123047587	A	G	exonic	nonsynonymous SNV	NM_001199642	6	c.T659C	p.V220A		0.64988558	153	284	SNV
YHIM-03	Non-synergy	ADCY8	chr8	132002709	132002709	C	T	exonic	nonsynonymous SNV	NM_001115	2	c.G1040A	p.R347H		0.40983607	36	25	SNV
YHIM-03	Non-synergy	ADGRF5	chr6	46856220	46856220	A	G	exonic	synonymous SNV	NM_001098518	4	c.T180C	p.A60A		0.47904192	87	80	SNV
YHIM-03	Non-synergy	ANKHD1,ANI	chr5	139819705	139819705	C	T	exonic	nonsynonymous SNV	NM_001197030	4	c.C619T	p.R207C		0.97058824	5	165	SNV
YHIM-03	Non-synergy	APBB3	chr5	139938354	139938354	C	T	exonic	nonsynonymous SNV	NM_006051	12	c.G1298A	p.G433D		0.53333333	28	32	SNV
YHIM-03	Non-synergy	ARHGAP6	chrX	11204518	11204518	C	A	exonic	nonsynonymous SNV	NM_001287242	5	c.G571T	p.D191Y		0.39516129	75	49	SNV
YHIM-03	Non-synergy	ATXN3	chr14	92537353	92537353	-	GCT	exonic	nonframeshift insertion	NM_001164782	2	c.68_69insAGC	p.G23delinsGA					Indel
YHIM-03	Non-synergy	BVES	chr6	105563626	105563626	C	A	exonic	nonsynonymous SNV	NM_001199563	7	c.G893T	p.S298I		0.30434783	64	28	SNV
YHIM-03	Non-synergy	C10orf90	chr10	128193152	128193152	C	T	exonic	nonsynonymous SNV	NM_001004298	3	c.G617A	p.R206Q		0.52354571	172	189	SNV
YHIM-03	Non-synergy	C6orf62	chr6	24718792	24718792	A	G	exonic	synonymous SNV	NM_030939	1	c.T105C	p.Y35Y		0.06422018	102	7	SNV
YHIM-03	Non-synergy	CABLES2	chr20	60967976	60967976	C	T	exonic	nonsynonymous SNV	NM_031215	7	c.G984A	p.M328I		0.27868853	176	68	SNV
YHIM-03	Non-synergy	CABLES2	chr20	60968638	60968638	C	T	exonic	synonymous SNV	NM_031215	6	c.G738A	p.L246L		0.28633406	329	132	SNV
YHIM-03	Non-synergy	CACNB1	chr17	37351150	37351150	C	T	exonic	nonsynonymous SNV	NM_000723	2	c.G158A	p.S53N		1	0	45	SNV
YHIM-03	Non-synergy	CARD6	chr5	40852830	40852830	T	C	exonic	nonsynonymous SNV	NM_032587	3	c.T1396C	p.S466P		0.23778502	234	73	SNV
YHIM-03	Non-synergy	CDHR5	chr11	621419	621419	G	A	exonic	nonsynonymous SNV	NM_001171968	6	c.C544T	p.R182C		0.44827586	80	65	SNV

YHIM-03	Non-synergy	CLNK	chr4	10492097	10492097	A	G	exonic	synonymous SNV	NM_052964	19	c.T1281C	p.P427P		0.28834356	116	47	SNV
YHIM-03	Non-synergy	DARS	chr2	136718971	136718971	A	T	exonic	synonymous SNV	NM_001293312	3	c.T15A	p.A5A		0.03448276	196	7	SNV
YHIM-03	Non-synergy	DDX17	chr22	38894131	38894131	T	C	exonic	nonsynonymous SNV	NM_001098504	5	c.A697G	p.I233V		0.70247934	36	85	SNV
YHIM-03	Non-synergy	DNAH2	chr17	7678616	7678616	T	C	exonic	nonsynonymous SNV	NM_020877	30	c.T4777C	p.Y1593H		1	0	62	SNV
YHIM-03	Non-synergy	DNAH8	chr6	38690687	38690687	G	A	exonic	synonymous SNV	NM_001206927	2	c.G102A	p.P34P		0.3956044	55	36	SNV
YHIM-03	Non-synergy	DSPP	chr4	88537224	88537232	ACAG	-	exonic	nonframeshift deletion	NM_014208	5	c.3410_3418del	p.1137_1140del					Indel
YHIM-03	Non-synergy	ELF3	chr1	201982164	201982164	G	A	exonic	nonsynonymous SNV	NM_001114309	6	c.G688A	p.D230N		0.28742515	238	96	SNV
YHIM-03	Non-synergy	FAM65C	chr20	49219014	49219014	C	T	exonic	synonymous SNV	NM_001290268	13	c.G1254A	p.T418T		0.67829457	83	175	SNV
YHIM-03	Non-synergy	FAR1	chr11	13736138	13736138	T	C	exonic	synonymous SNV	NM_032228	9	c.T1038C	p.H346H		0.06557377	57	4	SNV
YHIM-03	Non-synergy	FGFRL1	chr4	1018268	1018268	C	A	exonic	synonymous SNV	NM_021923	5	c.C888A	p.I296I		0.65567766	188	358	SNV
YHIM-03	Non-synergy	FOXH1	chr8	145700618	145700618	G	A	exonic	synonymous SNV	NM_003923	2	c.C201T	p.F67F		0.30397727	245	107	SNV
YHIM-03	Non-synergy	GALNT9	chr12	132824545	132824545	T	C	exonic	nonsynonymous SNV	NM_001122636	6	c.A995G	p.D332G		0.43312102	89	68	SNV
YHIM-03	Non-synergy	GFRA3	chr5	137593444	137593444	G	A	exonic	synonymous SNV	NM_001496	4	c.C669T	p.N223N		0.97222222	4	140	SNV
YHIM-03	Non-synergy	GMPS	chr3	155621717	155621717	C	T	exonic	synonymous SNV	NM_003875	4	c.C390T	p.N130N	CCG	0.64210526	34	61	SNV
YHIM-03	Non-synergy	HDLBP	chr2	242194571	242194571	G	A	exonic	synonymous SNV	NM_001243900	9	c.C984T	p.A328A		0.52222389	54	59	SNV
YHIM-03	Non-synergy	HIST1H2AH	chr6	27115097	27115097	C	A	exonic	nonsynonymous SNV	NM_080596	1	c.C190A	p.L64M		0.38181818	68	42	SNV
YHIM-03	Non-synergy	HOOK2	chr19	12882233	12882233	C	T	exonic	nonsynonymous SNV	NM_001100176	8	c.G578A	p.R193H		0.52272727	84	92	SNV
YHIM-03	Non-synergy	HS3ST4	chr16	26147447	26147447	C	G	exonic	nonsynonymous SNV	NM_006040	2	c.C1249G	p.H417D		0.44907407	119	97	SNV
YHIM-03	Non-synergy	IL1RAPL2	chrX	104984587	104984587	C	T	exonic	synonymous SNV	NM_017416	8	c.C951T	p.I317I		0.0875	73	7	SNV
YHIM-03	Non-synergy	IQGAP1	chr15	90934100	90934100	G	A	exonic	synonymous SNV	NM_003870	2	c.G150A	p.A50A		0.59649123	92	136	SNV
YHIM-03	Non-synergy	KANK1	chr9	744589	744589	G	A	exonic	synonymous SNV	NM_153186	10	c.G3522A	p.P1174P		0.48765432	83	79	SNV
YHIM-03	Non-synergy	KCNA4	chr11	30032585	30032585	C	T	exonic	synonymous SNV	NM_002233	2	c.G1641A	p.A547A		0.99333333	1	149	SNV
YHIM-03	Non-synergy	KCNMA1	chr10	79396180	79396180	G	C	exonic	nonsynonymous SNV	NM_001271522	2	c.C472G	p.L158V		0.43373494	94	72	SNV
YHIM-03	Non-synergy	KMT2C	chr7	151945285	151945285	A	G	exonic	nonsynonymous SNV	NM_170606	14	c.T2234C	p.I745T	CCG	0.22839506	250	74	SNV
YHIM-03	Non-synergy	KRT1	chr12	53073801	53073815	CCAC	-	exonic	nonframeshift deletion	NM_006121	1	c.318_332del	p.106_111del					Indel
YHIM-03	Non-synergy	LRCH1	chr13	47262104	47262104	G	A	exonic	nonsynonymous SNV	NM_001164211	6	c.G940A	p.V314M		0.31182796	128	58	SNV
YHIM-03	Non-synergy	LRPAP1	chr4	3517857	3517857	G	T	exonic	nonsynonymous SNV	NM_002337	6	c.C820A	p.L274M		0.29394813	245	102	SNV
YHIM-03	Non-synergy	MATR3	chr5	138643914	138643914	A	G	exonic	synonymous SNV	NM_001194955	2	c.A810G	p.R270R		0.16428571	117	23	SNV
YHIM-03	Non-synergy	MDGA2	chr14	47770689	47770689	G	A	exonic	synonymous SNV	NM_001113498	2	c.C138T	p.S46S		0.74342105	39	113	SNV
YHIM-03	Non-synergy	MFSD5	chr12	53647522	53647522	C	T	exonic	synonymous SNV	NM_001170790	2	c.C1224T	p.I408I		0.45	154	126	SNV
YHIM-03	Non-synergy	MUC2	chr11	1093204	1093204	C	A	exonic	nonsynonymous SNV	NM_002457	30	c.C5023A	p.P1675T		0.08474576	54	5	SNV
YHIM-03	Non-synergy	MYH11	chr16	15835424	15835424	C	A	exonic	stopgain	NM_002474	22	c.G2755T	p.E919X	CCG	0.375	50	30	SNV
YHIM-03	Non-synergy	MYO7B	chr2	128351194	128351194	C	T	exonic	nonsynonymous SNV	NM_001080527	18	c.C2219T	p.A740V		0.46486487	99	86	SNV
YHIM-03	Non-synergy	NUMA1	chr11	71730641	71730641	A	G	exonic	synonymous SNV	NM_006185	9	c.T483C	p.S161S	CCG	0.85903084	32	195	SNV
YHIM-03	Non-synergy	NUP188	chr9	131768593	131768593	G	A	exonic	synonymous SNV	NM_015354	43	c.G5019A	p.P1673P		0.39388145	317	206	SNV
YHIM-03	Non-synergy	PLXNA2	chr1	208390827	208390827	G	A	exonic	synonymous SNV	NM_025179	2	c.C441T	p.I147I		0.34878049	267	143	SNV
YHIM-03	Non-synergy	POU6F2	chr7	39125590	39125590	T	C	exonic	nonsynonymous SNV	NM_001166018	3	c.T149C	p.V50A		0.04878049	234	12	SNV
YHIM-03	Non-synergy	POU6F2	chr7	39125588	39125588	T	C	exonic	synonymous SNV	NM_001166018	3	c.T147C	p.P49P		0.04032258	238	10	SNV
YHIM-03	Non-synergy	PTCHD2	chr1	11561388	11561388	C	T	exonic	synonymous SNV	NM_020780	2	c.C339T	p.H113H		0.33863636	291	149	SNV
YHIM-03	Non-synergy	QSOX2	chr9	139137426	139137426	G	A	exonic	nonsynonymous SNV	NM_181701	1	c.C224T	p.T75I		0.29385965	161	67	SNV
YHIM-03	Non-synergy	RBCK1	chr20	400259	400259	G	C	exonic	nonsynonymous SNV	NM_006462	5	c.G514C	p.E172Q		0.95789474	4	91	SNV
YHIM-03	Non-synergy	RHPN2	chr19	33517507	33517507	C	T	exonic	nonsynonymous SNV	NM_033103	3	c.G217A	p.V73M		0.05617978	84	5	SNV
YHIM-03	Non-synergy	RPS15	chr19	1440068	1440068	G	C	exonic	nonsynonymous SNV	NM_001018	3	c.G140C	p.R47P		0.13043478	20	3	SNV
YHIM-03	Non-synergy	RPS5	chr19	58904475	58904475	C	T	exonic	nonsynonymous SNV	NM_001009	3	c.C241T	p.R81C		0.56183746	124	159	SNV
YHIM-03	Non-synergy	SAMD4A	chr14	55203869	55203869	C	T	exonic	synonymous SNV	NM_015589	3	c.C843T	p.P281P		0.04232804	362	16	SNV
YHIM-03	Non-synergy	SCAI	chr9	127905704	127905704	C	T	exonic	nonsynonymous SNV	NM_001144877	1	c.G13A	p.A5T		0.47368421	10	9	SNV
YHIM-03	Non-synergy	SEMA4D	chr9	92017816	92017816	G	A	exonic	synonymous SNV	NM_001142287	6	c.C222T	p.N74N		0.31785714	191	89	SNV
YHIM-03	Non-synergy	SLC44A2	chr19	10742189	10742189	T	A	exonic	synonymous SNV	NM_001145056	7	c.T474A	p.A158A		0.38690476	103	65	SNV
YHIM-03	Non-synergy	SORCS1	chr10	108447990	108447990	G	A	exonic	nonsynonymous SNV	NM_001013031	10	c.C1520T	p.P507L		0.48543689	53	50	SNV
YHIM-03	Non-synergy	SSC4D	chr7	76029869	76029869	C	T	exonic	nonsynonymous SNV	NM_080744	4	c.G209A	p.R70H		0.38596491	70	44	SNV
YHIM-03	Non-synergy	STOX2	chr4	184932032	184932032	G	A	exonic	nonsynonymous SNV	NM_020225	3	c.G2041A	p.V681I		0.53773585	98	114	SNV
YHIM-03	Non-synergy	TADA2B	chr4	7045393	7045393	C	T	exonic	synonymous SNV	NM_152293	1	c.C87T	p.I29I		0.33640553	144	73	SNV
YHIM-03	Non-synergy	TAF4	chr20	60640306	60640311	GCCA	-	exonic	nonframeshift deletion	NM_003185	1	c.556_561del	p.186_187del					Indel
YHIM-03	Non-synergy	TECR	chr19	14676573	14676573	G	C	exonic	nonsynonymous SNV	NM_138501	13	c.G817C	p.V273L		0.41333333	88	62	SNV
YHIM-03	Non-synergy	TGFBR2	chr3	30713774	30713774	C	-	exonic	frameshift deletion	NM_003242	4	c.1099delC	p.P367fs	CCG				Indel
YHIM-03	Non-synergy	TMEM72	chr10	45430287	45430287	G	T	exonic	nonsynonymous SNV	NM_001123376	5	c.G533T	p.G178V		0.03333333	174	6	SNV
YHIM-03	Non-synergy	TMPRSS13	chr11	117789342	117789342	T	C	exonic	nonsynonymous SNV	NM_001077263	2	c.A233G	p.Q78R		0.54901961	23	28	SNV
YHIM-03	Non-synergy	TMPRSS13	chr11	117789345	117789345	G	C	exonic	nonsynonymous SNV	NM_001077263	2	c.C230G	p.A77G		0.48	26	24	SNV
YHIM-03	Non-synergy	TOMM70A	chr3	100096715	100096715	T	C	exonic	nonsynonymous SNV	NM_014820	6	c.A926G	p.N309S		0.68181818	28	60	SNV
YHIM-03	Non-synergy	TONSL	chr8	145665485	145665485	C	T	exonic	nonsynonymous SNV	NM_013432	11	c.G1399A	p.E467K		0.25405405	138	47	SNV
YHIM-03	Non-synergy	TP53	chr17	7578461	7578461	C	A	exonic	nonsynonymous SNV	NM_001126115	1	c.G73T	p.V25F	CCG	1	0	195	SNV
YHIM-03	Non-synergy	TUBA3C	chr13	19748035	19748035	C	G	exonic	nonsynonymous SNV	NM_006001	5	c.G1321C	p.E441Q		0.69512195	50	114	SNV

YHIM-03	Non-synergy	UROC1	chr3	126224559	126224559	G	A	exonic	synonymous SNV	NM_001165974	8	c.C798T	p.I266I		0.29770992	368	156	SNV
YHIM-03	Non-synergy	ZC3H12A	chr1	37941525	37941525	G	A	exonic	nonsynonymous SNV	NM_025079	2	c.G428A	p.S143N		0.32577904	238	115	SNV
YHIM-03	Non-synergy	ZFP62	chr5	180275916	180275916	C	A	exonic	nonsynonymous SNV	NM_001172638	2	c.G2579T	p.R860I		0.06470588	159	11	SNV
YHIM-03	Non-synergy	ZNF131	chr5	43161813	43161813	G	A	exonic	synonymous SNV	NM_001297548	5	c.G834A	p.E278E		0.05521472	308	18	SNV
YHIM-03	Non-synergy	ZNF566	chr19	36940278	36940278	T	C	exonic	synonymous SNV	NM_001300970	4	c.A546G	p.K182K		0.50704225	35	36	SNV
YHIM-03	Non-synergy	ZNF608	chr5	123984804	123984804	C	T	exonic	nonsynonymous SNV	NM_020747	4	c.G1273A	p.D425N		0.04285714	134	6	SNV
YHIM-03	Non-synergy	CABLES2	chr20	60966304	60966304	C	T	splicing	splicing	NM_031215	9	c.1296+1G>A			0.2745098	111	42	SNV
YHIM-03	Non-synergy	SLC3A2	chr11	62638311	62638313	TAA	-	splicing	splicing									Indel
YHIM-03	Non-synergy	STUB1	chr16	731151	731151	G	A	splicing	splicing	NM_005861	2	c.160-1G>A			0.58510638	39	55	SNV
YHIM-04	Non-synergy	ABCB9	chr12	123414541	123414541	G	A	exonic	nonsynonymous SNV	NM_001243013	11	c.C2029T	p.R677W		0.54901961	69	84	SNV
YHIM-04	Non-synergy	ABCC1	chr16	16138457	16138457	C	A	exonic	synonymous SNV	NM_004996	8	c.C960A	p.T320T		0.1037037	121	14	SNV
YHIM-04	Non-synergy	ABCD2	chr12	40012533	40012533	C	G	exonic	synonymous SNV	NM_005164	1	c.G885C	p.V295V		0.49386503	165	161	SNV
YHIM-04	Non-synergy	ACLY	chr17	40048603	40048603	C	G	exonic	nonsynonymous SNV	NM_001303275	15	c.G1831C	p.D611H		0.24590164	92	30	SNV
YHIM-04	Non-synergy	ACLY	chr17	40048630	40048630	C	T	exonic	nonsynonymous SNV	NM_001303275	15	c.G1804A	p.D602N		0.24427481	99	32	SNV
YHIM-04	Non-synergy	ACTL9	chr19	8808056	8808056	C	T	exonic	synonymous SNV	NM_178525	1	c.G996A	p.A332A		0.48039216	212	196	SNV
YHIM-04	Non-synergy	ADAMTS1	chr21	28210800	28210800	C	T	exonic	nonsynonymous SNV	NM_006988	8	c.G2162A	p.G721E		0.46623794	166	145	SNV
YHIM-04	Non-synergy	ADAR	chr1	154574821	154574821	G	A	exonic	synonymous SNV	NM_001111	2	c.C297T	p.L99L		0.47535211	149	135	SNV
YHIM-04	Non-synergy	AGO3	chr1	36505518	36505518	C	G	exonic	stopgain	NM_177422	13	c.C1268G	p.S423X		1	0	79	SNV
YHIM-04	Non-synergy	ALDH9A1	chr1	165649823	165649823	G	A	exonic	synonymous SNV	NM_000696	5	c.C690T	p.F230F		0.49166667	122	118	SNV
YHIM-04	Non-synergy	AMBN	chr4	71472365	71472365	A	T	exonic	nonsynonymous SNV	NM_016519	13	c.A1262T	p.N421I		0.10273973	131	15	SNV
YHIM-04	Non-synergy	AMIGO3	chr3	49756422	49756422	G	A	exonic	synonymous SNV	NM_198722	1	c.C477T	p.L159L		1	0	354	SNV
YHIM-04	Non-synergy	ANAPC2	chr9	140069435	140069435	A	G	exonic	nonsynonymous SNV	NM_013366	13	c.T2428C	p.Y810H		0.48275862	45	42	SNV
YHIM-04	Non-synergy	ANKRD11	chr16	89348873	89348873	C	G	exonic	nonsynonymous SNV	NM_001256183	9	c.G4077C	p.K1359N		0.07692308	96	8	SNV
YHIM-04	Non-synergy	ANKRD26	chr10	27337812	27337812	C	G	exonic	nonsynonymous SNV	NM_001256053	17	c.G1732C	p.D578H		0.3836478	98	61	SNV
YHIM-04	Non-synergy	ANKRD30B	chr18	14787040	14787040	C	G	exonic	nonsynonymous SNV	NM_001145029	15	c.C1675G	p.Q559E		0.47692308	34	31	SNV
YHIM-04	Non-synergy	ANKS1A	chr6	34857321	34857321	-	GCA	exonic	nonframeshift insertion	NM_015245	1	c.142_143insGC	p.G48delinsGS					Indel
YHIM-04	Non-synergy	AP2B1	chr17	33963429	33963429	G	A	exonic	nonsynonymous SNV	NM_001030006	10	c.G1225A	p.E409K		0.42857143	80	60	SNV
YHIM-04	Non-synergy	AP4E1	chr15	51289601	51289601	A	C	exonic	nonsynonymous SNV	NM_001252127	18	c.A2200C	p.S734R		0.49640288	70	69	SNV
YHIM-04	Non-synergy	APBA2	chr15	29409336	29409336	G	C	exonic	stoploss	NM_001130414	13	c.G2214C	p.X738Y		0.54961832	59	72	SNV
YHIM-04	Non-synergy	ARFGEF3	chr6	138528220	138528220	A	G	exonic	nonsynonymous SNV	NM_020340	3	c.A179G	p.K60R		0.44255319	131	104	SNV
YHIM-04	Non-synergy	ARHGEF18	chr19	7523507	7523507	A	G	exonic	nonsynonymous SNV	NM_001130955	9	c.A1727G	p.Y576C		0.53125	45	51	SNV
YHIM-04	Non-synergy	ARID5B	chr10	63852728	63852728	A	T	exonic	nonsynonymous SNV	NM_001244638	7	c.A2777T	p.N926I		0.33105023	293	145	SNV
YHIM-04	Non-synergy	ARX	chrX	25031117	25031117	C	T	exonic	nonsynonymous SNV	NM_139058	2	c.G995A	p.R332H		0.08035714	103	9	SNV
YHIM-04	Non-synergy	ASH1L	chr1	155491223	155491223	T	C	exonic	nonsynonymous SNV	NM_018489	2	c.A88G	p.T30A		0.50696379	177	182	SNV
YHIM-04	Non-synergy	ATP2B3	chrX	152813339	152813339	G	C	exonic	nonsynonymous SNV	NM_001001344	7	c.G1005C	p.K335N	CCG	0.47169811	56	50	SNV
YHIM-04	Non-synergy	ATP7B	chr13	52544694	52544694	G	A	exonic	stopgain	NM_000053	3	c.C1477T	p.Q493X		1	0	157	SNV
YHIM-04	Non-synergy	BAP1	chr3	52442604	52442604	G	C	exonic	nonsynonymous SNV	NM_004656	4	c.C141G	p.I47M	CCG	1	0	46	SNV
YHIM-04	Non-synergy	BMS1	chr10	43291928	43291928	G	A	exonic	nonsynonymous SNV	NM_014753	10	c.G1236A	p.M412I		0.46710526	81	71	SNV
YHIM-04	Non-synergy	BPIFB3	chr20	31652552	31652552	C	A	exonic	nonsynonymous SNV	NM_182658	8	c.C825A	p.D275E		0.29956897	325	139	SNV
YHIM-04	Non-synergy	BTG2	chr1	203276548	203276548	G	C	exonic	synonymous SNV	NM_006763	2	c.G459C	p.V153V		0.41666667	91	65	SNV
YHIM-04	Non-synergy	C11orf80	chr11	66512322	66512322	T	G	exonic	nonsynonymous SNV	NM_024650	1	c.T109G	p.Y37D		0.03314917	175	6	SNV
YHIM-04	Non-synergy	C15orf40	chr15	83677340	83677340	T	G	exonic	nonsynonymous SNV	NM_001160113	3	c.A326C	p.K109T		0.03942652	268	11	SNV
YHIM-04	Non-synergy	C21orf62	chr21	34166168	34166168	C	T	exonic	nonsynonymous SNV	NM_001162496	2	c.G565A	p.V189I		0.47302905	127	114	SNV
YHIM-04	Non-synergy	C2CD3	chr11	73803498	73803498	C	G	exonic	nonsynonymous SNV	NM_001286577	19	c.G3480C	p.R1160S		0.53508772	53	61	SNV
YHIM-04	Non-synergy	C2orf71	chr2	29293679	29293679	G	C	exonic	nonsynonymous SNV	NM_001029883	1	c.C3449G	p.P1150R		0.46794872	166	146	SNV
YHIM-04	Non-synergy	C2orf72	chr2	231911713	231911713	G	A	exonic	nonsynonymous SNV	NM_001144994	3	c.G865A	p.E289K		0.52486188	172	190	SNV
YHIM-04	Non-synergy	C6orf203	chr6	107372338	107372338	C	G	exonic	synonymous SNV	NM_001142468	4	c.C621G	p.L207L		0.46666667	144	126	SNV
YHIM-04	Non-synergy	CABLES2	chr20	60968546	60968546	G	C	exonic	stopgain	NM_031215	6	c.C830G	p.S277X		1	0	110	SNV
YHIM-04	Non-synergy	CARD11	chr7	2979402	2979402	T	G	exonic	nonsynonymous SNV	NM_032415	6	c.A845C	p.E282A	CCG	0.49758454	104	103	SNV
YHIM-04	Non-synergy	CCDC102B	chr18	66721306	66721306	T	C	exonic	nonsynonymous SNV	NM_024781	8	c.T1474C	p.S492P		0.37711864	147	89	SNV
YHIM-04	Non-synergy	CCL23	chr17	34341416	34341416	T	G	exonic	nonsynonymous SNV	NM_005064	2	c.A94C	p.M32L		0.04230769	249	11	SNV
YHIM-04	Non-synergy	CCT7	chr2	73478516	73478516	G	T	exonic	nonsynonymous SNV	NM_001009570	6	c.G754T	p.D252Y		0.59375	26	38	SNV
YHIM-04	Non-synergy	CFAP65	chr2	219894278	219894278	G	A	exonic	synonymous SNV	NM_001278296	10	c.C1302T	p.F434F		0.48270181	314	293	SNV
YHIM-04	Non-synergy	CGREF1	chr2	27325437	27325437	G	A	exonic	stopgain	NM_001166239	3	c.C103T	p.Q35X		1	0	56	SNV
YHIM-04	Non-synergy	CLCN1	chr7	143016910	143016910	G	C	exonic	nonsynonymous SNV	NM_000083	2	c.G243C	p.K81N		0.4566474	94	79	SNV
YHIM-04	Non-synergy	CLEC12B	chr12	10167300	10167300	G	C	exonic	nonsynonymous SNV	NM_001129998	3	c.G369C	p.M123I		0.36614173	161	93	SNV
YHIM-04	Non-synergy	CMYA5	chr5	79027658	79027658	G	C	exonic	nonsynonymous SNV	NM_153610	2	c.G3070C	p.D1024H		1	0	192	SNV
YHIM-04	Non-synergy	COL27A1	chr9	116930832	116930832	C	G	exonic	nonsynonymous SNV	NM_032888	3	c.C997G	p.L333V		0.47222222	114	102	SNV
YHIM-04	Non-synergy	COL7A1	chr3	48610988	48610988	A	C	exonic	synonymous SNV	NM_000094	82	c.T6576G	p.G2192G		1	0	90	SNV
YHIM-04	Non-synergy	CRB2	chr9	126133476	126133476	G	C	exonic	nonsynonymous SNV	NM_173689	8	c.G2055C	p.L685F		0.51104972	177	185	SNV
YHIM-04	Non-synergy	CRNKL1	chr20	20017975	20017975	C	T	exonic	nonsynonymous SNV	NM_001278628	13	c.G1888A	p.D630N		0.48543689	159	150	SNV
YHIM-04	Non-synergy	CSAD	chr12	53552385	53552385	G	T	exonic	nonsynonymous SNV	NM_001244706	8	c.C693A	p.F231L		0.48324022	185	173	SNV

YHIM-04	Non-synergy	CSPG4	chr15	75982329	75982329	A	T	exonic	nonsynonymous SNV	NM_001897	3	c.T1077A	p.S359R		0.05555556	68	4	SNV
YHIM-04	Non-synergy	CYP17A1	chr10	104591358	104591358	A	T	exonic	nonsynonymous SNV	NM_000102	7	c.T1150A	p.F384I		0.43137255	145	110	SNV
YHIM-04	Non-synergy	CYP2B6	chr19	41518206	41518206	G	C	exonic	nonsynonymous SNV	NM_000767	7	c.G968C	p.R323T		0.28169014	102	40	SNV
YHIM-04	Non-synergy	DBN1	chr5	176893985	176893985	C	G	exonic	nonsynonymous SNV	NM_004395	7	c.G634C	p.E212Q		0.06081081	139	9	SNV
YHIM-04	Non-synergy	DCAF6	chr1	168037695	168037695	C	T	exonic	stopgain	NM_001017977	18	c.C2512T	p.R838X		0.50326797	76	77	SNV
YHIM-04	Non-synergy	DDI1	chr11	103907672	103907672	C	G	exonic	nonsynonymous SNV	NM_001001711	1	c.C122G	p.P41R		1	0	219	SNV
YHIM-04	Non-synergy	DIAPH3	chr13	60554943	60554943	C	G	exonic	nonsynonymous SNV	NM_001258370	8	c.G742C	p.E248Q		0.21212121	104	28	SNV
YHIM-04	Non-synergy	DIP2B	chr12	51138487	51138487	C	G	exonic	synonymous SNV	NM_173602	38	c.C4596G	p.L1532L		0.16716418	279	56	SNV
YHIM-04	Non-synergy	DIRC2	chr3	122591302	122591302	G	C	exonic	nonsynonymous SNV	NM_032839	8	c.G1179C	p.L393F		0.45844875	391	331	SNV
YHIM-04	Non-synergy	DLG2	chr11	83676456	83676456	T	C	exonic	nonsynonymous SNV	NM_001142700	6	c.A644G	p.K215R		0.58119658	49	68	SNV
YHIM-04	Non-synergy	DNAH17	chr17	76521172	76521172	G	A	exonic	synonymous SNV	NM_173628	25	c.C3783T	p.V1261V		0.39106145	218	140	SNV
YHIM-04	Non-synergy	DNAI1	chr9	34506717	34506717	G	A	exonic	nonsynonymous SNV	NM_001281428	13	c.G1168A	p.V390I		0.48129676	208	193	SNV
YHIM-04	Non-synergy	DNASE1L1	chrX	153631445	153631445	T	A	exonic	synonymous SNV	NM_001009934	7	c.A612T	p.P204P		0.4631579	204	176	SNV
YHIM-04	Non-synergy	DOCK11	chrX	117805039	117805039	A	T	exonic	nonsynonymous SNV	NM_144658	46	c.A5130T	p.E1710D		0.29457364	91	38	SNV
YHIM-04	Non-synergy	DRGX	chr10	50599241	50599241	C	T	exonic	nonsynonymous SNV	NM_001276451	3	c.G101A	p.R34H		0.03215434	301	10	SNV
YHIM-04	Non-synergy	DSCAML1	chr11	117351900	117351900	A	T	exonic	nonsynonymous SNV	NM_020693	13	c.T2825A	p.L942H		1	0	98	SNV
YHIM-04	Non-synergy	EIF4B	chr12	53428493	53428493	A	T	exonic	nonsynonymous SNV	NM_001300821	10	c.A1306T	p.S436C		0.45519713	152	127	SNV
YHIM-04	Non-synergy	EIF4H	chr7	73588714	73588714	A	G	exonic	nonsynonymous SNV	NM_022170	1	c.A1G	p.M1V		0.39037433	114	73	SNV
YHIM-04	Non-synergy	ERBB3	chr12	56495327	56495327	C	T	exonic	nonsynonymous SNV	NM_001982	28	c.C3517T	p.R1173W	CCG	0.48888889	46	44	SNV
YHIM-04	Non-synergy	ERC1	chr12	1219500	1219500	T	G	exonic	nonsynonymous SNV	NM_001301248	4	c.T1304G	p.F435C	CCG	0.44444444	135	108	SNV
YHIM-04	Non-synergy	ERCC6	chr10	50668459	50668459	A	T	exonic	nonsynonymous SNV	NM_000124	20	c.T4022A	p.V1341E		0.45985402	74	63	SNV
YHIM-04	Non-synergy	ESRRA	chr11	64074676	64074676	G	C	exonic	nonsynonymous SNV	NM_001282450	2	c.G25C	p.E9Q		0.60638298	37	57	SNV
YHIM-04	Non-synergy	ESYT3	chr3	138186414	138186414	G	A	exonic	nonsynonymous SNV	NM_031913	11	c.G1181A	p.C394Y		0.495	202	198	SNV
YHIM-04	Non-synergy	EXTL1	chr1	26360306	26360306	C	T	exonic	synonymous SNV	NM_004455	9	c.C1638T	p.N546N		0.05714286	132	8	SNV
YHIM-04	Non-synergy	EYA4	chr6	133789800	133789800	G	A	exonic	nonsynonymous SNV	NM_001301012	9	c.G739A	p.D247N		0.49535604	163	160	SNV
YHIM-04	Non-synergy	FABP4	chr8	82391677	82391677	C	G	exonic	nonsynonymous SNV	NM_001442	3	c.G320C	p.R107T		0.49819495	139	138	SNV
YHIM-04	Non-synergy	FAM13B	chr5	137289864	137289864	G	C	exonic	nonsynonymous SNV	NM_001101800	14	c.C1643G	p.S548C		0.5890411	30	43	SNV
YHIM-04	Non-synergy	FAM179B	chr14	45431778	45431778	G	-	exonic	frameshift deletion	NM_001308120	1	c.154delG	p.G52fs					Indel
YHIM-04	Non-synergy	FAM213B	chr1	2518628	2518628	C	A	exonic	synonymous SNV	NM_001195736	2	c.C252A	p.L84L		1	0	62	SNV
YHIM-04	Non-synergy	FAM98B	chr15	38776807	38776815	GGTG	-	exonic	nonframeshift deletion	NM_173611	8	c.1249_1257del	p.417_419del					Indel
YHIM-04	Non-synergy	FAN1	chr15	31217463	31217463	T	G	exonic	nonsynonymous SNV	NM_014967	9	c.T2306G	p.L769R		0.45833333	143	121	SNV
YHIM-04	Non-synergy	FBXO15	chr18	71814931	71814931	C	A	exonic	synonymous SNV	NM_001142958	1	c.G90T	p.R30R		0.37931035	36	22	SNV
YHIM-04	Non-synergy	FCRL5	chr1	157514314	157514314	C	G	exonic	synonymous SNV	NM_001195388	5	c.G582C	p.L194L		0.5443038	36	43	SNV
YHIM-04	Non-synergy	FIBCD1	chr9	133805230	133805230	G	T	exonic	synonymous SNV	NM_032843	2	c.C276A	p.I92I		0.37345679	203	121	SNV
YHIM-04	Non-synergy	FLNC	chr7	128487832	128487832	T	-	exonic	frameshift deletion	NM_001127487	25	c.4370delT	p.V1457fs					Indel
YHIM-04	Non-synergy	FLRT3	chr20	14306598	14306598	T	C	exonic	nonsynonymous SNV	NM_013281	2	c.A1555G	p.K519E		0.48178138	256	238	SNV
YHIM-04	Non-synergy	FNDC1	chr6	159650894	159650894	T	G	exonic	nonsynonymous SNV	NM_032532	10	c.T1228G	p.S410A		0.25675676	165	57	SNV
YHIM-04	Non-synergy	FPR2	chr19	52272366	52272366	T	C	exonic	nonsynonymous SNV	NM_001005738	2	c.T455C	p.L152P		0.99418605	1	171	SNV
YHIM-04	Non-synergy	FRG1	chr4	190878612	190878612	C	T	exonic	synonymous SNV	NM_004477	6	c.C492T	p.D164D		0.05144033	461	25	SNV
YHIM-04	Non-synergy	FSTL1	chr3	120130785	120130785	T	A	exonic	nonsynonymous SNV	NM_007085	4	c.A214T	p.T72S		0.19799499	320	79	SNV
YHIM-04	Non-synergy	G6PD	chrX	153761284	153761284	G	A	exonic	synonymous SNV	NM_000402	9	c.C1014T	p.Y338Y		0.48525469	192	181	SNV
YHIM-04	Non-synergy	GALNT9	chr12	132834269	132834269	C	T	exonic	synonymous SNV	NM_001122636	5	c.G918A	p.P306P		0.51058201	185	193	SNV
YHIM-04	Non-synergy	GAREM1	chr18	29867548	29867548	C	T	exonic	nonsynonymous SNV	NM_001242409	4	c.G1012A	p.E338K		0.47938144	202	186	SNV
YHIM-04	Non-synergy	GCC2	chr2	109086698	109086698	G	C	exonic	nonsynonymous SNV	NM_181453	6	c.G913C	p.A305P		0.44660194	57	46	SNV
YHIM-04	Non-synergy	GPC3	chrX	132826424	132826424	C	G	exonic	nonsynonymous SNV	NM_001164619	4	c.G1103C	p.C368S	CCG	0.47104247	137	122	SNV
YHIM-04	Non-synergy	GPS1	chr17	80014959	80014959	C	G	exonic	nonsynonymous SNV	NM_004127	13	c.C1432G	p.L478V		0.22827939	453	134	SNV
YHIM-04	Non-synergy	GRIP1	chr12	66990637	66990637	C	T	exonic	synonymous SNV	NM_001178074	2	c.G126A	p.Q42Q		0.50549451	90	92	SNV
YHIM-04	Non-synergy	GRK7	chr3	141497222	141497222	G	A	exonic	synonymous SNV	NM_139209	1	c.G96A	p.R32R		0.27568922	289	110	SNV
YHIM-04	Non-synergy	HAPLN4	chr19	19368732	19368732	C	T	exonic	nonsynonymous SNV	NM_023002	5	c.G1103A	p.G368E		0.41747573	60	43	SNV
YHIM-04	Non-synergy	HAPLN4	chr19	19368816	19368816	C	G	exonic	nonsynonymous SNV	NM_023002	5	c.G1019C	p.G340A		0.46496815	84	73	SNV
YHIM-04	Non-synergy	HAVCR1	chr5	156456801	156456801	C	G	exonic	nonsynonymous SNV	NM_001308156	8	c.G1004C	p.R335T		1	0	38	SNV
YHIM-04	Non-synergy	HELB	chr12	66703560	66703560	C	G	exonic	synonymous SNV	NM_033647	4	c.C852G	p.L284L		0.50920245	80	83	SNV
YHIM-04	Non-synergy	HGC6.3	chr6	168377079	168377079	C	G	exonic	nonsynonymous SNV	NM_001129895	1	c.G254C	p.G85A		0.21311475	96	26	SNV
YHIM-04	Non-synergy	HGC6.3	chr6	168377121	168377121	C	G	exonic	nonsynonymous SNV	NM_001129895	1	c.G212C	p.G71A		0.05263158	126	7	SNV
YHIM-04	Non-synergy	HIST1H1E	chr6	26157047	26157047	G	A	exonic	synonymous SNV	NM_005321	1	c.G429A	p.G143G		1	0	37	SNV
YHIM-04	Non-synergy	HIST1H3C	chr6	26045740	26045740	T	G	exonic	synonymous SNV	NM_003531	1	c.T102G	p.G34G		0.99242424	1	131	SNV
YHIM-04	Non-synergy	HMCN1	chr1	185964040	185964040	C	G	exonic	nonsynonymous SNV	NM_031935	24	c.C3599G	p.P1200R		0.47419355	163	147	SNV
YHIM-04	Non-synergy	HOXD8	chr2	176996238	176996238	C	G	exonic	nonsynonymous SNV	NM_001199746	2	c.C768G	p.N256K		0.50476191	52	53	SNV
YHIM-04	Non-synergy	HRH2	chr5	175110714	175110714	A	C	exonic	nonsynonymous SNV	NM_022304	1	c.A478C	p.S160R		0.48704663	198	188	SNV
YHIM-04	Non-synergy	HRNR	chr1	152192454	152192454	C	G	exonic	nonsynonymous SNV	NM_001009931	3	c.G1651C	p.G551R		0.43478261	104	80	SNV
YHIM-04	Non-synergy	HS3ST4	chr16	26147309	26147309	G	C	exonic	nonsynonymous SNV	NM_006040	2	c.G1111C	p.V371L		0.0625	180	12	SNV
YHIM-04	Non-synergy	ICOS	chr2	204820666	204820666	T	A	exonic	synonymous SNV	NM_012092	2	c.T366A	p.T122T		0.51	49	51	SNV

YHIM-04	Non-synergy	ICOSLG	chr21	45656780	45656780	A	C	exonic	nonsynonymous SNV	NM_001283050	3	c.T376G	p.L126V		0.47328244	138	124	SNV
YHIM-04	Non-synergy	IKBKB	chr8	42128973	42128973	C	G	exonic	nonsynonymous SNV	NM_001190720	1	c.C85G	p.R29G	CCG	0.42307692	15	11	SNV
YHIM-04	Non-synergy	IL17RC	chr3	9971774	9971774	A	G	exonic	nonsynonymous SNV	NM_001203265	12	c.A1123G	p.R375G		1	0	173	SNV
YHIM-04	Non-synergy	INA	chr10	105048340	105048340	G	C	exonic	nonsynonymous SNV	NM_032727	3	c.G1414C	p.E472Q		0.5	15	15	SNV
YHIM-04	Non-synergy	INSR	chr19	7267579	7267579	A	G	exonic	synonymous SNV	NM_000208	2	c.T429C	p.S143S		0.49754902	205	203	SNV
YHIM-04	Non-synergy	IRF2BP1	chr19	46387708	46387708	C	G	exonic	nonsynonymous SNV	NM_015649	1	c.G1325C	p.G442A		0.18699187	300	69	SNV
YHIM-04	Non-synergy	ITGA1	chr5	52161610	52161634	GACC	-	exonic	frameshift deletion	NM_181501	6	c.583_607del	p.D195fs					Indel
YHIM-04	Non-synergy	ITGAV	chr2	187521085	187521085	G	-	exonic	frameshift deletion	NM_001145000	15	c.1568delG	p.R523fs					Indel
YHIM-04	Non-synergy	ITIH3	chr3	52831834	52831855	TCGA	-	exonic	frameshift deletion	NM_002217	6	c.551_572del	p.I184fs					Indel
YHIM-04	Non-synergy	ITSN2	chr2	24516561	24516561	-	G	exonic	frameshift insertion	NM_006277	15	c.1718dupC	p.T573fs					Indel
YHIM-04	Non-synergy	JAKMIP1	chr4	6081910	6081936	CGTC	-	exonic	nonframeshift deletion	NM_001306134	6	c.710_736del	p.237_246del					Indel
YHIM-04	Non-synergy	JAKMIP1	chr4	6083466	6083466	T	C	exonic	nonsynonymous SNV	NM_001306134	5	c.A476G	p.E159G		1	0	95	SNV
YHIM-04	Non-synergy	KAT6B	chr10	76780349	76780349	T	C	exonic	nonsynonymous SNV	NM_001256468	14	c.T2090C	p.L697P	CCG	0.52325581	82	90	SNV
YHIM-04	Non-synergy	KCNQ4	chr1	41296771	41296771	C	T	exonic	synonymous SNV	NM_172163	9	c.C1146T	p.I382I		0.33333333	126	63	SNV
YHIM-04	Non-synergy	KIAA1210	chrX	118221402	118221402	G	A	exonic	nonsynonymous SNV	NM_020721	11	c.C3791T	p.S1264F		0.29454546	194	81	SNV
YHIM-04	Non-synergy	KLF14	chr7	130418529	130418529	G	A	exonic	nonsynonymous SNV	NM_138693	1	c.C332T	p.S111L		0.48275862	45	42	SNV
YHIM-04	Non-synergy	KRT17	chr17	39779233	39779233	C	T	exonic	nonsynonymous SNV	NM_000422	2	c.G484A	p.A162T		0.48529412	35	33	SNV
YHIM-04	Non-synergy	KRT37	chr17	39577200	39577200	G	A	exonic	nonsynonymous SNV	NM_003770	7	c.C1280T	p.T427I		0.43902439	115	90	SNV
YHIM-04	Non-synergy	KRT83	chr12	52711471	52711471	G	A	exonic	synonymous SNV	NM_002282	4	c.C744T	p.Y248Y		0.78571429	3	11	SNV
YHIM-04	Non-synergy	LAG3	chr12	6886579	6886583	GCCC	-	exonic	frameshift deletion	NM_002286	6	c.1207_1211del	p.A403fs					Indel
YHIM-04	Non-synergy	LAG3	chr12	6884683	6884683	C	A	exonic	synonymous SNV	NM_002286	5	c.C1026A	p.L342L		0.39638554	501	329	SNV
YHIM-04	Non-synergy	LAMA3	chr18	21427474	21427474	C	T	exonic	synonymous SNV	NM_001127717	32	c.C3978T	p.C1326C		0.52830189	175	196	SNV
YHIM-04	Non-synergy	LAMB4	chr7	107720150	107720150	C	T	exonic	nonsynonymous SNV	NM_007356	15	c.G1783A	p.G595R		0.55238095	94	116	SNV
YHIM-04	Non-synergy	LAMC2	chr1	183192307	183192307	A	C	exonic	nonsynonymous SNV	NM_005562	7	c.A801C	p.Q267H		0.48148148	56	52	SNV
YHIM-04	Non-synergy	LCE3C	chr1	152573417	152573434	CAAC	-	exonic	nonframeshift deletion	NM_178434	1	c.210_227del	p.70_76del					Indel
YHIM-04	Non-synergy	LDB1	chr10	103868036	103868036	G	A	exonic	synonymous SNV	NM_001113407	11	c.C1050T	p.F350F		0.53813559	109	127	SNV
YHIM-04	Non-synergy	LINS1	chr15	101114418	101114418	G	A	exonic	synonymous SNV	NM_001040616	5	c.C660T	p.F220F		0.47619048	44	40	SNV
YHIM-04	Non-synergy	LMAN1L	chr15	75108552	75108552	G	C	exonic	nonsynonymous SNV	NM_021819	2	c.G230C	p.S77T		0.48113208	55	51	SNV
YHIM-04	Non-synergy	LMF2	chr22	50944800	50944800	A	T	exonic	nonsynonymous SNV	NM_033200	4	c.T509A	p.L170Q		0.24396783	282	91	SNV
YHIM-04	Non-synergy	LPCAT4	chr15	34657835	34657835	A	T	exonic	nonsynonymous SNV	NM_153613	2	c.T182A	p.L61H		0.53846154	30	35	SNV
YHIM-04	Non-synergy	LRGUK	chr7	133863333	133863333	G	C	exonic	nonsynonymous SNV	NM_144648	10	c.G1186C	p.V396L		0.45248869	121	100	SNV
YHIM-04	Non-synergy	LRP2	chr2	170112630	170112630	A	T	exonic	nonsynonymous SNV	NM_004525	19	c.T2756A	p.L919H		1	0	114	SNV
YHIM-04	Non-synergy	LY9	chr1	160793476	160793476	G	T	exonic	nonsynonymous SNV	NM_001261457	7	c.G1450T	p.D484Y		0.46654275	287	251	SNV
YHIM-04	Non-synergy	MACF1	chr1	39826516	39826516	C	A	exonic	nonsynonymous SNV	NM_012090	42	c.C6110A	p.S2037Y		0.66666667	57	114	SNV
YHIM-04	Non-synergy	MAGEB17	chrX	16188910	16188910	G	A	exonic	nonsynonymous SNV	NM_001277307	2	c.G405A	p.M135I		0.47317073	108	97	SNV
YHIM-04	Non-synergy	MAGEB6	chrX	26212043	26212043	G	T	exonic	nonsynonymous SNV	NM_173523	2	c.G80T	p.G27V		0.45619335	180	151	SNV
YHIM-04	Non-synergy	MAMLD1	chrX	149638110	149638110	G	C	exonic	nonsynonymous SNV	NM_001177466	2	c.G190C	p.E64Q		0.49056604	54	52	SNV
YHIM-04	Non-synergy	MAP10	chr1	232940868	232940868	G	A	exonic	synonymous SNV	NM_019090	1	c.G99A	p.L33L		0.26704546	258	94	SNV
YHIM-04	Non-synergy	MAP7D3	chrX	135326933	135326933	A	T	exonic	nonsynonymous SNV	NM_001173516	4	c.T221A	p.L74H		0.62068966	55	90	SNV
YHIM-04	Non-synergy	MATR3	chr5	138643173	138643173	G	A	exonic	synonymous SNV	NM_001194955	2	c.G69A	p.A23A		0.02960526	295	9	SNV
YHIM-04	Non-synergy	MATR3	chr5	138643914	138643914	A	G	exonic	synonymous SNV	NM_001194955	2	c.A810G	p.R270R		0.04587156	208	10	SNV
YHIM-04	Non-synergy	ME3	chr11	86161003	86161003	C	G	exonic	nonsynonymous SNV	NM_001014811	9	c.G1059C	p.E353D		0.50314465	79	80	SNV
YHIM-04	Non-synergy	MED12	chrX	70342984	70342984	G	C	exonic	nonsynonymous SNV	NM_005120	11	c.G1525C	p.E509Q	CCG	0.46992481	141	125	SNV
YHIM-04	Non-synergy	MED14OS	chrX	40594987	40594987	G	A	exonic	nonsynonymous SNV	NM_001289773	1	c.G287A	p.R96Q		0.03913044	221	9	SNV
YHIM-04	Non-synergy	MEIOB	chr16	1891849	1891849	C	A	exonic	stopgain	NM_001163560	11	c.G1006T	p.E336X		0.52631579	27	30	SNV
YHIM-04	Non-synergy	MEP1B	chr18	29797033	29797033	T	G	exonic	nonsynonymous SNV	NM_001308171	13	c.T1839G	p.C613W		0.47803618	202	185	SNV
YHIM-04	Non-synergy	MFRP	chr11	119213375	119213375	A	C	exonic	nonsynonymous SNV	NM_031433	11	c.T1318G	p.C440G		0.99300699	1	142	SNV
YHIM-04	Non-synergy	MICAL2	chr11	12241964	12241964	C	A	exonic	nonsynonymous SNV	NM_001282666	7	c.C1165A	p.H389N		0.05434783	87	5	SNV
YHIM-04	Non-synergy	MLLT4	chr6	168349031	168349031	G	C	exonic	nonsynonymous SNV	NM_001207008	27	c.G3632C	p.R1211T	CCG	0.45098039	84	69	SNV
YHIM-04	Non-synergy	MMRN2	chr10	88702438	88702438	G	C	exonic	synonymous SNV	NM_024756	6	c.C2103G	p.L701L		1	0	46	SNV
YHIM-04	Non-synergy	MPI	chr15	75190013	75190013	C	A	exonic	stopgain	NM_001289156	7	c.C1064A	p.S355X		0.47435897	123	111	SNV
YHIM-04	Non-synergy	MRAS	chr3	138116250	138116250	C	G	exonic	nonsynonymous SNV	NM_001252091	2	c.C50G	p.S17C		0.25088339	636	213	SNV
YHIM-04	Non-synergy	MROH7	chr1	55152080	55152080	C	A	exonic	nonsynonymous SNV	NM_001291332	13	c.C1224A	p.D408E		0.47039474	161	143	SNV
YHIM-04	Non-synergy	MS4A6A	chr11	59947358	59947358	G	C	exonic	synonymous SNV	NM_022349	3	c.C228G	p.T76T		0.45418327	137	114	SNV
YHIM-04	Non-synergy	MST1L	chr1	17087577	17087577	C	G	exonic	nonsynonymous SNV	NM_001271733	2	c.G88C	p.D30H		0.55555556	72	90	SNV
YHIM-04	Non-synergy	MTPAP	chr10	30638115	30638115	C	G	exonic	nonsynonymous SNV	NM_018109	1	c.G90C	p.L30F		0.43930636	194	152	SNV
YHIM-04	Non-synergy	MUC4	chr3	195512078	195512078	C	G	exonic	nonsynonymous SNV	NM_018406	2	c.G6373C	p.D2125H		0.05714286	132	8	SNV
YHIM-04	Non-synergy	MYH15	chr3	108158630	108158630	C	G	exonic	nonsynonymous SNV	NM_014981	25	c.G3089C	p.S1030T		0.31372549	140	64	SNV
YHIM-04	Non-synergy	MYO18B	chr22	26423161	26423161	G	C	exonic	nonsynonymous SNV	NM_032608	43	c.G7221C	p.Q2407H		0.48563969	197	186	SNV
YHIM-04	Non-synergy	MYOC	chr1	171605567	171605567	C	T	exonic	nonsynonymous SNV	NM_000261	3	c.G1013A	p.G338D		0.4778157	153	140	SNV
YHIM-04	Non-synergy	MYRF	chr11	61536746	61536746	C	G	exonic	nonsynonymous SNV	NM_001127392	4	c.C413G	p.S138C		0.048	119	6	SNV
YHIM-04	Non-synergy	N4BP3	chr5	177546586	177546586	T	G	exonic	nonsynonymous SNV	NM_015111	2	c.T2G	p.M1R		1	0	130	SNV

YHIM-04	Non-synergy	NAA25	chr12	112481560	112481560	C	A	exonic	nonsynonymous SNV	NM_024953	18	c.G2119T	p.V707L		0.45378151	130	108	SNV
YHIM-04	Non-synergy	NAV3	chr12	78392198	78392198	G	C	exonic	nonsynonymous SNV	NM_001024383	7	c.G822C	p.Q274H		0.46511628	46	40	SNV
YHIM-04	Non-synergy	NCOR2	chr12	124838676	124838676	G	A	exonic	nonsynonymous SNV	NM_001077261	28	c.C3626T	p.S1209L	CCG	0.45714286	152	128	SNV
YHIM-04	Non-synergy	NFAT5	chr16	69726323	69726323	C	T	exonic	synonymous SNV	NM_006599	12	c.C2541T	p.S847S		0.03472222	139	5	SNV
YHIM-04	Non-synergy	NLGN3	chrX	70389565	70389565	A	G	exonic	nonsynonymous SNV	NM_001166660	6	c.A2045G	p.N682S		0.515625	124	132	SNV
YHIM-04	Non-synergy	NPEPPS	chr17	45663749	45663749	G	A	exonic	nonsynonymous SNV	NM_006310	8	c.G965A	p.G322D		0.07142857	65	5	SNV
YHIM-04	Non-synergy	NPFFR1	chr10	72015144	72015144	C	G	exonic	nonsynonymous SNV	NM_022146	4	c.G862C	p.A288P		0.50503018	246	251	SNV
YHIM-04	Non-synergy	NT5C1A	chr1	40137615	40137615	A	T	exonic	synonymous SNV	NM_032526	1	c.T96A	p.I32I		0.30510949	476	209	SNV
YHIM-04	Non-synergy	OPRM1	chr6	154412540	154412540	C	T	exonic	nonsynonymous SNV	NM_001285527	2	c.C797T	p.T266I		0.43831169	173	135	SNV
YHIM-04	Non-synergy	OR10J5	chr1	159505213	159505213	C	G	exonic	nonsynonymous SNV	NM_001004469	1	c.G585C	p.E195D		0.47619048	154	140	SNV
YHIM-04	Non-synergy	OR2A25	chr7	143771960	143771960	T	G	exonic	synonymous SNV	NM_001004488	1	c.T648G	p.S216S		0.5	97	97	SNV
YHIM-04	Non-synergy	ORC1	chr1	52859371	52859371	T	A	exonic	stopgain	NM_001190818	6	c.A826T	p.K276X		0.49606299	64	63	SNV
YHIM-04	Non-synergy	ORC1	chr1	52859372	52859372	A	G	exonic	synonymous SNV	NM_001190818	6	c.T825C	p.S275S		0.488	64	61	SNV
YHIM-04	Non-synergy	OSTM1	chr6	108370484	108370484	C	G	exonic	nonsynonymous SNV	NM_014028	5	c.G922C	p.E308Q		0.48743719	102	97	SNV
YHIM-04	Non-synergy	OTUD5	chrX	48791832	48791832	C	A	exonic	nonsynonymous SNV	NM_001136157	5	c.G964T	p.V322F		0.47533632	117	106	SNV
YHIM-04	Non-synergy	OVCH1	chr12	29604487	29604487	G	C	exonic	nonsynonymous SNV	NM_183378	22	c.C2546G	p.A849G		0.55333333	67	83	SNV
YHIM-04	Non-synergy	PABPC1L	chr20	43538967	43538967	G	C	exonic	nonsynonymous SNV	NM_001124756	1	c.G183C	p.Q61H		0.48598131	55	52	SNV
YHIM-04	Non-synergy	PAPD7	chr5	6753066	6753066	T	C	exonic	synonymous SNV	NM_001171806	6	c.T810C	p.S270S		0.19631902	393	96	SNV
YHIM-04	Non-synergy	PCDH15	chr10	55591229	55591229	G	A	exonic	nonsynonymous SNV	NM_001142765	28	c.C3835T	p.R1279C		0.41071429	66	46	SNV
YHIM-04	Non-synergy	PCDH18	chr4	138452270	138452270	G	A	exonic	stopgain	NM_001300828	1	c.C973T	p.Q325X		1	0	57	SNV
YHIM-04	Non-synergy	PCDHA4	chr5	140188586	140188586	C	T	exonic	nonsynonymous SNV	NM_018907	1	c.C1814T	p.A605V		0.52879581	90	101	SNV
YHIM-04	Non-synergy	PCDHB15	chr5	140625258	140625258	G	C	exonic	nonsynonymous SNV	NM_018935	1	c.G112C	p.E38Q		0.49242424	67	65	SNV
YHIM-04	Non-synergy	PDE4B	chr1	66838323	66838323	G	C	exonic	nonsynonymous SNV	NM_001037339	10	c.G1657C	p.D553H		1	0	66	SNV
YHIM-04	Non-synergy	PDE4DIP	chr1	144879325	144879325	G	C	exonic	synonymous SNV	NM_001198834	27	c.C4125G	p.L1375L	CCG	0.31372549	70	32	SNV
YHIM-04	Non-synergy	PDZD7	chr10	102769095	102769095	G	C	exonic	nonsynonymous SNV	NM_001195263	16	c.C2630G	p.S877C		0.49211357	161	156	SNV
YHIM-04	Non-synergy	PER1	chr17	8049721	8049721	C	G	exonic	nonsynonymous SNV	NM_002616	16	c.G2007C	p.R669S	CCG	1	0	88	SNV
YHIM-04	Non-synergy	PEX6	chr6	42946116	42946116	C	T	exonic	nonsynonymous SNV	NM_000287	1	c.G773A	p.G258D		0.55208333	129	159	SNV
YHIM-04	Non-synergy	PFKFB4	chr3	48587314	48587314	C	G	exonic	nonsynonymous SNV	NM_001317135	3	c.G294C	p.E98D		1	0	54	SNV
YHIM-04	Non-synergy	PHF14	chr7	11022400	11022400	G	C	exonic	nonsynonymous SNV	NM_014660	3	c.G514C	p.A172P		0.42857143	88	66	SNV
YHIM-04	Non-synergy	PI4KA	chr22	21157486	21157486	G	A	exonic	synonymous SNV	NM_058004	13	c.C1584T	p.Y528Y		0.44584383	220	177	SNV
YHIM-04	Non-synergy	PIAS3	chr1	145584148	145584148	C	G	exonic	synonymous SNV	NM_006099	11	c.C1299G	p.V433V		0.49431818	89	87	SNV
YHIM-04	Non-synergy	PIFO	chr1	111891261	111891261	T	G	exonic	nonsynonymous SNV	NM_001300831	3	c.T283G	p.Y95D		0.05945946	174	11	SNV
YHIM-04	Non-synergy	PKN3	chr9	131475440	131475440	C	G	exonic	synonymous SNV	NM_013355	7	c.C945G	p.T315T		0.39393939	220	143	SNV
YHIM-04	Non-synergy	PLA2G4D	chr15	42379633	42379633	C	T	exonic	synonymous SNV	NM_178034	3	c.G120A	p.L40L		0.47206704	189	169	SNV
YHIM-04	Non-synergy	PLCE1	chr10	96053343	96053343	C	A	exonic	nonsynonymous SNV	NM_001165979	22	c.C4190A	p.P1397Q		0.30769231	198	88	SNV
YHIM-04	Non-synergy	PLPPR4	chr1	99762325	99762325	G	A	exonic	nonsynonymous SNV	NM_001166252	3	c.G440A	p.C147Y		1	0	72	SNV
YHIM-04	Non-synergy	PLTP	chr20	44530924	44530924	G	A	exonic	nonsynonymous SNV	NM_001242920	10	c.C872T	p.T291M		0.6119403	52	82	SNV
YHIM-04	Non-synergy	PODXL	chr7	131194225	131194225	G	A	exonic	nonsynonymous SNV	NM_005397	3	c.C826T	p.P276S		0.48325359	108	101	SNV
YHIM-04	Non-synergy	POFUT1	chr20	30795852	30795852	C	G	exonic	synonymous SNV	NM_015352	1	c.C108G	p.L36L		0.27131783	188	70	SNV
YHIM-04	Non-synergy	POLG	chr15	89871750	89871757	CAGT	-	exonic	frameshift deletion	NM_001126131	6	c.1180_1187del	p.Q394fs					Indel
YHIM-04	Non-synergy	POM121L12	chr7	53104169	53104169	G	T	exonic	stopgain	NM_182595	1	c.G805T	p.E269X		0.4778157	153	140	SNV
YHIM-04	Non-synergy	POU6F2	chr7	39125590	39125590	T	C	exonic	nonsynonymous SNV	NM_001166018	3	c.T149C	p.V50A		0.05714286	99	6	SNV
YHIM-04	Non-synergy	POU6F2	chr7	39125588	39125588	T	C	exonic	synonymous SNV	NM_001166018	3	c.T147C	p.P49P		0.05769231	98	6	SNV
YHIM-04	Non-synergy	PPARA	chr22	46627912	46627912	G	A	exonic	nonsynonymous SNV	NM_001001928	7	c.G935A	p.G312E		0.32545455	371	179	SNV
YHIM-04	Non-synergy	PPFIBP1	chr12	27835386	27835386	G	C	exonic	nonsynonymous SNV	NM_001198915	20	c.G1672C	p.E558Q	CCG	0.35955056	114	64	SNV
YHIM-04	Non-synergy	PPFIBP2	chr11	7652251	7652251	A	C	exonic	nonsynonymous SNV	NM_001256569	7	c.A631C	p.K211Q		1	0	91	SNV
YHIM-04	Non-synergy	PPM1N	chr19	46002036	46002036	C	G	exonic	synonymous SNV	NM_001080401	1	c.C306G	p.L102L		0.19525959	713	173	SNV
YHIM-04	Non-synergy	PPP1R13B	chr14	104201523	104201523	G	A	exonic	nonsynonymous SNV	NM_015316	17	c.C3241T	p.R1081W		1	0	37	SNV
YHIM-04	Non-synergy	PRDM6	chr5	122506580	122506580	C	A	exonic	nonsynonymous SNV	NM_001136239	6	c.C1274A	p.P425Q		0.45487365	151	126	SNV
YHIM-04	Non-synergy	PRNP	chr20	4680302	4680302	G	T	exonic	stopgain	NM_000311	2	c.G436T	p.E146X		0.34080718	294	152	SNV
YHIM-04	Non-synergy	PRSS56	chr2	233388648	233388648	G	T	exonic	synonymous SNV	NM_001195129	9	c.G1179T	p.R393R		0.05454546	104	6	SNV
YHIM-04	Non-synergy	PSG5	chr19	43679455	43679455	A	C	exonic	synonymous SNV	NM_001130014	4	c.T876G	p.T292T		0.31623932	80	37	SNV
YHIM-04	Non-synergy	PSMC4	chr19	40486432	40486432	A	G	exonic	nonsynonymous SNV	NM_006503	9	c.A1058G	p.N353S		0.25549451	271	93	SNV
YHIM-04	Non-synergy	PTCH1	chr9	98242303	98242303	C	A	exonic	stopgain	NM_000264	7	c.G1015T	p.E339X	CCG	0.54424779	103	123	SNV
YHIM-04	Non-synergy	PTPN23	chr3	47437641	47437641	G	A	exonic	synonymous SNV	NM_015466	2	c.G93A	p.L31L		1	0	53	SNV
YHIM-04	Non-synergy	PTPRZ1	chr7	121652780	121652780	C	G	exonic	stopgain	NM_002851	12	c.C3680G	p.S1227X		0.73359073	69	190	SNV
YHIM-04	Non-synergy	QSOX1	chr1	180124158	180124158	C	T	exonic	nonsynonymous SNV	NM_001004128	1	c.C116T	p.S39F		0.44262295	34	27	SNV
YHIM-04	Non-synergy	RAB8B	chr15	63547749	63547749	A	G	exonic	nonsynonymous SNV	NM_016530	4	c.A290G	p.D97G		0.52688172	44	49	SNV
YHIM-04	Non-synergy	RAPGEF5	chr7	22194218	22194218	G	C	exonic	nonsynonymous SNV	NM_012294	17	c.C1182G	p.F394L		0.52173913	44	48	SNV
YHIM-04	Non-synergy	RARRES3	chr11	63312314	63312314	G	C	exonic	nonsynonymous SNV	NM_004585	3	c.G340C	p.E114Q		0.02910053	367	11	SNV
YHIM-04	Non-synergy	RB1CC1	chr8	53537329	53537329	C	G	exonic	nonsynonymous SNV	NM_001083617	23	c.G4646C	p.R1549T		0.36144578	53	30	SNV
YHIM-04	Non-synergy	RBP3	chr10	48389779	48389779	C	T	exonic	nonsynonymous SNV	NM_002900	1	c.G1099A	p.D367N		0.53518124	218	251	SNV

YHIM-04	Non-synergy	RBP5	chr12	7280923	7280923	G	A	exonic	synonymous SNV	NM_031491	2	c.C165T	p.L55L		0.29671717	557	235	SNV
YHIM-04	Non-synergy	RIPK1	chr6	3106131	3106144	TGGA	-	exonic	frameshift deletion	NM_003804	9	c.1422_1435del	p.F474fs					Indel
YHIM-04	Non-synergy	RLBP1	chr15	89758330	89758330	-	T	exonic	frameshift insertion	NM_000326	6	c.485dupA	p.N162fs					Indel
YHIM-04	Non-synergy	RNF187	chr1	228675250	228675250	G	A	exonic	nonsynonymous SNV	NM_001010858	1	c.G61A	p.E21K		0.30769231	27	12	SNV
YHIM-04	Non-synergy	RPRD2	chr1	150429974	150429974	C	G	exonic	nonsynonymous SNV	NM_001297673	7	c.C1003G	p.P335A		0.43684211	107	83	SNV
YHIM-04	Non-synergy	RSPH6A	chr19	46308221	46308221	G	A	exonic	synonymous SNV	NM_030785	3	c.C942T	p.A314A		0.20432692	662	170	SNV
YHIM-04	Non-synergy	RYR3	chr15	34014922	34014922	G	C	exonic	nonsynonymous SNV	NM_001036	44	c.G6626C	p.S2209T		0.64948454	34	63	SNV
YHIM-04	Non-synergy	SAMD4A	chr14	55203869	55203869	C	T	exonic	synonymous SNV	NM_015589	3	c.C843T	p.P281P		0.06145251	168	11	SNV
YHIM-04	Non-synergy	SCIN	chr7	12610465	12610465	G	A	exonic	nonsynonymous SNV	NM_001112706	1	c.G53A	p.G18E		0.52542373	112	124	SNV
YHIM-04	Non-synergy	SEMA4D	chr9	91995972	91995972	G	A	exonic	nonsynonymous SNV	NM_001142287	17	c.C1661T	p.P554L		0.47609562	263	239	SNV
YHIM-04	Non-synergy	SERINC4	chr15	44089074	44089074	G	C	exonic	nonsynonymous SNV	NM_001258032	6	c.C177G	p.F59L		0.44444444	80	64	SNV
YHIM-04	Non-synergy	SETBP1	chr18	42643645	42643645	G	C	exonic	nonsynonymous SNV	NM_015559	6	c.G4773C	p.E1591D	CCG	0.35897436	75	42	SNV
YHIM-04	Non-synergy	SFI1	chr22	32002358	32002358	A	T	exonic	nonsynonymous SNV	NM_001258325	19	c.A1934T	p.D645V		0.44144144	62	49	SNV
YHIM-04	Non-synergy	SH2D3C	chr9	130501043	130501043	G	C	exonic	synonymous SNV	NM_001142533	10	c.C2091G	p.V697V		0.30172414	324	140	SNV
YHIM-04	Non-synergy	SHISA8	chr22	42310526	42310526	G	T	exonic	nonsynonymous SNV	NM_001207020	1	c.C146A	p.P49H		0.60465116	17	26	SNV
YHIM-04	Non-synergy	SIRPA	chr20	1903024	1903024	C	G	exonic	nonsynonymous SNV	NM_001040023	4	c.C820G	p.Q274E		0.35987261	201	113	SNV
YHIM-04	Non-synergy	SLC25A27	chr6	46626740	46626740	G	C	exonic	nonsynonymous SNV	NM_001204051	3	c.G340C	p.E114Q		0.49640288	140	138	SNV
YHIM-04	Non-synergy	SLC25A52	chr18	29340436	29340436	G	C	exonic	nonsynonymous SNV	NM_001034172	1	c.C219G	p.I73M		0.4520548	40	33	SNV
YHIM-04	Non-synergy	SLC33A1	chr3	155547499	155547499	C	T	exonic	nonsynonymous SNV	NM_001190992	5	c.G1460A	p.C487Y		0.23826715	422	132	SNV
YHIM-04	Non-synergy	SLC35F1	chr6	118229057	118229057	G	T	exonic	synonymous SNV	NM_001029858	1	c.G168T	p.L56L		0.52272727	21	23	SNV
YHIM-04	Non-synergy	SLC46A2	chr9	115652919	115652919	G	A	exonic	nonsynonymous SNV	NM_033051	1	c.C43T	p.R15C		0.49019608	26	25	SNV
YHIM-04	Non-synergy	SLC5A10	chr17	18863873	18863873	C	G	exonic	nonsynonymous SNV	NM_001042450	5	c.C361G	p.P121A		0.95454546	6	126	SNV
YHIM-04	Non-synergy	SLC7A8	chr14	23597363	23597363	A	T	exonic	nonsynonymous SNV	NM_001267037	7	c.T634A	p.F212I		1	0	298	SNV
YHIM-04	Non-synergy	SLCO2B1	chr11	74913962	74913962	G	C	exonic	nonsynonymous SNV	NM_001145212	9	c.G1348C	p.D450H		0.40322581	111	75	SNV
YHIM-04	Non-synergy	SOAT2	chr12	53514581	53514581	-	T	exonic	frameshift insertion	NM_003578	11	c.1052dupT	p.L351fs					Indel
YHIM-04	Non-synergy	SON	chr21	34948143	34948143	G	C	exonic	nonsynonymous SNV	NM_001291412	10	c.G1249C	p.E417Q		0.39285714	51	33	SNV
YHIM-04	Non-synergy	SPATA5L1	chr15	45695044	45695044	G	C	exonic	synonymous SNV	NM_024063	1	c.G417C	p.L139L		0.46502058	130	113	SNV
YHIM-04	Non-synergy	SPHKAP	chr2	228855720	228855720	T	C	exonic	nonsynonymous SNV	NM_030623	10	c.A4868G	p.E1623G		0.45689655	126	106	SNV
YHIM-04	Non-synergy	SPTAN1	chr9	131394432	131394432	G	A	exonic	nonsynonymous SNV	NM_001195532	51	c.G6714A	p.M2238I		0.29834254	254	108	SNV
YHIM-04	Non-synergy	ST3GAL3	chr1	44365320	44365320	G	A	exonic	nonsynonymous SNV	NM_001270462	7	c.G524A	p.R175H		0.65924658	199	385	SNV
YHIM-04	Non-synergy	STEAP3	chr2	120005340	120005340	G	A	exonic	nonsynonymous SNV	NM_001008410	3	c.G578A	p.G193E		0.45454546	144	120	SNV
YHIM-04	Non-synergy	STK32B	chr4	5469791	5469791	G	C	exonic	nonsynonymous SNV	NM_018401	11	c.G1100C	p.R367T		1	0	67	SNV
YHIM-04	Non-synergy	SUPT20HL1	chrX	24381692	24381696	AAAG	-	exonic	frameshift deletion	NM_001136234	1	c.815_819del	p.E272fs					Indel
YHIM-04	Non-synergy	SUPT6H	chr17	27003284	27003284	G	A	exonic	nonsynonymous SNV	NM_003170	7	c.G733A	p.D245N		0.47639485	122	111	SNV
YHIM-04	Non-synergy	SVOPL	chr7	138356765	138356765	G	T	exonic	nonsynonymous SNV	NM_001139456	3	c.C272A	p.T91K		0.42857143	36	27	SNV
YHIM-04	Non-synergy	SYNE1	chr6	152737905	152737905	T	G	exonic	nonsynonymous SNV	NM_033071	41	c.A5688C	p.Q1896H		0.48746518	184	175	SNV
YHIM-04	Non-synergy	SYT15	chr10	46965869	46965869	A	G	exonic	nonsynonymous SNV	NM_031912	5	c.T668C	p.I223T		0.24193548	188	60	SNV
YHIM-04	Non-synergy	TAF2	chr8	120816203	120816203	G	A	exonic	nonsynonymous SNV	NM_003184	5	c.C475T	p.L159F		0.25117371	319	107	SNV
YHIM-04	Non-synergy	TAOK1	chr17	27844513	27844513	G	C	exonic	nonsynonymous SNV	NM_020791	16	c.G1747C	p.E583Q		0.57471264	37	50	SNV
YHIM-04	Non-synergy	TAS1R2	chr1	19181268	19181268	G	C	exonic	nonsynonymous SNV	NM_152232	3	c.C696G	p.I232M		0.42183623	233	170	SNV
YHIM-04	Non-synergy	TBC1D16	chr17	77924295	77924295	C	G	exonic	nonsynonymous SNV	NM_001271844	2	c.G89C	p.S30T		1	0	178	SNV
YHIM-04	Non-synergy	TCEB3C,TCE	chr18	44555349	44555349	C	G	exonic	nonsynonymous SNV	NM_001100817	1	c.G865C	p.E289Q		0.05223881	127	7	SNV
YHIM-04	Non-synergy	TLR5	chr1	223284507	223284507	A	G	exonic	nonsynonymous SNV	NM_003268	6	c.T1867C	p.S623P		0.375	120	72	SNV
YHIM-04	Non-synergy	TLR6	chr4	38830005	38830005	C	T	exonic	nonsynonymous SNV	NM_006068	2	c.G1090A	p.V364I		1	0	56	SNV
YHIM-04	Non-synergy	TMEM107	chr17	8079586	8079586	G	T	exonic	nonsynonymous SNV	NM_032354	1	c.C19A	p.L7I		1	0	65	SNV
YHIM-04	Non-synergy	TMEM132E	chr17	32961837	32961837	G	A	exonic	nonsynonymous SNV	NM_001304438	7	c.G1708A	p.D570N		0.52	72	78	SNV
YHIM-04	Non-synergy	TMEM173	chr5	138860775	138860775	G	C	exonic	nonsynonymous SNV	NM_001301738	3	c.C380G	p.S127W		0.4	42	28	SNV
YHIM-04	Non-synergy	TMEM232	chr5	109963526	109963526	T	C	exonic	nonsynonymous SNV	NM_001039763	6	c.A557G	p.E186G		0.52631579	9	10	SNV
YHIM-04	Non-synergy	TMEM245	chr9	111826790	111826790	C	A	exonic	synonymous SNV	NM_032012	10	c.G1590T	p.V530V		0.46464647	53	46	SNV
YHIM-04	Non-synergy	TMEM25	chr11	118403156	118403156	C	T	exonic	nonsynonymous SNV	NM_001144034	3	c.C362T	p.S121F		1	0	180	SNV
YHIM-04	Non-synergy	TMEM91	chr19	41888787	41888787	C	T	exonic	synonymous SNV	NM_001042595	3	c.C321T	p.F107F		0.23684211	609	189	SNV
YHIM-04	Non-synergy	TNR	chr1	175365895	175365895	A	T	exonic	nonsynonymous SNV	NM_003285	5	c.T1025A	p.I342N		0.41588785	125	89	SNV
YHIM-04	Non-synergy	TRIM51	chr11	55659025	55659025	G	T	exonic	nonsynonymous SNV	NM_032681	7	c.G1276T	p.D426Y		0.53608247	45	52	SNV
YHIM-04	Non-synergy	TRPM7	chr15	50929649	50929649	T	C	exonic	nonsynonymous SNV	NM_001301212	7	c.A802G	p.K268E		0.53820598	139	162	SNV
YHIM-04	Non-synergy	TRRAP	chr7	98569565	98569565	G	A	exonic	synonymous SNV	NM_003496	51	c.G7761A	p.V2587V	CCG	0.50561798	44	45	SNV
YHIM-04	Non-synergy	TSC1	chr9	135802686	135802686	C	A	exonic	nonsynonymous SNV	NM_000368	4	c.G112T	p.G38C	CCG	0.47826087	156	143	SNV
YHIM-04	Non-synergy	TSHZ3	chr19	31770434	31770434	C	G	exonic	nonsynonymous SNV	NM_020856	2	c.G265C	p.E89Q		0.35977337	226	127	SNV
YHIM-04	Non-synergy	TTC17	chr11	43400872	43400872	G	T	exonic	nonsynonymous SNV	NM_001307943	2	c.G249T	p.E83D		1	0	176	SNV
YHIM-04	Non-synergy	TYRO3	chr15	41859620	41859621	CT	-	exonic	frameshift deletion	NM_006293	7	c.846_847del	p.T282fs					Indel
YHIM-04	Non-synergy	TYSND1	chr10	71905823	71905823	C	G	exonic	nonsynonymous SNV	NM_001040273	1	c.G520C	p.E174Q		0.02888087	269	8	SNV
YHIM-04	Non-synergy	UBC	chr12	125397364	125397364	A	G	exonic	synonymous SNV	NM_021009	2	c.T954C	p.T318T		0.07272727	51	4	SNV
YHIM-04	Non-synergy	UGT2A1,UG	chr4	70504929	70504929	A	T	exonic	nonsynonymous SNV	NM_001105677	1	c.T430A	p.L144M		0.04195804	274	12	SNV

YHIM-04	Non-synergy	UNC5C	chr4	96141313	96141313	C	T	exonic	nonsynonymous SNV	NM_003728	8	c.G1123A	p.D375N		0.99074074	1	107	SNV
YHIM-04	Non-synergy	UNC5C	chr4	96141319	96141319	C	G	exonic	nonsynonymous SNV	NM_003728	8	c.G1117C	p.D373H		0.99056604	1	105	SNV
YHIM-04	Non-synergy	URB2	chr1	229787055	229787055	G	A	exonic	nonsynonymous SNV	NM_001314021	8	c.G4223A	p.R1408Q		0.30787037	299	133	SNV
YHIM-04	Non-synergy	USP21	chr1	161130610	161130610	T	A	exonic	nonsynonymous SNV	NM_012475	2	c.T180A	p.D60E		0.52784504	195	218	SNV
YHIM-04	Non-synergy	USP22	chr17	20931949	20931949	G	T	exonic	synonymous SNV	NM_015276	2	c.C210A	p.L70L		1	0	69	SNV
YHIM-04	Non-synergy	USP32	chr17	58258729	58258729	C	G	exonic	nonsynonymous SNV	NM_032582	32	c.G4504C	p.E1502Q		1	0	17	SNV
YHIM-04	Non-synergy	UTP14A	chrX	129045868	129045868	C	T	exonic	nonsynonymous SNV	NM_006649	6	c.C508T	p.P170S		0.11111111	104	13	SNV
YHIM-04	Non-synergy	UTP15	chr5	72865362	72865362	G	A	exonic	nonsynonymous SNV	NM_001284430	5	c.G352A	p.V118M		1	0	28	SNV
YHIM-04	Non-synergy	WDR20	chr14	102606537	102606537	G	A	exonic	nonsynonymous SNV	NM_001242418	1	c.G277A	p.A93T		1	0	31	SNV
YHIM-04	Non-synergy	WNT8A	chr5	137426721	137426721	T	A	exonic	nonsynonymous SNV	NM_001300939	5	c.T1069A	p.S357T		0.51724138	42	45	SNV
YHIM-04	Non-synergy	WNT8A	chr5	137426722	137426722	C	A	exonic	nonsynonymous SNV	NM_001300939	5	c.C1070A	p.S357Y		0.51136364	43	45	SNV
YHIM-04	Non-synergy	YEATS4	chr12	69753758	69753758	C	G	exonic	nonsynonymous SNV	NM_001300950	1	c.C6G	p.F2L		0.43162393	133	101	SNV
YHIM-04	Non-synergy	ZBTB4	chr17	7365330	7365330	G	A	exonic	nonsynonymous SNV	NM_001128833	4	c.C2971T	p.P991S		1	0	125	SNV
YHIM-04	Non-synergy	ZBTB4	chr17	7365351	7365351	G	C	exonic	nonsynonymous SNV	NM_001128833	4	c.C2950G	p.P984A		1	0	138	SNV
YHIM-04	Non-synergy	ZFYVE9	chr1	52704470	52704470	G	T	exonic	nonsynonymous SNV	NM_004799	4	c.G1381T	p.D461Y		0.02721088	286	8	SNV
YHIM-04	Non-synergy	ZNF225	chr19	44635893	44635893	G	C	exonic	nonsynonymous SNV	NM_013362	5	c.G1126C	p.E376Q		0.15057915	660	117	SNV
YHIM-04	Non-synergy	ZNF277	chr7	111936316	111936316	G	A	exonic	nonsynonymous SNV	NM_021994	4	c.G415A	p.V139I		0.37142857	22	13	SNV
YHIM-04	Non-synergy	ZNF404	chr19	44377566	44377566	C	G	exonic	nonsynonymous SNV	NM_001033719	2	c.G791C	p.G264A		0.365625	203	117	SNV
YHIM-04	Non-synergy	ZNF467	chr7	149463119	149463119	T	C	exonic	nonsynonymous SNV	NM_207336	5	c.A472G	p.K158E		0.41904762	61	44	SNV
YHIM-04	Non-synergy	ZNF598	chr16	2049786	2049786	C	A	exonic	synonymous SNV	NM_178167	11	c.G1764T	p.G588G		0.41860465	100	72	SNV
YHIM-04	Non-synergy	ZNF670	chr1	247201527	247201527	C	G	exonic	nonsynonymous SNV	NM_001204220	4	c.G391C	p.E131Q		0.3443804	455	239	SNV
YHIM-04	Non-synergy	ZNF746	chr7	149171908	149171919	CCAC	-	exonic	nonframeshift deletion	NM_001163474	7	c.1494_1505del	p.498_502del					Indel
YHIM-04	Non-synergy	ZNF775	chr7	150093847	150093847	C	G	exonic	nonsynonymous SNV	NM_173680	3	c.C278G	p.S93C		0.51709402	113	121	SNV
YHIM-04	Non-synergy	ZNF804B	chr7	88962750	88962750	C	A	exonic	nonsynonymous SNV	NM_181646	4	c.C454A	p.P152T		0.4738806	141	127	SNV
YHIM-04	Non-synergy	ZNF844	chr19	12187606	12187606	A	G	exonic	synonymous SNV	NM_001136501	4	c.A1671G	p.R557R		0.57	43	57	SNV
YHIM-04	Non-synergy	ZSWIM2	chr2	187693485	187693485	G	C	exonic	nonsynonymous SNV	NM_182521	9	c.C1128G	p.F376L		0.46511628	46	40	SNV
YHIM-04	Non-synergy	ZXDA	chrX	57934489	57934489	A	G	exonic	nonsynonymous SNV	NM_007156	1	c.T2366C	p.L789P		0.47318612	167	150	SNV
YHIM-04	Non-synergy	DAB2IP	chr9	124525783	124525783	G	C	splicing	splicing	NM_032552	7	c.1087-1G>C			0.51028807	119	124	SNV
YHIM-04	Non-synergy	EGFLAM	chr5	38258954	38258954	G	C	splicing	splicing	NM_152403	1	c.97+1G>C			0.49074074	55	53	SNV
YHIM-04	Non-synergy	NECAP1	chr12	8248196	8248196	G	C	splicing	splicing	NM_015509	7	c.677-1G>C			0.08805031	145	14	SNV
YHIM-04	Non-synergy	SLC16A12	chr10	91200864	91200864	C	G	splicing	splicing	NM_213606	5	c.448+1G>C			0.55629139	67	84	SNV
YHIM-04	Non-synergy	UNC79	chr14	94112953	94112953	G	C	splicing	splicing	NM_020818	36	c.5631-1G>C			1	0	98	SNV
YHIM-05	Synergy	ABI2	chr2	204291967	204291967	T	A	exonic	synonymous SNV	NM_001282927	9	c.T1098A	p.I366I		0.11191336	246	31	SNV
YHIM-05	Synergy	ABI2	chr2	204276030	204276030	C	T	exonic	synonymous SNV	NM_005759	8	c.C1014T	p.P338P		0.03875969	124	5	SNV
YHIM-05	Synergy	ABR	chr17	973329	973329	G	A	exonic	nonsynonymous SNV	NM_001282149	4	c.C242T	p.T81M		0.515625	31	33	SNV
YHIM-05	Synergy	ABTB2	chr11	34378912	34378912	C	T	exonic	synonymous SNV	NM_145804	1	c.G219A	p.T73T		0.08189655	213	19	SNV
YHIM-05	Synergy	ACIN1	chr14	23564274	23564274	C	A	exonic	synonymous SNV	NM_001164814	1	c.G222T	p.A74A		0.19047619	136	32	SNV
YHIM-05	Synergy	ADAMTS10	chr19	8651549	8651549	G	A	exonic	nonsynonymous SNV	NM_001282352	7	c.C757T	p.P253S		0.48684211	39	37	SNV
YHIM-05	Synergy	ADGRB3	chr6	69640509	69640509	A	G	exonic	synonymous SNV	NM_001704	4	c.A816G	p.E272E		0.21212121	130	35	SNV
YHIM-05	Synergy	AEBP2	chr12	19615455	19615455	C	T	exonic	nonsynonymous SNV	NM_001114176	2	c.C683T	p.T228I		0.52763819	94	105	SNV
YHIM-05	Synergy	AHNAK	chr11	62295770	62295770	T	C	exonic	nonsynonymous SNV	NM_001620	5	c.A6119G	p.H2040R		0.10497238	162	19	SNV
YHIM-05	Synergy	AHNAK	chr11	62295116	62295116	C	G	exonic	nonsynonymous SNV	NM_001620	5	c.G6773C	p.G2258A		0.05072464	131	7	SNV
YHIM-05	Synergy	AHNAK	chr11	62295075	62295075	C	T	exonic	nonsynonymous SNV	NM_001620	5	c.G6814A	p.V2272I		0.04964539	134	7	SNV
YHIM-05	Synergy	AHNAK	chr11	62295772	62295772	A	C	exonic	synonymous SNV	NM_001620	5	c.T6117G	p.V2039V		0.09497207	162	17	SNV
YHIM-05	Synergy	ANKRD17	chr4	74005979	74005979	C	T	exonic	nonsynonymous SNV	NM_001286771	15	c.G2015A	p.S672N		0.23728814	45	14	SNV
YHIM-05	Synergy	ANKRD17	chr4	74088709	74088709	A	C	exonic	synonymous SNV	NM_001286771	1	c.T36G	p.V12V		0.15163934	207	37	SNV
YHIM-05	Synergy	ANKRD17	chr4	73957144	73957144	A	G	exonic	synonymous SNV	NM_198889	28	c.T5448C	p.S1816S		0.09356725	155	16	SNV
YHIM-05	Synergy	ANKS1B	chr12	99222973	99222973	T	C	exonic	synonymous SNV	NM_001204065	3	c.A63G	p.K21K		0.35772358	79	44	SNV
YHIM-05	Synergy	AP4E1	chr15	51207682	51207682	G	C	exonic	nonsynonymous SNV	NM_001252127	3	c.G35C	p.C12S		0.2027027	59	15	SNV
YHIM-05	Synergy	APP	chr21	27394280	27394280	G	A	exonic	synonymous SNV	NM_001136129	5	c.C573T	p.D191D		0.671875	21	43	SNV
YHIM-05	Synergy	ARHGAP26	chr5	142586795	142586795	C	T	exonic	nonsynonymous SNV	NM_001135608	21	c.C2021T	p.S674L	CCG	0.2948718	110	46	SNV
YHIM-05	Synergy	ARHGEF10L	chr1	17991030	17991030	C	T	exonic	synonymous SNV	NM_001011722	24	c.C2832T	p.A944A		0.61016949	23	36	SNV
YHIM-05	Synergy	ARIH1	chr15	72873236	72873236	G	A	exonic	synonymous SNV	NM_005744	12	c.G1380A	p.K460K		0.06735751	180	13	SNV
YHIM-05	Synergy	ARMCX5-GP	chrX	101971698	101971698	T	C	exonic	nonsynonymous SNV	NM_001184875	4	c.T1901C	p.I634T		0.58536585	17	24	SNV
YHIM-05	Synergy	ATP5E	chr20	57605390	57605390	C	T	exonic	nonsynonymous SNV	NM_006886	2	c.G127A	p.V43I		0.26436782	128	46	SNV
YHIM-05	Synergy	ATXN7L3	chr17	42273183	42273183	A	G	exonic	synonymous SNV	NM_001098833	7	c.T537C	p.N179N		0.16438356	61	12	SNV
YHIM-05	Synergy	AZIN1	chr8	103841565	103841565	A	G	exonic	synonymous SNV	NM_148174	11	c.T1170C	p.H390H		0.42465753	126	93	SNV
YHIM-05	Synergy	BAAT	chr9	104133272	104133272	G	A	exonic	stopgain	NM_001127610	2	c.C415T	p.R139X		0.27272727	48	18	SNV
YHIM-05	Synergy	BCL11A	chr2	60689444	60689444	G	A	exonic	synonymous SNV	NM_018014	4	c.C603T	p.S201S	CCG	0.17040359	185	38	SNV
YHIM-05	Synergy	BCL9	chr1	147084783	147084783	A	C	exonic	nonsynonymous SNV	NM_004326	5	c.A155C	p.Q52P	CCG	0.1641791	56	11	SNV
YHIM-05	Synergy	BCL9	chr1	147084940	147084940	C	G	exonic	synonymous SNV	NM_004326	5	c.C312G	p.S104S	CCG	0.13402062	84	13	SNV
YHIM-05	Synergy	BRINP3	chr1	190067973	190067973	C	T	exonic	synonymous SNV	NM_001317188	7	c.G1170A	p.L390L		0.46992481	141	125	SNV

YHIM-05	Synergy	C12orf56	chr12	64784299	64784299	C	T	exonic	nonsynonymous SNV	NM_001099676	1	c.G47A	p.R16H		0.2295082	47	14	SNV
YHIM-05	Synergy	C1orf87	chr1	60456396	60456396	C	T	exonic	synonymous SNV	NM_152377	12	c.G1590A	p.S530S		0.35	26	14	SNV
YHIM-05	Synergy	C6orf118	chr6	165715503	165715503	G	A	exonic	nonsynonymous SNV	NM_144980	2	c.C308T	p.A103V		1	0	91	SNV
YHIM-05	Synergy	C6orf229	chr6	24798860	24798860	C	T	exonic	nonsynonymous SNV	NM_001282492	1	c.G226A	p.A76T		0.44594595	82	66	SNV
YHIM-05	Synergy	C6orf62	chr6	24718792	24718792	A	G	exonic	synonymous SNV	NM_030939	1	c.T105C	p.Y35Y		0.416	73	52	SNV
YHIM-05	Synergy	C8orf34	chr8	69434113	69434113	A	G	exonic	nonsynonymous SNV	NM_001195639	6	c.A845G	p.D282G		0.53608247	90	104	SNV
YHIM-05	Synergy	CA1	chr8	86250607	86250607	T	A	exonic	nonsynonymous SNV	NM_001164830	2	c.A109T	p.S37C		0.60992908	110	172	SNV
YHIM-05	Synergy	CABP7	chr22	30116500	30116500	C	T	exonic	synonymous SNV	NM_182527	1	c.C87T	p.D29D		0.42105263	33	24	SNV
YHIM-05	Synergy	CABP7	chr22	30116506	30116506	G	T	exonic	synonymous SNV	NM_182527	1	c.G93T	p.P31P		0.36	32	18	SNV
YHIM-05	Synergy	CACNG8	chr19	54485660	54485660	-	GAG	exonic	nonframeshift insertion	NM_031895	4	c.835_836insGA[illegible]	p.R279delinsRG					Indel
YHIM-05	Synergy	CACNG8	chr19	54485566	54485566	G	C	exonic	synonymous SNV	NM_031895	4	c.G741C	p.G247G		0.35	26	14	SNV
YHIM-05	Synergy	CACNG8	chr19	54485653	54485653	T	C	exonic	synonymous SNV	NM_031895	4	c.T828C	p.S276S		0.66666667	2	4	SNV
YHIM-05	Synergy	CALU	chr7	128394676	128394676	A	T	exonic	synonymous SNV	NM_001130674	3	c.A315T	p.I105I		0.18120805	122	27	SNV
YHIM-05	Synergy	CASK	chrX	41712459	41712459	T	C	exonic	synonymous SNV	NM_001126054	2	c.A81G	p.R27R		0.26612903	91	33	SNV
YHIM-05	Synergy	CASP8	chr2	202131411	202131411	C	T	exonic	stopgain	NM_001080125	2	c.C379T	p.R127X	CCG	0.51807229	40	43	SNV
YHIM-05	Synergy	CBLN2	chr18	70205516	70205516	C	A	exonic	synonymous SNV	NM_182511	5	c.G570T	p.L190L		0.61904762	16	26	SNV
YHIM-05	Synergy	CCDC169-S[illegible]	chr13	36776120	36776120	C	T	exonic	synonymous SNV	NM_001282147	2	c.G159A	p.T53T		0.44134078	100	79	SNV
YHIM-05	Synergy	CCDC40	chr17	78064092	78064092	G	A	exonic	nonsynonymous SNV	NM_001243342	18	c.G2987A	p.R996H		0.10769231	174	21	SNV
YHIM-05	Synergy	CCNB2	chr15	59409123	59409123	G	A	exonic	nonsynonymous SNV	NM_004701	6	c.G832A	p.E278K		0.42857143	36	27	SNV
YHIM-05	Synergy	CD58	chr1	117078587	117078587	C	T	exonic	nonsynonymous SNV	NM_001144822	3	c.G628A	p.G210S		0.45588235	37	31	SNV
YHIM-05	Synergy	CDC27	chr17	45234725	45234725	T	C	exonic	synonymous SNV	NM_001293091	5	c.A318G	p.T106T		0.14285714	36	6	SNV
YHIM-05	Synergy	CDH19	chr18	64239346	64239346	C	T	exonic	synonymous SNV	NM_001271028	2	c.G96A	p.K32K		0.25210084	89	30	SNV
YHIM-05	Synergy	CDH2	chr18	25591876	25591876	G	A	exonic	synonymous SNV	NM_001308176	3	c.C387T	p.D129D		0.52777778	68	76	SNV
YHIM-05	Synergy	CDH8	chr16	61687851	61687851	A	G	exonic	synonymous SNV	NM_001796	12	c.T2061C	p.N687N		0.23282443	201	61	SNV
YHIM-05	Synergy	CELF4	chr18	34839207	34839207	A	G	exonic	nonsynonymous SNV	NM_001025087	11	c.T1267C	p.F423L		0.46017699	61	52	SNV
YHIM-05	Synergy	CILP	chr15	65490828	65490828	G	A	exonic	nonsynonymous SNV	NM_003613	9	c.C1796T	p.A599V		0.525	38	42	SNV
YHIM-05	Synergy	CNOT11	chr2	101869651	101869651	A	C	exonic	synonymous SNV	NM_017546	1	c.A225C	p.I75I		0.05263158	72	4	SNV
YHIM-05	Synergy	CNOT6L	chr4	78641795	78641795	G	A	exonic	synonymous SNV	NM_001286790	12	c.C1458T	p.G486G		0.26966292	65	24	SNV
YHIM-05	Synergy	COL5A1	chr9	137704332	137704332	G	A	exonic	nonsynonymous SNV	NM_000093	47	c.G3728A	p.G1243E		0.03370787	172	6	SNV
YHIM-05	Synergy	COL9A3	chr20	61453154	61453154	G	A	exonic	nonsynonymous SNV	NM_001853	8	c.G415A	p.G139S		0.5	8	8	SNV
YHIM-05	Synergy	CPPED1	chr16	12798895	12798895	G	A	exonic	nonsynonymous SNV	NM_018340	3	c.C301T	p.R101W		0.46511628	23	20	SNV
YHIM-05	Synergy	CPS1	chr2	211471635	211471635	G	A	exonic	nonsynonymous SNV	NM_001122634	8	c.G809A	p.R270Q		0.49019608	26	25	SNV
YHIM-05	Synergy	CPSF4	chr7	99051670	99051670	A	G	exonic	nonsynonymous SNV	NM_001081559	7	c.A577G	p.T193A		0.22591362	233	68	SNV
YHIM-05	Synergy	CSMD3	chr8	113702215	113702215	T	A	exonic	synonymous SNV	NM_052900	13	c.A1725T	p.G575G		0.04444444	86	4	SNV
YHIM-05	Synergy	DAB2IP	chr9	124534970	124534970	C	T	exonic	synonymous SNV	NM_138709	10	c.C1791T	p.P597P		0.0754717	196	16	SNV
YHIM-05	Synergy	DARS	chr2	136718977	136718977	T	C	exonic	synonymous SNV	NM_001293312	3	c.A9G	p.K3K		0.21804511	312	87	SNV
YHIM-05	Synergy	DARS	chr2	136718971	136718971	A	T	exonic	synonymous SNV	NM_001293312	3	c.T15A	p.A5A		0.47368421	210	189	SNV
YHIM-05	Synergy	DENND1A	chr9	126554884	126554884	C	T	exonic	synonymous SNV	NM_020946	3	c.G114A	p.P38P		0.24401914	158	51	SNV
YHIM-05	Synergy	DLX1	chr2	172950459	172950459	G	T	exonic	synonymous SNV	NM_001038493	1	c.G54T	p.A18A		0.03680982	314	12	SNV
YHIM-05	Synergy	DMAP1	chr1	44680570	44680570	G	A	exonic	synonymous SNV	NM_019100	3	c.G393A	p.K131K		0.46296296	145	125	SNV
YHIM-05	Synergy	DNAH5	chr5	13862766	13862766	C	T	exonic	nonsynonymous SNV	NM_001369	29	c.G4687A	p.G1563S		0.69724771	33	76	SNV
YHIM-05	Synergy	DNAJB5	chr9	34996431	34996431	T	C	exonic	synonymous SNV	NM_001135005	3	c.T597C	p.D199D		0.03488372	581	21	SNV
YHIM-05	Synergy	DNAJC8	chr1	28531787	28531787	G	C	exonic	nonsynonymous SNV	NM_014280	7	c.C545G	p.A182G		0.8	1	4	SNV
YHIM-05	Synergy	DOCK11	chrX	117700570	117700570	A	G	exonic	nonsynonymous SNV	NM_144658	9	c.A905G	p.N302S		0.375	40	24	SNV
YHIM-05	Synergy	DPF1	chr19	38713285	38713285	G	A	exonic	synonymous SNV	NM_001135155	2	c.C174T	p.C58C		0.06	47	3	SNV
YHIM-05	Synergy	EBF3	chr10	131761931	131761931	C	T	exonic	synonymous SNV	NM_001005463	1	c.G102A	p.A34A		0.15789474	64	12	SNV
YHIM-05	Synergy	EBF3	chr10	131638582	131638582	G	A	exonic	synonymous SNV	NM_001005463	15	c.C1551T	p.A517A		0.3452381	55	29	SNV
YHIM-05	Synergy	EBF3	chr10	131638579	131638579	G	A	exonic	synonymous SNV	NM_001005463	15	c.C1554T	p.F518F		0.32	51	24	SNV
YHIM-05	Synergy	EFCAB7	chr1	63997594	63997594	G	C	exonic	nonsynonymous SNV	NM_032437	3	c.G291C	p.R97S		0.1971831	57	14	SNV
YHIM-05	Synergy	EFCAB7	chr1	63997564	63997564	G	A	exonic	synonymous SNV	NM_032437	3	c.G261A	p.L87L		0.25203252	92	31	SNV
YHIM-05	Synergy	EGF	chr4	110883055	110883055	G	A	exonic	nonsynonymous SNV	NM_001178131	7	c.G1100A	p.S367N		0.60180996	88	133	SNV
YHIM-05	Synergy	EHBP1	chr2	63223882	63223882	T	C	exonic	synonymous SNV	NM_001142614	19	c.T3192C	p.L1064L		0.22608696	89	26	SNV
YHIM-05	Synergy	EP400	chr12	132547090	132547090	A	G	exonic	synonymous SNV	NM_015409	47	c.A8178G	p.Q2726Q		0.17142857	29	6	SNV
YHIM-05	Synergy	EXT1	chr8	119123210	119123210	A	C	exonic	nonsynonymous SNV	NM_000127	1	c.T76G	p.L26V	CCG	0.24561404	43	14	SNV
YHIM-05	Synergy	EXT1	chr8	119123217	119123217	G	A	exonic	synonymous SNV	NM_000127	1	c.C69T	p.F23F	CCG	0.29508197	43	18	SNV
YHIM-05	Synergy	FA2H	chr16	74752984	74752984	C	T	exonic	nonsynonymous SNV	NM_024306	5	c.G688A	p.E230K		0.46923077	69	61	SNV
YHIM-05	Synergy	FAM120A	chr9	96294642	96294642	T	C	exonic	nonsynonymous SNV	NM_001286723	10	c.T1940C	p.F647S		0.34375	63	33	SNV
YHIM-05	Synergy	FAM134A	chr2	220044855	220044855	G	A	exonic	nonsynonymous SNV	NM_024293	4	c.G443A	p.R148Q		0.15873016	53	10	SNV
YHIM-05	Synergy	FAM13C	chr10	61028447	61028447	G	A	exonic	nonsynonymous SNV	NM_001001971	8	c.C808T	p.R270W		0.88888889	1	8	SNV
YHIM-05	Synergy	FAM155A	chr13	108518727	108518727	T	C	exonic	nonsynonymous SNV	NM_001080396	1	c.A218G	p.Q73R		0.14035088	49	8	SNV
YHIM-05	Synergy	FAM155A	chr13	108518525	108518525	G	A	exonic	synonymous SNV	NM_001080396	1	c.C420T	p.G140G		0.64516129	11	20	SNV
YHIM-05	Synergy	FAM193B	chr5	176966065	176966065	A	T	exonic	synonymous SNV	NM_001190946	2	c.T294A	p.S98S		0.06422018	306	21	SNV

YHIM-05	Synergy	FAM98A	chr2	33813408	33813408	T	C	exonic	synonymous SNV	NM_015475	4	c.A516G	p.E172E		0.39259259	82	53	SNV
YHIM-05	Synergy	FANCL	chr2	58390196	58390196	C	T	exonic	synonymous SNV	NM_001114636	10	c.G804A	p.L268L		0.3125	11	5	SNV
YHIM-05	Synergy	FAR1	chr11	13736138	13736138	T	C	exonic	synonymous SNV	NM_032228	9	c.T1038C	p.H346H		0.48818898	65	62	SNV
YHIM-05	Synergy	FBN2	chr5	127673750	127673750	G	T	exonic	synonymous SNV	NM_001999	27	c.C3537A	p.G1179G		0.08333333	132	12	SNV
YHIM-05	Synergy	FBRS	chr16	30675684	30675684	T	C	exonic	synonymous SNV	NM_001105079	7	c.T1204C	p.L402L		0.45714286	19	16	SNV
YHIM-05	Synergy	FBXO22	chr15	76209668	76209668	A	T	exonic	synonymous SNV	NM_012170	5	c.A561T	p.I187I		0.24137931	22	7	SNV
YHIM-05	Synergy	FBXO22	chr15	76209671	76209671	A	G	exonic	synonymous SNV	NM_012170	5	c.A564G	p.Q188Q		0.22222222	21	6	SNV
YHIM-05	Synergy	FGFBP2	chr4	15964409	15964409	G	A	exonic	nonsynonymous SNV	NM_031950	1	c.C344T	p.S115F		0.2739726	53	20	SNV
YHIM-05	Synergy	FHL1	chrX	135290664	135290664	T	G	exonic	nonsynonymous SNV	NM_001159699	5	c.T600G	p.D200E		0.08695652	126	12	SNV
YHIM-05	Synergy	FILIP1L	chr3	99567229	99567229	G	A	exonic	synonymous SNV	NM_001282794	2	c.C2571T	p.N857N		0.090301	272	27	SNV
YHIM-05	Synergy	FOXB2	chr9	79634834	79634834	G	A	exonic	synonymous SNV	NM_001013735	1	c.G264A	p.A88A		0.38857143	107	68	SNV
YHIM-05	Synergy	FYN	chr6	112021464	112021464	A	G	exonic	synonymous SNV	NM_002037	9	c.T705C	p.A235A		0.32467533	52	25	SNV
YHIM-05	Synergy	FZD2	chr17	42635110	42635110	C	G	exonic	synonymous SNV	NM_001466	1	c.C54G	p.P18P		0.23529412	13	4	SNV
YHIM-05	Synergy	GNAI3	chr1	110125065	110125065	A	C	exonic	synonymous SNV	NM_006496	5	c.A468C	p.L156L		0.49418605	87	85	SNV
YHIM-05	Synergy	GPAM	chr10	113923558	113923558	C	G	exonic	nonsynonymous SNV	NM_001244949	14	c.G1324C	p.D442H		0.50205761	121	122	SNV
YHIM-05	Synergy	GRID2	chr4	94693328	94693328	T	C	exonic	synonymous SNV	NM_001286838	15	c.T2418C	p.D806D		0.08695652	378	36	SNV
YHIM-05	Synergy	GRIN2B	chr12	13716877	13716877	G	A	exonic	nonsynonymous SNV	NM_000834	13	c.C3295T	p.R1099C		0.12359551	234	33	SNV
YHIM-05	Synergy	GRK4	chr4	3009529	3009529	G	C	exonic	nonsynonymous SNV	NM_001004056	5	c.G414C	p.Q138H		0.45	22	18	SNV
YHIM-05	Synergy	HDAC8	chrX	71571605	71571605	T	C	exonic	synonymous SNV	NM_001166418	8	c.A816G	p.Q272Q		0.11827957	82	11	SNV
YHIM-05	Synergy	HELZ	chr17	65184619	65184619	T	G	exonic	synonymous SNV	NM_014877	12	c.A978C	p.P326P		0.10492505	418	49	SNV
YHIM-05	Synergy	HELZ	chr17	65184616	65184616	T	C	exonic	synonymous SNV	NM_014877	12	c.A981G	p.E327E		0.12970711	416	62	SNV
YHIM-05	Synergy	HERC1	chr15	63955270	63955270	C	T	exonic	synonymous SNV	NM_003922	44	c.G8814A	p.A2938A		0.10606061	177	21	SNV
YHIM-05	Synergy	HIST1H2AE	chr6	26217247	26217247	T	A	exonic	synonymous SNV	NM_021052	1	c.T45A	p.A15A		0.48484849	119	112	SNV
YHIM-05	Synergy	HIVEP3	chr1	41990515	41990515	C	T	exonic	synonymous SNV	NM_001127714	5	c.G5274A	p.E1758E		0.11940299	59	8	SNV
YHIM-05	Synergy	HNRNPH1	chr5	179044095	179044095	T	C	exonic	synonymous SNV	NM_001257293	10	c.A1074G	p.E358E		0.12690355	172	25	SNV
YHIM-05	Synergy	HNRNPH3	chr10	70098278	70098278	A	G	exonic	synonymous SNV	NM_012207	4	c.A270G	p.R90R		0.4375	27	21	SNV
YHIM-05	Synergy	HNRNPR	chr1	23637097	23637097	C	T	exonic	synonymous SNV	NM_001297621	9	c.G1272A	p.K424K		0.03856749	349	14	SNV
YHIM-05	Synergy	HOXA6	chr7	27187336	27187336	G	A	exonic	synonymous SNV	NM_024014	1	c.C33T	p.P11P		0.20408163	78	20	SNV
YHIM-05	Synergy	HOXA7	chr7	27196105	27196105	C	G	exonic	synonymous SNV	NM_006896	1	c.G60C	p.L20L		0.17567568	61	13	SNV
YHIM-05	Synergy	HOXC4	chr12	54448818	54448818	C	T	exonic	synonymous SNV	NM_153633	2	c.C624T	p.R208R		0.22065728	166	47	SNV
YHIM-05	Synergy	HOXC8	chr12	54403461	54403461	A	G	exonic	synonymous SNV	NM_022658	1	c.A393G	p.Q131Q		0.29078014	100	41	SNV
YHIM-05	Synergy	HOXD10	chr2	176983779	176983779	A	G	exonic	synonymous SNV	NM_002148	2	c.A843G	p.L281L		0.20952381	83	22	SNV
YHIM-05	Synergy	HOXD8	chr2	176995655	176995655	G	C	exonic	synonymous SNV	NM_001199746	1	c.G561C	p.P187P		0.33333333	78	39	SNV
YHIM-05	Synergy	HRAS	chr11	534288	534288	C	T	exonic	nonsynonymous SNV	NM_001130442	2	c.G35A	p.G12D	CCG	1	0	286	SNV
YHIM-05	Synergy	HTR1E	chr6	87725869	87725869	G	A	exonic	nonsynonymous SNV	NM_000865	2	c.G817A	p.D273N		0.1754386	94	20	SNV
YHIM-05	Synergy	IDE	chr10	94239175	94239175	T	C	exonic	synonymous SNV	NM_001165946	5	c.A78G	p.P26P		0.13333333	78	12	SNV
YHIM-05	Synergy	IDE	chr10	94239072	94239072	A	G	exonic	synonymous SNV	NM_001165946	5	c.T181C	p.L61L		0.09142857	159	16	SNV
YHIM-05	Synergy	IKZF4	chr12	56415283	56415283	T	C	exonic	synonymous SNV	NM_022465	1	c.T6C	p.H2H		0.07526882	86	7	SNV
YHIM-05	Synergy	IL1RN	chr2	113877704	113877704	G	T	exonic	nonsynonymous SNV	NM_173841	2	c.G72T	p.K24N		0.48837209	22	21	SNV
YHIM-05	Synergy	INO80D	chr2	206927659	206927659	T	G	exonic	synonymous SNV	NM_017759	3	c.A82C	p.R28R		0.21839081	136	38	SNV
YHIM-05	Synergy	INPP4A	chr2	99193477	99193477	G	A	exonic	nonsynonymous SNV	NM_001134225	24	c.G2657A	p.R886Q		0.46969697	70	62	SNV
YHIM-05	Synergy	ITGA9	chr3	37670769	37670769	C	T	exonic	nonsynonymous SNV	NM_002207	16	c.C1781T	p.P594L		0.56	11	14	SNV
YHIM-05	Synergy	ITPR2	chr12	26752258	26752258	C	T	exonic	nonsynonymous SNV	NM_002223	30	c.G3822A	p.M1274I		0.48927039	119	114	SNV
YHIM-05	Synergy	JRKL	chr11	96124845	96124845	G	T	exonic	nonsynonymous SNV	NM_003772	1	c.G1032T	p.Q344H		0.08888889	246	24	SNV
YHIM-05	Synergy	JRKL	chr11	96124893	96124893	A	G	exonic	synonymous SNV	NM_003772	1	c.A1080G	p.L360L		0.14414414	285	48	SNV
YHIM-05	Synergy	JRKL	chr11	96124851	96124851	C	T	exonic	synonymous SNV	NM_003772	1	c.C1038T	p.N346N		0.15050167	254	45	SNV
YHIM-05	Synergy	KAT6B	chr10	76732344	76732344	A	G	exonic	synonymous SNV	NM_001256468	7	c.A1008G	p.R336R	CCG	0.05825243	97	6	SNV
YHIM-05	Synergy	KAZN	chr1	14925619	14925619	-	GGA	exonic	nonframeshift insertion	NM_015209	1	c.126_127insGG	p.G42delinsGG					Indel
YHIM-05	Synergy	KAZN	chr1	14925611	14925611	C	T	exonic	synonymous SNV	NM_015209	1	c.C118T	p.L40L		0.78985507	29	109	SNV
YHIM-05	Synergy	KCMF1	chr2	85280388	85280388	A	G	exonic	synonymous SNV	NM_020122	7	c.A1002G	p.S334S		0.19205298	122	29	SNV
YHIM-05	Synergy	KCNN3	chr1	154841529	154841529	G	C	exonic	synonymous SNV	NM_001204087	1	c.C912G	p.L304L		0.36909871	147	86	SNV
YHIM-05	Synergy	KCNN3	chr1	154841532	154841532	C	T	exonic	synonymous SNV	NM_001204087	1	c.G909A	p.E303E		0.40160643	149	100	SNV
YHIM-05	Synergy	KCTD15	chr19	34292186	34292186	G	A	exonic	nonsynonymous SNV	NM_001129994	4	c.G181A	p.D61N		0.45614035	62	52	SNV
YHIM-05	Synergy	KDM2A	chr11	67018079	67018081	GAG	-	exonic	nonframeshift deletion	NM_001256405	6	c.1261_1263del	p.421_421del					Indel
YHIM-05	Synergy	KDM6A	chrX	44928940	44928940	C	T	exonic	synonymous SNV	NM_001291418	15	c.C1803T	p.N601N	CCG	0.07017544	159	12	SNV
YHIM-05	Synergy	KIF26A	chr14	104642264	104642264	G	A	exonic	nonsynonymous SNV	NM_015656	12	c.G3139A	p.G1047R		0.45098039	56	46	SNV
YHIM-05	Synergy	KIF4A	chrX	69573472	69573472	C	A	exonic	nonsynonymous SNV	NM_012310	15	c.C1489A	p.Q497K		0.3125	11	5	SNV
YHIM-05	Synergy	KIR2DL4	chr19	55324635	55324635	T	C	exonic	synonymous SNV	NM_001080772	6	c.T762C	p.F254F		0.04395604	87	4	SNV
YHIM-05	Synergy	KLHL14	chr18	30349700	30349700	T	G	exonic	synonymous SNV	NM_020805	2	c.A855C	p.R285R		0.16853933	74	15	SNV
YHIM-05	Synergy	KLHL41	chr2	170366493	170366493	G	C	exonic	nonsynonymous SNV	NM_006063	1	c.G205C	p.A69P		0.48611111	74	70	SNV
YHIM-05	Synergy	KMT2A	chr11	118307306	118307306	C	T	exonic	synonymous SNV	NM_001197104	1	c.C79T	p.L27L	CCG	1	0	2	SNV
YHIM-05	Synergy	KMT2C	chr7	152132806	152132806	A	G	exonic	synonymous SNV	NM_170606	1	c.T66C	p.P22P	CCG	0.39285714	17	11	SNV

YHIM-05	Synergy	LCORL	chr4	17885757	17885757	T	G	exonic	nonsynonymous SNV	NM_001166139	7	c.A1395C	p.E465D		0.1745283	175	37	SNV
YHIM-05	Synergy	LCORL	chr4	17885649	17885649	T	C	exonic	synonymous SNV	NM_001166139	7	c.A1503G	p.E501E		0.14516129	159	27	SNV
YHIM-05	Synergy	LDB2	chr4	16507594	16507594	C	G	exonic	synonymous SNV	NM_001130834	8	c.G903C	p.A301A		0.08	115	10	SNV
YHIM-05	Synergy	LHFPL3	chr7	103969380	103969380	C	T	exonic	synonymous SNV	NM_199000	1	c.C153T	p.N51N		0.24844721	242	80	SNV
YHIM-05	Synergy	LHX9	chr1	197898248	197898248	A	C	exonic	synonymous SNV	NM_020204	5	c.A1053C	p.G351G		0.04411765	260	12	SNV
YHIM-05	Synergy	LRRC57	chr15	42839520	42839520	C	T	exonic	nonsynonymous SNV	NM_153260	4	c.G431A	p.R144Q		0.53278689	57	65	SNV
YHIM-05	Synergy	LRRTM3	chr10	68686904	68686904	A	T	exonic	nonsynonymous SNV	NM_178011	2	c.A230T	p.K77M		0.51079137	68	71	SNV
YHIM-05	Synergy	LVRN	chr5	115336880	115336880	G	T	exonic	nonsynonymous SNV	NM_173800	10	c.G1764T	p.M588I		0.66285714	59	116	SNV
YHIM-05	Synergy	MADD	chr11	47345227	47345227	C	T	exonic	synonymous SNV	NM_001135943	28	c.C4074T	p.C1358C		0.23943662	54	17	SNV
YHIM-05	Synergy	MAF	chr16	79632822	79632822	T	C	exonic	synonymous SNV	NM_001031804	1	c.A978G	p.Q326Q	CCG	0.10441767	223	26	SNV
YHIM-05	Synergy	MAFB	chr20	39316687	39316687	A	G	exonic	synonymous SNV	NM_005461	1	c.T804C	p.N268N	CCG	0.13664596	139	22	SNV
YHIM-05	Synergy	MAGEB3	chrX	30254976	30254976	G	C	exonic	nonsynonymous SNV	NM_002365	5	c.G935C	p.R312T		0.46428571	45	39	SNV
YHIM-05	Synergy	MAGI1	chr3	65344811	65344811	A	T	exonic	nonsynonymous SNV	NM_015520	24	c.T3735A	p.N1245K		0.30645161	43	19	SNV
YHIM-05	Synergy	MAPK8IP1	chr11	45924091	45924091	G	A	exonic	nonsynonymous SNV	NM_005456	5	c.G773A	p.G258D		0.19230769	189	45	SNV
YHIM-05	Synergy	MATR3	chr5	138643914	138643914	A	G	exonic	synonymous SNV	NM_001194955	2	c.A810G	p.R270R		0.74074074	140	400	SNV
YHIM-05	Synergy	MATR3	chr5	138643569	138643569	C	A	exonic	synonymous SNV	NM_001194955	2	c.C465A	p.T155T		0.26086957	153	54	SNV
YHIM-05	Synergy	MATR3	chr5	138643173	138643173	G	A	exonic	synonymous SNV	NM_001194955	2	c.G69A	p.A23A		0.43044619	217	164	SNV
YHIM-05	Synergy	MBD5	chr2	149227320	149227320	G	A	exonic	nonsynonymous SNV	NM_018328	9	c.G1808A	p.S603N		0.1835443	129	29	SNV
YHIM-05	Synergy	MBP	chr18	74728826	74728826	C	T	exonic	nonsynonymous SNV	NM_001025081	1	c.G139A	p.G47S		0.15730337	75	14	SNV
YHIM-05	Synergy	MCM4	chr8	48882522	48882522	G	A	exonic	nonsynonymous SNV	NM_005914	10	c.G1339A	p.E447K		0.10368664	389	45	SNV
YHIM-05	Synergy	MCM4	chr8	48882419	48882419	C	T	exonic	synonymous SNV	NM_005914	10	c.C1236T	p.Y412Y		0.07054674	527	40	SNV
YHIM-05	Synergy	MDGA2	chr14	47343266	47343266	G	A	exonic	nonsynonymous SNV	NM_001113498	13	c.C2368T	p.R790C		0.43421053	43	33	SNV
YHIM-05	Synergy	MDN1	chr6	90390344	90390344	G	C	exonic	nonsynonymous SNV	NM_014611	74	c.C12229G	p.L4077V		0.46842105	101	89	SNV
YHIM-05	Synergy	MECOM	chr3	168833847	168833847	T	G	exonic	nonsynonymous SNV	NM_001105078	7	c.A1249C	p.I417L	CCG	0.16666667	215	43	SNV
YHIM-05	Synergy	MECOM	chr3	168833952	168833952	A	G	exonic	nonsynonymous SNV	NM_001105078	7	c.T1144C	p.F382L	CCG	0.12937063	249	37	SNV
YHIM-05	Synergy	MED1	chr17	37566896	37566896	G	A	exonic	synonymous SNV	NM_004774	17	c.C1578T	p.S526S		0.34965035	93	50	SNV
YHIM-05	Synergy	MEIS1	chr2	66739390	66739390	A	C	exonic	synonymous SNV	NM_002398	8	c.A852C	p.T284T		0.17857143	161	35	SNV
YHIM-05	Synergy	MGA	chr15	42028743	42028743	G	T	exonic	nonsynonymous SNV	NM_001080541	13	c.G4281T	p.E1427D		0.21052632	90	24	SNV
YHIM-05	Synergy	MPP3	chr17	41903134	41903134	C	T	exonic	nonsynonymous SNV	NM_001932	9	c.G599A	p.S200N		0.49615385	131	129	SNV
YHIM-05	Synergy	MTMR3	chr22	30418060	30418060	T	G	exonic	nonsynonymous SNV	NM_021090	18	c.T3268G	p.L1090V		0.33333333	124	62	SNV
YHIM-05	Synergy	MYH13	chr17	10248600	10248600	T	C	exonic	synonymous SNV	NM_003802	15	c.A1503G	p.E501E		0.06349206	59	4	SNV
YHIM-05	Synergy	NEIL1	chr15	75640284	75640284	C	T	exonic	synonymous SNV	NM_001256552	1	c.C153T	p.C51C		0.55172414	13	16	SNV
YHIM-05	Synergy	NEO1	chr15	73528708	73528708	C	T	exonic	synonymous SNV	NM_001172623	8	c.C1312T	p.L438L		0.17741936	255	55	SNV
YHIM-05	Synergy	NEUROD6	chr7	31378142	31378142	A	G	exonic	synonymous SNV	NM_022728	2	c.T741C	p.T247T		0.14857143	149	26	SNV
YHIM-05	Synergy	NEUROD6	chr7	31378037	31378037	A	G	exonic	synonymous SNV	NM_022728	2	c.T846C	p.G282G		0.23648649	113	35	SNV
YHIM-05	Synergy	NFIX	chr19	13135852	13135852	C	T	exonic	synonymous SNV	NM_001271043	2	c.C69T	p.F23F		0.19421488	195	47	SNV
YHIM-05	Synergy	NKX2-1	chr14	36988572	36988572	G	T	exonic	synonymous SNV	NM_001079668	2	c.C81A	p.R27R	CCG	0.48	52	48	SNV
YHIM-05	Synergy	NKX2-1	chr14	36988266	36988266	C	A	exonic	synonymous SNV	NM_003317	1	c.G297T	p.P99P	CCG	0.26373626	67	24	SNV
YHIM-05	Synergy	NKX2-1	chr14	36987191	36987191	G	A	exonic	synonymous SNV	NM_003317	2	c.C408T	p.N136N	CCG	0.06493507	72	5	SNV
YHIM-05	Synergy	NLGN2	chr17	7312024	7312024	C	T	exonic	synonymous SNV	NM_020795	1	c.C450T	p.T150T		0.0625	120	8	SNV
YHIM-05	Synergy	NLRP9	chr19	56243994	56243994	C	A	exonic	nonsynonymous SNV	NM_176820	2	c.G1203T	p.E401D		0.64102564	28	50	SNV
YHIM-05	Synergy	NOL4	chr18	31537341	31537341	T	G	exonic	synonymous SNV	NM_001198549	4	c.A522C	p.I174I		0.15957447	237	45	SNV
YHIM-05	Synergy	NOVA1	chr14	27064746	27064746	A	G	exonic	synonymous SNV	NM_002515	2	c.T150C	p.Y50Y		0.06818182	82	6	SNV
YHIM-05	Synergy	NOVA1	chr14	26917951	26917951	T	C	exonic	synonymous SNV	NM_006489	4	c.A666G	p.Q222Q		0.05405405	210	12	SNV
YHIM-05	Synergy	NPTN	chr15	73889631	73889631	G	A	exonic	synonymous SNV	NM_001161363	2	c.C171T	p.S57S		0.4193038	367	265	SNV
YHIM-05	Synergy	NR2E1	chr6	108501544	108501544	T	C	exonic	synonymous SNV	NM_001286102	6	c.T771C	p.D257D		0.25757576	98	34	SNV
YHIM-05	Synergy	NR2E1	chr6	108501547	108501547	T	G	exonic	synonymous SNV	NM_001286102	6	c.T774G	p.A258A		0.31972789	100	47	SNV
YHIM-05	Synergy	NR2F2	chr15	96875583	96875583	A	G	exonic	synonymous SNV	NM_021005	1	c.A249G	p.G83G		0.38420108	343	214	SNV
YHIM-05	Synergy	NR2F2	chr15	96875715	96875715	T	C	exonic	synonymous SNV	NM_021005	1	c.T381C	p.H127H		0.24618736	346	113	SNV
YHIM-05	Synergy	NR4A2	chr2	157186546	157186546	G	A	exonic	synonymous SNV	NM_006186	3	c.C153T	p.I51I		0.27868853	176	68	SNV
YHIM-05	Synergy	NRG2	chr5	139245201	139245201	A	G	exonic	synonymous SNV	NM_004883	5	c.T1122C	p.N374N		0.15662651	210	39	SNV
YHIM-05	Synergy	NRN1	chr6	6002669	6002669	C	T	exonic	synonymous SNV	NM_016588	2	c.G117A	p.S39S		0.02893891	302	9	SNV
YHIM-05	Synergy	NUMBL	chr19	41179419	41179419	G	A	exonic	nonsynonymous SNV	NM_001289979	7	c.C743T	p.A248V		0.671875	21	43	SNV
YHIM-05	Synergy	OR10G8	chr11	123901027	123901027	A	G	exonic	nonsynonymous SNV	NM_001004464	1	c.A698G	p.K233R		0.16197183	119	23	SNV
YHIM-05	Synergy	OR5AS1	chr11	55798515	55798515	G	T	exonic	nonsynonymous SNV	NM_001001921	1	c.G621T	p.Q207H		0.49238579	100	97	SNV
YHIM-05	Synergy	PABPC1	chr8	101733758	101733758	G	C	exonic	synonymous SNV	NM_002568	1	c.C54G	p.L18L		0.35555556	58	32	SNV
YHIM-05	Synergy	PABPC1	chr8	101733644	101733644	A	G	exonic	synonymous SNV	NM_002568	1	c.T168C	p.Y56Y		0.36144578	53	30	SNV
YHIM-05	Synergy	PAIP2	chr5	138700324	138700324	G	A	exonic	synonymous SNV	NM_001033112	3	c.G210A	p.E70E		0.17391304	266	56	SNV
YHIM-05	Synergy	PAK2	chr3	196529957	196529957	C	A	exonic	nonsynonymous SNV	NM_002577	4	c.C358A	p.P120T		0.93004115	17	226	SNV
YHIM-05	Synergy	PAX2	chr10	102509645	102509645	C	T	exonic	synonymous SNV	NM_000278	2	c.C186T	p.H62H		0.26829268	30	11	SNV
YHIM-05	Synergy	PAX6	chr11	31822288	31822288	G	T	exonic	synonymous SNV	NM_001310160	4	c.C66A	p.T22T		0.25	165	55	SNV
YHIM-05	Synergy	PCDHA3	chr5	140182165	140182165	G	A	exonic	synonymous SNV	NM_018906	1	c.G1383A	p.T461T		0.29292929	70	29	SNV

YHIM-05	Synergy	PIK3CA	chr3	178916944	178916946	AAG	-	exonic	nonframeshift deletion	NM_006218	2	c.331_333del	p.111_111del	CCG				Indel
YHIM-05	Synergy	PLA2G6	chr22	38528839	38528839	G	A	exonic	nonsynonymous SNV	NM_001004426	7	c.C1076T	p.S359L		0.3030303	23	10	SNV
YHIM-05	Synergy	PLEKHG4B	chr5	171473	171473	G	A	exonic	nonsynonymous SNV	NM_052909	14	c.G2896A	p.E966K		0.33980583	68	35	SNV
YHIM-05	Synergy	PNOC	chr8	28186740	28186740	G	A	exonic	synonymous SNV	NM_006228	2	c.G66A	p.Q22Q		0.61290323	60	95	SNV
YHIM-05	Synergy	POU3F2	chr6	99284027	99284027	C	T	exonic	synonymous SNV	NM_005604	1	c.C1278T	p.Y426Y		0.24444444	34	11	SNV
YHIM-05	Synergy	POU6F2	chr7	39125590	39125590	T	C	exonic	nonsynonymous SNV	NM_001166018	3	c.T149C	p.V50A		0.66666667	90	180	SNV
YHIM-05	Synergy	POU6F2	chr7	39125588	39125588	T	C	exonic	synonymous SNV	NM_001166018	3	c.T147C	p.P49P		0.67259787	92	189	SNV
YHIM-05	Synergy	PPFIA3	chr19	49631290	49631290	G	A	exonic	nonsynonymous SNV	NM_003660	2	c.G160A	p.A54T		0.45714286	57	48	SNV
YHIM-05	Synergy	PPM1E	chr17	56833454	56833454	-	CCGG	exonic	nonframeshift insertion	NM_014906	1	c.96_97insCCGC	p.E32delinsEPE					Indel
YHIM-05	Synergy	PPM1E	chr17	56833457	56833457	G	C	exonic	synonymous SNV	NM_014906	1	c.G99C	p.P33P		0.55555556	12	15	SNV
YHIM-05	Synergy	PPP1R9A	chr7	94539498	94539498	A	G	exonic	nonsynonymous SNV	NM_001166161	1	c.A73G	p.T25A		0.44444444	35	28	SNV
YHIM-05	Synergy	PPP3CB	chr10	75238290	75238290	G	A	exonic	synonymous SNV	NM_001142353	3	c.C378T	p.G126G		0.15753425	123	23	SNV
YHIM-05	Synergy	PPP3CB	chr10	75198093	75198093	A	G	exonic	synonymous SNV	NM_001142354	13	c.T1455C	p.P485P		0.04255319	180	8	SNV
YHIM-05	Synergy	PRMT8	chr12	3678693	3678693	G	A	exonic	synonymous SNV	NM_001256536	6	c.G648A	p.A216A		0.20560748	85	22	SNV
YHIM-05	Synergy	PUM1	chr1	31467969	31467969	A	T	exonic	synonymous SNV	NM_001020658	6	c.T819A	p.G273G		0.06772009	413	30	SNV
YHIM-05	Synergy	RBBP5	chr1	205064043	205064043	C	T	exonic	nonsynonymous SNV	NM_001193273	13	c.G1165A	p.A389T		0.37755102	61	37	SNV
YHIM-05	Synergy	RIMS2	chr8	104933928	104933928	G	T	exonic	nonsynonymous SNV	NM_001282881	8	c.G1677T	p.M559I		0.60363636	109	166	SNV
YHIM-05	Synergy	RNF11	chr1	51736958	51736958	T	G	exonic	synonymous SNV	NM_014372	3	c.T429G	p.V143V		0.26035503	125	44	SNV
YHIM-05	Synergy	RPGRIP1L	chr16	53726156	53726156	C	T	exonic	synonymous SNV	NM_001127897	4	c.G351A	p.E117E		0.08609272	138	13	SNV
YHIM-05	Synergy	RPRD1B	chr20	36676826	36676826	G	A	exonic	nonsynonymous SNV	NM_021215	3	c.G358A	p.E120K		0.27125506	180	67	SNV
YHIM-05	Synergy	SAMD4A	chr14	55203869	55203869	C	T	exonic	synonymous SNV	NM_015589	3	c.C843T	p.P281P		0.71688742	171	433	SNV
YHIM-05	Synergy	SCGN	chr6	25661872	25661872	G	A	exonic	synonymous SNV	NM_006998	3	c.G246A	p.E82E		0.47668394	101	92	SNV
YHIM-05	Synergy	SEC61A2	chr10	12197881	12197881	G	A	exonic	synonymous SNV	NM_001142628	6	c.G501A	p.E167E		0.11409396	132	17	SNV
YHIM-05	Synergy	SETD5	chr3	9476059	9476059	T	C	exonic	synonymous SNV	NM_001080517	5	c.T219C	p.P73P		0.31724138	99	46	SNV
YHIM-05	Synergy	SHANK2	chr11	70332469	70332469	G	A	exonic	nonsynonymous SNV	NM_133266	10	c.C2165T	p.S722L		0.48910412	211	202	SNV
YHIM-05	Synergy	SI	chr3	164741422	164741422	G	T	exonic	nonsynonymous SNV	NM_001041	26	c.C3035A	p.S1012Y		1	0	260	SNV
YHIM-05	Synergy	SIX4	chr14	61190147	61190147	G	A	exonic	synonymous SNV	NM_017420	1	c.C646T	p.L216L		0.32692308	105	51	SNV
YHIM-05	Synergy	SKIDA1	chr10	21806728	21806728	A	G	exonic	synonymous SNV	NM_207371	4	c.T24C	p.F8F		0.05479452	138	8	SNV
YHIM-05	Synergy	SLC38A10	chr17	79254455	79254455	C	T	exonic	nonsynonymous SNV	NM_001037984	6	c.G580A	p.V194I		0.54421769	67	80	SNV
YHIM-05	Synergy	SLC39A5	chr12	56628705	56628705	A	T	exonic	nonsynonymous SNV	NM_001135195	4	c.A569T	p.Y190F		0.40909091	52	36	SNV
YHIM-05	Synergy	SLC39A5	chr12	56629410	56629410	G	C	exonic	nonsynonymous SNV	NM_001135195	6	c.G871C	p.V291L		0.53932584	41	48	SNV
YHIM-05	Synergy	SLC9A8	chr20	48467301	48467301	T	-	exonic	frameshift deletion	NM_001260491	7	c.537delT	p.G179fs					Indel
YHIM-05	Synergy	SLF2	chr10	102672968	102672968	A	C	exonic	nonsynonymous SNV	NM_001136123	1	c.A101C	p.H34P		0.29365079	89	37	SNV
YHIM-05	Synergy	SLTM	chr15	59172206	59172206	G	A	exonic	stopgain	NM_001013843	21	c.C3043T	p.R1015X		0.47058824	54	48	SNV
YHIM-05	Synergy	SLTM	chr15	59224565	59224565	A	G	exonic	synonymous SNV	NM_001013843	2	c.T240C	p.T80T		0.06329114	74	5	SNV
YHIM-05	Synergy	SMAD7	chr18	46476198	46476198	G	A	exonic	synonymous SNV	NM_001190821	1	c.C597T	p.S199S		0.40839695	310	214	SNV
YHIM-05	Synergy	SMAD7	chr18	46476357	46476357	A	G	exonic	synonymous SNV	NM_001190821	1	c.T438C	p.P146P		0.69902913	62	144	SNV
YHIM-05	Synergy	SMARCA1	chrX	128582379	128582379	C	T	exonic	synonymous SNV	NM_001282875	23	c.G3036A	p.E1012E		0.125	35	5	SNV
YHIM-05	Synergy	SMARCB1	chr22	24134041	24134041	T	G	exonic	synonymous SNV	NM_001007468	2	c.T192G	p.V64V	CCG	0.0982659	156	17	SNV
YHIM-05	Synergy	SMARCC2	chr12	56558148	56558148	T	C	exonic	synonymous SNV	NM_003075	27	c.A3507G	p.Q1169Q		0.34117647	56	29	SNV
YHIM-05	Synergy	SNTB2	chr16	69221519	69221519	T	G	exonic	synonymous SNV	NM_006750	1	c.T450G	p.A150A		0.08823529	62	6	SNV
YHIM-05	Synergy	SOX11	chr2	5834158	5834158	C	T	exonic	synonymous SNV	NM_003108	1	c.C1305T	p.S435S		0.5	41	41	SNV
YHIM-05	Synergy	SP9	chr2	175201845	175201845	G	C	exonic	synonymous SNV	NM_001145250	2	c.G1032C	p.G344G		0.14285714	24	4	SNV
YHIM-05	Synergy	SP9	chr2	175201959	175201959	T	C	exonic	synonymous SNV	NM_001145250	2	c.T1146C	p.H382H		0.16513762	91	18	SNV
YHIM-05	Synergy	SPTBN4	chr19	41071595	41071595	C	G	exonic	nonsynonymous SNV	NM_020971	29	c.C6083G	p.T2028S		0.49019608	26	25	SNV
YHIM-05	Synergy	SRCIN1	chr17	36718273	36718273	G	A	exonic	nonsynonymous SNV	NM_025248	7	c.C1126T	p.P376S		0.47222222	19	17	SNV
YHIM-05	Synergy	SRL	chr16	4247828	4247828	C	T	exonic	synonymous SNV	NM_001098814	4	c.G348A	p.L116L		0.43243243	42	32	SNV
YHIM-05	Synergy	STRBP	chr9	125921478	125921478	T	C	exonic	synonymous SNV	NM_001171137	9	c.A690G	p.E230E		0.475	105	95	SNV
YHIM-05	Synergy	STRN4	chr19	47230730	47230730	G	A	exonic	synonymous SNV	NM_001039877	9	c.C1248T	p.N416N		0.07553957	257	21	SNV
YHIM-05	Synergy	SUMO2	chr17	73177233	73177233	C	T	exonic	synonymous SNV	NM_001005849	2	c.G72A	p.G24G		0.1372549	44	7	SNV
YHIM-05	Synergy	SYNRG	chr17	35913320	35913320	C	T	exonic	synonymous SNV	NM_001163547	12	c.G2022A	p.Q674Q		0.03463204	223	8	SNV
YHIM-05	Synergy	TBC1D8B	chrX	106065407	106065407	C	G	exonic	nonsynonymous SNV	NM_017752	4	c.C561G	p.F187L		0.08176101	146	13	SNV
YHIM-05	Synergy	TET1	chr10	70446399	70446399	G	A	exonic	nonsynonymous SNV	NM_030625	11	c.G5339A	p.R1780Q	CCG	0.55251142	98	121	SNV
YHIM-05	Synergy	TLE4	chr9	82188645	82188645	C	A	exonic	synonymous SNV	NM_001282748	2	c.C87A	p.S29S		0.03968254	121	5	SNV
YHIM-05	Synergy	TMEM151A	chr11	66062852	66062852	C	T	exonic	nonsynonymous SNV	NM_153266	2	c.C1135T	p.R379C		0.625	3	5	SNV
YHIM-05	Synergy	TMPRSS13	chr11	117789342	117789342	T	C	exonic	nonsynonymous SNV	NM_001077263	2	c.A233G	p.Q78R		0.57894737	8	11	SNV
YHIM-05	Synergy	TNRC18	chr7	5428905	5428905	G	A	exonic	nonsynonymous SNV	NM_001080495	5	c.C550T	p.R184W		0.5	2	2	SNV
YHIM-05	Synergy	TNRC6B	chr22	40661404	40661404	G	A	exonic	synonymous SNV	NM_001162501	5	c.G1170A	p.E390E		0.24137931	132	42	SNV
YHIM-05	Synergy	TP53BP1	chr15	43762144	43762144	G	C	exonic	stopgain	NM_001141979	11	c.C1301G	p.S434X		0.41463415	24	17	SNV
YHIM-05	Synergy	TRIM42	chr3	140401340	140401340	C	T	exonic	synonymous SNV	NM_152616	2	c.C378T	p.T126T		1	0	275	SNV
YHIM-05	Synergy	TRPS1	chr8	116426279	116426279	A	G	exonic	nonsynonymous SNV	NM_001282902	6	c.T3830C	p.V1277A		0.26630435	135	49	SNV
YHIM-05	Synergy	TRPS1	chr8	116426278	116426278	C	T	exonic	synonymous SNV	NM_001282902	6	c.G3831A	p.V1277V		0.27683616	128	49	SNV

YHIM-05	Synergy	UACA	chr15	70976663	70976663	A	G	exonic	nonsynonymous SNV	NM_001008224	8	c.T686C	p.I229T	0.47685185	113	103	SNV
YHIM-05	Synergy	USP34	chr2	61441453	61441453	G	C	exonic	synonymous SNV	NM_014709	68	c.C8424G	p.V2808V	0.18979592	397	93	SNV
YHIM-05	Synergy	USP5	chr12	6972432	6972432	A	T	exonic	synonymous SNV	NM_001098536	15	c.A1845T	p.P615P	0.27710843	120	46	SNV
YHIM-05	Synergy	VCX3A	chrX	6451798	6451798	T	C	exonic	synonymous SNV	NM_016379	3	c.A549G	p.L183L	0.10869565	41	5	SNV
YHIM-05	Synergy	VEZF1	chr17	56060095	56060095	G	A	exonic	synonymous SNV	NM_007146	2	c.C693T	p.P231P	0.07291667	267	21	SNV
YHIM-05	Synergy	VWC2L	chr2	215301443	215301443	G	A	exonic	nonsynonymous SNV	NM_001080500	3	c.G481A	p.V161I	0.10493827	145	17	SNV
YHIM-05	Synergy	WDR20	chr14	102606479	102606479	G	C	exonic	synonymous SNV	NM_001242414	1	c.G219C	p.L73L	0.31531532	76	35	SNV
YHIM-05	Synergy	WDR20	chr14	102675218	102675218	C	T	exonic	synonymous SNV	NM_001242416	2	c.C528T	p.C176C	0.23809524	160	50	SNV
YHIM-05	Synergy	WNK1	chr12	977142	977142	C	T	exonic	synonymous SNV	NM_001184985	9	c.C2250T	p.C750C	0.25974026	171	60	SNV
YHIM-05	Synergy	XPO4	chr13	21381617	21381617	T	C	exonic	nonsynonymous SNV	NM_022459	13	c.A1796G	p.N599S	0.38181818	34	21	SNV
YHIM-05	Synergy	ZBTB20	chr3	114070607	114070607	T	C	exonic	synonymous SNV	NM_001164342	4	c.A318G	p.V106V	0.11597374	404	53	SNV
YHIM-05	Synergy	ZFHX4	chr8	77776706	77776706	G	C	exonic	nonsynonymous SNV	NM_024721	11	c.G10756C	p.E3586Q	0.14388489	119	20	SNV
YHIM-05	Synergy	ZFP91	chr11	58384702	58384702	G	A	exonic	synonymous SNV	NM_001197051	11	c.G1233A	p.K411K	0.05128205	74	4	SNV
YHIM-05	Synergy	ZFYVE26	chr14	68264909	68264909	C	T	exonic	synonymous SNV	NM_015346	11	c.G2070A	p.R690R	0.44761905	58	47	SNV
YHIM-05	Synergy	ZFYVE9	chr1	52732439	52732439	T	C	exonic	synonymous SNV	NM_004799	6	c.T2391C	p.A797A	0.31862745	139	65	SNV
YHIM-05	Synergy	ZIC3	chrX	136652205	136652205	T	C	exonic	synonymous SNV	NM_003413	3	c.T1380C	p.P460P	0.19285714	113	27	SNV
YHIM-05	Synergy	ZIC5	chr13	100623371	100623371	C	T	exonic	nonsynonymous SNV	NM_033132	1	c.G559A	p.G187S	0.15384615	22	4	SNV
YHIM-05	Synergy	ZMYM4	chr1	35824589	35824589	A	C	exonic	nonsynonymous SNV	NM_005095	3	c.A149C	p.D50A	0.15185185	229	41	SNV
YHIM-05	Synergy	ZMYM4	chr1	35824696	35824696	G	A	exonic	nonsynonymous SNV	NM_005095	3	c.G256A	p.G86S	0.18390805	213	48	SNV
YHIM-05	Synergy	ZNF131	chr5	43161698	43161698	C	T	exonic	nonsynonymous SNV	NM_001297548	5	c.C719T	p.T240M	0.20487805	489	126	SNV
YHIM-05	Synergy	ZNF131	chr5	43161699	43161699	G	A	exonic	synonymous SNV	NM_001297548	5	c.G720A	p.T240T	0.21634615	489	135	SNV
YHIM-05	Synergy	ZNF131	chr5	43161813	43161813	G	A	exonic	synonymous SNV	NM_001297548	5	c.G834A	p.E278E	0.53263158	222	253	SNV
YHIM-05	Synergy	ZNF207	chr17	30693698	30693698	C	A	exonic	synonymous SNV	NM_003457	8	c.C795A	p.V265V	0.39130435	182	117	SNV
YHIM-05	Synergy	ZNF3	chr7	99668982	99668982	T	C	exonic	synonymous SNV	NM_001278291	4	c.A1017G	p.G339G	0.0877193	52	5	SNV
YHIM-05	Synergy	ZNF3	chr7	99669003	99669003	G	A	exonic	synonymous SNV	NM_001278291	4	c.C996T	p.Y332Y	0.12698413	55	8	SNV
YHIM-05	Synergy	ZNF326	chr1	90472996	90472996	G	C	exonic	nonsynonymous SNV	NM_182976	5	c.G302C	p.S101T	0.17441861	71	15	SNV
YHIM-05	Synergy	ZNF462	chr9	109691965	109691965	T	C	exonic	synonymous SNV	NM_021224	3	c.T5772C	p.R1924R	0.09195402	237	24	SNV
YHIM-05	Synergy	ZNF502	chr3	44762438	44762438	G	A	exonic	synonymous SNV	NM_001134441	3	c.G129A	p.K43K	0.42176871	85	62	SNV
YHIM-05	Synergy	ZNF677	chr19	53741341	53741341	T	A	exonic	nonsynonymous SNV	NM_182609	5	c.A639T	p.E213D	0.47058824	54	48	SNV
YHIM-05	Synergy	ZNF98	chr19	22575733	22575733	T	A	exonic	nonsynonymous SNV	NM_001098626	4	c.A304T	p.N102Y	0.59259259	22	32	SNV
YHIM-05	Synergy	CD58	chr1	117078586	117078586	C	T	splicing	splicing	NM_001779	3	c.628+1G>A		0.45588235	37	31	SNV
YHIM-05	Synergy	EP300	chr22	41568668	41568668	G	A	splicing	splicing	NM_001429	28	c.4617+1G>A		0.53846154	60	70	SNV
YHIM-05	Synergy	NUP188	chr9	131748959	131748959	T	C	splicing	splicing	NM_015354	21	c.2197+2T>C		0.30538922	116	51	SNV
YHIM-05	Synergy	SMARCA4	chr19	11129700	11129700	G	T	splicing	splicing	NM_001128844	18	c.2505+1G>T		0.64383562	26	47	SNV
YHIM-05	Synergy	TMEM171	chr5	72427363	72427363	A	C	splicing	splicing	NM_173490	4	c.783-2A>C		0.35443038	51	28	SNV
YHIM-06	Synergy	A3GALT2	chr1	33772530	33772530	C	T	exonic	nonsynonymous SNV	NM_001080438	5	c.G860A	p.R287H	0.34065934	120	62	SNV
YHIM-06	Synergy	ABCA2	chr9	139904788	139904788	G	A	exonic	synonymous SNV	NM_001606	41	c.C6210T	p.A2070A	0.5875	33	47	SNV
YHIM-06	Synergy	ABCA7	chr19	1046317	1046317	C	T	exonic	nonsynonymous SNV	NM_019112	13	c.C1534T	p.R512C	0.54362416	68	81	SNV
YHIM-06	Synergy	ACSM4	chr12	7477192	7477192	G	C	exonic	nonsynonymous SNV	NM_001080454	11	c.G1534C	p.E512Q	0.46511628	115	100	SNV
YHIM-06	Synergy	ACSM5	chr16	20422859	20422859	C	A	exonic	nonsynonymous SNV	NM_017888	2	c.C53A	p.A18E	0.34285714	46	24	SNV
YHIM-06	Synergy	ADAMTS5	chr21	28338024	28338024	G	A	exonic	synonymous SNV	NM_007038	1	c.C687T	p.S229S	0.31	69	31	SNV
YHIM-06	Synergy	ADAMTSL1	chr9	18721564	18721564	A	G	exonic	nonsynonymous SNV	NM_001040272	15	c.A1907G	p.N636S	0.20754717	42	11	SNV
YHIM-06	Synergy	ADCY5	chr3	123010005	123010005	G	A	exonic	synonymous SNV	NM_001199642	18	c.C2232T	p.V744V	0.67910448	43	91	SNV
YHIM-06	Synergy	ADCY8	chr8	131795955	131795955	T	G	exonic	nonsynonymous SNV	NM_001115	17	c.A3250C	p.N1084H	0.18382353	222	50	SNV
YHIM-06	Synergy	AGGF1	chr5	76342196	76342196	G	A	exonic	nonsynonymous SNV	NM_018046	6	c.G895A	p.A299T	0.375	10	6	SNV
YHIM-06	Synergy	AKAP8L	chr19	15512116	15512116	G	C	exonic	nonsynonymous SNV	NM_001291478	5	c.C478G	p.L160V	0.36309524	107	61	SNV
YHIM-06	Synergy	ANHX	chr12	133795544	133795544	T	C	exonic	nonsynonymous SNV	NM_001191054	9	c.A1067G	p.D356G	0.31606218	132	61	SNV
YHIM-06	Synergy	ANKRD30B	chr18	14852253	14852253	T	C	exonic	nonsynonymous SNV	NM_001145029	36	c.T3953C	p.V1318A	0.53125	105	119	SNV
YHIM-06	Synergy	ANO4	chr12	101490357	101490357	G	C	exonic	synonymous SNV	NM_001286616	18	c.G1782C	p.L594L	0.30049261	142	61	SNV
YHIM-06	Synergy	ANO7	chr2	242144370	242144370	C	T	exonic	nonsynonymous SNV	NM_001001891	10	c.C1142T	p.T381M	0.51612903	30	32	SNV
YHIM-06	Synergy	AP3B1	chr5	77452138	77452138	G	A	exonic	nonsynonymous SNV	NM_001271769	14	c.C1270T	p.P424S	0.6097561	32	50	SNV
YHIM-06	Synergy	APOB	chr2	21228119	21228119	C	A	exonic	nonsynonymous SNV	NM_000384	26	c.G11621T	p.G3874V	0.45833333	78	66	SNV
YHIM-06	Synergy	APOBEC3H	chr22	39497505	39497505	C	T	exonic	nonsynonymous SNV	NM_001166002	3	c.C414T	p.F138F	0.27272727	8	3	SNV
YHIM-06	Synergy	ATP10A	chr15	25925003	25925003	G	-	exonic	frameshift deletion	NM_024490	21	c.3985delC	p.R1329fs				Indel
YHIM-06	Synergy	AZGP1	chr7	99564751	99564751	C	T	exonic	nonsynonymous SNV	NM_001185	4	c.G772A	p.G258R	0.11330049	180	23	SNV
YHIM-06	Synergy	BCLAF1	chr6	136582552	136582552	G	C	exonic	nonsynonymous SNV	NM_001077440	11	c.C2455G	p.Q819E	0.07116788	509	39	SNV
YHIM-06	Synergy	BCORL1	chrX	129189904	129189904	C	T	exonic	synonymous SNV	NM_021946	12	c.C4929T	p.F1643F	0.82055215	117	535	SNV
YHIM-06	Synergy	BPI	chr20	36935984	36935984	C	T	exonic	nonsynonymous SNV	NM_001725	2	c.C158T	p.T53M	0.5	54	54	SNV
YHIM-06	Synergy	BTAF1	chr10	93722386	93722386	C	T	exonic	nonsynonymous SNV	NM_003972	12	c.C1355T	p.A452V	0.41428571	82	58	SNV
YHIM-06	Synergy	C6orf62	chr6	24718792	24718792	A	G	exonic	synonymous SNV	NM_030939	1	c.T105C	p.Y35Y	0.03181818	213	7	SNV
YHIM-06	Synergy	CACNB3	chr12	49217216	49217216	G	A	exonic	synonymous SNV	NM_000725	2	c.G135A	p.E45E	0.66666667	39	78	SNV
YHIM-06	Synergy	CAMK2N2	chr3	183977968	183977968	C	T	exonic	nonsynonymous SNV	NM_033259	2	c.G217A	p.E73K	0.2283737	223	66	SNV

YHIM-06	Synergy	CASK	chrX	41712459	41712459	T	C	exonic	synonymous SNV	NM_001126054	2	c.A81G	p.R27R		0.08474576	54	5	SNV
YHIM-06	Synergy	CCAR2	chr8	22473543	22473543	C	G	exonic	nonsynonymous SNV	NM_021174	14	c.C1627G	p.L543V		1	0	81	SNV
YHIM-06	Synergy	CCDC180	chr9	100124561	100124561	G	C	exonic	nonsynonymous SNV	NM_020893	28	c.G3872C	p.R1291P		0.0979021	129	14	SNV
YHIM-06	Synergy	CCDC88A	chr2	55582824	55582824	C	T	exonic	nonsynonymous SNV	NM_001135597	8	c.G691A	p.A231T		0.30837004	157	70	SNV
YHIM-06	Synergy	CD209	chr19	7810624	7810624	C	T	exonic	synonymous SNV	NM_001144894	2	c.G396A	p.L132L		0.38461539	8	5	SNV
YHIM-06	Synergy	CD300LG	chr17	41926209	41926209	C	T	exonic	synonymous SNV	NM_001168322	2	c.C327T	p.V109V		0.08	115	10	SNV
YHIM-06	Synergy	CDAN1	chr15	43028875	43028875	A	T	exonic	nonsynonymous SNV	NM_138477	2	c.T194A	p.L65H		0.33507853	127	64	SNV
YHIM-06	Synergy	CDC42BPG	chr11	64606663	64606663	G	A	exonic	nonsynonymous SNV	NM_017525	7	c.C718T	p.P240S		0.49019608	104	100	SNV
YHIM-06	Synergy	CDH24	chr14	23523799	23523799	C	T	exonic	nonsynonymous SNV	NM_022478	5	c.G700A	p.G234S		0.3956044	55	36	SNV
YHIM-06	Synergy	CDH4	chr20	60499482	60499482	C	T	exonic	synonymous SNV	NM_001252338	10	c.C1608T	p.S536S		0.51612903	180	192	SNV
YHIM-06	Synergy	CDH8	chr16	61687650	61687650	T	A	exonic	synonymous SNV	NM_001796	12	c.A2262T	p.S754S		0.03703704	130	5	SNV
YHIM-06	Synergy	CDKN2A	chr9	21971028	21971028	C	T	exonic	stopgain	NM_000077	2	c.G330A	p.W110X	CCG	1	0	43	SNV
YHIM-06	Synergy	CDRT15	chr17	14139702	14139702	G	A	exonic	nonsynonymous SNV	NM_001007530	2	c.C308T	p.A103V		0.36363636	21	12	SNV
YHIM-06	Synergy	CELF4	chr18	34839207	34839207	A	G	exonic	nonsynonymous SNV	NM_001025087	11	c.T1267C	p.F423L		0.06557377	57	4	SNV
YHIM-06	Synergy	CENPV	chr17	16256686	16256686	-	CGGA(	exonic	nonframeshift insertion	NM_181716	1	c.64_65insGGG(	p.A22delinsGASA					Indel
YHIM-06	Synergy	CEP250	chr20	34053895	34053895	C	A	exonic	nonsynonymous SNV	NM_007186	7	c.C358A	p.L120I		0.03267974	296	10	SNV
YHIM-06	Synergy	CEP295	chr11	93429951	93429951	G	A	exonic	nonsynonymous SNV	NM_033395	15	c.G1873A	p.D625N		0.64	63	112	SNV
YHIM-06	Synergy	CEP76	chr18	12695273	12695273	G	A	exonic	stopgain	NM_001271989	5	c.C559T	p.Q187X		0.5	23	23	SNV
YHIM-06	Synergy	CHID1	chr11	903725	903725	G	C	exonic	synonymous SNV	NM_001142676	3	c.C165G	p.L55L		0.4	3	2	SNV
YHIM-06	Synergy	CHUK	chr10	101964269	101964269	C	T	exonic	nonsynonymous SNV	NM_001278	13	c.G1501A	p.G501R		0.2917847	250	103	SNV
YHIM-06	Synergy	CLCA4	chr1	87045902	87045902	A	T	exonic	synonymous SNV	NM_012128	14	c.A2634T	p.T878T		0.08695652	147	14	SNV
YHIM-06	Synergy	CLPTM1L	chr5	1344525	1344525	C	T	exonic	synonymous SNV	NM_030782	2	c.G204A	p.E68E		0.55913979	164	208	SNV
YHIM-06	Synergy	CLTCL1	chr22	19223348	19223348	G	C	exonic	synonymous SNV	NM_001835	6	c.C840G	p.G280G	CCG	0.18566775	250	57	SNV
YHIM-06	Synergy	CNOT6L	chr4	78641795	78641795	G	A	exonic	synonymous SNV	NM_001286790	12	c.C1458T	p.G486G		0.0617284	76	5	SNV
YHIM-06	Synergy	CNR2	chr1	24201329	24201329	G	T	exonic	nonsynonymous SNV	NM_001841	2	c.C779A	p.P260Q		0.04780877	239	12	SNV
YHIM-06	Synergy	CNTN6	chr3	1262409	1262409	C	G	exonic	nonsynonymous SNV	NM_001289080	3	c.C94G	p.P32A		0.24170616	160	51	SNV
YHIM-06	Synergy	COL10A1	chr6	116441305	116441305	C	G	exonic	nonsynonymous SNV	NM_000493	3	c.G1974C	p.E658D		0.15053763	79	14	SNV
YHIM-06	Synergy	COL14A1	chr8	121256165	121256165	T	C	exonic	synonymous SNV	NM_021110	20	c.T2397C	p.L799L		0.17571885	258	55	SNV
YHIM-06	Synergy	COL5A2	chr2	189932832	189932832	G	A	exonic	nonsynonymous SNV	NM_000393	21	c.C1310T	p.P437L		0.53448276	27	31	SNV
YHIM-06	Synergy	CRHR2	chr7	30693204	30693204	C	T	exonic	nonsynonymous SNV	NM_001202482	12	c.G1105A	p.V369M		0.11403509	101	13	SNV
YHIM-06	Synergy	CRYBA4	chr22	27021563	27021563	T	G	exonic	nonsynonymous SNV	NM_001886	4	c.T277G	p.S93A		0.3	105	45	SNV
YHIM-06	Synergy	CTNNB1	chr3	41266630	41266630	C	G	exonic	nonsynonymous SNV	NM_001098209	4	c.C427G	p.Q143E	CCG	0.62425447	189	314	SNV
YHIM-06	Synergy	CYP11B2	chr8	143996209	143996209	G	A	exonic	synonymous SNV	NM_000498	4	c.C711T	p.L237L		0.16260163	206	40	SNV
YHIM-06	Synergy	DAB2IP	chr9	124544747	124544747	C	T	exonic	synonymous SNV	NM_138709	14	c.C3168T	p.N1056N		0.4691358	86	76	SNV
YHIM-06	Synergy	DARS	chr2	136718971	136718971	A	T	exonic	synonymous SNV	NM_001293312	3	c.T15A	p.A5A		0.09708738	186	20	SNV
YHIM-06	Synergy	DCC	chr18	50589754	50589754	T	C	exonic	synonymous SNV	NM_005215	6	c.T1065C	p.S355S		0.55102041	88	108	SNV
YHIM-06	Synergy	DCHS1	chr11	6653884	6653884	C	G	exonic	nonsynonymous SNV	NM_003737	6	c.G2859C	p.M953I		0.46875	17	15	SNV
YHIM-06	Synergy	DCSTAMP	chr8	105361485	105361485	G	T	exonic	nonsynonymous SNV	NM_001257317	2	c.G705T	p.L235F		0.32448378	229	110	SNV
YHIM-06	Synergy	DDIAS	chr11	82645045	82645045	C	A	exonic	nonsynonymous SNV	NM_145018	6	c.C2665A	p.L889I		0.25791855	164	57	SNV
YHIM-06	Synergy	DDR2	chr1	162724986	162724986	A	T	exonic	nonsynonymous SNV	NM_006182	6	c.A458T	p.K153M		0.36507937	40	23	SNV
YHIM-06	Synergy	DDX3X	chrX	41206135	41206135	G	A	exonic	nonsynonymous SNV	NM_001193417	14	c.G1591A	p.E531K		1	0	32	SNV
YHIM-06	Synergy	DEFB114	chr6	49931769	49931769	A	T	exonic	nonsynonymous SNV	NM_001037499	1	c.T50A	p.L17Q		0.11111111	160	20	SNV
YHIM-06	Synergy	DISC1	chr1	231906768	231906768	C	T	exonic	nonsynonymous SNV	NM_001164556	4	c.C536T	p.S179L		0.63265306	54	93	SNV
YHIM-06	Synergy	DNAH7	chr2	196756381	196756381	C	T	exonic	nonsynonymous SNV	NM_018897	31	c.G5044A	p.V1682M		0.4921466	97	94	SNV
YHIM-06	Synergy	DNMT3B	chr20	31390268	31390268	G	A	exonic	synonymous SNV	NM_001207056	17	c.G1935A	p.G645G		0.45454546	138	115	SNV
YHIM-06	Synergy	DRAM1	chr12	102301967	102301967	T	C	exonic	synonymous SNV	NM_018370	4	c.T346C	p.L116L		0.37209302	27	16	SNV
YHIM-06	Synergy	DSCAM	chr21	41385130	41385130	G	A	exonic	nonsynonymous SNV	NM_001271534	33	c.C5816T	p.T1939M		0.48453608	50	47	SNV
YHIM-06	Synergy	DUSP27	chr1	167095453	167095453	C	G	exonic	stopgain	NM_001080426	5	c.C1085G	p.S362X		0.37704918	76	46	SNV
YHIM-06	Synergy	EDAR	chr2	109546648	109546648	G	A	exonic	synonymous SNV	NM_022336	3	c.C102T	p.N34N		0.52336449	51	56	SNV
YHIM-06	Synergy	EEF1D	chr8	144671667	144671667	C	T	exonic	synonymous SNV	NM_001130053	3	c.G585A	p.G195G		0.31034483	160	72	SNV
YHIM-06	Synergy	EHD1	chr11	64645900	64645900	T	C	exonic	nonsynonymous SNV	NM_006795	1	c.A37G	p.K13E		0.54	46	54	SNV
YHIM-06	Synergy	EIF4G1	chr3	184049813	184049813	G	A	exonic	synonymous SNV	NM_004953	25	c.G3972A	p.Q1324Q		0.51893939	127	137	SNV
YHIM-06	Synergy	EIF4G1	chr3	184035162	184035162	G	A	exonic	synonymous SNV	NM_182917	4	c.G201A	p.Q67Q		0.51492537	65	69	SNV
YHIM-06	Synergy	EMC1	chr1	19570474	19570474	C	-	exonic	frameshift deletion	NM_001271427	3	c.256delG	p.A86fs					Indel
YHIM-06	Synergy	ENPEP	chr4	111427909	111427909	A	C	exonic	nonsynonymous SNV	NM_001977	4	c.A1035C	p.K345N		0.14285714	30	5	SNV
YHIM-06	Synergy	ENPP3	chr6	132061476	132061476	C	A	exonic	nonsynonymous SNV	NM_005021	24	c.C2413A	p.P805T		0.24170616	160	51	SNV
YHIM-06	Synergy	EPHA2	chr1	16464900	16464900	A	-	exonic	frameshift deletion	NM_004431	4	c.849delT	p.F283fs					Indel
YHIM-06	Synergy	ESR1	chr6	152201791	152201791	A	T	exonic	nonsynonymous SNV	NM_001291230	4	c.A651T	p.R217S	CCG	0.66911765	45	91	SNV
YHIM-06	Synergy	ESRRB	chr14	76957944	76957944	C	T	exonic	synonymous SNV	NM_004452	8	c.C942T	p.L314L		0.19444444	58	14	SNV
YHIM-06	Synergy	EVPL	chr17	74004317	74004317	G	A	exonic	nonsynonymous SNV	NM_001988	22	c.C4969T	p.R1657W		0.34262948	165	86	SNV
YHIM-06	Synergy	FAM131C	chr1	16385131	16385131	C	A	exonic	nonsynonymous SNV	NM_182623	7	c.G644T	p.S215I		0.41176471	20	14	SNV
YHIM-06	Synergy	FAM192A	chr16	57197923	57197923	G	A	exonic	synonymous SNV	NM_024946	6	c.C537T	p.D179D		0.30357143	156	68	SNV

YHIM-06	Synergy	FAM83H	chr8	144809205	144809205	G	C	exonic	nonsynonymous SNV	NM_198488	5	c.C2426G	p.P809R		0.17435897	161	34	SNV
YHIM-06	Synergy	FAT1	chr4	187521308	187521309	AT	-	exonic	frameshift deletion	NM_005245	22	c.11846_11847d	p.Y3949fs	CCG				Indel
YHIM-06	Synergy	FAT1	chr4	187530425	187530425	G	C	exonic	stopgain	NM_005245	16	c.C10118G	p.S3373X	CCG	0.46794872	83	73	SNV
YHIM-06	Synergy	FAT4	chr4	126370701	126370701	C	T	exonic	stopgain	NM_001291285	9	c.C8536T	p.R2846X	CCG	0.51587302	122	130	SNV
YHIM-06	Synergy	FBXO42	chr1	16632378	16632378	G	T	exonic	nonsynonymous SNV	NM_018994	3	c.C287A	p.A96D		0.10714286	75	9	SNV
YHIM-06	Synergy	FGF23	chr12	4479908	4479908	C	T	exonic	synonymous SNV	NM_020638	3	c.G357A	p.T119T		0.53787879	122	142	SNV
YHIM-06	Synergy	FGFBP3	chr10	93668364	93668364	C	G	exonic	synonymous SNV	NM_152429	2	c.G363C	p.R121R		0.72972973	10	27	SNV
YHIM-06	Synergy	FIGN	chr2	164467367	164467367	C	A	exonic	synonymous SNV	NM_018086	3	c.G975T	p.G325G		0.46209386	149	128	SNV
YHIM-06	Synergy	FOXD3	chr1	63789932	63789932	C	T	exonic	synonymous SNV	NM_012183	1	c.C1203T	p.A401A		0.7016129	37	87	SNV
YHIM-06	Synergy	FOXI2	chr10	129535619	129535619	G	C	exonic	nonsynonymous SNV	NM_207426	1	c.G82C	p.E28Q		0.23529412	13	4	SNV
YHIM-06	Synergy	FRY	chr13	32869449	32869449	T	C	exonic	nonsynonymous SNV	NM_023037	61	c.T8894C	p.L2965P		0.96946565	4	127	SNV
YHIM-06	Synergy	FSD2	chr15	83430889	83430889	G	C	exonic	synonymous SNV	NM_001281806	11	c.C1827G	p.L609L		0.34027778	95	49	SNV
YHIM-06	Synergy	GABRB3	chr15	26793233	26793233	C	T	exonic	nonsynonymous SNV	NM_001191320	7	c.G874A	p.E292K		0.27065527	256	95	SNV
YHIM-06	Synergy	GDF5	chr20	34025221	34025221	C	T	exonic	nonsynonymous SNV	NM_000557	1	c.G488A	p.R163H		0.18730159	256	59	SNV
YHIM-06	Synergy	GPC5	chr13	92346045	92346045	C	T	exonic	synonymous SNV	NM_004466	3	c.C930T	p.Y310Y		0.98795181	2	164	SNV
YHIM-06	Synergy	GRAMD4	chr22	47064594	47064594	C	T	exonic	synonymous SNV	NM_015124	11	c.C939T	p.F313F		1	0	170	SNV
YHIM-06	Synergy	GRID1	chr10	87487618	87487618	G	C	exonic	nonsynonymous SNV	NM_017551	10	c.C1527G	p.I509M		0.225	31	9	SNV
YHIM-06	Synergy	GSTT2,GSTT	chr22	24325091	24325091	G	T	exonic	synonymous SNV	NM_000854	4	c.G381T	p.V127V		0.54	46	54	SNV
YHIM-06	Synergy	HHATL	chr3	42739072	42739072	A	C	exonic	nonsynonymous SNV	NM_020707	7	c.T793G	p.F265V		0.15673981	269	50	SNV
YHIM-06	Synergy	HMCN1	chr1	185969290	185969290	C	A	exonic	nonsynonymous SNV	NM_031935	26	c.C3988A	p.P1330T		0.26190476	155	55	SNV
YHIM-06	Synergy	HMGCLL1	chr6	55406616	55406616	T	G	exonic	nonsynonymous SNV	NM_001042406	3	c.A208C	p.I70L		0.17948718	32	7	SNV
YHIM-06	Synergy	HOXB6	chr17	46675432	46675432	G	C	exonic	synonymous SNV	NM_018952	3	c.C81G	p.L27L		0.63366337	37	64	SNV
YHIM-06	Synergy	HOXC10	chr12	54379175	54379175	G	A	exonic	nonsynonymous SNV	NM_017409	1	c.G132A	p.M44I		0.33519553	119	60	SNV
YHIM-06	Synergy	HOXC10	chr12	54379717	54379717	G	A	exonic	nonsynonymous SNV	NM_017409	1	c.G674A	p.G225E		0.19318182	71	17	SNV
YHIM-06	Synergy	HOXC10	chr12	54379785	54379785	G	A	exonic	nonsynonymous SNV	NM_017409	1	c.G742A	p.E248K		0.29787234	33	14	SNV
YHIM-06	Synergy	HOXC10	chr12	54383085	54383085	G	A	exonic	nonsynonymous SNV	NM_017409	2	c.G884A	p.R295Q		0.26153846	144	51	SNV
YHIM-06	Synergy	HOXC10	chr12	54379308	54379308	G	T	exonic	stopgain	NM_017409	1	c.G265T	p.E89X		0.35333333	97	53	SNV
YHIM-06	Synergy	HOXC10	chr12	54379664	54379664	G	A	exonic	synonymous SNV	NM_017409	1	c.G621A	p.Q207Q		0.30681818	122	54	SNV
YHIM-06	Synergy	HOXC10	chr12	54379718	54379718	G	A	exonic	synonymous SNV	NM_017409	1	c.G675A	p.G225G		0.19318182	71	17	SNV
YHIM-06	Synergy	HOXD12	chr2	176964894	176964894	A	T	exonic	nonsynonymous SNV	NM_021193	1	c.A365T	p.E122V		0.10204082	88	10	SNV
YHIM-06	Synergy	HPD	chr12	122277916	122277916	G	A	exonic	synonymous SNV	NM_002150	13	c.C993T	p.Y331Y		0.2752809	129	49	SNV
YHIM-06	Synergy	IL1RAP	chr3	190374353	190374353	A	G	exonic	nonsynonymous SNV	NM_001167931	12	c.A2021G	p.H674R		0.27513228	137	52	SNV
YHIM-06	Synergy	IMPG1	chr6	76744499	76744499	G	C	exonic	nonsynonymous SNV	NM_001282368	2	c.C73G	p.Q25E		0.43951613	139	109	SNV
YHIM-06	Synergy	INTS7	chr1	212148668	212148668	T	A	exonic	nonsynonymous SNV	NM_001199809	12	c.A1508T	p.Q503L		0.17712177	223	48	SNV
YHIM-06	Synergy	INTS8	chr8	95871808	95871808	A	T	exonic	nonsynonymous SNV	NM_017864	16	c.A2024T	p.Y675F		0.17880795	124	27	SNV
YHIM-06	Synergy	IRAK2	chr3	10254904	10254904	C	G	exonic	nonsynonymous SNV	NM_001570	5	c.C542G	p.S181C		0.6	12	18	SNV
YHIM-06	Synergy	ISM2	chr14	77948785	77948785	G	C	exonic	nonsynonymous SNV	NM_199296	4	c.C853G	p.Q285E		0.75636364	67	208	SNV
YHIM-06	Synergy	ITGA1	chr5	52216182	52216182	C	A	exonic	nonsynonymous SNV	NM_181501	17	c.C2176A	p.L726I		0.33695652	61	31	SNV
YHIM-06	Synergy	ITGA7	chr12	56092338	56092338	C	A	exonic	nonsynonymous SNV	NM_001144996	7	c.G1033T	p.A345S		0.22131148	95	27	SNV
YHIM-06	Synergy	KALRN	chr3	124160808	124160808	G	A	exonic	nonsynonymous SNV	NM_001024660	19	c.G3209A	p.R1070Q		0.29885058	61	26	SNV
YHIM-06	Synergy	KAZN	chr1	14925611	14925611	C	T	exonic	synonymous SNV	NM_015209	1	c.C118T	p.L40L		0.10891089	90	11	SNV
YHIM-06	Synergy	KCNA1	chr12	5021343	5021343	A	T	exonic	nonsynonymous SNV	NM_000217	2	c.A799T	p.I267F		0.06293706	134	9	SNV
YHIM-06	Synergy	KCNA1	chr12	5021344	5021344	T	A	exonic	nonsynonymous SNV	NM_000217	2	c.T800A	p.I267N		0.05673759	133	8	SNV
YHIM-06	Synergy	KCNB2	chr8	73849455	73849455	C	T	exonic	nonsynonymous SNV	NM_004770	3	c.C1865T	p.P622L		0.17327766	396	83	SNV
YHIM-06	Synergy	KCND2	chr7	120381599	120381599	G	T	exonic	synonymous SNV	NM_012281	3	c.G1290T	p.L430L		0.37853107	110	67	SNV
YHIM-06	Synergy	KCNJ16	chr17	68128977	68128977	C	T	exonic	nonsynonymous SNV	NM_001291623	3	c.C854T	p.P285L		0.65777778	77	148	SNV
YHIM-06	Synergy	KCNMA1	chr10	78846318	78846318	G	T	exonic	nonsynonymous SNV	NM_001271518	10	c.C1206A	p.F402L		0.81122449	37	159	SNV
YHIM-06	Synergy	KCNN3	chr1	154841690	154841690	T	C	exonic	nonsynonymous SNV	NM_001204087	1	c.A751G	p.S251G		0.0334728	231	8	SNV
YHIM-06	Synergy	KCNN3	chr1	154841529	154841529	G	C	exonic	synonymous SNV	NM_001204087	1	c.C912G	p.L304L		0.04032258	238	10	SNV
YHIM-06	Synergy	KCNQ4	chr1	41285564	41285564	C	T	exonic	synonymous SNV	NM_004700	6	c.C852T	p.I284I		0.46666667	72	63	SNV
YHIM-06	Synergy	KDM3A	chr2	86697467	86697467	C	G	exonic	nonsynonymous SNV	NM_001146688	11	c.C1660G	p.L554V		0.06569343	128	9	SNV
YHIM-06	Synergy	KDM6A	chrX	44921965	44921965	G	T	exonic	nonsynonymous SNV	NM_001291421	13	c.G611T	p.S204I	CCG	1	0	22	SNV
YHIM-06	Synergy	KIAA0100	chr17	26971140	26971140	C	T	exonic	nonsynonymous SNV	NM_014680	2	c.G134A	p.G45D		0.45098039	28	23	SNV
YHIM-06	Synergy	KIAA1211L	chr2	99443587	99443587	C	G	exonic	nonsynonymous SNV	NM_207362	6	c.G586C	p.A196P		0.49673203	77	76	SNV
YHIM-06	Synergy	KLHDC4	chr16	87742018	87742018	T	G	exonic	nonsynonymous SNV	NM_001184854	9	c.A1331C	p.E444A		0.29787234	66	28	SNV
YHIM-06	Synergy	KLKB1	chr4	187158067	187158067	C	T	exonic	nonsynonymous SNV	NM_000892	5	c.C461T	p.T154M		0.39189189	135	87	SNV
YHIM-06	Synergy	KMT2D	chr12	49438688	49438688	C	T	exonic	nonsynonymous SNV	NM_003482	19	c.G4802A	p.R1601H	CCG	0.13253012	72	11	SNV
YHIM-06	Synergy	KRT5	chr12	52914060	52914060	C	A	exonic	synonymous SNV	NM_000424	1	c.G21T	p.V7V		0.27777778	39	15	SNV
YHIM-06	Synergy	LAMA1	chr18	6993735	6993735	G	A	exonic	nonsynonymous SNV	NM_005559	35	c.C4913T	p.A1638V		0.52830189	50	56	SNV
YHIM-06	Synergy	LCN1	chr9	138413373	138413373	T	C	exonic	synonymous SNV	NM_001252617	1	c.T30C	p.L10L		0.09375	29	3	SNV
YHIM-06	Synergy	LGR4	chr11	27398758	27398758	-	T	exonic	stopgain	NM_018490	12	c.1055dupA	p.Y352_N353delinsX					Indel
YHIM-06	Synergy	LIN9	chr1	226453289	226453289	G	T	exonic	nonsynonymous SNV	NM_001270409	9	c.C926A	p.S309Y		0.26119403	99	35	SNV

YHIM-06	Synergy	LINGO3	chr19	2290490	2290490	C	T	exonic	nonsynonymous SNV	NM_001101391	2	c.G1286A	p.C429Y		0.52941177	72	81	SNV
YHIM-06	Synergy	LIPK	chr10	90503037	90503037	G	C	exonic	nonsynonymous SNV	NM_001080518	8	c.G919C	p.D307H		0.33333333	50	25	SNV
YHIM-06	Synergy	LRCH1	chr13	47315957	47315957	T	A	exonic	nonsynonymous SNV	NM_015116	19	c.T2161A	p.Y721N		0.58823529	14	20	SNV
YHIM-06	Synergy	LRRC56	chr11	552611	552611	T	G	exonic	synonymous SNV	NM_198075	13	c.T1224G	p.A408A		0.28571429	15	6	SNV
YHIM-06	Synergy	MACC1	chr7	20197894	20197894	A	C	exonic	nonsynonymous SNV	NM_182762	5	c.T2090G	p.I697R		0.74657534	37	109	SNV
YHIM-06	Synergy	MAN1B1	chr9	139995598	139995598	G	A	exonic	nonsynonymous SNV	NM_016219	7	c.G1058A	p.R353K		0.6	12	18	SNV
YHIM-06	Synergy	MAPK1	chr22	22127164	22127164	C	T	exonic	nonsynonymous SNV	NM_002745	7	c.G964A	p.E322K		0.38421053	117	73	SNV
YHIM-06	Synergy	MATR3	chr5	138643569	138643569	C	A	exonic	synonymous SNV	NM_001194955	2	c.C465A	p.T155T		0.03821656	151	6	SNV
YHIM-06	Synergy	MATR3	chr5	138643173	138643173	G	A	exonic	synonymous SNV	NM_001194955	2	c.G69A	p.A23A		0.08796296	197	19	SNV
YHIM-06	Synergy	MDH1B	chr2	207620112	207620112	G	C	exonic	synonymous SNV	NM_001039845	5	c.C531G	p.L177L		0.45505618	97	81	SNV
YHIM-06	Synergy	MED1	chr17	37566896	37566896	G	A	exonic	synonymous SNV	NM_004774	17	c.C1578T	p.S526S		0.04040404	95	4	SNV
YHIM-06	Synergy	MESP2	chr15	90320135	90320146	GGG(	-	exonic	nonframeshift deletion	NM_001039958	1	c.547_558del	p.183_186del					Indel
YHIM-06	Synergy	MGAM2	chr7	141857806	141857806	C	T	exonic	nonsynonymous SNV	NM_001293626	18	c.C1993T	p.R665C		0.71428571	44	110	SNV
YHIM-06	Synergy	MOGS	chr2	74689799	74689799	C	G	exonic	nonsynonymous SNV	NM_006302	4	c.G1117C	p.E373Q		1	0	111	SNV
YHIM-06	Synergy	MRPL51	chr12	6601456	6601456	C	T	exonic	nonsynonymous SNV	NM_016497	3	c.G368A	p.R123Q		0.51219512	160	168	SNV
YHIM-06	Synergy	MTUS1	chr8	17541967	17541967	A	T	exonic	nonsynonymous SNV	NM_020749	2	c.T206A	p.L69H		0.21052632	60	16	SNV
YHIM-06	Synergy	MUC1	chr1	155161733	155161733	G	C	exonic	nonsynonymous SNV	NM_001204285	2	c.C400G	p.P134A	CCG	0.15789474	16	3	SNV
YHIM-06	Synergy	MUC12	chr7	100645731	100645731	G	A	exonic	nonsynonymous SNV	NM_001164462	2	c.G11887A	p.E3963K		0.10869565	41	5	SNV
YHIM-06	Synergy	MUC16	chr19	9045609	9045609	G	T	exonic	nonsynonymous SNV	NM_024690	5	c.C36022A	p.P12008T		0.192	101	24	SNV
YHIM-06	Synergy	MUC17	chr7	100685891	100685891	-	T	exonic	frameshift insertion	NM_001040105	3	c.11195dupT	p.I3732fs					Indel
YHIM-06	Synergy	MUC4	chr3	195515194	195515194	G	C	exonic	nonsynonymous SNV	NM_018406	2	c.C3257G	p.T1086S		0.11904762	37	5	SNV
YHIM-06	Synergy	MUC4	chr3	195481175	195481175	C	T	exonic	synonymous SNV	NM_138297	17	c.G2376A	p.E792E		0.18461539	53	12	SNV
YHIM-06	Synergy	MUL1	chr1	20827645	20827645	G	T	exonic	synonymous SNV	NM_024544	4	c.C597A	p.G199G		0.35046729	139	75	SNV
YHIM-06	Synergy	MUM1	chr19	1373103	1373103	G	C	exonic	nonsynonymous SNV	NM_032853	13	c.G2022C	p.E674D		0.44262295	68	54	SNV
YHIM-06	Synergy	MXRA7	chr17	74684201	74684201	C	A	exonic	stopgain	NM_001008528	2	c.G400T	p.E134X		0.2631579	14	5	SNV
YHIM-06	Synergy	MYDGF	chr19	4670307	4670307	T	G	exonic	nonsynonymous SNV	NM_019107	1	c.A40C	p.S14R		0.25641026	87	30	SNV
YHIM-06	Synergy	MYF5	chr12	81111311	81111311	A	-	exonic	frameshift deletion	NM_005593	1	c.469delA	p.T157fs					Indel
YHIM-06	Synergy	MYH2	chr17	10440578	10440578	G	C	exonic	synonymous SNV	NM_001100112	16	c.C1869G	p.L623L		0.32298137	109	52	SNV
YHIM-06	Synergy	MYO5C	chr15	52567803	52567803	C	T	exonic	nonsynonymous SNV	NM_018728	5	c.G562A	p.A188T		0.30025445	275	118	SNV
YHIM-06	Synergy	MYO9B	chr19	17322826	17322826	C	T	exonic	nonsynonymous SNV	NM_004145	40	c.C6181T	p.R2061W		0.41176471	30	21	SNV
YHIM-06	Synergy	MYRIP	chr3	40211480	40211480	G	T	exonic	stopgain	NM_001284426	6	c.G208T	p.E70X		0.06382979	176	12	SNV
YHIM-06	Synergy	NCOR1	chr17	16062082	16062082	C	T	exonic	nonsynonymous SNV	NM_001190438	3	c.G397A	p.E133K	CCG	0.34156379	160	83	SNV
YHIM-06	Synergy	NEIL1	chr15	75644563	75644563	C	T	exonic	synonymous SNV	NM_001256552	3	c.C804T	p.I268I		0.33576642	182	92	SNV
YHIM-06	Synergy	NELL1	chr11	21250969	21250969	G	A	exonic	synonymous SNV	NM_001288714	13	c.G1347A	p.P449P		0.56221198	95	122	SNV
YHIM-06	Synergy	NEUROD6	chr7	31378763	31378763	C	G	exonic	nonsynonymous SNV	NM_022728	2	c.G120C	p.Q40H		0.44818653	213	173	SNV
YHIM-06	Synergy	NLGN3	chrX	70367787	70367787	T	-	exonic	frameshift deletion	NM_001166660	2	c.188delT	p.L63fs					Indel
YHIM-06	Synergy	NLRC5	chr16	57067544	57067544	C	A	exonic	nonsynonymous SNV	NM_032206	11	c.C2506A	p.Q836K		0.31666667	41	19	SNV
YHIM-06	Synergy	NMBR	chr6	142400023	142400023	T	C	exonic	nonsynonymous SNV	NM_002511	2	c.A440G	p.N147S		0.796875	13	51	SNV
YHIM-06	Synergy	NOTCH1	chr9	139417639	139417639	C	-	exonic	frameshift deletion	NM_017617	4	c.405delG	p.G135fs	CCG				Indel
YHIM-06	Synergy	NOTCH1	chr9	139399551	139399551	-	G	exonic	frameshift insertion	NM_017617	26	c.4591dupC	p.L1531fs	CCG				Indel
YHIM-06	Synergy	NPC1	chr18	21124380	21124380	C	T	exonic	synonymous SNV	NM_000271	13	c.G2058A	p.V686V		0.37634409	58	35	SNV
YHIM-06	Synergy	NPTN	chr15	73889631	73889631	G	A	exonic	synonymous SNV	NM_001161363	2	c.C171T	p.S57S		0.04347826	418	19	SNV
YHIM-06	Synergy	NR2F2	chr15	96875583	96875583	A	G	exonic	synonymous SNV	NM_021005	1	c.A249G	p.G83G		0.04741379	221	11	SNV
YHIM-06	Synergy	NR4A2	chr2	157186546	157186546	G	A	exonic	synonymous SNV	NM_006186	3	c.C153T	p.I51I		0.04519774	169	8	SNV
YHIM-06	Synergy	NRAP	chr10	115422448	115422448	G	T	exonic	nonsynonymous SNV	NM_001261463	3	c.C245A	p.A82D		0.30653266	276	122	SNV
YHIM-06	Synergy	NRAP	chr10	115422447	115422447	G	A	exonic	synonymous SNV	NM_001261463	3	c.C246T	p.A82A		0.3101737	278	125	SNV
YHIM-06	Synergy	NRG1	chr8	32406320	32406320	T	C	exonic	nonsynonymous SNV	NM_001160002	1	c.T76C	p.S26P	CCG	0.40540541	22	15	SNV
YHIM-06	Synergy	NSMCE4A	chr10	123734604	123734604	G	C	exonic	nonsynonymous SNV	NM_001167865	1	c.C77G	p.S26C		0.1875	13	3	SNV
YHIM-06	Synergy	NUBPL	chr14	32030666	32030666	G	C	exonic	synonymous SNV	NM_025152	1	c.G21C	p.L7L		0.2625	118	42	SNV
YHIM-06	Synergy	NUP88	chr17	5289589	5289589	C	A	exonic	nonsynonymous SNV	NM_002532	17	c.G2163T	p.E721D		0.2	12	3	SNV
YHIM-06	Synergy	NXPH1	chr7	8791205	8791205	A	G	exonic	nonsynonymous SNV	NM_152745	3	c.A622G	p.T208A		0.15225807	657	118	SNV
YHIM-06	Synergy	OR51E1	chr11	4674661	4674661	G	A	exonic	nonsynonymous SNV	NM_152430	2	c.G905A	p.R302Q		1	0	154	SNV
YHIM-06	Synergy	OR51S1	chr11	4870283	4870283	G	T	exonic	synonymous SNV	NM_001004758	1	c.C156A	p.T52T		0.45132743	62	51	SNV
YHIM-06	Synergy	OSBPL11	chr3	125286257	125286257	C	G	exonic	nonsynonymous SNV	NM_022776	6	c.G849C	p.Q283H		0.65758755	88	169	SNV
YHIM-06	Synergy	OTOP1	chr4	4228274	4228282	CCAC	-	exonic	nonframeshift deletion	NM_177998	1	c.310_318del	p.104_106del					Indel
YHIM-06	Synergy	OTUD5	chrX	48781326	48781326	G	A	exonic	nonsynonymous SNV	NM_001136157	7	c.C1267T	p.R423W		1	0	10	SNV
YHIM-06	Synergy	PALD1	chr10	72285838	72285838	A	G	exonic	nonsynonymous SNV	NM_014431	2	c.A131G	p.H44R		0.19633508	307	75	SNV
YHIM-06	Synergy	PANX2	chr22	50616301	50616301	C	T	exonic	nonsynonymous SNV	NM_001160300	2	c.C1160T	p.A387V		1	0	259	SNV
YHIM-06	Synergy	PCDH18	chr4	138452913	138452913	C	G	exonic	synonymous SNV	NM_001300828	1	c.G330C	p.V110V		0.48863636	135	129	SNV
YHIM-06	Synergy	PCDH18	chr4	138452202	138452202	A	T	exonic	synonymous SNV	NM_001300828	1	c.T1041A	p.V347V		0.62	19	31	SNV
YHIM-06	Synergy	PCDH20	chr13	61985575	61985575	C	G	exonic	nonsynonymous SNV	NM_022843	2	c.G2657C	p.C886S		1	0	132	SNV
YHIM-06	Synergy	PCDH8	chr13	53420575	53420575	C	T	exonic	nonsynonymous SNV	NM_002590	1	c.G1997A	p.R666H		1	0	123	SNV

YHIM-06	Synergy	PCDHB15	chr5	140625723	140625723	G	C	exonic	nonsynonymous SNV	NM_018935	1	c.G577C	p.E193Q		0.47894737	99	91	SNV
YHIM-06	Synergy	PCDHGA1	chr5	140710254	140710254	G	T	exonic	nonsynonymous SNV	NM_018912	1	c.G3T	p.M1I		0.44444444	35	28	SNV
YHIM-06	Synergy	PCDHGA3	chr5	140723794	140723794	G	A	exonic	nonsynonymous SNV	NM_018916	1	c.G194A	p.R65H		0.66	17	33	SNV
YHIM-06	Synergy	PCDHGB1	chr5	140731391	140731391	C	T	exonic	nonsynonymous SNV	NM_018922	1	c.C1564T	p.R522C		0.53333333	35	40	SNV
YHIM-06	Synergy	PCNXL2	chr1	233296122	233296122	A	T	exonic	nonsynonymous SNV	NM_014801	19	c.T3424A	p.Y1142N		0.3	98	42	SNV
YHIM-06	Synergy	PCSK5	chr9	78784630	78784630	C	A	exonic	nonsynonymous SNV	NM_001190482	13	c.C1630A	p.H544N		0.2601626	91	32	SNV
YHIM-06	Synergy	PDE3B	chr11	14810687	14810687	G	T	exonic	synonymous SNV	NM_000922	4	c.G1314T	p.L438L		0.51851852	39	42	SNV
YHIM-06	Synergy	PDE4DIP	chr1	144882656	144882656	T	G	exonic	nonsynonymous SNV	NM_001198834	24	c.A3363C	p.E1121D	CCG	0.08	207	18	SNV
YHIM-06	Synergy	PEBP4	chr8	22570924	22570924	C	T	exonic	nonsynonymous SNV	NM_144962	7	c.G643A	p.E215K		0.19230769	21	5	SNV
YHIM-06	Synergy	PKD1L2	chr16	81232481	81232481	T	C	exonic	synonymous SNV	NM_001076780	7	c.A1329G	p.T443T		0.15384615	66	12	SNV
YHIM-06	Synergy	PLA2G4F	chr15	42442937	42442937	C	T	exonic	nonsynonymous SNV	NM_213600	8	c.G640A	p.E214K		0.32307692	44	21	SNV
YHIM-06	Synergy	PLCH2	chr1	2430086	2430086	-	GTGG(	exonic	nonframeshift insertion	NM_001303012	17	c.2268_2269ins(	p.E756delinsEVGA					Indel
YHIM-06	Synergy	PLEC	chr8	144993126	144993126	G	A	exonic	synonymous SNV	NM_201378	32	c.C10821T	p.I3607I		0.2496925	610	203	SNV
YHIM-06	Synergy	PNISR	chr6	99848745	99848746	TT	-	exonic	frameshift deletion	NM_015491	11	c.2088_2089del	p.K696fs					Indel
YHIM-06	Synergy	POM121L12	chr7	53103931	53103931	C	T	exonic	synonymous SNV	NM_182595	1	c.C567T	p.S189S		0.53650794	146	169	SNV
YHIM-06	Synergy	POP1	chr8	99170248	99170248	G	A	exonic	nonsynonymous SNV	NM_001145860	16	c.G2824A	p.A942T		0.1402439	282	46	SNV
YHIM-06	Synergy	POU4F1	chr13	79176423	79176423	G	A	exonic	synonymous SNV	NM_006237	2	c.C387T	p.A129A		1	0	24	SNV
YHIM-06	Synergy	POU6F2	chr7	39125590	39125590	T	C	exonic	nonsynonymous SNV	NM_001166018	3	c.T149C	p.V50A		0.06086957	216	14	SNV
YHIM-06	Synergy	POU6F2	chr7	39125588	39125588	T	C	exonic	synonymous SNV	NM_001166018	3	c.T147C	p.P49P		0.06008584	219	14	SNV
YHIM-06	Synergy	PPARG	chr3	12458209	12458209	G	T	exonic	nonsynonymous SNV	NM_005037	6	c.G742T	p.V248F	CCG	0.25	63	21	SNV
YHIM-06	Synergy	PPM1M	chr3	52281211	52281211	G	T	exonic	synonymous SNV	NM_144641	3	c.G564T	p.V188V		0.44329897	54	43	SNV
YHIM-06	Synergy	PTPN6	chr12	7069578	7069578	G	C	exonic	nonsynonymous SNV	NM_002831	14	c.G1653C	p.K551N		0.4765625	134	122	SNV
YHIM-06	Synergy	PTPRM	chr18	8244073	8244073	G	A	exonic	nonsynonymous SNV	NM_001105244	15	c.G2318A	p.R773Q		0.424	72	53	SNV
YHIM-06	Synergy	PXN	chr12	120662133	120662133	G	A	exonic	nonsynonymous SNV	NM_001080855	2	c.C61T	p.R21W		0.35057471	113	61	SNV
YHIM-06	Synergy	RAB3C	chr5	58147090	58147090	T	C	exonic	nonsynonymous SNV	NM_138453	5	c.T596C	p.L199S		0.53246753	108	123	SNV
YHIM-06	Synergy	RAD54B	chr8	95416414	95416414	T	C	exonic	nonsynonymous SNV	NM_001205263	4	c.A283G	p.N95D		0.17330677	415	87	SNV
YHIM-06	Synergy	RAET1G	chr6	150240835	150240835	C	T	exonic	nonsynonymous SNV	NM_001001788	2	c.G203A	p.S68N		0.14814815	23	4	SNV
YHIM-06	Synergy	RAPH1	chr2	204304670	204304671	CT	-	exonic	frameshift deletion	NM_213589	14	c.3242_3243del	p.E1081fs					Indel
YHIM-06	Synergy	RBBP5	chr1	205064043	205064043	C	T	exonic	nonsynonymous SNV	NM_001193273	13	c.G1165A	p.A389T		0.04444444	86	4	SNV
YHIM-06	Synergy	RELN	chr7	103180884	103180884	C	T	exonic	nonsynonymous SNV	NM_005045	44	c.G6690A	p.M2230I		0.59016393	50	72	SNV
YHIM-06	Synergy	REXO2	chr11	114314580	114314580	T	C	exonic	synonymous SNV	NM_015523	3	c.T234C	p.G78G		0.37142857	132	78	SNV
YHIM-06	Synergy	RFX6	chr6	117250106	117250106	T	C	exonic	synonymous SNV	NM_173560	18	c.T2583C	p.S861S		0.04166667	92	4	SNV
YHIM-06	Synergy	RIMS1	chr6	72922910	72922910	C	G	exonic	nonsynonymous SNV	NM_001168407	1	c.C85G	p.R29G		0.4122807	201	141	SNV
YHIM-06	Synergy	RSPH6A	chr19	46307866	46307866	C	T	exonic	nonsynonymous SNV	NM_030785	3	c.G1297A	p.E433K		0.48	104	96	SNV
YHIM-06	Synergy	SAMD4A	chr14	55203869	55203869	C	T	exonic	synonymous SNV	NM_015589	3	c.C843T	p.P281P		0.07342657	265	21	SNV
YHIM-06	Synergy	SCPEP1	chr17	55078301	55078301	G	C	exonic	synonymous SNV	NM_021626	11	c.G1080C	p.G360G		0.3046875	89	39	SNV
YHIM-06	Synergy	SCRT1	chr8	145557305	145557305	C	T	exonic	nonsynonymous SNV	NM_031309	2	c.G589A	p.G197S		0.16231343	449	87	SNV
YHIM-06	Synergy	SEH1L	chr18	12963299	12963299	C	T	exonic	synonymous SNV	NM_001013437	4	c.C450T	p.L150L		0.23660714	171	53	SNV
YHIM-06	Synergy	04-Sep	chr17	56599160	56599160	G	A	exonic	synonymous SNV	NM_001198713	7	c.C828T	p.I276I		0.09259259	98	10	SNV
YHIM-06	Synergy	SGCA	chr17	48244771	48244771	C	T	exonic	nonsynonymous SNV	NM_000023	2	c.C80T	p.T27M		0.29679144	263	111	SNV
YHIM-06	Synergy	SGSM1	chr22	25297841	25297841	C	T	exonic	synonymous SNV	NM_001098498	19	c.C2469T	p.I823I		0.76237624	24	77	SNV
YHIM-06	Synergy	SHROOM2	chrX	9864449	9864449	G	A	exonic	nonsynonymous SNV	NM_001649	4	c.G2501A	p.R834H		1	0	87	SNV
YHIM-06	Synergy	SIGLEC12	chr19	52001369	52001369	C	T	exonic	synonymous SNV	NM_033329	4	c.G954A	p.V318V		0.54716981	24	29	SNV
YHIM-06	Synergy	SLC17A4	chr6	25762281	25762281	G	A	exonic	nonsynonymous SNV	NM_005495	2	c.G91A	p.G31S		0.05027933	340	18	SNV
YHIM-06	Synergy	SLC25A2	chr5	140682837	140682837	G	A	exonic	nonsynonymous SNV	NM_031947	1	c.C596T	p.A199V		0.44512195	91	73	SNV
YHIM-06	Synergy	SLC3A2	chr11	62652984	62652984	C	T	exonic	synonymous SNV	NM_001013251	7	c.C1047T	p.L349L		0.29516539	277	116	SNV
YHIM-06	Synergy	SLC6A12	chr12	313792	313792	A	G	exonic	nonsynonymous SNV	NM_001206931	3	c.T287C	p.L96S		0.46835443	126	111	SNV
YHIM-06	Synergy	SLC6A6	chr3	14513720	14513720	C	T	exonic	synonymous SNV	NM_003043	10	c.C1104T	p.G368G		0.66304348	31	61	SNV
YHIM-06	Synergy	SLFN12L	chr17	33806550	33806550	A	C	exonic	nonsynonymous SNV	NM_001195790	2	c.T679G	p.F227V		0.13157895	66	10	SNV
YHIM-06	Synergy	SLITRK3	chr3	164907198	164907198	C	A	exonic	nonsynonymous SNV	NM_014926	2	c.G1421T	p.G474V		0.52	120	130	SNV
YHIM-06	Synergy	SMAD7	chr18	46476357	46476357	A	G	exonic	synonymous SNV	NM_001190821	1	c.T438C	p.P146P		0.05594406	135	8	SNV
YHIM-06	Synergy	SMCR8	chr17	18221303	18221303	G	A	exonic	nonsynonymous SNV	NM_144775	1	c.G2200A	p.V734M		0.07365439	327	26	SNV
YHIM-06	Synergy	SMOX	chr20	4162532	4162532	G	A	exonic	nonsynonymous SNV	NM_001270691	4	c.G518A	p.R173Q		0.4591195	86	73	SNV
YHIM-06	Synergy	SNAP91	chr6	84417611	84417611	G	A	exonic	synonymous SNV	NM_001242792	2	c.C36T	p.A12A		0.3526971	156	85	SNV
YHIM-06	Synergy	SORCS1	chr10	108536330	108536330	G	A	exonic	stopgain	NM_001013031	4	c.C847T	p.Q283X		0.30447761	233	102	SNV
YHIM-06	Synergy	SORCS2	chr4	7728529	7728529	C	G	exonic	nonsynonymous SNV	NM_020777	21	c.C2768G	p.S923C		0.36363636	133	76	SNV
YHIM-06	Synergy	SORL1	chr11	121348846	121348846	A	T	exonic	nonsynonymous SNV	NM_003105	3	c.A422T	p.Y141F		0.27	73	27	SNV
YHIM-06	Synergy	SPEF2	chr5	35806951	35806951	A	C	exonic	nonsynonymous SNV	NM_024867	35	c.A5153C	p.K1718T		0.29718876	175	74	SNV
YHIM-06	Synergy	ST7L	chr1	113134186	113134186	C	T	exonic	nonsynonymous SNV	NM_001308264	5	c.G113A	p.R38Q		0.36363636	14	8	SNV
YHIM-06	Synergy	STARD9	chr15	42986852	42986852	G	A	exonic	stopgain	NM_020759	24	c.G12891A	p.W4297X		0.07614213	182	15	SNV
YHIM-06	Synergy	STAU2	chr8	74495043	74495043	C	A	exonic	nonsynonymous SNV	NM_001164383	7	c.G668T	p.S223I		0.10569106	110	13	SNV
YHIM-06	Synergy	STRBP	chr9	125921478	125921478	T	C	exonic	synonymous SNV	NM_001171137	9	c.A690G	p.E230E		0.04807692	99	5	SNV

YHIM-06	Synergy	STX18	chr4	4440184	4440184	C	G	exonic	nonsynonymous SNV	NM_016930	6	c.G550C	p.E184Q		0.65714286	24	46	SNV
YHIM-06	Synergy	SULF1	chr8	70536352	70536352	C	A	exonic	synonymous SNV	NM_001128204	14	c.C1770A	p.S590S		0.12635379	242	35	SNV
YHIM-06	Synergy	SUPT5H	chr19	39964672	39964672	C	T	exonic	synonymous SNV	NM_001130825	25	c.C2550T	p.T850T		0.46601942	55	48	SNV
YHIM-06	Synergy	SYNE1	chr6	152711482	152711482	G	T	exonic	nonsynonymous SNV	NM_033071	53	c.C8131A	p.Q2711K		0.29166667	51	21	SNV
YHIM-06	Synergy	SYNPO2L	chr10	75408036	75408036	G	A	exonic	synonymous SNV	NM_024875	2	c.C702T	p.T234T		0.66423358	46	91	SNV
YHIM-06	Synergy	TACR2	chr10	71164660	71164660	C	T	exonic	synonymous SNV	NM_001057	5	c.G1119A	p.A373A		0.79710145	84	330	SNV
YHIM-06	Synergy	TAF4	chr20	60640111	60640119	GGG(	-	exonic	nonframeshift deletion	NM_003185	1	c.748_756del	p.250_252del					Indel
YHIM-06	Synergy	TARS	chr5	33461798	33461798	T	C	exonic	synonymous SNV	NM_152295	14	c.T1578C	p.F526F		0.42857143	136	102	SNV
YHIM-06	Synergy	TBX3	chr12	115109830	115109830	G	A	exonic	nonsynonymous SNV	NM_005996	7	c.C1988T	p.S663L	CCG	0.3968254	76	50	SNV
YHIM-06	Synergy	TCTN1	chr12	111080168	111080168	C	G	exonic	stopgain	NM_001082537	11	c.C1305G	p.Y435X		0.04	168	7	SNV
YHIM-06	Synergy	TDG	chr12	104376608	104376608	G	A	exonic	synonymous SNV	NM_003211	5	c.G510A	p.E170E		0.08571429	32	3	SNV
YHIM-06	Synergy	TEX15	chr8	30701203	30701203	C	G	exonic	nonsynonymous SNV	NM_031271	1	c.G5331C	p.K1777N		0.49056604	27	26	SNV
YHIM-06	Synergy	TEX2	chr17	62290200	62290200	C	-	exonic	frameshift deletion	NM_001288732	2	c.1378delG	p.D460fs					Indel
YHIM-06	Synergy	TFB2M	chr1	246704420	246704420	T	C	exonic	synonymous SNV	NM_022366	8	c.A1104G	p.Q368Q		0.11363636	78	10	SNV
YHIM-06	Synergy	TG	chr8	134042164	134042164	C	T	exonic	nonsynonymous SNV	NM_003235	41	c.C7135T	p.R2379C		0.16097561	172	33	SNV
YHIM-06	Synergy	TGM2	chr20	36758627	36758627	G	A	exonic	synonymous SNV	NM_004613	13	c.C2058T	p.P686P		0.18110236	312	69	SNV
YHIM-06	Synergy	TGS1	chr8	56695371	56695371	G	A	exonic	nonsynonymous SNV	NM_024831	2	c.G166A	p.G56R		0.0875	73	7	SNV
YHIM-06	Synergy	TLN1	chr9	35700258	35700258	C	T	exonic	nonsynonymous SNV	NM_006289	49	c.G6590A	p.R2197H		0.66666667	110	220	SNV
YHIM-06	Synergy	TMEM151B	chr6	44241225	44241225	C	T	exonic	synonymous SNV	NM_001137560	2	c.C558T	p.D186D		0.04347826	176	8	SNV
YHIM-06	Synergy	TMEM163	chr2	135470857	135470857	G	A	exonic	nonsynonymous SNV	NM_030923	2	c.C235T	p.H79Y		0.43333333	51	39	SNV
YHIM-06	Synergy	TMEM200C	chr18	5890763	5890763	C	T	exonic	nonsynonymous SNV	NM_001080209	1	c.G1300A	p.G434R		0.16666667	35	7	SNV
YHIM-06	Synergy	TMEM201	chr1	9662127	9662127	G	T	exonic	nonsynonymous SNV	NM_001010866	6	c.G957T	p.R319S		0.38333333	37	23	SNV
YHIM-06	Synergy	TMEM266	chr15	76430064	76430064	G	A	exonic	nonsynonymous SNV	NM_152335	3	c.G55A	p.G19R		0.28078818	146	57	SNV
YHIM-06	Synergy	TMPRSS13	chr11	117789342	117789342	T	C	exonic	nonsynonymous SNV	NM_001077263	2	c.A233G	p.Q78R		0.12765957	41	6	SNV
YHIM-06	Synergy	TMPRSS13	chr11	117789345	117789345	G	C	exonic	nonsynonymous SNV	NM_001077263	2	c.C230G	p.A77G		0.12765957	41	6	SNV
YHIM-06	Synergy	TMPRSS13	chr11	117780660	117780660	C	T	exonic	nonsynonymous SNV	NM_001206789	7	c.G865A	p.G289R		0.25225225	83	28	SNV
YHIM-06	Synergy	TMPRSS5	chr11	113558983	113558983	C	A	exonic	stoploss	NM_001288749	10	c.G1034T	p.X345L		0.2605042	88	31	SNV
YHIM-06	Synergy	TNFRSF17	chr16	12060107	12060107	G	A	exonic	synonymous SNV	NM_001192	2	c.G186A	p.L62L	CCG	0.29179331	233	96	SNV
YHIM-06	Synergy	TNFRSF25	chr1	6525507	6525507	G	T	exonic	stopgain	NM_001039664	2	c.C153A	p.C51X		0.30046948	149	64	SNV
YHIM-06	Synergy	TNN	chr1	175046791	175046791	G	A	exonic	synonymous SNV	NM_022093	2	c.G237A	p.R79R		0.35964912	146	82	SNV
YHIM-06	Synergy	TP53	chr17	7577120	7577120	C	T	exonic	nonsynonymous SNV	NM_001126115	4	c.G422A	p.R141H	CCG	1	0	46	SNV
YHIM-06	Synergy	TRIM36	chr5	114499230	114499230	G	A	exonic	stopgain	NM_001300759	2	c.C247T	p.R83X		0.46391753	52	45	SNV
YHIM-06	Synergy	TRIM49	chr11	89531764	89531764	T	C	exonic	nonsynonymous SNV	NM_020358	8	c.A893G	p.N298S		0.05825243	97	6	SNV
YHIM-06	Synergy	TRMT6	chr20	5925497	5925497	G	A	exonic	nonsynonymous SNV	NM_015939	3	c.C320T	p.T107I		0.5	25	25	SNV
YHIM-06	Synergy	TRPS1	chr8	116616812	116616812	G	T	exonic	nonsynonymous SNV	NM_001282902	3	c.C1357A	p.L453I		0.17437722	232	49	SNV
YHIM-06	Synergy	TRPV4	chr12	110222126	110222126	C	T	exonic	nonsynonymous SNV	NM_001177433	12	c.G2132A	p.R711H		0.28712871	72	29	SNV
YHIM-06	Synergy	TRRAP	chr7	98508869	98508869	G	C	exonic	nonsynonymous SNV	NM_001244580	17	c.G1982C	p.R661T	CCG	0.5	40	40	SNV
YHIM-06	Synergy	TSPAN5	chr4	99579354	99579354	A	G	exonic	synonymous SNV	NM_005723	1	c.T24C	p.G8G		0.63157895	56	96	SNV
YHIM-06	Synergy	TXLNB	chr6	139583740	139583740	G	C	exonic	nonsynonymous SNV	NM_153235	5	c.C858G	p.I286M		0.19417476	166	40	SNV
YHIM-06	Synergy	USP42	chr7	6193602	6193602	G	A	exonic	nonsynonymous SNV	NM_032172	15	c.G2417A	p.G806D		0.64495114	109	198	SNV
YHIM-06	Synergy	VLDLR	chr9	2652835	2652835	C	G	exonic	nonsynonymous SNV	NM_001018056	17	c.C2388G	p.H796Q		0.07403055	788	63	SNV
YHIM-06	Synergy	VPS4A	chr16	69355096	69355096	G	A	exonic	nonsynonymous SNV	NM_013245	9	c.G994A	p.A332T		0.28358209	96	38	SNV
YHIM-06	Synergy	WDFY4	chr10	49998790	49998790	C	T	exonic	nonsynonymous SNV	NM_020945	23	c.C4085T	p.S1362F		0.12946429	390	58	SNV
YHIM-06	Synergy	WDFY4	chr10	49982607	49982607	C	G	exonic	synonymous SNV	NM_020945	14	c.C2658G	p.L886L		0.8619403	37	231	SNV
YHIM-06	Synergy	WDR37	chr10	1175210	1175210	C	A	exonic	nonsynonymous SNV	NM_014023	14	c.C1411A	p.L471M		0.36263736	116	66	SNV
YHIM-06	Synergy	WDR54	chr2	74652600	74652601	AG	-	exonic	frameshift deletion	NM_032118	9	c.855_856del	p.P285fs					Indel
YHIM-06	Synergy	WDR66	chr12	122406040	122406040	C	T	exonic	synonymous SNV	NM_001178003	17	c.C2736T	p.H912H		0.30737705	169	75	SNV
YHIM-06	Synergy	WNK2	chr9	96021405	96021405	G	T	exonic	nonsynonymous SNV	NM_001282394	11	c.G2575T	p.V859L		0.09462916	354	37	SNV
YHIM-06	Synergy	WWP1	chr8	87473544	87473544	T	A	exonic	nonsynonymous SNV	NM_007013	23	c.T2591A	p.L864H		0.11458333	255	33	SNV
YHIM-06	Synergy	XIRP1	chr3	39225676	39225676	G	A	exonic	nonsynonymous SNV	NM_194293	2	c.C5261T	p.T1754M		0.36170213	90	51	SNV
YHIM-06	Synergy	XPO4	chr13	21381617	21381617	T	C	exonic	nonsynonymous SNV	NM_022459	13	c.A1796G	p.N599S		0.09756098	37	4	SNV
YHIM-06	Synergy	XYLT1	chr16	17352970	17352970	T	A	exonic	nonsynonymous SNV	NM_022166	3	c.A788T	p.E263V		0.16326531	205	40	SNV
YHIM-06	Synergy	YLPM1	chr14	75265123	75265123	C	T	exonic	synonymous SNV	NM_019589	5	c.C3123T	p.R1041R		0.80544747	50	207	SNV
YHIM-06	Synergy	ZBTB14	chr18	5291025	5291025	G	A	exonic	synonymous SNV	NM_003409	3	c.C1182T	p.C394C		0.22426471	211	61	SNV
YHIM-06	Synergy	ZBTB40	chr1	22843893	22843893	C	A	exonic	stopgain	NM_014870	13	c.C2769A	p.C923X		0.34200744	177	92	SNV
YHIM-06	Synergy	ZFHX3	chr16	72845497	72845497	G	A	exonic	synonymous SNV	NM_001164766	6	c.C1101T	p.C367C	CCG	0.27272727	16	6	SNV
YHIM-06	Synergy	ZFHX4	chr8	77764152	77764152	A	T	exonic	nonsynonymous SNV	NM_024721	10	c.A4995T	p.L1665F		0.03778338	382	15	SNV
YHIM-06	Synergy	ZFP64	chr20	50701312	50701312	G	A	exonic	synonymous SNV	NM_199427	9	c.C1722T	p.I574I		0.33397313	347	174	SNV
YHIM-06	Synergy	ZFYVE28	chr4	2343208	2343208	G	A	exonic	synonymous SNV	NM_001172658	2	c.C174T	p.A58A		0.33333333	12	6	SNV
YHIM-06	Synergy	ZNF131	chr5	43161813	43161813	G	A	exonic	synonymous SNV	NM_001297548	5	c.G834A	p.E278E		0.05660377	300	18	SNV
YHIM-06	Synergy	ZNF18	chr17	11881561	11881561	G	A	exonic	stopgain	NM_001303281	7	c.C1363T	p.Q455X		0.37037037	68	40	SNV
YHIM-06	Synergy	ZNF207	chr17	30693698	30693698	C	A	exonic	synonymous SNV	NM_003457	8	c.C795A	p.V265V		0.03652968	211	8	SNV

YHIM-06	Synergy	ZNF334	chr20	45131328	45131328	G	T	exonic	nonsynonymous SNV	NM_018102	5	c.C650A	p.T217N		0.09348442	320	33	SNV
YHIM-06	Synergy	ZNF598	chr16	2048833	2048833	G	A	exonic	nonsynonymous SNV	NM_178167	13	c.C2231T	p.P744L		0.2972973	78	33	SNV
YHIM-06	Synergy	ZNF629	chr16	30794920	30794920	G	A	exonic	synonymous SNV	NM_001080417	3	c.C729T	p.F243F		0.68472906	64	139	SNV
YHIM-06	Synergy	ZNF667	chr19	56953705	56953705	C	A	exonic	nonsynonymous SNV	NM_022103	5	c.G659T	p.R220I		0.45033113	83	68	SNV
YHIM-06	Synergy	ZNF669	chr1	247267330	247267330	C	A	exonic	nonsynonymous SNV	NM_024804	1	c.G172T	p.A58S		0.36170213	30	17	SNV
YHIM-06	Synergy	ZNF718	chr4	155362	155362	C	G	exonic	stopgain	NM_001039127	5	c.C887G	p.S296X		0.29166667	68	28	SNV
YHIM-06	Synergy	ZNF727	chr7	63538455	63538455	A	T	exonic	nonsynonymous SNV	NM_001159522	4	c.A1028T	p.E343V		0.52325581	41	45	SNV
YHIM-06	Synergy	ZNF763	chr19	12089188	12089188	A	C	exonic	nonsynonymous SNV	NM_001012753	4	c.A458C	p.K153T		0.33185841	302	150	SNV
YHIM-06	Synergy	ZNF780B	chr19	40541501	40541501	C	G	exonic	nonsynonymous SNV	NM_001005851	5	c.G1265C	p.C422S		0.41304348	27	19	SNV
YHIM-06	Synergy	ZSWIM4	chr19	13920010	13920010	T	C	exonic	nonsynonymous SNV	NM_023072	5	c.T988C	p.C330R		0.58571429	29	41	SNV
YHIM-06	Synergy	ZSWIM5	chr1	45553697	45553697	C	T	exonic	nonsynonymous SNV	NM_020883	2	c.G808A	p.E270K		0.33630952	223	113	SNV
YHIM-06	Synergy	ZW10	chr11	113631237	113631237	C	G	exonic	nonsynonymous SNV	NM_004724	4	c.G418C	p.E140Q		0.25203252	92	31	SNV
YHIM-06	Synergy	CNTRL	chr9	123874857	123874857	G	T	splicing	splicing	NM_007018	7	c.1122+1G>T			0.08641975	74	7	SNV
YHIM-06	Synergy	MST1L	chr1	17086183	17086183	T	G	splicing	splicing	NM_001271733	7	c.716-2A>C			0.11363636	39	5	SNV
YHIM-06	Synergy	MUT	chr6	49419428	49419428	C	A	splicing	splicing	NM_000255	6	c.1084-1G>T			0.04356847	461	21	SNV
YHIM-06	Synergy	RNF13	chr3	149619858	149619858	G	A	splicing	splicing	NM_183381	6	c.410-1G>A			0.29787234	33	14	SNV
YHIM-07	Synergy	ABCA13	chr7	48443403	48443403	G	A	exonic	synonymous SNV	NM_152701	39	c.G11997A	p.L3999L		0.34693878	448	238	SNV
YHIM-07	Synergy	ABCA4	chr1	94508353	94508353	G	A	exonic	nonsynonymous SNV	NM_000350	22	c.C3292T	p.R1098C		0.48131868	236	219	SNV
YHIM-07	Synergy	ABCB5	chr7	20698214	20698214	G	A	exonic	nonsynonymous SNV	NM_001163942	5	c.G287A	p.R96Q		0.11527378	307	40	SNV
YHIM-07	Synergy	ADCY5	chr3	123166376	123166376	G	C	exonic	nonsynonymous SNV	NM_183357	1	c.C1017G	p.I339M		0.30990415	432	194	SNV
YHIM-07	Synergy	ADGB	chr6	147103289	147103289	G	T	exonic	nonsynonymous SNV	NM_024694	30	c.G3996T	p.K1332N		0.47916667	75	69	SNV
YHIM-07	Synergy	ADGRL3	chr4	62801694	62801694	C	T	exonic	nonsynonymous SNV	NM_015236	12	c.C2146T	p.P716S		0.484	129	121	SNV
YHIM-07	Synergy	AGO2	chr8	141542698	141542698	G	A	exonic	nonsynonymous SNV	NM_001164623	17	c.C2186T	p.S729L		0.44303798	88	70	SNV
YHIM-07	Synergy	ANGEL2	chr1	213186654	213186654	T	C	exonic	nonsynonymous SNV	NM_144567	2	c.A166G	p.R56G		0.30088496	158	68	SNV
YHIM-07	Synergy	ANKDD1A	chr15	65208036	65208036	G	A	exonic	synonymous SNV	NM_182703	2	c.G75A	p.Q25Q		0.40880503	94	65	SNV
YHIM-07	Synergy	ANKS1A	chr6	34857302	34857302	-	GGCG	exonic	nonframeshift insertion	NM_015245	1	c.123_124insGG	p.G41delinsGGG					Indel
YHIM-07	Synergy	ANO9	chr11	420945	420945	G	A	exonic	nonsynonymous SNV	NM_001012302	17	c.C1490T	p.P497L		0.36538462	33	19	SNV
YHIM-07	Synergy	AOC2	chr17	40998144	40998144	C	T	exonic	nonsynonymous SNV	NM_001158	1	c.C1501T	p.L501F		0.49321267	112	109	SNV
YHIM-07	Synergy	AOC2	chr17	40998146	40998146	C	T	exonic	synonymous SNV	NM_001158	1	c.C1503T	p.L501L		0.49773756	111	110	SNV
YHIM-07	Synergy	APOBEC2	chr6	41029396	41029396	T	A	exonic	nonsynonymous SNV	NM_006789	2	c.T461A	p.L154H		0.39259259	82	53	SNV
YHIM-07	Synergy	APOL6	chr22	36055312	36055312	G	A	exonic	nonsynonymous SNV	NM_030641	3	c.G701A	p.R234H		0.2012987	123	31	SNV
YHIM-07	Synergy	ARHGAP33	chr19	36277635	36277635	C	T	exonic	nonsynonymous SNV	NM_001172630	19	c.C1855T	p.P619S		1	0	21	SNV
YHIM-07	Synergy	ARID2	chr12	46246405	46246405	C	A	exonic	stopgain	NM_152641	15	c.C4499A	p.S1500X	CCG	0.99684543	1	316	SNV
YHIM-07	Synergy	ARNT2	chr15	80845032	80845032	G	C	exonic	nonsynonymous SNV	NM_014862	10	c.G1006C	p.E336Q		0.32068966	197	93	SNV
YHIM-07	Synergy	ASPM	chr1	197070978	197070978	-	A	exonic	frameshift insertion	NM_018136	18	c.7402dupT	p.Y2468fs					Indel
YHIM-07	Synergy	ASTN1	chr1	176926855	176926855	G	A	exonic	nonsynonymous SNV	NM_001286164	11	c.C1846T	p.R616C		0.37333333	47	28	SNV
YHIM-07	Synergy	ATP10A	chr15	25959194	25959194	C	G	exonic	nonsynonymous SNV	NM_024490	10	c.G1971C	p.R657S		0.32512315	137	66	SNV
YHIM-07	Synergy	AVIL	chr12	58197374	58197374	C	A	exonic	stopgain	NM_006576	14	c.G1750T	p.E584X		0.20238095	67	17	SNV
YHIM-07	Synergy	AZIN1	chr8	103841565	103841565	A	G	exonic	synonymous SNV	NM_148174	11	c.T1170C	p.H390H		0.04918033	174	9	SNV
YHIM-07	Synergy	B3GALNT1	chr3	160804010	160804010	T	G	exonic	nonsynonymous SNV	NM_033168	4	c.A533C	p.K178T		0.32432432	50	24	SNV
YHIM-07	Synergy	B9D1	chr17	19266018	19266018	G	C	exonic	nonsynonymous SNV	NM_001243473	1	c.C17G	p.S6W		0.41538462	114	81	SNV
YHIM-07	Synergy	BANK1	chr4	102791687	102791687	C	G	exonic	synonymous SNV	NM_001127507	4	c.C390G	p.V130V		0.47727273	92	84	SNV
YHIM-07	Synergy	BCHE	chr3	165547425	165547425	T	C	exonic	nonsynonymous SNV	NM_000055	2	c.A1397G	p.H466R		0.32134832	302	143	SNV
YHIM-07	Synergy	BHLHA9	chr17	1174243	1174243	G	T	exonic	nonsynonymous SNV	NM_001164405	1	c.G386T	p.G129V		0.5625	35	45	SNV
YHIM-07	Synergy	BIN2	chr12	51689626	51689626	C	G	exonic	nonsynonymous SNV	NM_001290008	8	c.G715C	p.E239Q		0.48780488	21	20	SNV
YHIM-07	Synergy	BLVRA	chr7	43830892	43830892	A	C	exonic	nonsynonymous SNV	NM_000712	4	c.A179C	p.E60A		0.27906977	93	36	SNV
YHIM-07	Synergy	BPIFB6	chr20	31631147	31631147	A	-	exonic	frameshift deletion	NM_174897	14	c.1303delA	p.N435fs					Indel
YHIM-07	Synergy	C6orf62	chr6	24718792	24718792	A	G	exonic	synonymous SNV	NM_030939	1	c.T105C	p.Y35Y		0.03333333	145	5	SNV
YHIM-07	Synergy	C9orf172	chr9	139741437	139741437	C	G	exonic	synonymous SNV	NM_001080482	1	c.C2571G	p.P857P		0.41269841	74	52	SNV
YHIM-07	Synergy	CACNG8	chr19	54485653	54485653	T	C	exonic	synonymous SNV	NM_031895	4	c.T828C	p.S276S		0.06382979	44	3	SNV
YHIM-07	Synergy	CADM3	chr1	159159611	159159611	G	T	exonic	stopgain	NM_021189	2	c.G139T	p.E47X		0.2972973	78	33	SNV
YHIM-07	Synergy	CAMK2N2	chr3	183978942	183978942	T	G	exonic	synonymous SNV	NM_033259	1	c.A132C	p.R44R		0.35148515	131	71	SNV
YHIM-07	Synergy	CAMSAP2	chr1	200818970	200818970	G	C	exonic	nonsynonymous SNV	NM_001297708	11	c.G3025C	p.D1009H		0.31486146	272	125	SNV
YHIM-07	Synergy	CAND1	chr12	67699490	67699490	A	G	exonic	nonsynonymous SNV	NM_018448	10	c.A2042G	p.Y681C		0.99593496	1	245	SNV
YHIM-07	Synergy	CAPN6	chrX	110492210	110492210	G	C	exonic	nonsynonymous SNV	NM_014289	9	c.C1234G	p.R412G		1	0	143	SNV
YHIM-07	Synergy	CASK	chrX	41712459	41712459	T	C	exonic	synonymous SNV	NM_001126054	2	c.A81G	p.R27R		0.05882353	80	5	SNV
YHIM-07	Synergy	CATSPER1	chr11	65793355	65793355	C	T	exonic	nonsynonymous SNV	NM_053054	1	c.G496A	p.V166M		0.4661017	126	110	SNV
YHIM-07	Synergy	CCDC109B	chr4	110605767	110605767	A	G	exonic	nonsynonymous SNV	NM_017918	6	c.A781G	p.M261V		0.625	21	35	SNV
YHIM-07	Synergy	CCDC138	chr2	109489910	109489910	C	G	exonic	nonsynonymous SNV	NM_001303106	14	c.C749G	p.S250C		0.47131148	129	115	SNV
YHIM-07	Synergy	CCDC150	chr2	197521724	197521724	C	T	exonic	nonsynonymous SNV	NM_001080539	4	c.C440T	p.S147F		0.2122449	193	52	SNV
YHIM-07	Synergy	CCDC182	chr17	55822631	55822631	C	T	exonic	nonsynonymous SNV	NM_001282544	1	c.G3A	p.M1I		0.53571429	39	45	SNV
YHIM-07	Synergy	CCDC88A	chr2	55544715	55544715	C	G	exonic	nonsynonymous SNV	NM_001135597	20	c.G3584C	p.R1195T		0.57352941	29	39	SNV

YHIM-07	Synergy	CCPG1	chr15	55653113	55653113	C	G	exonic	nonsynonymous SNV	NM_001204450	8	c.G858C	p.W286C		0.6091954	34	53	SNV
YHIM-07	Synergy	CD101	chr1	117556029	117556029	G	A	exonic	synonymous SNV	NM_001256106	4	c.G843A	p.V281V		1	0	70	SNV
YHIM-07	Synergy	CDC20	chr1	43825460	43825460	G	C	exonic	nonsynonymous SNV	NM_001255	4	c.G395C	p.R132P		0.58333333	55	77	SNV
YHIM-07	Synergy	CDH22	chr20	44879859	44879859	C	T	exonic	synonymous SNV	NM_021248	2	c.G75A	p.L25L		0.12195122	108	15	SNV
YHIM-07	Synergy	CELSR1	chr22	46930635	46930635	G	A	exonic	synonymous SNV	NM_014246	1	c.C2433T	p.L811L		0.48880597	274	262	SNV
YHIM-07	Synergy	CELSR3	chr3	48691776	48691776	C	G	exonic	nonsynonymous SNV	NM_001407	7	c.G5098C	p.D1700H		0.44525547	152	122	SNV
YHIM-07	Synergy	CEP131	chr17	79177345	79177345	G	A	exonic	nonsynonymous SNV	NM_001009811	6	c.C578T	p.S193L		0.48603352	92	87	SNV
YHIM-07	Synergy	CES1	chr16	55853481	55853481	G	A	exonic	nonsynonymous SNV	NM_001025194	7	c.C869T	p.T290M		0.05172414	110	6	SNV
YHIM-07	Synergy	CES4A	chr16	67043016	67043016	C	G	exonic	nonsynonymous SNV	NM_001190201	12	c.C1363G	p.Q455E		0.41975309	47	34	SNV
YHIM-07	Synergy	CFAP46	chr10	134748307	134748307	C	G	exonic	nonsynonymous SNV	NM_001200049	8	c.G815C	p.R272T		0.47959184	51	47	SNV
YHIM-07	Synergy	CFAP65	chr2	219888029	219888029	G	A	exonic	nonsynonymous SNV	NM_194302	16	c.C2720T	p.S907L		0.4875	82	78	SNV
YHIM-07	Synergy	CFAP69	chr7	89906434	89906434	A	T	exonic	nonsynonymous SNV	NM_001039706	10	c.A1032T	p.E344D		0.42857143	8	6	SNV
YHIM-07	Synergy	CGNL1	chr15	57731497	57731497	C	T	exonic	nonsynonymous SNV	NM_032866	2	c.C1300T	p.R434C		0.71914132	157	402	SNV
YHIM-07	Synergy	CHN1	chr2	175779798	175779798	G	C	exonic	nonsynonymous SNV	NM_001822	5	c.C248G	p.T83S	CCG	0.43209877	46	35	SNV
YHIM-07	Synergy	CHPF2	chr7	150935248	150935248	G	C	exonic	synonymous SNV	NM_019015	4	c.G1800C	p.G600G		0.35784314	393	219	SNV
YHIM-07	Synergy	CHST2	chr3	142840789	142840789	C	T	exonic	synonymous SNV	NM_004267	2	c.C1131T	p.G377G		0.08606557	223	21	SNV
YHIM-07	Synergy	CHST2	chr3	142841248	142841248	C	G	exonic	synonymous SNV	NM_004267	2	c.C1590G	p.L530L		0.23913044	105	33	SNV
YHIM-07	Synergy	CILP	chr15	65491294	65491294	C	A	exonic	nonsynonymous SNV	NM_003613	9	c.G1330T	p.G444W		0.29902913	361	154	SNV
YHIM-07	Synergy	CITED2	chr6	139694328	139694328	C	T	exonic	nonsynonymous SNV	NM_001168388	2	c.G754A	p.E252K		0.5	92	92	SNV
YHIM-07	Synergy	CLCN3	chr4	170634250	170634250	G	A	exonic	nonsynonymous SNV	NM_001243372	11	c.G2089A	p.E697K		0.49568966	117	115	SNV
YHIM-07	Synergy	CLCNKA	chr1	16353849	16353849	C	T	exonic	nonsynonymous SNV	NM_001257139	7	c.C571T	p.R191W		0.0754717	49	4	SNV
YHIM-07	Synergy	CLDN18	chr3	137749971	137749971	C	T	exonic	synonymous SNV	NM_001002026	5	c.C774T	p.H258H		0.0952381	57	6	SNV
YHIM-07	Synergy	CLIC5	chr6	45882072	45882072	C	G	exonic	nonsynonymous SNV	NM_001114086	5	c.G958C	p.D320H		0.55223881	60	74	SNV
YHIM-07	Synergy	CLPTM1L	chr5	1339066	1339066	A	G	exonic	nonsynonymous SNV	NM_030782	4	c.T508C	p.W170R		0.31007752	178	80	SNV
YHIM-07	Synergy	CLRN1	chr3	150645966	150645966	C	T	exonic	nonsynonymous SNV	NM_052995	3	c.G228A	p.M76I		0.29032258	88	36	SNV
YHIM-07	Synergy	CMYA5	chr5	79029752	79029752	A	T	exonic	nonsynonymous SNV	NM_153610	2	c.A5164T	p.S1722C		0.12093023	189	26	SNV
YHIM-07	Synergy	CMYA5	chr5	79028672	79028672	G	A	exonic	nonsynonymous SNV	NM_153610	2	c.G4084A	p.D1362N		0.27509294	195	74	SNV
YHIM-07	Synergy	CNPPD1	chr2	220039824	220039824	T	C	exonic	synonymous SNV	NM_015680	4	c.A267G	p.A89A		0.46	81	69	SNV
YHIM-07	Synergy	CNTD2	chr19	40732514	40732514	G	T	exonic	nonsynonymous SNV	NM_024877	1	c.C35A	p.S12Y		0.26229508	45	16	SNV
YHIM-07	Synergy	CNTN3	chr3	74535628	74535628	C	T	exonic	nonsynonymous SNV	NM_020872	3	c.G337A	p.E113K		0.10144928	62	7	SNV
YHIM-07	Synergy	COL3A1	chr2	189873712	189873712	C	T	exonic	synonymous SNV	NM_000090	48	c.C3588T	p.C1196C		0.50704225	35	36	SNV
YHIM-07	Synergy	COL6A5	chr3	130187875	130187875	C	G	exonic	nonsynonymous SNV	NM_001278298	38	c.C7027G	p.P2343A		0.27748691	138	53	SNV
YHIM-07	Synergy	CPED1	chr7	120767230	120767230	C	T	exonic	synonymous SNV	NM_001105533	9	c.C1221T	p.F407F		0.05825243	97	6	SNV
YHIM-07	Synergy	CPS1	chr2	211507264	211507264	C	G	exonic	nonsynonymous SNV	NM_001122634	15	c.C1663G	p.L555V		0.49509804	103	101	SNV
YHIM-07	Synergy	CRISP3	chr6	49696452	49696452	G	A	exonic	synonymous SNV	NM_001190986	8	c.C798T	p.S266S		0.50755287	163	168	SNV
YHIM-07	Synergy	CSF1R	chr5	149460339	149460339	A	G	exonic	nonsynonymous SNV	NM_001288705	2	c.T298C	p.Y100H		0.38053097	70	43	SNV
YHIM-07	Synergy	CSMD1	chr8	4494966	4494966	C	T	exonic	nonsynonymous SNV	NM_033225	2	c.G200A	p.R67H		0.57236842	65	87	SNV
YHIM-07	Synergy	CSMD3	chr8	113364680	113364680	C	A	exonic	nonsynonymous SNV	NM_052900	38	c.G5908T	p.V1970L		0.61904762	24	39	SNV
YHIM-07	Synergy	CTDP1	chr18	77474834	77474834	C	T	exonic	synonymous SNV	NM_001202504	8	c.C1017T	p.S339S		0.44268775	141	112	SNV
YHIM-07	Synergy	CXXC4	chr4	105412143	105412143	G	A	exonic	stopgain	NM_025212	2	c.C817T	p.Q273X		0.49253731	102	99	SNV
YHIM-07	Synergy	DAG1	chr3	49569982	49569982	C	T	exonic	nonsynonymous SNV	NM_001177639	3	c.C2038T	p.R680W		0.55477032	126	157	SNV
YHIM-07	Synergy	DARS	chr2	136718971	136718971	A	T	exonic	synonymous SNV	NM_001293312	3	c.T15A	p.A5A		0.06188925	288	19	SNV
YHIM-07	Synergy	DCAF8L2	chrX	27765400	27765408	GAGC	-	exonic	nonframeshift deletion	NM_001136533	1	c.388_396del	p.130_132del					Indel
YHIM-07	Synergy	DDX25	chr11	125791184	125791184	C	T	exonic	nonsynonymous SNV	NM_013264	11	c.C1300T	p.R434W		0.53846154	24	28	SNV
YHIM-07	Synergy	DIEXF	chr1	210010185	210010185	C	T	exonic	stopgain	NM_014388	6	c.C691T	p.Q231X		0.29457364	273	114	SNV
YHIM-07	Synergy	DMD	chrX	32383256	32383256	C	T	exonic	nonsynonymous SNV	NM_004011	7	c.G883A	p.E295K		1	0	164	SNV
YHIM-07	Synergy	DNAJC10	chr2	183605075	183605075	A	G	exonic	nonsynonymous SNV	NM_001271581	11	c.A899G	p.N300S		0.5	36	36	SNV
YHIM-07	Synergy	DNAJC7	chr17	40142386	40142386	C	T	exonic	nonsynonymous SNV	NM_001144766	6	c.G327A	p.M109I		0.29931973	103	44	SNV
YHIM-07	Synergy	DNAJC7	chr17	40142373	40142373	C	T	exonic	nonsynonymous SNV	NM_001144766	6	c.G340A	p.E114K		0.25954199	97	34	SNV
YHIM-07	Synergy	DNMT3L	chr21	45681066	45681066	G	C	exonic	stopgain	NM_013369	2	c.C77G	p.S26X		0.13580247	70	11	SNV
YHIM-07	Synergy	DRC7	chr16	57756741	57756741	G	A	exonic	nonsynonymous SNV	NM_001289163	9	c.G1201A	p.E401K		0.48128342	97	90	SNV
YHIM-07	Synergy	E4F1	chr16	2273649	2273649	C	A	exonic	nonsynonymous SNV	NM_001288776	1	c.C35A	p.A12D		0.44736842	21	17	SNV
YHIM-07	Synergy	EFNB1	chrX	68060483	68060483	T	C	exonic	nonsynonymous SNV	NM_004429	5	c.T1027C	p.Y343H		1	0	53	SNV
YHIM-07	Synergy	EIF3A	chr10	120801788	120801788	C	A	exonic	nonsynonymous SNV	NM_003750	19	c.G3244T	p.G1082C		0.43902439	23	18	SNV
YHIM-07	Synergy	EMILIN2	chr18	2892402	2892402	G	A	exonic	synonymous SNV	NM_032048	4	c.G2277A	p.K759K		0.18796993	108	25	SNV
YHIM-07	Synergy	EPHA2	chr1	16475407	16475407	C	A	exonic	stopgain	NM_004431	3	c.G289T	p.E97X		0.49321267	112	109	SNV
YHIM-07	Synergy	EPHA2	chr1	16451711	16451711	C	T	exonic	synonymous SNV	NM_004431	17	c.G2930A	p.X977X		0.42473118	107	79	SNV
YHIM-07	Synergy	ERBB3	chr12	56495664	56495664	C	T	exonic	nonsynonymous SNV	NM_001982	28	c.C3854T	p.S1285F	CCG	0.52095808	80	87	SNV
YHIM-07	Synergy	EXOC4	chr7	133749206	133749206	G	C	exonic	nonsynonymous SNV	NM_021807	18	c.G2850C	p.Q950H		0.33128834	218	108	SNV
YHIM-07	Synergy	EXOSC1	chr10	99203048	99203048	C	T	exonic	synonymous SNV	NM_016046	3	c.G168A	p.V56V		0.51256281	97	102	SNV
YHIM-07	Synergy	FAM120A	chr9	96294642	96294642	T	C	exonic	nonsynonymous SNV	NM_001286723	10	c.T1940C	p.F647S		0.03521127	137	5	SNV
YHIM-07	Synergy	FAM135A	chr6	71234834	71234834	C	T	exonic	stopgain	NM_001162529	13	c.C2047T	p.R683X		0.46666667	104	91	SNV

YHIM-07	Synergy	FAM135B	chr8	139164364	139164364	T	A	exonic	nonsynonymous SNV	NM_015912	13	c.A2354T	p.D785V		0.51239669	59	62	SNV
YHIM-07	Synergy	FAM135B	chr8	139163990	139163990	C	G	exonic	nonsynonymous SNV	NM_015912	13	c.G2728C	p.D910H		0.49009901	103	99	SNV
YHIM-07	Synergy	FAM193B	chr5	176959575	176959575	C	T	exonic	nonsynonymous SNV	NM_001190946	5	c.G1144A	p.E382K		0.34010152	130	67	SNV
YHIM-07	Synergy	FAM216A	chr12	110924130	110924130	C	A	exonic	nonsynonymous SNV	NM_013300	4	c.C307A	p.H103N		1	0	130	SNV
YHIM-07	Synergy	FAM46B	chr1	27332578	27332578	C	T	exonic	nonsynonymous SNV	NM_052943	2	c.G1135A	p.E379K		0.12322275	185	26	SNV
YHIM-07	Synergy	FAM73B	chr9	131811668	131811668	A	G	exonic	synonymous SNV	NM_032809	5	c.A429G	p.A143A		0.28395062	232	92	SNV
YHIM-07	Synergy	FAM83G	chr17	18874872	18874872	G	A	exonic	nonsynonymous SNV	NM_001039999	6	c.C2272T	p.P758S		0.47904192	87	80	SNV
YHIM-07	Synergy	FAM83G	chr17	18882981	18882981	G	C	exonic	synonymous SNV	NM_001039999	4	c.C696G	p.L232L		0.46153846	35	30	SNV
YHIM-07	Synergy	FAR1	chr11	13736138	13736138	T	C	exonic	synonymous SNV	NM_032228	9	c.T1038C	p.H346H		0.11764706	60	8	SNV
YHIM-07	Synergy	FARP2	chr2	242423713	242423713	G	T	exonic	synonymous SNV	NM_014808	21	c.G2388T	p.R796R		0.53431373	95	109	SNV
YHIM-07	Synergy	FAT1	chr4	187629328	187629328	C	A	exonic	stopgain	NM_005245	2	c.G1654T	p.E552X	CCG	0.50896057	137	142	SNV
YHIM-07	Synergy	FAT2	chr5	150892086	150892086	C	T	exonic	nonsynonymous SNV	NM_001447	20	c.G11545A	p.D3849N		0.46357616	162	140	SNV
YHIM-07	Synergy	FAT3	chr11	92498074	92498074	G	A	exonic	synonymous SNV	NM_001008781	5	c.G4014A	p.Q1338Q		0.51111111	22	23	SNV
YHIM-07	Synergy	FBF1	chr17	73908387	73908387	G	A	exonic	synonymous SNV	NM_001080542	28	c.C3204T	p.A1068A		0.41860465	25	18	SNV
YHIM-07	Synergy	FBXL20	chr17	37457274	37457274	-	A	exonic	stopgain	NM_001184906	4	c.213dupT	p.D72_F73delinsX					Indel
YHIM-07	Synergy	FBXO41	chr2	73487554	73487554	C	T	exonic	synonymous SNV	NM_001080410	10	c.G2412A	p.A804A		0.37383178	67	40	SNV
YHIM-07	Synergy	FER	chr5	108134085	108134085	T	G	exonic	nonsynonymous SNV	NM_005246	3	c.T202G	p.S68A		0.24	19	6	SNV
YHIM-07	Synergy	FGF10	chr5	44388754	44388754	A	T	exonic	nonsynonymous SNV	NM_004465	1	c.T31A	p.S11T		0.3442623	80	42	SNV
YHIM-07	Synergy	FIG4	chr6	110064947	110064947	C	G	exonic	nonsynonymous SNV	NM_014845	10	c.C1109G	p.S370C		0.50660793	112	115	SNV
YHIM-07	Synergy	FN3KRP	chr17	80684438	80684438	C	A	exonic	nonsynonymous SNV	NM_024619	5	c.C551A	p.S184Y		0.14851485	86	15	SNV
YHIM-07	Synergy	FSTL4	chr5	132535210	132535210	G	A	exonic	synonymous SNV	NM_015082	16	c.C2106T	p.V702V		0.23121387	133	40	SNV
YHIM-07	Synergy	FXR1	chr3	180688011	180688011	G	A	exonic	nonsynonymous SNV	NM_001013438	15	c.G1468A	p.D490N		0.26206897	214	76	SNV
YHIM-07	Synergy	FYN	chr6	112021464	112021464	A	G	exonic	synonymous SNV	NM_002037	9	c.T705C	p.A235A		0.04098361	117	5	SNV
YHIM-07	Synergy	GABRE	chrX	151128393	151128393	G	A	exonic	synonymous SNV	NM_004961	6	c.C702T	p.I234I		1	0	42	SNV
YHIM-07	Synergy	GAD1	chr2	171702086	171702086	G	C	exonic	nonsynonymous SNV	NM_000817	8	c.G822C	p.K274N		0.43333333	51	39	SNV
YHIM-07	Synergy	GADL1	chr3	30842509	30842509	G	C	exonic	nonsynonymous SNV	NM_207359	12	c.C1122G	p.S374R		0.50649351	38	39	SNV
YHIM-07	Synergy	GAL	chr11	68456340	68456340	G	C	exonic	nonsynonymous SNV	NM_015973	5	c.G247C	p.E83Q		0.51327434	165	174	SNV
YHIM-07	Synergy	GFRAL	chr6	55198624	55198624	-	A	exonic	frameshift insertion	NM_207410	3	c.199dupA	p.C66fs					Indel
YHIM-07	Synergy	GOLGB1	chr3	121413035	121413035	G	C	exonic	stopgain	NM_001256488	12	c.C6095G	p.S2032X		0.10548523	424	50	SNV
YHIM-07	Synergy	GPRASP1	chrX	101912217	101912217	C	G	exonic	nonsynonymous SNV	NM_001099411	3	c.C3376G	p.R1126G		1	0	105	SNV
YHIM-07	Synergy	GRIK1	chr21	30949390	30949390	G	T	exonic	nonsynonymous SNV	NM_175611	13	c.C1979A	p.T660K		0.51408451	69	73	SNV
YHIM-07	Synergy	HIST1H4L	chr6	27841146	27841146	G	C	exonic	stopgain	NM_003546	1	c.C143G	p.S48X		0.48373984	127	119	SNV
YHIM-07	Synergy	HIST2H2AC	chr1	149858741	149858741	G	C	exonic	nonsynonymous SNV	NM_003517	1	c.G217C	p.D73H		0.38271605	50	31	SNV
YHIM-07	Synergy	HIST2H2AC	chr1	149858806	149858806	G	C	exonic	synonymous SNV	NM_003517	1	c.G282C	p.L94L		0.23026316	117	35	SNV
YHIM-07	Synergy	HMGB2	chr4	174254317	174254317	C	T	exonic	nonsynonymous SNV	NM_001130689	2	c.G199A	p.D67N		0.1948718	157	38	SNV
YHIM-07	Synergy	HOOK2	chr19	12881838	12881838	C	T	exonic	synonymous SNV	NM_001100176	10	c.G810A	p.E270E		0.51785714	27	29	SNV
YHIM-07	Synergy	HOXC8	chr12	54403461	54403461	A	G	exonic	synonymous SNV	NM_022658	1	c.A393G	p.Q131Q		0.03365385	201	7	SNV
YHIM-07	Synergy	HR	chr8	21976569	21976569	G	A	exonic	nonsynonymous SNV	NM_005144	16	c.C3107T	p.S1036L		0.49685535	80	79	SNV
YHIM-07	Synergy	HS3ST3A1	chr17	13503902	13503902	C	G	exonic	nonsynonymous SNV	NM_006042	1	c.G545C	p.G182A		0.42016807	69	50	SNV
YHIM-07	Synergy	HSD17B13	chr4	88226371	88226371	C	T	exonic	synonymous SNV	NM_001136230	6	c.G794A	p.X265X		0.45588235	37	31	SNV
YHIM-07	Synergy	IFNA10	chr9	21206569	21206569	C	A	exonic	synonymous SNV	NM_002171	1	c.G528T	p.S176S		0.05	76	4	SNV
YHIM-07	Synergy	IFT172	chr2	27680622	27680622	C	G	exonic	nonsynonymous SNV	NM_015662	29	c.G3112C	p.E1038Q		0.52941177	64	72	SNV
YHIM-07	Synergy	IK	chr5	140038603	140038603	C	T	exonic	nonsynonymous SNV	NM_006083	12	c.C1030T	p.R344W		0.56989247	80	106	SNV
YHIM-07	Synergy	IMPG2	chr3	100963168	100963168	A	G	exonic	synonymous SNV	NM_016247	13	c.T2007C	p.H669H		0.33419689	257	129	SNV
YHIM-07	Synergy	INPP5J	chr22	31520906	31520906	C	T	exonic	nonsynonymous SNV	NM_001284285	2	c.C181T	p.P61S		0.18803419	190	44	SNV
YHIM-07	Synergy	IQGAP2	chr5	75936775	75936775	C	T	exonic	synonymous SNV	NM_001285460	16	c.C1791T	p.V597V		0.4109589	86	60	SNV
YHIM-07	Synergy	IVL	chr1	152882978	152882978	C	T	exonic	synonymous SNV	NM_005547	2	c.C705T	p.L235L		0.15625	27	5	SNV
YHIM-07	Synergy	JRKL	chr11	96125206	96125206	G	A	exonic	nonsynonymous SNV	NM_003772	1	c.G1393A	p.E465K		0.18709677	126	29	SNV
YHIM-07	Synergy	KAT6B	chr10	76781035	76781035	G	A	exonic	nonsynonymous SNV	NM_001256468	15	c.G2464A	p.E822K	CCG	0.16774194	129	26	SNV
YHIM-07	Synergy	KBTBD11	chr8	1951202	1951202	C	T	exonic	nonsynonymous SNV	NM_014867	2	c.C1844T	p.P615L		0.38888889	11	7	SNV
YHIM-07	Synergy	KCNMA1	chr10	79010998	79010998	G	T	exonic	nonsynonymous SNV	NM_001271518	2	c.C395A	p.A132D		0.50704225	105	108	SNV
YHIM-07	Synergy	KCNS1	chr20	43726891	43726891	C	T	exonic	synonymous SNV	NM_002251	4	c.G522A	p.P174P		0.31446541	218	100	SNV
YHIM-07	Synergy	KDM7A	chr7	139824499	139824499	C	T	exonic	nonsynonymous SNV	NM_030647	7	c.G973A	p.E325K		0.30149254	234	101	SNV
YHIM-07	Synergy	KIF14	chr1	200534582	200534582	G	C	exonic	nonsynonymous SNV	NM_001305792	22	c.C2404G	p.Q802E		0.14285714	30	5	SNV
YHIM-07	Synergy	KIF1A	chr2	241683440	241683440	A	T	exonic	nonsynonymous SNV	NM_004321	30	c.T3200A	p.I1067N		0.49640288	140	138	SNV
YHIM-07	Synergy	KRI1	chr19	10664737	10664737	C	T	exonic	nonsynonymous SNV	NM_023008	19	c.G2020A	p.E674K		0.54166667	99	117	SNV
YHIM-07	Synergy	LAMA4	chr6	112450191	112450191	G	A	exonic	nonsynonymous SNV	NM_001105206	31	c.C4220T	p.S1407L		0.59310345	59	86	SNV
YHIM-07	Synergy	LARS	chr5	145524081	145524081	C	G	exonic	nonsynonymous SNV	NM_020117	17	c.G1609C	p.E537Q		0.40947075	212	147	SNV
YHIM-07	Synergy	LGR4	chr11	27389539	27389539	C	A	exonic	stopgain	NM_018490	18	c.G2731T	p.E911X		0.44782609	127	103	SNV
YHIM-07	Synergy	LIN9	chr1	226475434	226475434	C	T	exonic	nonsynonymous SNV	NM_001270409	4	c.G272A	p.R91H		0.23076923	40	12	SNV
YHIM-07	Synergy	LINS1	chr15	101112208	101112208	G	A	exonic	synonymous SNV	NM_001040616	6	c.C1285T	p.L429L		0.30222222	314	136	SNV
YHIM-07	Synergy	LMOD2	chr7	123302889	123302889	C	T	exonic	nonsynonymous SNV	NM_207163	2	c.C1249T	p.P417S		0.3030303	23	10	SNV

YHIM-07	Synergy	LOC1001343	chr17	71746825	71746825	C	A	exonic	nonsynonymous SNV	NM_001278587	3	c.C172A	p.P58T		0.57037037	58	77	SNV
YHIM-07	Synergy	LOC1001343	chr17	71746826	71746826	C	G	exonic	nonsynonymous SNV	NM_001278587	3	c.C173G	p.P58R		0.56390977	58	75	SNV
YHIM-07	Synergy	LPIN2	chr18	2937807	2937807	C	T	exonic	nonsynonymous SNV	NM_014646	7	c.G1051A	p.E351K		0.47126437	92	82	SNV
YHIM-07	Synergy	LRP8	chr1	53792551	53792551	C	T	exonic	nonsynonymous SNV	NM_001018054	2	c.G238A	p.D80N		0.23114355	316	95	SNV
YHIM-07	Synergy	LRPPRC	chr2	44174495	44174495	T	C	exonic	synonymous SNV	NM_133259	20	c.A1980G	p.T660T		0.49152542	30	29	SNV
YHIM-07	Synergy	LRRC16A	chr6	25420357	25420357	C	T	exonic	stopgain	NM_001173977	3	c.C154T	p.R52X		0.43292683	93	71	SNV
YHIM-07	Synergy	LRRC16B	chr14	24538611	24538611	T	A	exonic	stoploss	NM_138360	40	c.T4117A	p.X1373R		0.46938776	52	46	SNV
YHIM-07	Synergy	MALRD1	chr10	19417224	19417224	T	C	exonic	nonsynonymous SNV	NM_001142308	8	c.T1018C	p.S340P		0.45652174	75	63	SNV
YHIM-07	Synergy	MAN1B1	chr9	139990831	139990831	G	A	exonic	nonsynonymous SNV	NM_016219	4	c.G608A	p.R203K		0.390625	39	25	SNV
YHIM-07	Synergy	MAP1B	chr5	71493046	71493046	C	G	exonic	synonymous SNV	NM_005909	5	c.C3864G	p.V1288V		0.1	162	18	SNV
YHIM-07	Synergy	MATR3	chr5	138643914	138643914	A	G	exonic	synonymous SNV	NM_001194955	2	c.A810G	p.R270R		0.23188406	212	64	SNV
YHIM-07	Synergy	MCM3	chr6	52132692	52132692	G	C	exonic	nonsynonymous SNV	NM_001270472	13	c.C2073G	p.S691R		0.543379	100	119	SNV
YHIM-07	Synergy	MCM3AP	chr21	47660805	47660805	C	G	exonic	synonymous SNV	NM_003906	26	c.G5553C	p.L1851L		0.46022727	95	81	SNV
YHIM-07	Synergy	MED13	chr17	60062147	60062147	C	T	exonic	nonsynonymous SNV	NM_005121	14	c.G2556A	p.M852I		0.34615385	68	36	SNV
YHIM-07	Synergy	MED17	chr11	93517717	93517717	C	T	exonic	nonsynonymous SNV	NM_004268	1	c.C38T	p.S13L		0.5212766	90	98	SNV
YHIM-07	Synergy	MED23	chr6	131931346	131931346	T	-	exonic	frameshift deletion	NM_001270521	11	c.917delA	p.D306fs					Indel
YHIM-07	Synergy	MED24	chr17	38178678	38178678	C	T	exonic	nonsynonymous SNV	NM_001079518	21	c.G2453A	p.R818H		0.5	81	81	SNV
YHIM-07	Synergy	MED4	chr13	48651420	48651420	T	A	exonic	nonsynonymous SNV	NM_001270629	7	c.A530T	p.Q177L		0.50306749	81	82	SNV
YHIM-07	Synergy	MEGF9	chr9	123476074	123476074	G	A	exonic	nonsynonymous SNV	NM_001080497	1	c.C563T	p.T188I		0.40540541	44	30	SNV
YHIM-07	Synergy	MFSD9	chr2	103335455	103335455	G	T	exonic	synonymous SNV	NM_032718	6	c.C849A	p.S283S		0.45770065	250	211	SNV
YHIM-07	Synergy	MICU3	chr8	16956053	16956053	C	A	exonic	nonsynonymous SNV	NM_181723	9	c.C975A	p.D325E		0.43534483	131	101	SNV
YHIM-07	Synergy	MOB3B	chr9	27359208	27359208	G	T	exonic	nonsynonymous SNV	NM_024761	3	c.C445A	p.Q149K		0.17061611	350	72	SNV
YHIM-07	Synergy	MPP3	chr17	41907398	41907398	C	T	exonic	synonymous SNV	NM_001932	6	c.G300A	p.L100L		0.48214286	58	54	SNV
YHIM-07	Synergy	MRGPRX4	chr11	18195162	18195162	G	A	exonic	nonsynonymous SNV	NM_054032	1	c.G359A	p.R120H		0.43153527	137	104	SNV
YHIM-07	Synergy	MROH7	chr1	55139427	55139427	G	T	exonic	synonymous SNV	NM_001291332	7	c.G303T	p.V101V		0.44715447	204	165	SNV
YHIM-07	Synergy	MUC16	chr19	9090181	9090181	G	C	exonic	nonsynonymous SNV	NM_024690	1	c.C1634G	p.T545R		0.50364964	136	138	SNV
YHIM-07	Synergy	MUC16	chr19	9064089	9064089	G	T	exonic	stopgain	NM_024690	3	c.C23357A	p.S7786X		0.49152542	150	145	SNV
YHIM-07	Synergy	MUC4	chr3	195506974	195506974	G	A	exonic	nonsynonymous SNV	NM_018406	2	c.C11477T	p.P3826L		0.5	2	2	SNV
YHIM-07	Synergy	MUC5B	chr11	1257763	1257763	G	A	exonic	nonsynonymous SNV	NM_002458	24	c.G3028A	p.V1010M		0.43529412	96	74	SNV
YHIM-07	Synergy	MYH7B	chr20	33588861	33588861	C	T	exonic	nonsynonymous SNV	NM_020884	41	c.C5501T	p.T1834M		0.13333333	52	8	SNV
YHIM-07	Synergy	MYO9A	chr15	72143644	72143644	G	A	exonic	synonymous SNV	NM_006901	37	c.C6531T	p.L2177L		0.42758621	83	62	SNV
YHIM-07	Synergy	MYOC	chr1	171605179	171605179	G	A	exonic	synonymous SNV	NM_000261	3	c.C1401T	p.F467F		0.26777251	309	113	SNV
YHIM-07	Synergy	MYOM2	chr8	2046784	2046784	A	G	exonic	nonsynonymous SNV	NM_003970	19	c.A2411G	p.K804R		0.5	27	27	SNV
YHIM-07	Synergy	NAB2	chr12	57485330	57485330	A	G	exonic	nonsynonymous SNV	NM_005967	2	c.A506G	p.K169R	CCG	0.99224806	1	128	SNV
YHIM-07	Synergy	NACC2	chr9	138908211	138908211	G	A	exonic	synonymous SNV	NM_144653	3	c.C951T	p.L317L		0.36363636	42	24	SNV
YHIM-07	Synergy	NACC2	chr9	138908169	138908169	G	C	exonic	synonymous SNV	NM_144653	3	c.C993G	p.R331R		0.35211268	46	25	SNV
YHIM-07	Synergy	NAMPT	chr7	105912978	105912978	C	T	exonic	nonsynonymous SNV	NM_005746	4	c.G445A	p.E149K		0.1300813	107	16	SNV
YHIM-07	Synergy	NANS	chr9	100840618	100840618	C	T	exonic	nonsynonymous SNV	NM_018946	4	c.C592T	p.R198W		0.3902439	50	32	SNV
YHIM-07	Synergy	NCK2	chr2	106471630	106471630	G	A	exonic	synonymous SNV	NM_001004720	2	c.G111A	p.T37T		0.46428571	60	52	SNV
YHIM-07	Synergy	NEDD4	chr15	56122781	56122781	G	A	exonic	nonsynonymous SNV	NM_198400	21	c.C3572T	p.S1191L		0.39153439	115	74	SNV
YHIM-07	Synergy	NF2	chr22	30057209	30057209	G	T	exonic	stopgain	NM_181830	6	c.G442T	p.E148X	CCG	0.42857143	64	48	SNV
YHIM-07	Synergy	NFAT5	chr16	69681481	69681481	G	T	exonic	synonymous SNV	NM_006599	3	c.G750T	p.A250A		0.05223881	127	7	SNV
YHIM-07	Synergy	NIPBL	chr5	36985390	36985390	C	G	exonic	nonsynonymous SNV	NM_015384	10	c.C2108G	p.P703R		0.30526316	66	29	SNV
YHIM-07	Synergy	NLRP14	chr11	7064041	7064041	G	C	exonic	nonsynonymous SNV	NM_176822	4	c.G784C	p.D262H		0.48044693	186	172	SNV
YHIM-07	Synergy	NOD1	chr7	30472770	30472770	C	G	exonic	nonsynonymous SNV	NM_006092	12	c.G2647C	p.E883Q		0.33829787	311	159	SNV
YHIM-07	Synergy	NOL4	chr18	31537341	31537341	T	G	exonic	synonymous SNV	NM_001198549	4	c.A522C	p.I174I		0.02990033	292	9	SNV
YHIM-07	Synergy	NOM1	chr7	156742654	156742654	C	T	exonic	nonsynonymous SNV	NM_138400	1	c.C223T	p.R75C		0.18390805	142	32	SNV
YHIM-07	Synergy	NOS1AP	chr1	162335242	162335242	G	C	exonic	nonsynonymous SNV	NM_001126060	1	c.G103C	p.E35Q		0.35319149	152	83	SNV
YHIM-07	Synergy	NR2F2	chr15	96875583	96875583	A	G	exonic	synonymous SNV	NM_021005	1	c.A249G	p.G83G		0.02961918	688	21	SNV
YHIM-07	Synergy	NR4A2	chr2	157186546	157186546	G	A	exonic	synonymous SNV	NM_006186	3	c.C153T	p.I51I		0.04285714	268	12	SNV
YHIM-07	Synergy	OCA2	chr15	28277237	28277237	C	T	exonic	synonymous SNV	NM_000275	3	c.G300A	p.L100L		0.26633166	146	53	SNV
YHIM-07	Synergy	OR10H1	chr19	15918812	15918812	G	A	exonic	synonymous SNV	NM_013940	1	c.C36T	p.F12F		0.43243243	63	48	SNV
YHIM-07	Synergy	OR52J3	chr11	5067867	5067867	C	A	exonic	nonsynonymous SNV	NM_001001916	1	c.C112A	p.L38I		0.44202899	77	61	SNV
YHIM-07	Synergy	OR56A1	chr11	6048116	6048116	C	A	exonic	nonsynonymous SNV	NM_001001917	1	c.G819T	p.K273N		0.50714286	69	71	SNV
YHIM-07	Synergy	OR8K1	chr11	56113986	56113986	A	T	exonic	nonsynonymous SNV	NM_001002907	1	c.A472T	p.S158C		0.41333333	132	93	SNV
YHIM-07	Synergy	OR8K1	chr11	56113987	56113987	G	T	exonic	nonsynonymous SNV	NM_001002907	1	c.G473T	p.S158I		0.40990991	131	91	SNV
YHIM-07	Synergy	OXR1	chr8	107719353	107719353	C	T	exonic	nonsynonymous SNV	NM_181354	7	c.C1583T	p.S528F		0.5131579	74	78	SNV
YHIM-07	Synergy	P2RY10	chrX	78216386	78216386	C	T	exonic	synonymous SNV	NM_198333	2	c.C369T	p.F123F		1	0	119	SNV
YHIM-07	Synergy	PACRG	chr6	163735989	163735989	C	T	exonic	synonymous SNV	NM_001080379	5	c.C744T	p.V248V		0.11940299	118	16	SNV
YHIM-07	Synergy	PAPLN	chr14	73720524	73720524	C	G	exonic	nonsynonymous SNV	NM_173462	11	c.C1076G	p.S359C		0.45374449	124	103	SNV
YHIM-07	Synergy	PAPOLB	chr7	4900370	4900370	C	G	exonic	nonsynonymous SNV	NM_020144	1	c.G1072C	p.E358Q		0.07620818	497	41	SNV
YHIM-07	Synergy	PATZ1	chr22	31740573	31740573	G	C	exonic	nonsynonymous SNV	NM_014323	1	c.C1016G	p.S339C		0.47058824	144	128	SNV

YHIM-07	Synergy	PAX2	chr10	102509645	102509645	C	T	exonic	synonymous SNV	NM_000278	2	c.C186T	p.H62H		0.07272727	51	4	SNV
YHIM-07	Synergy	PCDHA5	chr5	140201855	140201855	A	C	exonic	synonymous SNV	NM_018908	1	c.A495C	p.A165A		0.24102564	148	47	SNV
YHIM-07	Synergy	PCDHB13	chr5	140593752	140593752	C	G	exonic	synonymous SNV	NM_018933	1	c.C57G	p.L19L		0.2278481	122	36	SNV
YHIM-07	Synergy	PDHA2	chr4	96761911	96761911	G	T	exonic	stopgain	NM_005390	1	c.G610T	p.E204X		0.47410359	132	119	SNV
YHIM-07	Synergy	PDX1	chr13	28498790	28498790	G	A	exonic	synonymous SNV	NM_000209	2	c.G804A	p.S268S		0.47619048	11	10	SNV
YHIM-07	Synergy	PDZK1IP1	chr1	47650729	47650729	C	G	exonic	nonsynonymous SNV	NM_005764	3	c.G217C	p.D73H		0.48461539	201	189	SNV
YHIM-07	Synergy	PIWIL2	chr8	22137073	22137073	C	T	exonic	synonymous SNV	NM_001135721	2	c.C174T	p.S58S		0.3740458	82	49	SNV
YHIM-07	Synergy	PLCXD3	chr5	41382182	41382182	C	T	exonic	synonymous SNV	NM_001005473	2	c.G558A	p.E186E		0.32786885	123	60	SNV
YHIM-07	Synergy	PLEC	chr8	144999110	144999110	C	T	exonic	nonsynonymous SNV	NM_201378	31	c.G4945A	p.E1649K		0.38461539	32	20	SNV
YHIM-07	Synergy	PLPPR2	chr19	11473210	11473210	C	G	exonic	nonsynonymous SNV	NM_001170635	7	c.C610G	p.R204G		0.40361446	198	134	SNV
YHIM-07	Synergy	PLXNA3	chrX	153689801	153689801	C	T	exonic	synonymous SNV	NM_017514	3	c.C957T	p.T319T		1	0	88	SNV
YHIM-07	Synergy	PLXNB1	chr3	48456344	48456344	T	C	exonic	nonsynonymous SNV	NM_001130082	21	c.A4073G	p.D1358G		0.46616541	71	62	SNV
YHIM-07	Synergy	PLXND1	chr3	129279189	129279189	C	T	exonic	nonsynonymous SNV	NM_015103	31	c.G5117A	p.R1706H		0.3490566	207	111	SNV
YHIM-07	Synergy	POLR1B	chr2	113309449	113309449	G	T	exonic	nonsynonymous SNV	NM_001282776	3	c.G227T	p.R76M		0.42907801	161	121	SNV
YHIM-07	Synergy	POLR1B	chr2	113300239	113300239	C	A	exonic	synonymous SNV	NM_001137604	1	c.C168A	p.L56L		0.1902439	166	39	SNV
YHIM-07	Synergy	POMT1	chr9	134393864	134393864	C	A	exonic	synonymous SNV	NM_001136114	12	c.C954A	p.V318V		0.34527221	457	241	SNV
YHIM-07	Synergy	POU4F3	chr5	145719478	145719478	A	G	exonic	nonsynonymous SNV	NM_002700	2	c.A488G	p.H163R		0.38436482	189	118	SNV
YHIM-07	Synergy	POU6F2	chr7	39125590	39125590	T	C	exonic	nonsynonymous SNV	NM_001166018	3	c.T149C	p.V50A		0.12288136	207	29	SNV
YHIM-07	Synergy	POU6F2	chr7	39125588	39125588	T	C	exonic	synonymous SNV	NM_001166018	3	c.T147C	p.P49P		0.12396694	212	30	SNV
YHIM-07	Synergy	PPFIBP2	chr11	7614448	7614448	T	A	exonic	nonsynonymous SNV	NM_003621	4	c.T365A	p.I122K		0.5	120	120	SNV
YHIM-07	Synergy	PPRC1	chr10	103899869	103899869	C	G	exonic	nonsynonymous SNV	NM_001288727	5	c.C1604G	p.S535C		0.47798742	83	76	SNV
YHIM-07	Synergy	PRMT7	chr16	68363043	68363043	C	G	exonic	nonsynonymous SNV	NM_001184824	4	c.C207G	p.I69M		0.52808989	84	94	SNV
YHIM-07	Synergy	PROKR1	chr2	68882573	68882573	C	T	exonic	synonymous SNV	NM_138964	2	c.C1047T	p.T349T		0.48669202	135	128	SNV
YHIM-07	Synergy	PRPF40A	chr2	153527792	153527792	C	G	exonic	nonsynonymous SNV	NM_017892	14	c.G1474C	p.E492Q		0.64285714	15	27	SNV
YHIM-07	Synergy	PRRC2B	chr9	134340458	134340458	C	G	exonic	synonymous SNV	NM_013318	11	c.C1713G	p.V571V		0.27272727	216	81	SNV
YHIM-07	Synergy	PRUNE2	chr9	79321415	79321415	A	T	exonic	synonymous SNV	NM_001308047	8	c.T5775A	p.L1925L		0.32446809	254	122	SNV
YHIM-07	Synergy	PTPN22	chr1	114400377	114400377	C	G	exonic	nonsynonymous SNV	NM_001193431	5	c.G391C	p.E131Q		0.16184971	145	28	SNV
YHIM-07	Synergy	PTPRD	chr9	8524968	8524968	G	A	exonic	synonymous SNV	NM_001040712	6	c.C627T	p.S209S		1	0	421	SNV
YHIM-07	Synergy	PTPRZ1	chr7	121652359	121652359	G	A	exonic	nonsynonymous SNV	NM_002851	12	c.G3259A	p.E1087K		0.38716356	296	187	SNV
YHIM-07	Synergy	PYHIN1	chr1	158911851	158911851	G	A	exonic	nonsynonymous SNV	NM_152501	5	c.G664A	p.A222T		0.30627306	188	83	SNV
YHIM-07	Synergy	QPCTL	chr19	46205075	46205075	G	C	exonic	nonsynonymous SNV	NM_001163377	5	c.G624C	p.L208F		1	0	132	SNV
YHIM-07	Synergy	QRFP	chr9	133768968	133768968	G	T	exonic	nonsynonymous SNV	NM_198180	3	c.C258A	p.F86L		0.31014493	238	107	SNV
YHIM-07	Synergy	RAD51	chr15	41023318	41023318	C	T	exonic	nonsynonymous SNV	NM_001164269	10	c.C965T	p.P322L		0.02962963	262	8	SNV
YHIM-07	Synergy	RAD51D	chr17	33443953	33443953	C	T	exonic	nonsynonymous SNV	NM_001142571	3	c.G248A	p.G83E		0.50533049	232	237	SNV
YHIM-07	Synergy	RALA	chr7	39729994	39729994	A	G	exonic	nonsynonymous SNV	NM_005402	3	c.A128G	p.Y43C		0.70430108	55	131	SNV
YHIM-07	Synergy	RFPL1	chr22	29834903	29834903	C	T	exonic	synonymous SNV	NM_021026	1	c.C123T	p.P41P		0.51937985	186	201	SNV
YHIM-07	Synergy	RNASE8	chr14	21526219	21526219	C	T	exonic	synonymous SNV	NM_138331	1	c.C168T	p.I56I		0.46153846	154	132	SNV
YHIM-07	Synergy	RNF213	chr17	78355525	78355525	G	A	exonic	nonsynonymous SNV	NM_001256071	57	c.G13976A	p.R4659K	CCG	0.47863248	122	112	SNV
YHIM-07	Synergy	RNF38	chr9	36353252	36353252	G	A	exonic	nonsynonymous SNV	NM_194329	6	c.C836T	p.S279L		0.17117117	92	19	SNV
YHIM-07	Synergy	RPGRIP1	chr14	21792894	21792894	T	G	exonic	nonsynonymous SNV	NM_020366	14	c.T1880G	p.L627R		0.50446429	111	113	SNV
YHIM-07	Synergy	RRAGA	chr9	19050276	19050276	G	C	exonic	nonsynonymous SNV	NM_006570	1	c.G619C	p.E207Q		0.06309751	490	33	SNV
YHIM-07	Synergy	RRP15	chr1	218458764	218458764	G	A	exonic	nonsynonymous SNV	NM_016052	1	c.G106A	p.E36K		0.34857143	114	61	SNV
YHIM-07	Synergy	RSPO3	chr6	127469813	127469813	G	T	exonic	nonsynonymous SNV	NM_032784	2	c.G118T	p.G40C	CCG	0.488	128	122	SNV
YHIM-07	Synergy	RUBCN	chr3	197427669	197427669	G	C	exonic	nonsynonymous SNV	NM_001145642	7	c.C896G	p.S299C		0.26989619	211	78	SNV
YHIM-07	Synergy	RUBCN	chr3	197427668	197427668	G	T	exonic	synonymous SNV	NM_001145642	7	c.C897A	p.S299S		0.26896552	212	78	SNV
YHIM-07	Synergy	RUVBL1	chr3	127842523	127842523	G	A	exonic	synonymous SNV	NM_003707	1	c.C45T	p.I15I		0.36200717	178	101	SNV
YHIM-07	Synergy	RYR1	chr19	38935232	38935232	G	A	exonic	synonymous SNV	NM_000540	7	c.G546A	p.S182S		1	0	32	SNV
YHIM-07	Synergy	S1PR4	chr19	3179009	3179009	C	T	exonic	synonymous SNV	NM_003775	1	c.C219T	p.S73S		0.41132076	156	109	SNV
YHIM-07	Synergy	SALL1	chr16	51174590	51174590	C	G	exonic	nonsynonymous SNV	NM_001127892	2	c.G1252C	p.E418Q		0.37349398	104	62	SNV
YHIM-07	Synergy	SAMD14	chr17	48193052	48193052	G	A	exonic	nonsynonymous SNV	NM_001257359	7	c.C698T	p.S233F		0.47428571	92	83	SNV
YHIM-07	Synergy	SAMD4A	chr14	55203869	55203869	C	T	exonic	synonymous SNV	NM_015589	3	c.C843T	p.P281P		0.10714286	200	24	SNV
YHIM-07	Synergy	SAMHD1	chr20	35526358	35526358	G	T	exonic	stopgain	NM_015474	15	c.C1613A	p.S538X		0.10746269	299	36	SNV
YHIM-07	Synergy	SARDH	chr9	136568038	136568038	C	T	exonic	synonymous SNV	NM_001134707	13	c.G1668A	p.T556T		0.33557047	198	100	SNV
YHIM-07	Synergy	SCN2A	chr2	166245808	166245808	T	C	exonic	nonsynonymous SNV	NM_001040143	26	c.T5492C	p.I1831T		0.47588424	163	148	SNV
YHIM-07	Synergy	SCUBE1	chr22	43625189	43625189	C	T	exonic	nonsynonymous SNV	NM_173050	9	c.G973A	p.D325N		0.48717949	120	114	SNV
YHIM-07	Synergy	SDE2	chr1	226175838	226175838	C	A	exonic	nonsynonymous SNV	NM_152608	6	c.G893T	p.C298F		0.39925373	161	107	SNV
YHIM-07	Synergy	SEC14L1	chr17	75210034	75210034	G	A	exonic	nonsynonymous SNV	NM_001144001	15	c.G1975A	p.G659S		0.53424658	34	39	SNV
YHIM-07	Synergy	SEC61B	chr9	101984862	101984862	C	T	exonic	nonsynonymous SNV	NM_006808	2	c.C38T	p.S13F		0.25714286	78	27	SNV
YHIM-07	Synergy	SERPINA12	chr14	94964582	94964582	C	T	exonic	synonymous SNV	NM_001304461	2	c.G153A	p.Q51Q		0.47096774	82	73	SNV
YHIM-07	Synergy	SERPINF2	chr17	1657808	1657808	C	T	exonic	stopgain	NM_001165921	9	c.C1264T	p.Q422X		0.48571429	72	68	SNV
YHIM-07	Synergy	SETBP1	chr18	42532122	42532122	G	C	exonic	nonsynonymous SNV	NM_015559	4	c.G2817C	p.K939N	CCG	0.19553073	144	35	SNV
YHIM-07	Synergy	SETDB1	chr1	150923439	150923439	G	C	exonic	nonsynonymous SNV	NM_001145415	13	c.G2086C	p.E696Q		0.33333333	220	110	SNV

YHIM-07	Synergy	SF1	chr11	64535633	64535633	G	T	exonic	nonsynonymous SNV	NM_001178030	9	c.C1388A	p.T463K		0.40769231	77	53	SNV
YHIM-07	Synergy	SH3TC2	chr5	148407178	148407178	A	C	exonic	nonsynonymous SNV	NM_024577	11	c.T2117G	p.L706R		0.42241379	67	49	SNV
YHIM-07	Synergy	SKA1	chr18	47902279	47902279	A	C	exonic	synonymous SNV	NM_001039535	2	c.A79C	p.R27R		0.52380952	40	44	SNV
YHIM-07	Synergy	SKI	chr1	2237615	2237615	G	C	exonic	nonsynonymous SNV	NM_003036	6	c.G1924C	p.E642Q		0.51525424	143	152	SNV
YHIM-07	Synergy	SLC13A4	chr7	135390348	135390348	C	T	exonic	nonsynonymous SNV	NM_012450	5	c.G589A	p.E197K		0.06410256	146	10	SNV
YHIM-07	Synergy	SLC26A9	chr1	205901036	205901036	C	G	exonic	nonsynonymous SNV	NM_052934	5	c.G504C	p.E168D		0.29457364	91	38	SNV
YHIM-07	Synergy	SLC28A1	chr15	85438674	85438674	G	C	exonic	stoploss	NM_201651	7	c.G527C	p.X176S		0.61111111	14	22	SNV
YHIM-07	Synergy	SLC2A9	chr4	9998453	9998453	C	T	exonic	nonsynonymous SNV	NM_020041	3	c.G362A	p.G121E		0.39130435	14	9	SNV
YHIM-07	Synergy	SLC36A4	chr11	92881826	92881826	G	A	exonic	synonymous SNV	NM_001286139	11	c.C987T	p.F329F		0.46376812	37	32	SNV
YHIM-07	Synergy	SLC4A7	chr3	27473085	27473085	G	C	exonic	stopgain	NM_003615	7	c.C827G	p.S276X		0.46078431	55	47	SNV
YHIM-07	Synergy	SLFN12	chr17	33749085	33749085	G	T	exonic	synonymous SNV	NM_001289009	2	c.C963A	p.S321S		0.44559586	107	86	SNV
YHIM-07	Synergy	SMC1A	chrX	53431982	53431982	C	T	exonic	nonsynonymous SNV	NM_006306	13	c.G2158A	p.E720K		0.99492386	1	196	SNV
YHIM-07	Synergy	SMC1A	chrX	53430774	53430774	C	T	exonic	nonsynonymous SNV	NM_006306	14	c.G2248A	p.D750N		1	0	65	SNV
YHIM-07	Synergy	SMC1A	chrX	53431977	53431977	C	T	exonic	synonymous SNV	NM_006306	13	c.G2163A	p.Q721Q		0.9949495	1	197	SNV
YHIM-07	Synergy	SNRNP40	chr1	31769482	31769482	C	T	exonic	synonymous SNV	NM_004814	1	c.G117A	p.A39A		0.43564356	57	44	SNV
YHIM-07	Synergy	SNW1	chr14	78189570	78189570	C	G	exonic	nonsynonymous SNV	NM_012245	11	c.G1084C	p.E362Q		0.47027027	98	87	SNV
YHIM-07	Synergy	SNX19	chr11	130785713	130785713	A	C	exonic	nonsynonymous SNV	NM_014758	1	c.T122G	p.V41G		0.51515152	64	68	SNV
YHIM-07	Synergy	SNX4	chr3	125208304	125208304	C	G	exonic	nonsynonymous SNV	NM_003794	6	c.G601C	p.D201H		0.32911392	53	26	SNV
YHIM-07	Synergy	SNX5	chr20	17928271	17928271	T	C	exonic	nonsynonymous SNV	NM_001282454	11	c.A622G	p.T208A		0.55384615	29	36	SNV
YHIM-07	Synergy	SORBS1	chr10	97114642	97114642	T	C	exonic	synonymous SNV	NM_001034957	14	c.A1329G	p.K443K		0.51612903	30	32	SNV
YHIM-07	Synergy	SPATA20	chr17	48626419	48626419	G	A	exonic	nonsynonymous SNV	NM_001258372	5	c.G484A	p.G162R		0.52976191	79	89	SNV
YHIM-07	Synergy	SPATS2	chr12	49920021	49920021	G	A	exonic	nonsynonymous SNV	NM_001293286	13	c.G1621A	p.E541K		0.12	44	6	SNV
YHIM-07	Synergy	SPG20	chr13	36903566	36903566	C	A	exonic	nonsynonymous SNV	NM_001142294	4	c.G1097T	p.G366V		0.58367347	102	143	SNV
YHIM-07	Synergy	SPTAN1	chr9	131346147	131346147	G	C	exonic	nonsynonymous SNV	NM_001130438	16	c.G2092C	p.E698Q		0.35493827	209	115	SNV
YHIM-07	Synergy	SPTAN1	chr9	131346225	131346225	G	A	exonic	nonsynonymous SNV	NM_001130438	16	c.G2170A	p.E724K		0.29452055	103	43	SNV
YHIM-07	Synergy	SPTAN1	chr9	131346618	131346618	G	A	exonic	nonsynonymous SNV	NM_001130438	17	c.G2251A	p.D751N		0.32208589	221	105	SNV
YHIM-07	Synergy	SPTAN1	chr9	131378043	131378043	C	T	exonic	nonsynonymous SNV	NM_001195532	39	c.C5206T	p.R1736W		0.11061947	201	25	SNV
YHIM-07	Synergy	SSH3	chr11	67077339	67077339	G	T	exonic	stopgain	NM_017857	12	c.G1309T	p.E437X		0.1962963	217	53	SNV
YHIM-07	Synergy	ST8SIA2	chr15	92973285	92973285	G	T	exonic	synonymous SNV	NM_006011	2	c.G105T	p.S35S		0.04166667	138	6	SNV
YHIM-07	Synergy	STK11	chr19	1223131	1223131	C	T	exonic	synonymous SNV	NM_000455	8	c.C1068T	p.I356I	CCG	0.46296296	145	125	SNV
YHIM-07	Synergy	STRBP	chr9	125921478	125921478	T	C	exonic	synonymous SNV	NM_001171137	9	c.A690G	p.E230E		0.03608247	187	7	SNV
YHIM-07	Synergy	SVOPL	chr7	138313104	138313104	C	A	exonic	stopgain	NM_174959	7	c.G412T	p.G138X		0.52542373	28	31	SNV
YHIM-07	Synergy	SYNE2	chr14	64545306	64545306	A	T	exonic	nonsynonymous SNV	NM_015180	55	c.A11145T	p.K3715N		0.5	99	99	SNV
YHIM-07	Synergy	TAB2	chr6	149730854	149730854	G	C	exonic	stoploss	NM_001292034	7	c.G2081C	p.X694S		0.54187192	93	110	SNV
YHIM-07	Synergy	TAF12	chr1	28948566	28948566	T	C	exonic	nonsynonymous SNV	NM_001135218	2	c.A28G	p.I10V		0.57777778	19	26	SNV
YHIM-07	Synergy	TBC1D4	chr13	75915343	75915343	T	A	exonic	nonsynonymous SNV	NM_001286658	7	c.A1529T	p.E510V		0.4977169	110	109	SNV
YHIM-07	Synergy	TBC1D4	chr13	75915344	75915344	C	A	exonic	stopgain	NM_001286658	7	c.G1528T	p.E510X		0.50458716	108	110	SNV
YHIM-07	Synergy	TBC1D9B	chr5	179302955	179302955	C	A	exonic	nonsynonymous SNV	NM_015043	11	c.G1865T	p.R622L		0.56291391	66	85	SNV
YHIM-07	Synergy	TBCE	chr1	235605132	235605132	A	G	exonic	nonsynonymous SNV	NM_001287802	13	c.A935G	p.Y312C		0.65517241	50	95	SNV
YHIM-07	Synergy	TBX21	chr17	45821598	45821598	G	A	exonic	nonsynonymous SNV	NM_013351	4	c.G806A	p.R269Q		0.21666667	47	13	SNV
YHIM-07	Synergy	TECPR1	chr7	97858352	97858352	T	C	exonic	synonymous SNV	NM_015395	16	c.A2409G	p.Q803Q		0.38888889	11	7	SNV
YHIM-07	Synergy	TEKT4	chr2	95537547	95537547	G	A	exonic	nonsynonymous SNV	NM_144705	1	c.G223A	p.E75K		0.37974684	49	30	SNV
YHIM-07	Synergy	TESK1	chr9	35609044	35609044	G	A	exonic	nonsynonymous SNV	NM_006285	10	c.G1186A	p.D396N		0.20577164	633	164	SNV
YHIM-07	Synergy	TGFBR2	chr3	30713559	30713559	C	G	exonic	stopgain	NM_003242	4	c.C884G	p.S295X	CCG	1	0	208	SNV
YHIM-07	Synergy	TICRR	chr15	90166958	90166958	G	C	exonic	nonsynonymous SNV	NM_001308025	20	c.G3414C	p.Q1138H		0.30612245	136	60	SNV
YHIM-07	Synergy	TLE6	chr19	2994038	2994038	G	A	exonic	nonsynonymous SNV	NM_024760	15	c.G1190A	p.G397E		0.46478873	38	33	SNV
YHIM-07	Synergy	TMC4	chr19	54672266	54672266	-	GCC	exonic	nonframeshift insertion	NM_001145303	4	c.600_601insGG	p.P201delinsGP					Indel
YHIM-07	Synergy	TP53	chr17	7578235	7578235	T	C	exonic	nonsynonymous SNV	NM_001126115	2	c.A218G	p.Y73C	CCG	1	0	208	SNV
YHIM-07	Synergy	TRAPPC12	chr2	3392423	3392423	C	G	exonic	synonymous SNV	NM_016030	2	c.C1029G	p.L343L		0.45210728	143	118	SNV
YHIM-07	Synergy	TRAPPC8	chr18	29432575	29432575	C	T	exonic	nonsynonymous SNV	NM_014939	23	c.G3485A	p.R1162K		0.17045455	73	15	SNV
YHIM-07	Synergy	TRIM33	chr1	114964233	114964233	G	T	exonic	nonsynonymous SNV	NM_015906	11	c.C1886A	p.P629H	CCG	0.44811321	117	95	SNV
YHIM-07	Synergy	TRIM67	chr1	231344948	231344948	T	C	exonic	nonsynonymous SNV	NM_001004342	8	c.T2075C	p.V692A		0.28903655	214	87	SNV
YHIM-07	Synergy	TRIO	chr5	14485289	14485289	A	T	exonic	nonsynonymous SNV	NM_007118	47	c.A6769T	p.S2257C		0.34108527	255	132	SNV
YHIM-07	Synergy	TRPS1	chr8	116426279	116426279	A	G	exonic	nonsynonymous SNV	NM_001282902	6	c.T3830C	p.V1277A		0.04072398	212	9	SNV
YHIM-07	Synergy	TRPS1	chr8	116426278	116426278	C	T	exonic	synonymous SNV	NM_001282902	6	c.G3831A	p.V1277V		0.03686636	209	8	SNV
YHIM-07	Synergy	TTLL10	chr1	1119450	1119450	C	T	exonic	synonymous SNV	NM_153254	8	c.C1020T	p.L340L		0.45127119	259	213	SNV
YHIM-07	Synergy	TTN	chr2	179474255	179474255	C	T	exonic	nonsynonymous SNV	NM_003319	101	c.G24587A	p.R8196Q		0.18506494	251	57	SNV
YHIM-07	Synergy	TTN	chr2	179398551	179398551	A	G	exonic	nonsynonymous SNV	NM_003319	186	c.T75596C	p.L25199P		0.50581395	170	174	SNV
YHIM-07	Synergy	TXNDC11	chr16	11785286	11785286	G	A	exonic	nonsynonymous SNV	NM_015914	8	c.C1760T	p.S587L		0.46892655	188	166	SNV
YHIM-07	Synergy	UBA5	chr3	132390712	132390712	C	G	exonic	nonsynonymous SNV	NM_024818	7	c.C671G	p.S224C		0.13354037	279	43	SNV
YHIM-07	Synergy	UBN1	chr16	4925104	4925104	C	G	exonic	nonsynonymous SNV	NM_001079514	15	c.C2693G	p.S898C		0.13142857	152	23	SNV
YHIM-07	Synergy	UGGT2	chr13	96513091	96513091	G	C	exonic	nonsynonymous SNV	NM_020121	32	c.C3691G	p.L1231V		0.37704918	38	23	SNV

YHIM-07	Synergy	UNC45B	chr17	33476004	33476004	C	G	exonic	nonsynonymous SNV	NM_001308281	2	c.C181G	p.Q61E		0.44444444	105	84	SNV
YHIM-07	Synergy	UNC45B	chr17	33476003	33476003	C	A	exonic	synonymous SNV	NM_001308281	2	c.C180A	p.V60V		0.44680851	104	84	SNV
YHIM-07	Synergy	URB1	chr21	33717037	33717037	C	A	exonic	nonsynonymous SNV	NM_014825	24	c.G4099T	p.A1367S		0.45588235	111	93	SNV
YHIM-07	Synergy	USH2A	chr1	215953299	215953299	T	C	exonic	nonsynonymous SNV	NM_206933	55	c.A10825G	p.S3609G		0.31521739	126	58	SNV
YHIM-07	Synergy	USP34	chr2	61441453	61441453	G	T	exonic	synonymous SNV	NM_014709	68	c.C8424A	p.V2808V		0.06567797	441	31	SNV
YHIM-07	Synergy	UTP18	chr17	49354619	49354619	C	G	exonic	synonymous SNV	NM_016001	7	c.C966G	p.V322V		0.49321267	112	109	SNV
YHIM-07	Synergy	UTY	chrY	15471078	15471078	T	C	exonic	nonsynonymous SNV	NM_001258261	12	c.A1111G	p.S371G		1	0	7	SNV
YHIM-07	Synergy	VCAM1	chr1	101200273	101200273	T	A	exonic	nonsynonymous SNV	NM_080682	7	c.T1732A	p.S578T		0.49753695	102	101	SNV
YHIM-07	Synergy	VHL	chr3	10188232	10188232	C	A	exonic	nonsynonymous SNV	NM_000551	2	c.C375A	p.H125Q	CCG	0.47115385	165	147	SNV
YHIM-07	Synergy	VIT	chr2	37032607	37032607	A	T	exonic	nonsynonymous SNV	NM_001177970	12	c.A1081T	p.N361Y		0.5	48	48	SNV
YHIM-07	Synergy	VPS18	chr15	41192037	41192037	G	C	exonic	nonsynonymous SNV	NM_020857	4	c.G1021C	p.D341H		0.35014006	232	125	SNV
YHIM-07	Synergy	XIRP2	chr2	167992444	167992444	G	A	exonic	nonsynonymous SNV	NM_001079810	3	c.G434A	p.C145Y		0.4695122	87	77	SNV
YHIM-07	Synergy	YTHDC2	chr5	112917198	112917198	C	G	exonic	nonsynonymous SNV	NM_022828	25	c.C3439G	p.Q1147E		0.12548263	453	65	SNV
YHIM-07	Synergy	ZBTB20	chr3	114057962	114057962	C	T	exonic	nonsynonymous SNV	NM_001164342	5	c.G2116A	p.V706M		0.30654762	233	103	SNV
YHIM-07	Synergy	ZC3H12A	chr1	37948497	37948497	C	T	exonic	nonsynonymous SNV	NM_025079	6	c.C1085T	p.S362F		0.44660194	57	46	SNV
YHIM-07	Synergy	ZC3H12A	chr1	37948407	37948407	C	G	exonic	nonsynonymous SNV	NM_025079	6	c.C995G	p.S332C		0.38075314	148	91	SNV
YHIM-07	Synergy	ZFP82	chr19	36898816	36898816	C	A	exonic	stopgain	NM_133466	3	c.G109T	p.E37X		1	0	99	SNV
YHIM-07	Synergy	ZFYVE9	chr1	52732439	52732439	T	C	exonic	synonymous SNV	NM_004799	6	c.T2391C	p.A797A		0.05291005	179	10	SNV
YHIM-07	Synergy	ZIC3	chrX	136652205	136652205	T	C	exonic	synonymous SNV	NM_003413	3	c.T1380C	p.P460P		0.05952381	79	5	SNV
YHIM-07	Synergy	ZNF131	chr5	43161813	43161813	G	A	exonic	synonymous SNV	NM_001297548	5	c.G834A	p.E278E		0.09480122	296	31	SNV
YHIM-07	Synergy	ZNF486	chr19	20295173	20295173	G	A	exonic	synonymous SNV	NM_052852	2	c.G39A	p.L13L		0.38888889	44	28	SNV
YHIM-07	Synergy	ZNF547	chr19	57879935	57879935	G	T	exonic	nonsynonymous SNV	NM_173631	2	c.G12T	p.M4I		0.51094891	67	70	SNV
YHIM-07	Synergy	ZNF556	chr19	2877366	2877366	G	A	exonic	nonsynonymous SNV	NM_001300843	4	c.G407A	p.R136H		0.0960452	320	34	SNV
YHIM-07	Synergy	ZNF609	chr15	64915173	64915173	G	A	exonic	nonsynonymous SNV	NM_015042	2	c.G895A	p.E299K		0.10408922	241	28	SNV
YHIM-07	Synergy	ZNF644	chr1	91406040	91406040	T	A	exonic	stopgain	NM_201269	3	c.A871T	p.R291X		0.50735294	67	69	SNV
YHIM-07	Synergy	ZNF675	chr19	23837355	23837355	C	G	exonic	nonsynonymous SNV	NM_138330	4	c.G380C	p.G127A		0.36440678	75	43	SNV
YHIM-07	Synergy	ZNF806	chr2	133075023	133075023	C	T	exonic	stopgain	NM_001304449	4	c.C484T	p.Q162X		0.20788531	221	58	SNV
YHIM-07	Synergy	ZXDC	chr3	126194425	126194425	T	A	exonic	nonsynonymous SNV	NM_001040653	1	c.A284T	p.E95V		0.34615385	119	63	SNV
YHIM-07	Synergy	ZXDC	chr3	126180894	126180894	G	C	exonic	nonsynonymous SNV	NM_001040653	6	c.C1611G	p.I537M		0.31147541	126	57	SNV
YHIM-07	Synergy	ZXDC	chr3	126194426	126194426	C	A	exonic	stopgain	NM_001040653	1	c.G283T	p.E95X		0.32631579	128	62	SNV
YHIM-07	Synergy	ADGRL2	chr1	82402522	82402522	G	A	splicing	splicing	NM_001297704	5	c.397+1G>A			0.46666667	40	35	SNV
YHIM-07	Synergy	EIF4G3	chr1	21144032	21144032	C	G	splicing	splicing	NM_001198801	32	c.4309-1G>C			0.60606061	26	40	SNV
YHIM-07	Synergy	IARS	chr9	94984955	94984955	C	T	splicing	splicing	NM_002161	33	c.3554-1G>A			0.05208333	91	5	SNV
YHIM-07	Synergy	MINK1	chr17	4789261	4789261	G	A	splicing	splicing	NM_015716	8	c.694+1G>A			0.60655738	48	74	SNV
YHIM-07	Synergy	NF2	chr22	30077427	30077427	G	C	splicing	splicing	NM_181829	14	c.1452-1G>C			0.45652174	25	21	SNV

Fig. S1.

Venn diagrams showing overlapping somatic mutations (single-nucleotide variants and insertions/deletions) for samples YHIM-01 and YHIM-02 between patient-derived (F0) and second-generation tissues (F2), using targeted deep sequencing of five patient-derived xenograft (PDX) models and corresponding patient tumor samples.

Fig. S2.

Representative histology and immunohistochemistry for seven patients and patient-derived xenografts (PDXs). SCCHNs are typically positive for p63. Magnification 200× in patient samples.

Fig. S3

(*A*) Mutational spectrum in patient tumors detected by targeted sequencing. Samples with a greater than 1% incidence of genetic alterations are shown and are stratified by response to buparlisib therapy. PR, partial response; SD, stable disease; PD, progressive disease. (*B*) Gene diagrams for key mutations in potentially targetable gene, TP53.

Fig. S4

(*A*) Gene expression profiles for buparlisib, cetuximab, buparlisib/cetuximab, and control in three PDX models (YHIM-01, -02, -03, -04), showing upregulation of genes associated with apoptosis and cell cycle arrest by combination therapy. (*B*) Comparison of cell viability by treatment (buparlisib, cetuximab, buparlisib/cetuximab, and control) using two cell lines from PDX models YUX-01 and -02. (*C*) Expression of apoptosis-related genes (cleaved caspase-9, Bcl-2, and Myc) in YUX-01 and -02. (*D*) Flow cytometry showing apoptosis in YUX-01 and -02.
